# Genomic selection for white spot syndrome virus resistance in whiteleg shrimp boosts survival under an experimental challenge test

**DOI:** 10.1038/s41598-020-77580-3

**Published:** 2020-11-25

**Authors:** Marie Lillehammer, Rama Bangera, Marcela Salazar, Sergio Vela, Edna C. Erazo, Andres Suarez, James Cock, Morten Rye, Nicholas Andrew Robinson

**Affiliations:** 1 Breeding and Genetics, Nofima, 1430 Ås, Norway; 2 Benchmark Genetics Norway AS, 6600 Sunndalsøra, Norway; 3 Benchmark Genetics Colombia, Bogota, Colombia; 4 Sustainable Aquaculture Laboratory-Temperate and Tropical (SALTT), School of BioSciences, The University of Melbourne, Parkville, 3010 Australia

**Keywords:** Animal breeding, Heritable quantitative trait, Quantitative trait

## Abstract

White spot syndrome virus (WSSV) causes major worldwide losses in shrimp aquaculture. The development of resistant shrimp populations is an attractive option for management of the disease. However, heritability for WSSV resistance is generally low and genetic improvement by conventional selection has been slow. This study was designed to determine the power and accuracy of genomic selection to improve WSSV resistance in *Litopenaeus vannamei*. Shrimp were experimentally challenged with WSSV and resistance was evaluated as dead or alive (DOA) 23 days after infestation. All shrimp in the challenge test were genotyped for 18,643 single nucleotide polymorphisms. Breeding candidates (G_0_) were ranked on genomic breeding values for WSSV resistance. Two G_1_ populations were produced, one from G_0_ breeders with high and the other with low estimated breeding values. A third population was produced from “random” mating of parent stock. The average survival was 25% in the low, 38% in the random and 51% in the high-genomic breeding value groups. Genomic heritability for DOA (0.41 in G_1_) was high for this type of trait. The realised genetic gain and high heritability clearly demonstrates large potential for further genetic improvement of WSSV resistance in the evaluated *L. vannamei* population using genomic selection.

## Introduction

Disease caused by the White Spot Syndrome Virus (WSSV) is a serious problem for global shrimp aquaculture. It infects all major cultured shrimp species and is highly virulent normally causing death in a few days^1^. Cultivation practices, mostly aimed at eliminating the causal agent, may reduce the economic risks associated with white spot disease^2^, but elimination in open pond systems is often impossible^3^. The main strategies used worldwide to cope with on farm viral infection involve the use of biosecure protocols and fast-growing WSSV pathogen-free but susceptible shrimp in a race to harvest before infection and death occurs^4–6^ or growth of shrimp when water temperatures are higher and the disease does not prosper^2^. Elimination strategies can be effective if used in combination with good management practices^7^, but shrimp often succumb rapidly to disease and provision of WSSV resistant, pathogen-free stocks would greatly benefit the industry.

Shrimp lack an adaptive immune system, relying on the innate immune system to prevent, tolerate and clear infection see review of crustacean immunity^8^. Hence, several conventional approaches used in other livestock, such as vaccination to boost the immune response and provide protection, have had limited success for shrimp. Although stimulation of the innate immune system through immune priming to prevent and control specific diseases in shrimp shows promise, the efficacy of such methods have not yet been proven under field conditions^9–11^. Knowledge about the defensive response to infection is building, but the specific mechanisms associated with greater resistance or tolerance to disease in specific populations or individuals have not been conclusively identified. Nevertheless, there is substantial information that can be used to guide breeders in their efforts to produce resistant stocks. Estimates of the heritability of WSSV resistance in *Litopenaeus vannamei* range between 0.01 and 0.31 depending on the batch of shrimp, the trait analysed (e.g. days survival or binary dead or alive), the challenge method applied and statistical models used for genetic parameter estimation^12–16^. The simultaneous improvement of growth and WSSV resistance with traditional index selection is complicated by a negative genetic correlation (− 0.55 to − 0.64) between these traits^12,16^. The heritability for white spot viral copy number (0.18) and pond survival (0.16) in the face of WSSV farm outbreaks is low^16,17^. The genetic gain after selection depends on the heritability of the trait selected in that population and its phenotypic variance (genetic variance), the accuracy of the breeding value estimation and on the intensity of selection applied. The reported genetic gain per generation for WSSV resistance ranges from 1.7 to 6.5%^16,18,19^.

If genetic loci with a large effect on WSSV resistance exist, then marker assisted selection (MAS) could be an efficient way to boost resistance. A gene set based association analyses for WSSV resistance (dead/alive) in *L. vannamei* identified a total of 5 SNPs within immune related genes, however, these SNPs were not validated further in a larger population for its application in MAS^20^. Robinson et al.^21^ identified several linkage groups containing quantitative trait loci associated with time to death after WSSV infection in black tiger shrimp (*Penaeus monodon*). However, as the estimated marker effects on hours survival were relatively small, WSSV resistance is likely to be a typical quantitative trait, affected by the additive effects of many genes of small effect^22^. Many of the single nucleotide polymorphisms (SNPs) that mapped close to the loci associated with survival occurred near transcripts with homology to putative innate immune genes of interest. For instance, c-type lectin protein which may be involved in immune recognition and microorganism phagocytosis^23^, is more highly expressed in resistant than susceptible shrimp exposed to WSSV^24–27^. This gene was mapped to a QTL region on linkage group 43.

Genomic selection^28,29^ has been proposed as the most effective form of selection available for typical quantitative (polygenic) traits^30^. Hence, we surmised that genomic selection could be used to improve the genetic gain in WSSV resistance. In this study we experimentally challenged families of white shrimp with WSSV virus and used single nucleotide polymorphism genotype data from challenged and candidate breeders to evaluate the power and accuracy of genomic selection for improving WSSV resistance. Breeding candidates (G_0_) were ranked in terms of genomic breeding values for WSSV resistance. The breeding candidates were mated to produce two G_1_ populations, one with high and the other with low genomic estimated breeding values. The survival of G_1_ populations and offspring from “randomly” mated parent stock was compared in a challenge test.

## Results

Filtering of the 465,500 putative SNPs identified from mRNAseq resulted in 10,323 SNPs (Table S1) which were selected for inclusion in the custom 18 K Illumina SNP array (MAF > 0.1, > 51X coverage and eliminating multiple SNPs per contig and SNP alleles fixed in the R- and S-lines). Of the total 19,290 SNPs printed on the chip, post-genotyping quality control left genotypes for 18,643 SNPs that were deemed acceptable for genomic estimated breeding value (gEBV) prediction.

**Figure 1:**
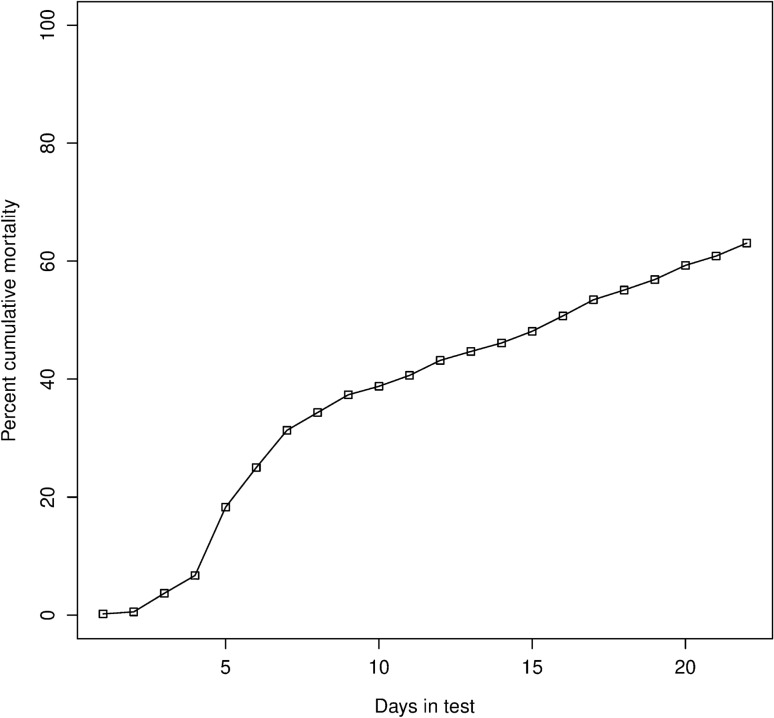
Cumulative mortality observed with WSSV infection among G_0_ training animals. The total number of animals evaluated in the challenge test was 1459.

In the G0 challenge test mortality increased rapidly up to around day five as expected and then, instead of the usual flattening off, a few animals continued to die each day until the test ended (Fig. 1). The test was ended at 22 days according to standard practices, even though some animals were still dying, to facilitate consistent comparisons between G0 and G1.

Estimates of heritability for the dead or alive (DOA) and days survival (DS) traits were similar for the overall G_0_ population (0.30 for DOA and 0.53 for DS) to those for the pure R-line animals (DOA 0.22 and DS 0.39) and for crossed resistant by susceptible line (RxS) animals (DOA 0.34 and DS 0.48, Table 1). Similar estimates were obtained both with and without the fixed effect of line included in the analysis. The fixed effect of line revealed 0.19 higher survival probability for purebred RxR than crossbred RxS. For the DS trait, the effect of being purebred was 2.71 days longer survival.

**Table 1:** Estimated variance components and heritabilities for both traits from analysing the G_0_-data together or each genetic group (purebred or crossbred) separately.

Data	DOA			DS		
	Phenotypic variance	Genetic variance	Heritability	Phenotypic variance	Genetic variance	Heritability
All data	0.23	0.07	0.32 ± 0.05	63.1	34.6	0.55 ± 0.05
Pure RxR	0.26	0.056	0.22 ± 0.06	58.4	22.5	0.39 ± 0.07
RxS cross	0.18	0.061	0.34 ± 0.07	57.6	27.7	0.48 ± 0.08

Genetic variation within the pure resistant line (R-line) and crossed RxS-line animals was found to be substantial (0.056 for DOA in the pure R-line, 0.061 for DOA in the crossed RxS line, and 22.5 for DS in the pure R-line and 27.7 for DS in the crossed RxS line individuals) so that a good selection response could be expected. This is supported by the accuracy of breeding values, estimated by cross validation of the full dataset, which was 0.69 for DOA and 0.64 for DS.

### Power analysis

The power analysis indicated that the minimum number of selected parents in the high resistance group should be 30–40 males and females when there are 20 males and females in the random group (Table 2).

**Table 2:** Power of experiment to evaluate genomic selection assuming one male is mated with one female to generate each separate family.

Number of families			Power	
High-gEBV	Random-gEBV	Low-gEBV	High-gEBV vs Random-gEBV	High-gEBV vs Low-gEBV
40	10	10	0.83	1
30	20	10	0.96	1
40	20	0	0.96	
50	20	0	0.95	

### Evaluation of genomic selection on G_1_

Out of the 1885 animals tested, 1 family of 35 siblings (random-gEBV group) was removed due to missing parental data, 3 animals could not be confirmed to be derived from the parents used in the experiment, leaving 1847 records from 59 G_1_ families (32 high-, 19 random- and 8 low-gEBV) for analysis.

**Figure 2:**
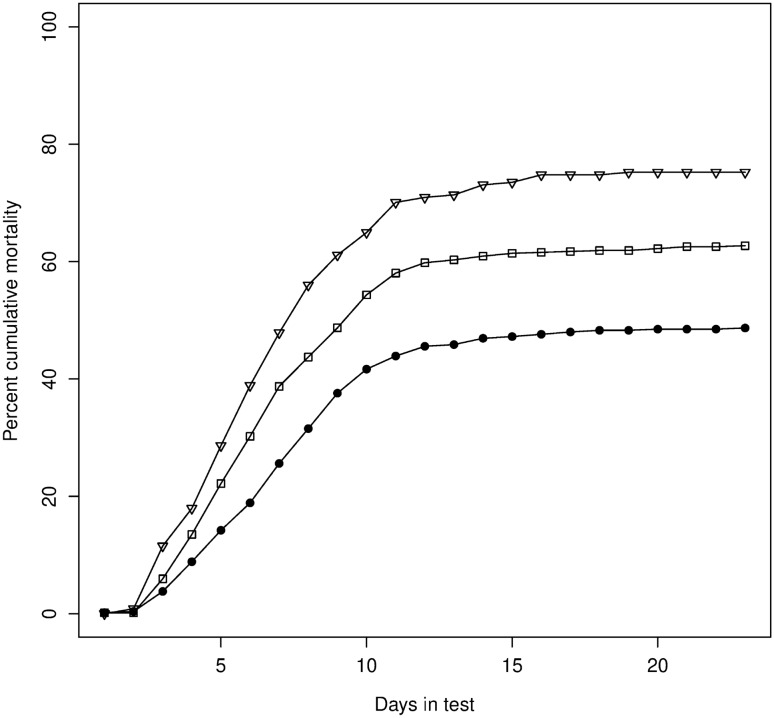
Percent cumulative mortality plotted over days in challenge test for G_1_ animals in the high—(closed circles), random—(open squares) and low—(open triangles) gEBV groups. The total number of animals evaluated in the challenge test was 1883 (high 1027, random 622 and low 234).

Cumulative mortality increased at a slower rate and plateaued at a lower level in the challenge test for the high gEBV compared to random and low gEBV groups (Fig. 2). The low gEBV group had the fastest rate of mortality and reached the highest levels of cumulative mortality of the three groups (cumulative mortality at the end of the test of 75% in the low, 63% in the random and 49% in the high gEBV group), giving a 13% improved rate of survival in the high gEBV group. The G_1_ random gEBV group reached the same final cumulative mortality during the test as was observed in the G_0_ training population (63%, Fig. 1).

**Figure 3:**
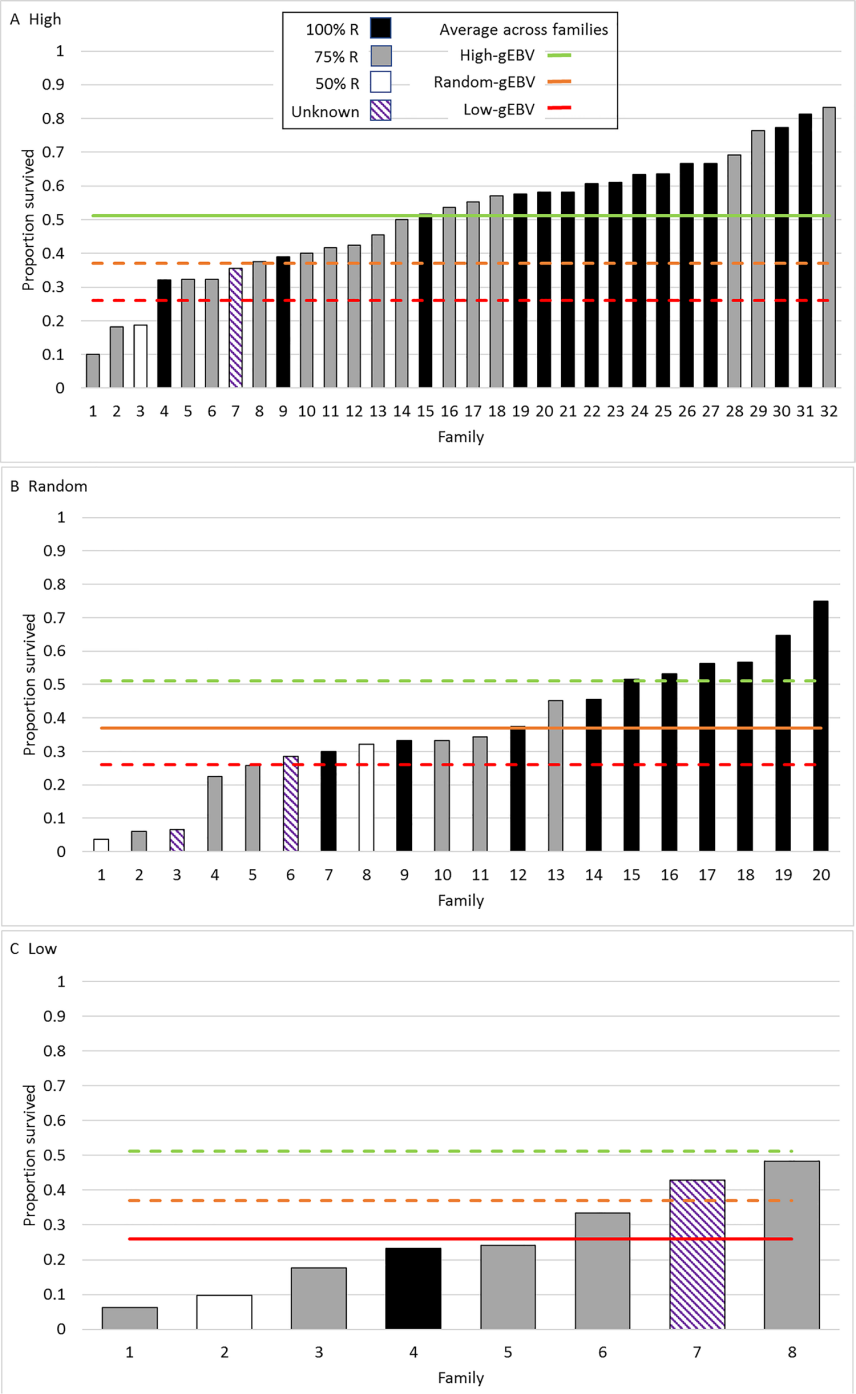
Proportion surviving in each challenged family for high (A) random (B) and low (C) gEBV selected G_1_ groups. Shading of the bar plots indicates % of resistant line ancestry in that family (black 100%, grey 75%, white 50% and stripes when the population origin of one parent was unknown. Horizontal lines show the average proportion across families surviving for the high (green), random (orange) and low (red) gEBV G_1_ groups. Solid lines relate to the G_1_ population that is represented in each plot.

Average survival of families in the high gEBV group at 51% was significantly greater than that of the random group (38%) which was, in turn, significantly greater than the 25% survival of the low gEBV group (Fig. 3). The range of survival within families was large, with the greatest variation in the high-gEBV families, which also had the largest range of average family survival. Families with the highest proportion of offspring surviving inherited a greater proportion of the resistant line genes (100% versus 75% or 50%), across the high-, random- and low-groups (Fig. 3). No families from the low group had greater than 50% survival whereas half the high group had greater than 50% survival with four of these families, out of a total of 32, with more than 70% survival.

**Figure 4:**
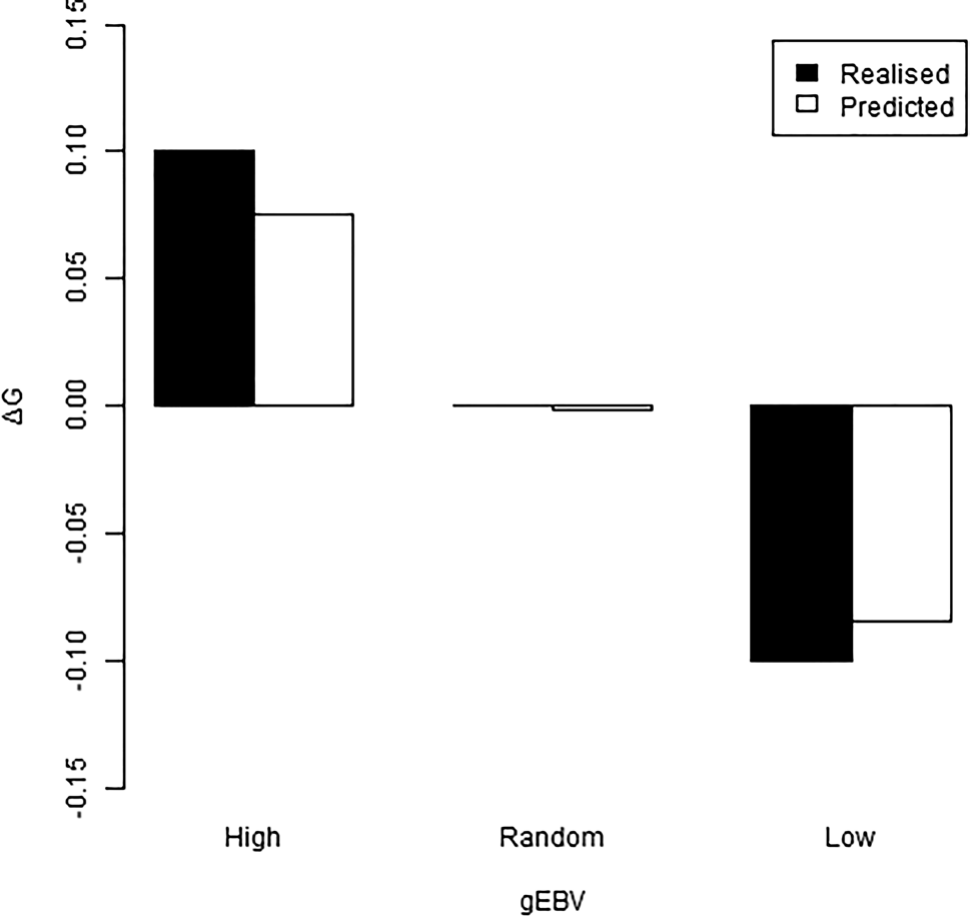
Effect of genomic selection showing realised and predicted genetic gain (ΔG) in the DOA trait for high, random and low gEBV selection groups.

The heritability of DOA in the G_1_ population (Table 3) was greater than those for the G_0_ population (Table 1), regardless of whether the effect of selection group was fitted in the model or not. The estimated realised response to selection was greater than predicted (Fig. 4, assuming heritability for DOA of 0.3 as calculated for the G_0_, Table 1), and the phenotypic differences between the groups were larger than the statistically estimated group effect. Tank was found to have a low but significant effect on DOA and was therefore included in the analysis model as a fixed effect.

**Table 3:** Estimated variance components and heritabilities for “days or alive” from analysing the G_1_-data with or without selection group fitted as a fixed effect.

Model	Phenotypic variance	Genetic variance	Heritability
With selection group fitted	0.26	0.11	0.41 ± 0.09
Without selection group fitted	0.27	0.13	0.47 ± 0.09

## Discussion

Average survival in the G_1_ population increased from 38 to 51% after one generation of genomic selection for high WSSV resistance for the dead or alive binary trait (DOA) relative to average survival in G_1_ randomly selected shrimp (Fig. 4). Selection for low WSSV resistance gave a very similar response in the opposite direction. This clearly demonstrates the power of genomic selection as a tool to develop WSSV resistant *L. vannamei* shrimp using this particular “synthetic” population which contains large variation for WSSV resistance. Due to the large variation in the DOA trait, and relative high heritability, further selection to produce a G_2_ would be expected to produce greater genetic gains than evaluated with production of the G_1_.

Greater genetic gains for the days survival (DS) trait, with genomic heritability (h^2^_DS_) of 0.55 and additive genetic variance (V_A DS_) of 34.6, would be expected than that for DOA (h^2^_DOA_ of 0.32, V_A DOA_ of 0.07) (Table 1). As a consistently strong positive correlation between DS and DOA is observed in the literature (e.g. r > 0.8)^31^ improvement in one trait is likely to result in improvement of the other. This was confirmed in our study, as the shrimp from the group selected for high resistance survived 2 days longer than the randomly selected shrimp, which again survived 2 days longer than the group selected for low resistance (results not shown). Moreover, farmers prefer shrimp populations that survive to those that merely take a few days more to die in the presence of WSSV. Hence, we suggest that genomic selection for WSSV resistance should be based on the DOA trait and not the DS trait despite a potentially faster improvement if DS were to be the trait selected.

Cross-validation, by elimination of individual values and comparison with the predicted value from the remaining data, has been used before to predict the accuracy of genomic selection e.g.^32^. The accuracy of genomic predictions through cross-validation has been reported in *L. vannamei* for resistance to bacterial disease^20^, feed efficiency traits^33^ and growth rate^34,35^. Nevertheless, cross-validation only provides an estimate or prediction of the likely genetic gain. The realized genetic gain is a direct measure of the genetic gain and of the effectiveness of a selection method. Hence, we prefer realized genetic gain to cross validation as a means of evaluating distinct selection methods. We have for the first time measured the realized genetic gain from genomic selection for disease resistance in a crustacean species, adding to the earlier evaluation of realized genetic gain from genomic selection for disease resistance in another aquatic species, rainbow trout^36^.

We used power calculations to design a selection experiment to: (1) give power > 0.95 to detect the selection response we expect from the estimated heritability and selection differential and (2) give as many high resistant families as possible, suitable for further breeding for increased resistance. To achieve this, we aimed to produce 30 high resistance, 20 random and 10 low resistance families. However, not all candidates matured and became available to make the planned crosses. Due to lack of available breeder candidates we produced 32 high, 20 random and 8 low resistance families. The predicted selection response, based on the heritability estimated from the G_0_ and the actual selection differential after mating (i.e. the average breeding value of the parents within each selection group) was 0.075 for the selected resistant group and − 0.084 for the selected susceptible group. However, the realized response to selection was higher than predicted (Fig. 4), and statistically significant (*p* < 0.05). The actual difference in survival between the groups in G_1_ was 0.13 in each direction. The statistical model used to analyse the G_1_ data gave an estimated effect of selection of 0.1, i.e. implying that survival should be 0.1 higher or lower in the selection groups than in the random group. However, the estimated response to selection is probably under-estimated because the model aims to distinguish between the effect of genetics and the effect of selection, while these effects are by nature confounded. As this was a controlled challenge test with few other factors to correct for, we suggest the phenotypic differences between the groups are a good estimate of genetic gain.

Heritability estimates differed between G_0_ and G_1_ and this could explain the discrepancy between predicted and realised selection response, if the predicted response was based on an underestimate of genetic variance. Our experience is that few new mortalities occur after day 15 of the challenge test (as was observed for the challenge of G_1_ animals) and the test is normally ended at 22 days. The sustained low level of mortality until the end of test in the G_0_ population was abnormal and could be attributed to a lower than normal viral load in the infecting tissue (which was obtained from a population of resistant animals). It is not clear how ending the test before mortalities ceased would affect the genetic parameter estimates for DOA and DS, however this discrepancy could explain the heritability differences between G_0_ and G_1_. If we estimate expected response based on the heritability estimate from the G_1_-population, this estimate is similar to the estimated regression coefficient for selection group, but still below the actual realized genetic gain, measured as the phenotypic difference between the selected group and the control group.

In general, our estimates for heritability of WSSV resistance based on genomics were greater than the estimates obtained from previous conventional evaluations^12–16^. The higher heritability found in G_1_ than in G_0_ could be due to a difference in true heritability, because of differing test conditions and/or differing genetic composition between the populations. It could however also be due to estimation inaccuracies with one estimate being closer to the true heritability than the other. Possible non-genetic effects common to full-sibs were not fitted, due to confounding with the genetic effect, and could cause over-estimation of the genetic variance. However, as the tested shrimp were young and mixed as soon as they reached a suitable size for tagging, we expect the common effect of full-sib to be small. The test conditions were controlled and standardized and hence should not have caused large differences between the two consecutive generations, although random variation in disease pressure and other environmental factors could still exist. The structures of the two populations tested were however quite different, which could affect the heritability. The G_0_-population consisted of a mix of purebred resistant line animals and crossbred resistant-by-susceptible line animals. The difference in susceptibility between these two genetic groups were fitted as a fixed effect in the analysis model, but the genetic variance was assumed to be the same within these two populations, even though natural selection for resistance in the purebred line might have reduced heritability. The G_1_ consisted of animals with various levels of strain composition, and the G_1_ also consisted of sub-groups that were selected for high or low resistance. The studied populations would therefore be expected to have unusually high genetic variance for resistance as they constituted a combination of strains with different selection history and due to the application of divergent genomic selection. Statistical corrections made to account for strain in G_0_ and selection group in G_1_ lead to downward bias in the heritability estimate, as the between-group genetic variance is fitted as a fixed effect. The argument for making this correction is that the genetic variance within the mixed population is not representative of most breeding populations. Analyses of G_1_ with and without correction for selection group shows that roughly 10% of the genetic variance exists between the groups, while 90% of the genetic variance is contained within group. This high within group genetic variance, and generally higher heritability with genomics than with conventional methods, suggests that further genomic selection to produce a G_2_ should achieve similar levels of genetic progress as documented for the G_1_.

Our finding of similar estimates of heritability for the overall G_0_ population, pure R-line animals and crossed RxS-line animals, and our findings of an intermediate level of WSSV resistance in crossed RxS-line animals between that found in the pure R and pure S-lines, indicates additive effects of alleles controlling resistance for both the DOA and DS traits. In this study the role of dominance or heterosis could not be elucidated as the pure S-line could not be compared in the same test (the S-line being highly susceptible). Hence, we could not identify possible non-additive genetic effects from these experiments.

Several factors were identified that make genomic selection for WSSV resistance in *L. vannamei* attractive. First, the methodology based on genomics identified genetic variation in resistance in the initial G_0_-population. Our previous trials characterising the starting populations show that hybrid lines contain a level of WSSV resistance somewhere between that of pure R-line and S-line animals, indicating possible additive effects of alleles promoting resistance. Second, the detection of significant differences between the selection groups shows that selection increased WSSV resistance in this first generation of selection. Third, broad variation was detected within groups, particularly in the high-gEBV group. This most likely reflected that the proportion of the R-line in the high-gEBV group which could, in theory, range from 0 to 100% (parents being a mixture of pure R-line and RxS-line individuals). The significant genetic variance remaining in the G_1_, including when selection group was accounted for in the model, coupled with higher heritability in G_1_ than in G_0,_ indicating no loss of genetic variation for resistance due to selection, indicate that further improvement through selection should be possible with a continued selection response over several generations. Fourth, the selection response was greater than expected from the selection intensity applied, estimated heritability and additive genetic variance. Finally, the 13% improved survival in one generation is greater than with conventional selection methodologies and the genetic gain is commercially interesting.

The survival levels reported here of slightly more than 60% in the best populations may not appear as satisfactory for commercial production. However, when vaccination is used as means of protecting populations against epidemics, the population can be effectively protected without vaccinating every individual due to the herd effect, see for example^37^. Anche et al.^38^ suggest that the basic reproduction ratio, R, is the key parameter determining the risk and severity of infectious diseases. Furthermore, they suggest that genetic improvement of R_0_ could be used for control of infectious diseases in host populations. Hence, the herd effect of resistant animals could have a similar effect to that of partial vaccination if resistance is associated with a reduced R. In the challenge tests for resistance we used, the animals were not individually infected but rather were maintained in an environment which encouraged reproduction of the disease. Thus, we suggest that not only were we selecting for resistance, but also for a low, desirable, R. Re-analysis of data on WSSV and Taura (TSV) epidemics in shrimp, suggest that the R of Taura was reduced by breeding to a level that contained the disease, whilst in the case of WSSV the reduction was not enough to control the disease^39^. Furthermore, they suggest that heritability of resistance is not enough on its own to evaluate the economic gain of selection for resistance to a specific disease, because it ignores the fact that resistant animals no longer infect other animals. The inoculum pressure with commercial production is almost certainly much lower than in our challenge tests in which several families now show survival of 70%. Anche et al.^38^ point out that levels of about 70% survival were sufficient in the case of Taura to maintain the disease under control. Hence, in a similar manner that disease epidemics can be slowed or stopped when a certain level of the population is vaccinated, a certain level of resistant animals in a population, coupled with practices that disfavour the disease, may reduce R to close to one and be sufficient to control WSSV in commercial populations. We have shown that by using genomic selection we can rapidly increase the level of resistance and hopefully will be able to use this tool to offer growers shrimp populations that can survive and produce in the presence of WSSV without having to totally eradicate the causal organism.

In applying genomic selection for disease resistance we also need to consider that an unfavourable genetic correlation between growth rate and WSSV resistance exists^16,40^ and that a faster growth rate is desirable for shrimp aquaculture. Simultaneous improvement of both traits could be achieved by combining a fast-growing S-line with high resistance R-line in a multiplier design for commercial broodstock supply. It may also be possible to use genomic selection in such a way that both traits are positively selected, but this needs further investigation. Another consideration for application is affordability and accessibility. The need for genotyping will add costs to the breeding program, but it may also reduce the need for systematic pedigree recording, which is costly and also induces inefficiencies in the selection process due to separate rearing, as that can be derived from the genotypes. Many SNP markers have already been identified for *L. vannamei*^41^ and technologies for ultra-high throughput genotyping of dense markers are becoming more readily available and less costly to apply^42^.

The other major difficulty faced when selecting for WSSV resistance in shrimp is that it is practically impossible to use survivors of challenge tests as breeding candidates as they can vertically transmit the virus and infect the offspring population. Furthermore, the practical difficulties of cleaning survivors to incorporate them into breeding programs which produce WSSV-specific pathogen free broodstock are immense. The practical impossibility of using survivors, and the lack of information on survival performance of breeding candidates and their true genetic relationships with candidates (pedigree relationships have been the standard for previous studies), limits the accuracy of conventional phenotypic selection. These problems also limit the selection intensity and the genetic variance that can be utilized. Greater accuracy for the prediction of breeding values can be gained by obtaining genomic relationships from the dense SNP genotype data. Genome-wide genetic markers make it possible to estimate the degree of genomic relationship between test-animals and individual candidate breeding animals within families. Whereas conventional pedigree relationships estimate the average relationship across the genome, dense SNP data enables estimation of genetic relationships at each position in the genome. The genomic relationship between every animal, and performance data from challenge tested animals, is used to estimate individual genomic breeding values: thus, combined family and individual selection is possible with genomic selection. We contend that the ability to evaluate the individual breeding value through genomic selection is the main reason why a higher rate of genetic gain was obtained with genomic selection (13% improved survival per generation) than that achieved with conventional phenotypic selection of WSSV resistance in *L. vannamei* 1.7% DOA, 6.3% survival and 6.5% survival selection reponses per generation^16,18,19^.

## Conclusion

We have shown that significant useful genetic improvement for WSSV resistance can be achieved in a breeding program for *L. vannamei* by applying genomic selection. Compared with conventional methods the use of genomic data resulted in higher heritability estimates and improved the accuracy of selection to levels that are commercially relevant. From a genomic selection experiment, and from cross-validation accuracy predictions, we demonstrated that the moderate marker density of ~ 18 K used was sufficient for accurate genomic breeding value prediction. This is the first study in *L. vannamei* to demonstrate realized genetic gain from genomic selection. Genomic selection is particularly promising for selection programs that challenge live animals with infectious agents when survivors cannot later be used as breeders.

## Materials and methods

### Animals used for the study

**Figure 5:**
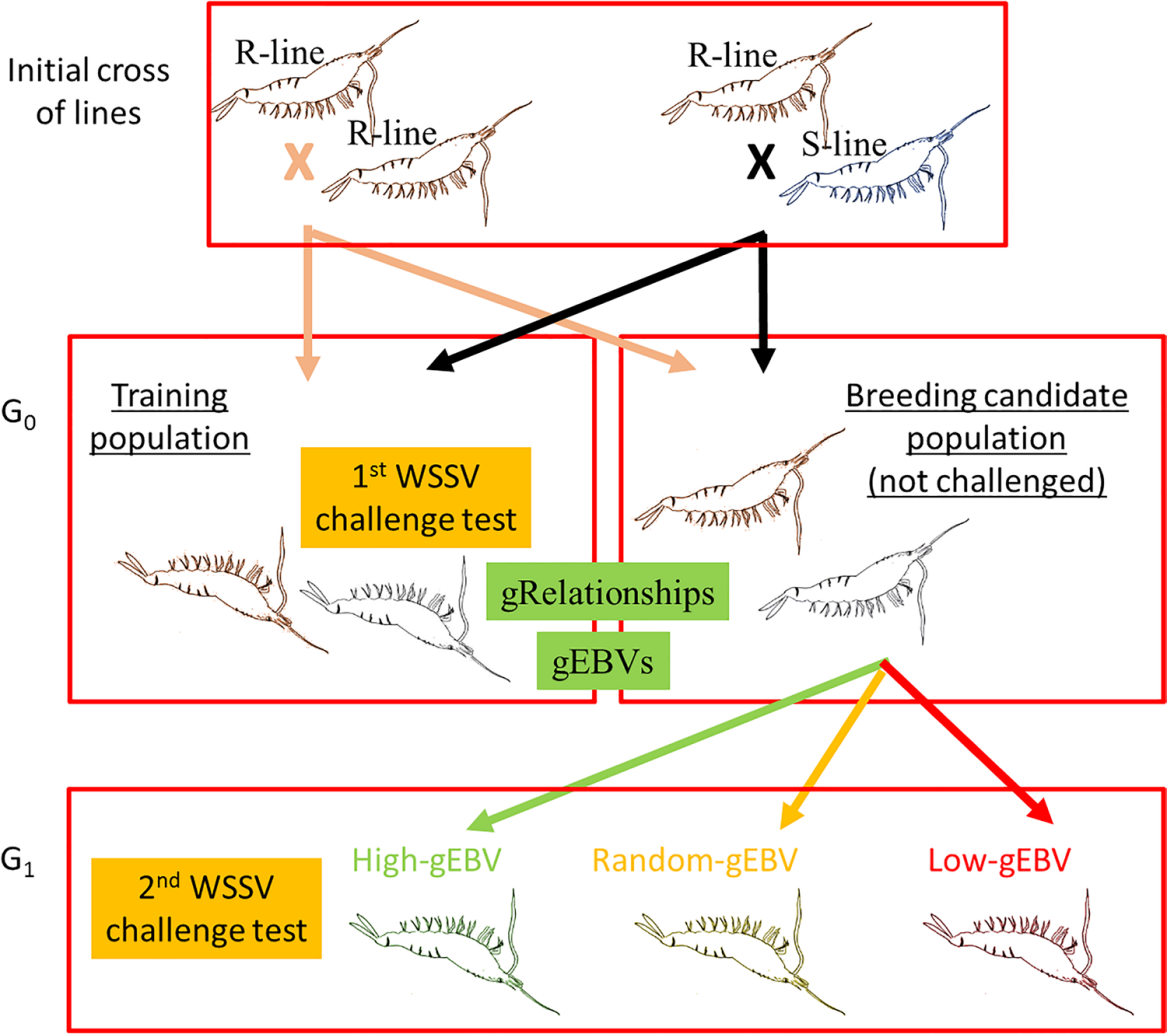
Origin of shrimp for the experiment. The animals were split into training and candidate breeding populations for estimation of white spot syndrome virus (WSSV) resistance genomic estimated breeding values (gEBV) at generation G_0_. G_0_ breeding candidates were selected and mated to produce high, random and low gEBV G_1_ groups whose performance (survival after an experimental challenge test) was compared for the final evaluation of the power of genomic selection.

Two populations of whiteleg shrimp (*Litopenaeus vannamei*) that had been selectively bred for several generations by Benchmark Genetics (formerly Ceniacua) in Colombia were utilised for the study (Fig. 5). The first population was derived from a breeding operation on the Atlantic Coast of Colombia that commenced in 1997. This population has not been exposed to WSSV and is herein referred to as the susceptible line (S-line). Combined family and within-family selection of this population focussed on fast growth, resistance to Taura Syndrome Virus, general pond survival and robustness for 16 generations under strict biosecurity protocols. The second population, herein referred to as the resistant line (R-line), derived from a Pacific Ocean population in 2008, was mass selected with high intensity (1 × 10^−4^) for survival in the presence of WSSV for 7 generations. The Atlantic and Pacific populations were maintained in isolation on their respective coasts of Colombia. All animals entering the study were considered as naïve (uninfected with the virus). Animals from the S-line were PCR tested every 3 months for all OIE listed pathogens and were found to be negative for all listed pathogens for more than 4 years. Before the challenge test a representative sample of the animals from the R-line was PCR tested for WSSV^43^ and found to be negative.

**Figure 6:**
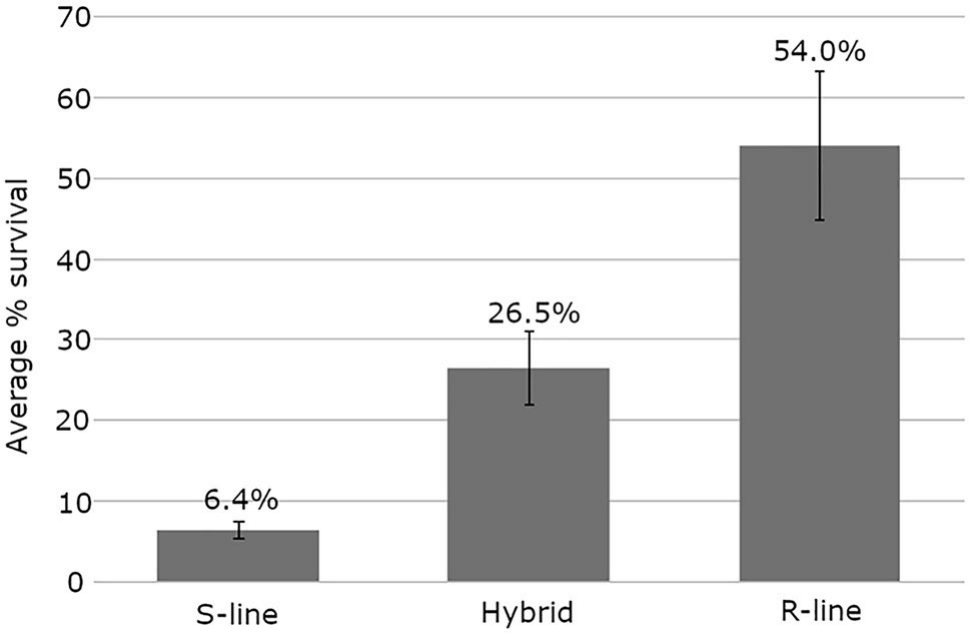
Comparison of average survival in the WSSV susceptible, resistant and hybrid starting populations.

The overall response of the Atlantic and Pacific starting populations to WSSV infection was known from previous trials. Past challenge test trials indicated that the S-line contains little phenotypic variation for resistance (with family survival rates in the range of 0–6%.). Deaths occur very rapidly after infection and no animals survive the challenge longer than 4 days. In contrast, variation in post-challenge survival in the R-line ranges from 20 to 54% 15–20 days after infection. The WSSV resistance of Hybrid F1 offspring, produced by crossing R- and S-lines, is intermediate between that of pure lines (Fig. 6).

The first challenge test used 751 pure R-line and 696 crossbred R- and S-line shrimp. These animals were used for training purposes to estimate genomic breeding values. Breeding candidates (not challenged with WSSV) consisted of offspring derived from 34 RxR-line and 24 RxS-line parental crosses (the pure R-line and RxS-line crosses being siblings of the challenged training animals). For the purposes of this experiment this starting population of animals, including test animals and breeding candidates, is referred to as the G_0_ population (Fig. 5).

### G_0_ WSSV challenge tests

Juvenile G_0_ shrimp of average weight 3 g were randomly divided into two tanks filled with 4 tons of artificial seawater at 30 ppt salinity at 26 °C temperature and infected with WSSV in a challenge test initiated on April 18th 2018, using minced infected tissue. The tissue used (with a viral load of 1 × 10^6^ copies/µg of DNA) was weighed and administered to the tanks at a ratio of 3% of the total shrimp biomass over two consecutive days. Mortalities were recorded and removed hourly until May 10th (22 days) to ensure that no animals were cannibalised and hence not registered as dead. Twelve days post infection, the shrimp were pooled into one single tank to avoid loss of infection pressure due to reduced shrimp density. Infection was confirmed by histopathology^44^ and nested PCR from pleopod samples^45^. Date and hour of mortality was recorded to provide data on two traits, dead or alive after 22 days (DOA) which is a binary trait (assessed 22 days post-infection) and days survival (DS). For the purpose of analysing DS it was assumed that survivors died the day after the trial ended.

### Collection of tissue samples and genotyping

Shrimp tissue samples, pleopod or muscle, were collected from all dead animals and survivors in April/May 2018 and November 2018, immersed in 95% ethanol at 4 °C and stored in 1.5 ml centrifuge tubes. SNP genotyping was performed by Neogen (USA) in Nov 2018. DNA was extracted using a BioKits DNA Extraction Kit (Neogen, USA, which utilises proprietary magnetic beads with a high affinity for DNA) and samples were genotyped using a custom 18 K Illumina SNP array designed by Neogen USA (described below).

### Single nucleotide polymorphisms

All shrimp were genotyped for 18,643 SNPs. A *L. vannamei* Illumina custom array was developed for SNP genotyping (Neogen USA). A total of 8,967 SNPs were previously identified and published^41^ while another 10,323 SNPs were identified using mRNAseq re-sequencing of pooled hepatopancreas, gill and pleoplod tissue from 20 naïve generation-3 R-line and 20 naïve generation-3 S-line individuals. Shrimp were dissected under sterile conditions and samples completely immersed in RNAlater (Qiagen Germany and stored at − 80 °C to preserve RNA until it could be purified and prepared for RNA-seq (previously described for *P. monodon*, Baranski et al.^46^). No RNA normalization was performed before the samples were pooled (creating one R-line and one S-line pool) and processed with 72 cycles of paired-end sequencing in two separate lanes on a Genome Analyser II (Illumina USA) according to manufacturer’s instructions. The CLC Assembly Cell “find variations” program was used to detect putative SNPs in the *L. vannamei* assembly as described for *P. monodon*^46^. Putative SNPs that were identified in homologous *L. vannamei* contigs were filtered to reject those with total sequence depth less than 51, minor allele frequency less than 0.1 and those lacking variation within the R-line or within the S-line (ie SNPs showing fixed allele differences between the two lines were not accepted). In cases where multiple SNPs per contig remained after filtering, the first SNP on the list was the only one accepted. Finally, the basic local alignment search tool (BLAST, National Centre for Biotechnology Information, USA) was used to check all flanking SNP sequences for matches against themselves and against the 8,967 SNPs previously published in order to filter out any repeated SNPs. Both the flanking sequence and the published SNPs were made into searchable BLAST databases using the *makeblastdb* command and matches were identified using the *blastn* command with e-value 10^−11^.

Post-genotyping quality control removed SNPs that were not in Hardy Weinberg equilibrium proportions, with minor allele frequency less than 0.05 or call rate less than 95%.

### Genomic breeding value and parameter estimation

A genomic relationship matrix (G) between all the animals in G_0_ was estimated as described by VanRaden^47^. Genetic evaluation was performed by running an animal model in a multivariate mixed model package DMU^48^. All animals challenged with the virus and all breeding candidates were included in the estimation. The traits DOA and DS were analysed separately, using the animal model:

$$y = Xb + Zu + e$$

where *y* is a vector of phenotypes (DOA or DS), *b* is a vector of estimated fixed effects of cross, fitted with two levels: purebred R or RxS cross, *u* is a vector of estimated breeding values (gEBV) for test-animals and candidates, *u* ~ *N*(0*,Gσ*_*a*_^2^), where *G* is the genomic relationship matrix and *σ*_*a*_^2^ is the additive genetic variance of *y*, and *e* is a vector of random residuals, assumed to be normally distributed with variance $\sigma_{e}^{2}$. *X* and *Z* are design matrices to link each observation to the correct fixed effect category and animal. Variance components for both traits were estimated separately in single trait models. Both traits were analysed as continuous traits, i.e. analysed on the observed scale only.

### Accuracy of genomic EBV prediction

From the G_0_ data we estimated the accuracy of genomic EBV prediction using cross validation. Each animal was allocated a number (1–10, creating 10 randomly selected non-overlapping validation subsets), and the analysis was repeated 10 times, each time masking the phenotypes for all traits for one of the validation subsets. Accuracy (Acc) was estimated for each trait as $Acc = \frac{{R_{gEBV,y} }}{h}$, where $R_{gEBV,y}$ is the correlation between the phenotype and gEBV, using, for each animal, gEBVs from the run in which its phenotype was masked, and *h* was the square root of the heritability, estimated from the full dataset..

### Power analysis

The response to selection can be predicted based on the estimated genetic parameters, given a certain selection intensity. A power analysis was applied to design a selection experiment suitable to document genetic gain and validate the genetic parameters within the limitations of available infrastructure. Predictions of the power for evaluating genomic selection were performed for the DOA trait, because this trait was thought to have the highest economic relevance. The maximum total number of full-sibling families was limited to 60. The number of offspring tested from each family was set at 25 to make sure that the total number of test animals did not exceed the capacity of the test facility. Different options for production of selection groups were compared. High gEBV groups were produced by crossing G_0_ individuals with the highest gEBV values. Similarly, low gEBV groups were made from crosses between individuals with the lowest gEBVs. The “random” family group was created by crossing remaining individuals at random. Expected differences between comparisons of the random- and high-gEBV groups, and between the low- and high-gEBV groups (as the trait was selected in both directions) were based on estimated genetic parameters and the selection intensity used. The power analysis assumed that observations were independent (ie. family structure was ignored) and power was estimated using the formula $t_{\beta } = t_{0.05} - \Delta G/se\left( {\Delta G} \right)$, where *t*_0.05_was the critical value needed to reject the null hypothesis of no difference between the selection groups (assuming a standard normal distribution) and Δ*G* was the expected difference between 2 selection groups. Δ*G* was estimated using a univariate breeder’s equation $\Delta G = h^{2} *S$, where h^2^ is the heritability estimated from the G_0_-test animals and S is the phenotypic selection differential applied when producing the families. *se*(Δ*G*) was the standard error of the contrast between two selection groups, estimated as $\left( {\frac{1}{n1} + \frac{1}{n2}} \right)\sigma^{2}$, where n1 and n2 are the number of animals in each group and *σ*^2^ is the phenotypic variance estimated from the challenge test of G_0_. The power was estimated as the area under the cumulative standard normal distribution from − ∞ to $1 - t_{\beta }$.

### Selection experiment

Breeders were selected from the G_0_ breeding candidate pool based on DOA gEBVs estimated from WSSV challenge tests run in 2018. Males and females, respectively, were ranked on gEBV, and both tails of the ranked list were selected. The top 60 males and 60 females were selected as possible high resistant breeding candidates and the bottom 20 males and 20 females were selected as possible low resistant breeding candidates. Animals not selected for either of these lists, and untested animals from the G_0_, were available as possible “random selection” parents. Sixty families were produced, 32 from matings between high resistant animals, 8 from matings between low resistant males and females and 20 from matings between randomly selected animals (Table 4, close to the 30 high, 20 random and 10 low power test with a value of 0.96 on a 0–1 scale). The average genomic breeding value of the parents were 0.25 for the resistant, − 0.005 random and − 0.28 for the susceptible group. Mating between likely full-or half-siblings (pairs having close genomic relationship, > 0.1) was avoided. The offspring are for the purposes of this experiment referred to as the G_1_ population.

**Table 4:** Choice of mate pairs used to produce the low, random and high gEBV groups showing average and range in genomic estimated breeding values (gEBV) and genomic relationship values (gRelat) for selected G_0_ individuals.

Group	Female gEBV		Male gEBV		gRelat	
	*Average*	*Range*	*Average*	*Range*	*Average*	*Range*
Low	0.30	0.28:0.33	0.30	0.28:0.33	0.00	− 0.06:0.07
Random	− 0.04	− 0.14 :0.05	0.00	− 0.18:0.18	− 0.03	− 0.12:0.05
High	− 0.28	− 0.48: − 0.21	− 0.30	− 0.58: − 0.20	0.01	− 0.12:0.11

Two females in the low gEBV group and two males in the high gEBV group were mated twice to produce half-siblings.

Mate lists were generated, and animals reproduced by artificial insemination in March 2019 at Ceniacua’s hatchery in Tumaco, Colombia. The artificial insemination of select broodstock, involving extraction of spermatophores from male candidates and transfer of the spermatic mass to artificially inseminate female candidates, is described by^12^. Females were placed in individual hatching tanks. After hatching a random sample of about 5,000 nauplii from each family were stocked in separate 50L-larvae-culture tanks. Larvae were fed a mixed diet of *Chaetoceros* sp. *Artemia* sp. and micro pellets. At the post-larvae 10 stage (PL10) a random sample of 200 post-larvae per family was transferred to separate tanks at a 100/m^2^ density for on-growing until they reached a body size suitable for elastomer-tagging (approximately 1 g). Tagged animals were then sent to the challenge test facilities in Bogotá Colombia in May 2019.

The 1885 G_1_-animals were evaluated in two tanks. Family size ranged from 12 to 53 records (average 31). The challenge test was continued for 23 days post-infection. The data from the challenge test was used to estimate genetic gain and to re-estimate genetic parameters, to validate the results from G_0_. The G_1_-data was analysed with a sire-dam-model in Asreml^49^ where,

$$DOA = X\beta + Zu + e$$

where DOA was a vector of the binary dead or alive phenotype. β was the fixed effects of tank and selection group (high, random or low resistance) and X was the design matrix to link observations to the fixed effect categories. *u* was a matrix of random effects of sire and dam, $u\sim G*\frac{1}{2}\sigma_{a}^{2}$ where $\sigma_{a}^{2}$ was the additive genetic variance, G was the genomic relationship matrix between the parents (the G_1_ test shrimp were not genotyped), and Z was the design matrix assigning DOA records to the correct sire and dam. Selection group was fitted as a fixed regression rather than categorical because despite the different number of parents selected for mating to produce the high- and low-gEBV selection groups, selection intensity was of a similar strength in both directions. Expected genetic gain from the realized selection intensity and heritability in G_0_ was estimated by average breeding values of the parents multiplied by estimated heritability. Realized and predicted genetic gain were compared.

## Supplementary information

Supplementary information.

## Data Availability

All raw and processed sequence data generated in this study have been deposited in the NCBI Short Read Archive (https://www.ncbi.nlm.nih.gov/sra) under BioProject accession PRJNA625155.
