# Supplementary material for: Genomic selection for white spot syndrome virus resistance in whiteleg shrimp boosts survival under an experimental challenge test
Source: Sci Rep. 2020 Nov 25;10:20571. doi: 10.1038/s41598-020-77580-3 (PMC7688931; doi:10.1038/s41598-020-77580-3)
Supplement: Supplementary file 1 — Supplementary information. [file 41598_2020_77580_MOESM1_ESM.docx]

Supplementary Information for

**Genomic selection for white spot syndrome virus resistance in whiteleg shrimp boosts survival under an experimental challenge test**

Lillehammer M.^a*^, Bangera R.^b^, Salazar M.^c^, Vela S. ^b^, Erazo E. C. ^c^, Suarez A. ^c^, Cock J. ^c^, Rye M. ^b^ and Robinson N. ^ad^

Marie Lillehammer

Email: [marie.lillehammer@nofima.no](mailto:marie.lillehammer@nofima.no)

**This PDF file includes:**

Tables S1

Table S1. *L. vannamei* SNPs detected by mRNAseq. Position in contigs of our assembly of the *L. vannamei* genome are indicated along with alternative alleles (1 and 2), sequence depth and frequencies of each allele in resistant and susceptible line populations and sequence (trailing and leading sequence including SNP annotation).

| **snp_id** | **contig_ID** | **Position_within_contig** | **allele1** | **allele2** | **seq_depth_R-line** | **freq_allele1_R-line** | **freq_allele2_R-line** | **seq_depth_S-line** | **freq_allele1_S-line** | **freq_allele2_S-line** | **SNP_annotation** |
| --- | --- | --- | --- | --- | --- | --- | --- | --- | --- | --- | --- |
| comp98937_c0_seq1:279-280 | comp98937 | 279 | G | A | 19 | 0.947368 | 0.0526316 | 72 | 0.0138889 | 0.986111 | CTGCATTCCCTTCGTGCAAATTCATTTACTGCTGAAAACGCACTTCGGTGGCCATAACTCTCTCGGCGCTTCCTTCGGTGTCTGTGTCATCCTATAGATAT[G/A]TTTCCCCTTTTTGCCAACGCCAATTTCTCGAAATAGGGTTTTGTGATATTTATTATTTCATACTGTATATGAATTTGCTGAATTCATTCAAATCCATGTGA |
| comp105413_c1_seq3:264-265 | comp105413 | 264 | T | C | 108 | 0.037037 | 0.962963 | 83 | 0.939759 | 0.060241 | TCGTTATGTAACCCTCCACAAAAAAACAAAACAAAACAATCTATGACGTCATAATACTGCGCAGAATAAAAAGAAGAAAAAAAAAATAGAGTTACATACCC[T/C]AAGCTTGTATCATCATATCATCTATCGTTTATCTTGGCTTATATATGTGTTTTTTTCCTTTTAAAATAGGCCTATAGAATTAGGTCTTGCTTCGGTGGACC |
| comp76879_c0_seq2:267-268 | comp76879 | 267 | A | C | 230 | 0.904348 | 0.0956522 | 1186 | 0.00168634 | 0.998314 | GCCGGTGCTGGAGCAGGCGCTGGCTTCGGTGCGGGCGTCGGGGGCGGCGTGGCTCAGCCCTCAGGTCTGTACTCCGCACCTTAGAAGCTCATGTACTTGGG[A/C]GCAAAAATGAGTACGCGCTGAACAAGGCGATGTTCAGATCTTTTATTTATTCGCTACTTATATAAACAATATTTATTTCAAAAATGTTCATTTCTCATTAG |
| comp102458_c2_seq20:364-365 | comp102458 | 364 | T | G | 449 | 0.919822 | 0.0801782 | 158 | 0.0189873 | 0.981013 | ACAAAATATTCTTTAATGGCAAATTTAGAATATCAAGAAAGAAAAAAAAACACAAAACATTGGTGATAAAACAATAGACCATTAGTAAAACCTTACAAGAT[T/G]AACCTCACCCCCTTTCAAGAAAACAAAACCCCCAACATAGAAACAACAATTTATCAATCATCTATTATCTCCATTGTCTTGAAATCACCCAGGGACCCTTA |
| comp104861_c0_seq10:993-994 | comp104861 | 993 | C | T | 183 | 0.912568 | 0.0874317 | 78 | 0.0128205 | 0.987179 | TTTTCCTTTGTAACCATTAACTTTTTCTTACACGAAGTATCATTTTAAAAGCTAAGTTGAGAACTAAGTAGGGTAGGGAATGAGTTTTTCATTGGATCTTG[C/T]CGAATCATTTACTTAAATACTAAGACAAATAATGCTGTTGGTTACATATGGTATTATTTAGTATCACGAAATGTATAGTCTTAACAAAAACAAAATGATTC |
| comp98070_c2_seq3:114-115 | comp98070 | 114 | T | C | 418 | 0.901914 | 0.0980861 | 214 | 0.0046729 | 0.995327 | CTTTTTTTTTGGTAACTGCATCTTTTATATCTAGACATGCATCACTTCAGACGCAACATACACATAATAAATATGAGTGTAAAATCCTTCGATTCCTTTAT[T/C]CTCCTCTTCGGTTCGTCTTCGTTAATCTTACTGTGGCTGTCCGTACAGATTCGATGGCGCTTCAGCTGTTTTCAGTGCGGCTTCGGCTTCAGCGGCGATGC |
| comp98992_c0_seq1:140-141 | comp98992 | 140 | T | C | 68 | 0.911765 | 0.0882353 | 64 | 0.015625 | 0.984375 | GTGTGTCGTCCGGCGCGGCACTCCCAGCCCTGCGCTAAGTGTGTTTACATTGCCTTCGCGCGTCGCCATCATCGCCTGCTGCCCACTTTACGATTCCATTG[T/C]GAGAGCTTTTTAACATACGAGAACATCCACTTCTTCACCTGCAAGAACTAGGAGTCTACGGTTCATACCAAGACAATGAAAAGAATATTATAGTGAAGCAG |
| comp93961_c0_seq2:995-996 | comp93961 | 995 | A | C | 66 | 0.909091 | 0.0909091 | 58 | 0.0172414 | 0.982759 | CTGGAGGAGCACAACGCCAAGCTCGAAGGGAGGGTCCTCTACGAGGGAAAGGAGTTCGATCGCCAGCTCGTGCAGGTCCTGGTCGACATGTTCAGCGCCGG[A/C]GAGGAGACCGTGAAGACGTGCCTGCTGTGGTCGCTGGTGTATCTGCTGCACAACCCCGAAGTGATGCGGAAGGTCCAGGACGAGCTGGACGCCGTCGTGGG |
| comp107404_c0_seq2:139-140 | comp107404 | 139 | T | G | 389 | 0.894602 | 0.105398 | 601 | 0.00998336 | 0.990017 | GACGCGAGAAGGAAGAAAGGAGAGAAAAAAGATGAGGTTGGAGGTCGTGTCGCTGCTGCTGGTCGGCCTCGCGGCGGGGTTTGGCTCGGCCGAAGGGGGCG[T/G]CGAGGGCGGCGTCGAGTGTCCTTTTCCAGAGGGACAGAGCGTGGCCAGCGAAGGAGAGTGTGCCAAATACAGTGCCTGCGAGTGGACGGAAGGTCAGTGCC |
| comp105898_c0_seq4:1036-1037 | comp105898 | 1036 | A | C | 34 | 0.941176 | 0.0588235 | 53 | 0.0566038 | 0.943396 | CCACATACCTTCAGTACAGCTACCATCCAAGGGAACTCATGCTCGATGGTGGCGGCTCCTCCAACAATCCTCGTTTTCTTGTTGCTCACGCCGCACTTCCC[A/C]GCCCAACCGTCGTCTCCGGGCCCCGTGCAGATGACAGTGTGTTCAGCCGCTGTGTTGTTGGTTGTGACAGTTCCAACGGCACCTGTACCATCTGCATTTAG |
| comp99568_c1_seq1:301-302 | comp99568 | 301 | G | A | 60 | 0.933333 | 0.0666667 | 41 | 0.0487805 | 0.951219 | GGCAAGTAGACATCGACAATCACGCGGAGGCAGACAATTTGCAGACATTTGTGTCAACCAAGCGGAAAGCAGTAATCCAAGCACCACCTACAGGCGGCCAC[G/A]GGCTCTCGACTCCTCACTCGGGTATGAAAAGACCATGGAGCGCCCTCGCCACGTCAGAGGAGGTCACGAACTCGACTCTACCCATTTCCTTCATGTCTGCG |
| comp105532_c0_seq1:1335-1336 | comp105532 | 1335 | C | T | 200 | 0.895 | 0.105 | 77 | 0.012987 | 0.987013 | TACAGAGAGTCAACAAGTAAGCAAAACACCGTGTCTGCCACGCCTTGGGCCGAACACAGAGCATGATATAGGCCTAAGTACCTGAATGAGTTTACCCTTGT[C/T]TATTCTCCTTCGGTGGAGCAGTAGGCCCTGTTGGTGTGTCCTGCGTCGTCCCCTGAGGGCTTGCTCTGCAGGACGGCCTTCTCGCTGCCTCGCGACGCCCC |
| comp105361_c2_seq12:364-365 | comp105361 | 364 | A | G | 65 | 0.923077 | 0.0769231 | 24 | 0.0416667 | 0.958333 | GGTCACTTCTCCGAACATCCCAGCAAAAACAATGGGTTTCTGTGGGGCAAGAACACGCACACCAACAAATCTGGTTATTACCCCGCTTACAAGACCGTCGA[A/G]GTTTTGAAAAAAGCAGATGTTCTATCCTTTGAACACATTGACAGAAAAAAATAAAATCTTATGTGTTGTTACATTTTTTGATAGATTTTTTTTCTACTTGA |
| comp107549_c0_seq3:2037-2038 | comp107549 | 2037 | G | T | 119 | 0.890756 | 0.109244 | 88 | 0.0113636 | 0.988636 | CTCTGATTTGCTCTCTATTTATCCATAGTTTATAATCAATCATTTATCCTAAACCTTTAATGTTCCTTTCTTTTATTCTGCAGTATTTTACCAGAACAAAT[G/T]ATTTCAGTATTTGTGCAATACACAATGAGTGAAAAGACTCTATATCTTTTTTTTAGCATATGATACCATTATTCGTAGACTAATACTAATAATAACGTTAA |
| comp102896_c0_seq12:232-233 | comp102896 | 232 | A | G | 335 | 0.0507463 | 0.949254 | 225 | 0.928889 | 0.0711111 | TTTCCCTCCTTCCTCAGTGTCACGTGACCAAAGCGCACGTCACTTGTTTTCGATTATCTCAATCTTCGAAATTCAAGACCTCTAAACATACAAATCAAACC[A/G]AAATCAGTAAGGAACTAACATACACGCTCTAAGGTTGTTGTTTTTTTTTTTATGTCGCAACAATTAACAATTAATTCTTTTTTTTATCCTTTTACTTTAGT |
| comp102524_c2_seq1:433-434 | comp102524 | 433 | A | G | 28 | 0.892857 | 0.107143 | 56 | 0.0178571 | 0.982143 | TTATTCATTTATCTATTCATTTGTCCCCGTCTGTAAATCTATTCGTTTATTTATTATTCTTAAGTTTATCCGATAGATTGTGGACAATTCCGTTTCACTTC[A/G]CTCTTTCCTCACGTCAACATCTACTCTTTAGATTCCTTACGCTGATATATTAAGAGTCAGTTTTCAACATGTCTGCGGTTTCACGAAGTTTGGTTTCTAAG |
| comp104010_c0_seq26:604-605 | comp104010 | 604 | T | A | 15 | 0.933333 | 0.0666667 | 83 | 0.060241 | 0.939759 | GGCTTTTCACAGTTGGCCTCCTCGAAGATTTCTGGGATTTTGTTGACAATCGCAAGGGTGCAAGTGTAGCCAACCTTTGCCCACTGAGCATGATGGTCCGG[T/A]GCGTTGAATGTGTAGAGATACACCTCCTTGGGGCATTCGACTTTTTTCTTGTTGGGAATCTGCTTATTGAGTTTCTTATTCTGGTTTTTGGCTCTTTTAGC |
| comp103117_c0_seq11:431-432 | comp103117 | 431 | C | T | 147 | 0.911565 | 0.0884354 | 175 | 0.04 | 0.96 | TAACCAACATGATCACACACCCCAACAAAAGAATTGAACTTTGTTGCTTCATAGGGATTGCCTGTCTTATCATATCAGAGTAATGGCAGGGATGTTTTACA[C/T]ACAGTCCTCCTAATACTCACCGAAAACGGTACATAAATTCAATTACAGTGTCAGATGCTCTGGAAATATGTAAAATTGAAAAGTACAAACATACAAGGATA |
| comp106467_c0_seq14:843-844 | comp106467 | 843 | A | G | 177 | 0.887006 | 0.112994 | 122 | 0.0163934 | 0.983607 | GATGGAAGCCCTTGTCCTGAAAATAGACGGGGGTGTCGACCAGGACTTGGAAAAGGTCAGGGTGTCTCTCGCGAAGGACGTCGGCCACGTTGAGACCGTCG[A/G]CGAGGTCATTGTCACCTCCTTTGCCCTCGTGTTGGCGCAGGCAGTGAAGCCAGGTCGTCCCGGGTGGATATTCGTAGTACGGCAGATCCGTGTGCAGACCG |
| comp107078_c2_seq21:2357-2358 | comp107078 | 2357 | T | C | 725 | 0.892414 | 0.107586 | 203 | 0.0246305 | 0.975369 | AGAAAATTATATCAGTTTGAGATAATAATAACAACTATAATGTTAATAATAACTAATAAAGGCAAATTATAATAATTTTAATTCGTTATAATTGGACAAAC[T/C]TATGGAAACAACATTTGGCAGCATTAGAGAAAACAGCCACTTTCTATGTGTACTGATGTGTGCGCGACAAAATTTCCAAGTTTATAAAATATAAATATTTA |
| comp97981_c3_seq8:134-135 | comp97981 | 134 | T | G | 834 | 0.934053 | 0.0659472 | 383 | 0.0678851 | 0.932115 | CATTCTGGGAGGGAGCGACAACTCTCCTCAGACCAACGTGTGTTTCAGAGCCAAGGCAACGGGGTGCAAAGAGGGAGACCTGACCTGACCTGACCTGACCT[T/G]ACCTGACCTGACCTGACCTGGCCTGATAAACTGTAAGAAGTGACCCGGACCAAAACGCTGGGAGACAGCGACTCGGTCTGACCCTCAAAGAGTGACGTTTA |
| comp99272_c0_seq3:170-171 | comp99272 | 170 | A | G | 361 | 0.886427 | 0.113573 | 49 | 0.0204082 | 0.979592 | CATGGCTGAACTACCATGCAGCCGACCGTCTTGCAAAAATAGATAAATAAATAAATAACTTTTTTAAAAAATCATGTAACCTAGGAAACTAATGTAAGGAA[A/G]GTCTTTTGAGATTAAAATGACAACGGAGGTGGGTACAGCCAAACTGTGACAAACTAGTAATAATCCCATATAGCGTTTACATACCACAGAACATATAATGG |
| comp107421_c0_seq3:1252-1253 | comp107421 | 1252 | C | T | 18 | 0.944444 | 0.0555556 | 62 | 0.0806452 | 0.919355 | AAGACGTGTGGATGGTTATCTGTGTAGAACGATATGACTGATATGAAACAATATGGGAAATATGAAACAAAAGTCAATGTAGAGTTTTATGATATTCTGCT[C/T]TACGGAAAGAACAGAGTAAGACGTTAATGTAAAATGAAAGGTTGAGTGTTAAAAGAATAATCGACATGATATGAAGTGATATGAAGCTTCTCAGATATGAA |
| comp107556_c1_seq7:1592-1593 | comp107556 | 1592 | A | G | 314 | 0.878981 | 0.121019 | 65 | 0.0153846 | 0.984615 | TCCCTCTTCCTTCCTTCATCACTGGCTCGAAATCAGCGTGGTCCCGACCCAGTTGCATGGCCCACCTTAATTTTTCTAAGAAACTCAAGGCACAGTCCTGT[A/G]GAAATAGCAGTCTTTTCCTGCCGTTCACACCGGCAACATTCTGGGCTGCTGTTTCGTTTCCGTCGTTCTTCTTCCTCTTCTCCCGCTCGCATCCATTCTCG |
| comp103685_c2_seq1:2467-2468 | comp103685 | 2467 | G | A | 717 | 0.0585774 | 0.941423 | 1112 | 0.919964 | 0.080036 | ATGTGTGTGGTGGGTTGGTTTTGGCTGGGGGGGGGGAGAAAAATGACTGACTACACTCACACCCTTTCCAGTCAGCCCATGTCTTAACGTTTTCCTTTCTC[G/A]TTATATTTATGATTACTTTTTCAAAAAATACCATGCTTCCTTGTTTTTCTTTCTCTCTCTCTCGCTCGCATTCTCTGTTTCTCTTCACTTCCTTTTCCTTC |
| comp106119_c1_seq35:295-296 | comp106119 | 295 | T | G | 56 | 0.0535714 | 0.946429 | 35 | 0.914286 | 0.0857143 | GGCCAACTGGATCATTCCGGATCCCGCCGGCTCGAGCCAGTTCCGACCGGTGCGCCAAGCCTTCCACTTTCCCTCCCTTTGGCCTTTTGCATCTCTTTGGG[T/G]GTTCTTGTCACTCTTCAGGGGGTTCTTGTCACGCTTCAGGGGGGAAGAACGGTCAGGGTCTGAAAGGTCTTCCAGAACAGCAGTTGAGGCGCACTTAGAGA |
| comp88673_c0_seq1:283-284 | comp88673 | 283 | A | G | 38 | 0.0526316 | 0.947368 | 315 | 0.911111 | 0.0888889 | CGAGAAATAATCTTACAAAAGGTAATCAATGTACATAGGAATGACCATGGGATTATTATGTGTCTCAAGGAGGACGACCGAGAGAACAGGAAGGAGAAAGA[A/G]GGAAGGAGGAGGAGGAAGATGAAAGGGAGGAGTAGAAGAGATTACGAAAGAGGAGGGAGAGAAGCGACAGAAAAGGGAGGGAGGGAGGAAAGGTGGGGGAG |
| comp97795_c1_seq13:1128-1129 | comp97795 | 1128 | G | T | 487 | 0.00821355 | 0.991786 | 1013 | 0.865745 | 0.134255 | ATATTTGCTCTCAATCTAAACGTTAGCTTTCTGGAGGCCCTCACCTTGTACCATTTAGGGACACGGAAGGTAAGGATCATGTCTCCTCCGGCACCATGGCC[G/T]CGCTGGTTGTCGTGGTTGTCAATGAAGACGAGTGAGTTGGCCCTGTGCATCATGCCCCAGCCTTCGCCGAAGTTGTTGAGGTACTTCAGCTGGTTGTTGCC |
| comp94549_c0_seq1:272-273 | comp94549 | 272 | C | T | 1035 | 0.864734 | 0.135266 | 692 | 0.00722543 | 0.992775 | TTCCTATTTAAAACTGGACCGAAGAATGAAGAAAAAAAGGATCAAGGAAGAAAAAGAGAAAGGACGAGTCGAAGAGTGATAAAAAAATTACAAAAAATATT[C/T]TAACTATTAAATAATTTTGTTGACAGATTAGAAATATGACAACCAAGATTTTTCTTCATGAAGGGCCAGGCAGTTCTCTCATCATTGTACAACTATGATTT |
| comp107804_c2_seq21:797-798 | comp107804 | 797 | G | A | 198 | 0.0858586 | 0.914141 | 194 | 0.943299 | 0.056701 | ACAAAGCTATTATTTACAAAAGAAATTAGGAAACTGTGTGACAAGTGATAGCAAGTGACAGTATTTTTATTACTATTTACACAATATAAATGTTTTTGATA[G/A]CATATTTTTTTATGGGGAATTATTTGTCTCTGTGCTTTGTGGATGATATATTTTTGGTATTGTTGATGAATATTTTCATAATTTTGTATAAAAAGATTTGA |
| comp107941_c0_seq10:1301-1302 | comp107941 | 1301 | T | C | 803 | 0.860523 | 0.139477 | 313 | 0.00319489 | 0.996805 | CCCATGAGCGGCGACCGGCAAATGGCGACCACGGACCAGCGGCCGGTTGACTGACGCCGACCGTTGCTGGCGAGACCTGGCACTTGCCGGCGTACGTGCGA[T/C]GGCCGCCCACTTAGACCTGGGTGTCGTCGTGCTTGAGTCCAGGCGAAGTGTCCCCCGCCCGCAGGTCCACGGTGCGGCCCGGACGCGGCTCCGGCGAGCTG |
| comp102424_c0_seq1:138-139 | comp102424 | 138 | C | A | 65 | 0.861538 | 0.138462 | 300 | 0.00666667 | 0.993333 | TTACATCAAAATCTTTTATAATTTTATATAATATATATAGAAATCTGGCCCTTCGTTAACAAATTTATATCATTAAATTACAAGTGGCAATTAAAACAACA[C/A]CAAAAACAAACAGCTACTCATAAAAATAGAAAAAAAAACTACTGTAGCTATGTATAATAATATTCTCTATTTCATTATTTTTACTTTTACGTTGACATTCA |
| comp100231_c0_seq2:420-421 | comp100231 | 420 | A | G | 78 | 0.923077 | 0.0769231 | 29 | 0.0689655 | 0.931035 | GTGGATAGAGTAAGGAAGGATAAGATATGGATGAAAAAGAAAGCTTGATATTTTGGATTGAGTTAGGGAGAGAAGGGTAAAAACTGTGGATGACAGACGGG[A/G]TAAAATTAAGAAAGAGAAAATGAAAAGGGTGAAAGGTAGACTAATGTAAAAGAGAGAAAATGACAGTTTAAATGAAAGGTATGGAAGAGACACTTGGTAGA |
| comp107906_c0_seq1:4606-4607 | comp107906 | 4606 | G | A | 228 | 0.934211 | 0.0657895 | 12 | 0.0833333 | 0.916667 | AGTGCAACGGCTGAAGTGCAGGTTCCCGAAGTGTCAGCTCCAACGGTTGCCATTTTCGAAACGGACGCGGGACCTGAGCCCGTTTTTGAAGTAGTAATGAT[G/A]TCAGAAGAACCAGCAGGCGCGGGACCCAGGACCAAGGTAACCATCATCACAGACGACGCCCCATCAGACCCCTTAGATTACGATGACGCAACAGTCCTCGT |
| comp104284_c0_seq7:1355-1356 | comp104284 | 1355 | C | T | 154 | 0.896104 | 0.103896 | 40 | 0.05 | 0.95 | TCTGATACACGCATTGAATTTATTTTTATTTTCCTTTTTCCCCTTTATAACAATACTTAATCGAGAGGAGACACTTCTATCATGAAGTCTTAACAGAATAC[C/T]TTATTTACTTCACCAATACACGTATTTAGTTACTATTCTATGGGAAGAGCAATGAAATGCTGACAAAAACATTATTTGAAAAGATTCCGGTGCACGATAGT |
| comp105092_c1_seq5:419-420 | comp105092 | 419 | C | T | 72 | 0.902778 | 0.0972222 | 50 | 0.06 | 0.94 | TATTCATGTACATTTTGCCTCACAATGCATAGATACAATACATGTTTTCTTTTCTGATAACAATACTCATATGTTGTGAAAGAAAAATTATTCAATAGATG[C/T]ATTTTTCTGTTTTATGTGGATATTGTCAATAACACAAGTGGAATGATCTCCTATGGAATTTTGTATGTTTGTGTGTACGTGTATGTATATGTTTGTGTGCA |
| comp99054_c0_seq3:686-687 | comp99054 | 686 | T | C | 90 | 0.888889 | 0.111111 | 43 | 0.0465116 | 0.953488 | TAGTATATAGTAACGCTAGGTGTATTTTTTTGCTGTTTGACATCCGTGTTTAGCAAAATTCTTTTATACATATCATTTCAAATGTCACTTCTTGCCCTCCA[T/C]AGATGTGAAATGTACAAGTATATCATTTCTATTAGGAGCTGTATGAACCATTCGTGATGGACACTTCTTTCCCGTTTCTAAATTTTCTTTATGACGAAAAT |
| comp96500_c0_seq1:785-786 | comp96500 | 785 | A | G | 432 | 0.93287 | 0.0671296 | 44 | 0.0909091 | 0.909091 | CCGACGCAGGAATTCTACGCAGTGACTGCAGACGCGCCGTCGCCCGTCAAGGTGCCTTACCTTAAGCCCCAACCCAAGCTGTCCCCGGGAAAGCCAGACCC[A/G]AAGGACGCGGTGCAGCTCCTGCCGGACAAGACGGTCTCTGACACGGCGGAGATGCCCGCGTTCACGATCGGGAAGAAAATCGTCGTCCGCCCCAAGAACGA |
| comp107927_c0_seq5:459-460 | comp107927 | 459 | A | G | 66 | 0.924242 | 0.0757576 | 12 | 0.0833333 | 0.916667 | GCCTATACCCCCTCCCCCTTGTACTAAGATAAGTTAGGTTCCTGAGAGCCTCGAATCCCGCAACAATGGATTAGCTAATCGGGCGTCTTAGCAGCCACGAA[A/G]ATAAAGAGAAAAAGAAACAGAGGACACAGATTAACGTTGTGCGAAACTGCCAAATTCAGAAATGTAGAAAAAGCAAAAAACAAAAATTCGTCGGCGTTTCG |
| comp94557_c2_seq1:341-342 | comp94557 | 341 | G | T | 1507 | 0.931652 | 0.0683477 | 2800 | 0.0925 | 0.9075 | AAAGGAGGTGAGAGAGAGAAAGAGAGATAGATAGATAGATGAGAGAGAGATTGAGAGAGAGAAAGAGAGAGAGAGTGAGAAGAACAAGAAAATAATAATAA[G/T]AAAAAAAAACAAAGACAGAAATAGAAGAGAAATCACAAGAACGGGGAAGAGAAGACCCAGAAAGAGTGAAAAGAGAATAAAATAAAAAAGCGAACTCAGAA |
| comp105625_c1_seq1:1082-1083 | comp105625 | 1082 | T | A | 73 | 0.863014 | 0.136986 | 39 | 0.025641 | 0.974359 | TACTCATTAGTTAAGGAGAGTGTACAAAATATAAGTAGATTTGCTTATGACGAACACCTATCCACCTATTTTCTACATTGAATTATTGCCAGTACCATAGC[T/A]GGTCTATAATGATTCCCGGCTAAAGGACGTGTATTCATCAGATATAGCAGCCCATAGTTTCTGGCTGGGAGGTTCCGATTCCACTGAAGGGCATTGGGTCT |
| comp107675_c0_seq3:2538-2539 | comp107675 | 2538 | A | G | 90 | 0.9 | 0.1 | 47 | 0.0638298 | 0.93617 | AGGTACTGCTTCTTTGCCCTCATGTTTATCGGAGGTTTTTAGTGTCGCTTCTTCGTTGCTGAGAGCTGTTCGATCGTCGTTGTCTGTGTCTTGCATTCTTG[A/G]TTCGCTGAGAATTGTATGACTTGTATGGCCTTTTTTGCACAATTCTTTTCCAGTTCAGTCAGTTTCTTAGGTGTAAAGATAATTTCAGTTTCGAGTGGCAT |
| comp104027_c0_seq1:948-949 | comp104027 | 948 | A | C | 83 | 0.060241 | 0.939759 | 38 | 0.894737 | 0.105263 | AGGTTTGCATCCTGTAAAGATTGCAAATGAATTGGAAACAACAATTTCTTGTGTACATTTTGTCACATATGAAAAGTTATTGTGTGAAATATGTTCCATAC[A/C]CTATCAAGACTGTACAGGCAGTTCGTTCACGTGTTCAATGAGTAATTTTACTTATGATATCATAACAGGATAGTAGCATAATATTGGATTGACAGGAAGGA |
| comp106617_c1_seq2:355-356 | comp106617 | 355 | T | C | 72 | 0.861111 | 0.138889 | 36 | 0.0277778 | 0.972222 | TATTCAATTTTAAAACGTGTTTGTCGGTGTTTTTGTTTTCTTTTTGGAATATGGACATTGTGTAAAACTTGGATGCATATATATATAAAAACAGGATTTTA[T/C]TTTATCTAATTTTTACGTATTTCTTGTATTTTTTCTTTTTTCTCTAGTTAATTGACCGGCATTTTTCGTTGCTGGCTTGTGATTGGCTGATAAGAGAGCGT |
| comp107079_c0_seq1:1085-1086 | comp107079 | 1085 | C | G | 51 | 0.862745 | 0.137255 | 34 | 0.0294118 | 0.970588 | AAGGTGAGAGGAATCAACAGCCGGGTCCTGAGCATCCTCAAGGTCTATTTGACTCTTAACGATCTAACGCCTTCCTCGCCACCCTTGAGCGATGTCACTTC[C/G]GTAGCGAGCTATAGCAAGGTCTACCAAAGGAACCTCGATGGGAAGTTGTCAGGTCATCGCAGAACCCATTCTGCACCACTAGACAGTGAGGACACAACAGG |
| comp107525_c2_seq10:1330-1331 | comp107525 | 1330 | T | G | 62 | 0.887097 | 0.112903 | 91 | 0.0549451 | 0.945055 | TGGAGCCACGTCGCTGCCGAACCCTGCTTGATCCTTCGACTGAGGAACCGCCTGCAAGTCCTCGTAAGGTCCCTCTAACAACGTGGACTCCCTTTCCATCA[T/G]CTCGTGGTTCAACTGCAACATTGTCTTTCGGCCTGGACTGGACCGACCTCTTCTGGCGTGGTTCCAAGACTCAGACTTGTCCCCTTCCACGTATAAAGAAT |
| comp106391_c0_seq1:1378-1379 | comp106391 | 1378 | C | G | 164 | 0.920732 | 0.0792683 | 56 | 0.0892857 | 0.910714 | TCGAGGGGCGGCGCGGGCGTCTGGCCCCTTCGTCGGGTCGCTCCAGAGCCGGAGCCGCCGGTCGCTACCCGGACGCGGTCCTTGAGGTCGGGCACGTCGTA[C/G]CCGGCGGCGATGGCGGCGGCGATGATGTCGGCGCTGACGCGGCCGCCCGACGACAGCGTCTGGAAGTACTCGTCGAAGTACTGCGAGTAGTAGTCGGCGCA |
| comp102801_c2_seq1:399-400 | comp102801 | 399 | C | A | 76 | 0.894737 | 0.105263 | 125 | 0.064 | 0.936 | TATTAAAGCAAAAAAACATTTAAGAAGGAAGGGGGTAAACTCAAAGCCGAGGAACTGGAGGAAAACAAAAATACATAGAATAAAACAGAAATAACATCTCC[C/A]TTTATTCACAGGAAGCTACAGGAATATCTCGTGGCCCTTCCGGATTATTCTCGGTCACAGGAGCTAACCAGAAAATACATAAATTACTTAACTCGTCCGCA |
| comp107901_c1_seq2:2137-2138 | comp107901 | 2137 | C | A | 13 | 0.846154 | 0.153846 | 63 | 0.015873 | 0.984127 | ATATTTTAAAATATTTTCAGCGTATTTTATGAAATTAGCATCAGTAGAGGATTGTGTACACTGTGTTTAGGCCACCACAACTAACTCCTCTTCAACAAGGG[C/A]AATGTTATAATATTTTAGATATATTTTAAAATGTATGTAGATTATAGTAGCCATGAAAAAATAAACGCATTCTCTTTTGCGTGGAACATAGGTAGAACAAG |
| comp101295_c0_seq4:464-465 | comp101295 | 464 | T | C | 333 | 0.876877 | 0.123123 | 170 | 0.0470588 | 0.952941 | AGCAACTTCATATGCCAGTACAAGCCGCAGAGGCAAGATGAGCGAGACCCAGCCCCGCCCACCCAAGCTCCAGAAGAGGCAGCTCGCGAGGTCCACAGCGG[T/C]CAGTGTCCGACCTTTTACGCGGAAGTCGGGGGCCTCTGTCTCATGTTCGTCACGTGGGCCGAGCAGACCTGGCACGAGGCACGGCAGACCTGCGGAGACTC |
| comp90381_c0_seq1:562-563 | comp90381 | 562 | T | C | 149 | 0.832215 | 0.167785 | 172 | 0.00581395 | 0.994186 | TGCGAGCGGAGGACGTGACAGGATGCGGCATGAGGCTGGTGTGCGAGCTGGCCGGCCAGGAGGAGGAGGATCTGGTGCAGGAGGAGCTGGCCATCCTGGCC[T/C]TGCTCGGACCCGACGTGAAGCCCGGGGAGGGAGTCCTCCCCCCCGGGGGCGCCAGGGGAGAGTACCTGCAGGCCAGGAACTTCGGGGCACAAGGCGGGGAC |
| comp104741_c1_seq5:418-419 | comp104741 | 418 | A | T | 18 | 0.888889 | 0.111111 | 63 | 0.0634921 | 0.936508 | ACAGGAGAGAAAAAAAAAGTAAAAAAAATGTGAATATTAACAGAAAGGACGGAATTGTGAAAAAAATTGGAATATTACAGAGAGGAGAGAATTAAAAACAT[A/T]AAAAACACAAGATTGAAGAAAAGAAGATTGCAAAAAAAGAAATCTATAAAATTCACAGAAAGATGAGGAAAAAAGTGAAAAAAAAATCAATGATATTCACA |
| comp104264_c3_seq9:803-804 | comp104264 | 803 | C | T | 141 | 0.93617 | 0.0638298 | 9 | 0.111111 | 0.888889 | TTTTTGATCCTATTTTATTACTGCCTAGTCTCATTGACGCTATGAACAACAGTAGAAATTTTAAGAAAAAGCAATAACGATAATGGAGATTTTTGTATCTA[C/T]CAAAATGGATTTAATTAATAATCCCTACAATAGCTGTGTGAGTGTGTATAGTATAGGCTATTTATATAACGTAAGACATGAGACTCAACAGAACCTATTGC |
| comp106260_c0_seq2:724-725 | comp106260 | 724 | T | G | 20 | 0.95 | 0.05 | 64 | 0.125 | 0.875 | GAACCAGAACCCGAAACGAAGGCAGAACCTGAACCAGAACCCGAAGTGAAGTCAGAACCTGAACCCGAACCAGAGGCTGAAGCAGAACCTAAAGCTTCGCC[T/G]GAACCAGAACCTGAAGCTACGTCCGAACCTGAACCCGAGCCAAAATCGGAGGTTCAGCTGGAACCCGAACCGGAAGCAGAACCCAAAGCTACAGAGCCTGA |
| comp105859_c0_seq3:104-105 | comp105859 | 104 | A | C | 402 | 0.0995025 | 0.900497 | 278 | 0.92446 | 0.0755396 | AGGCTAAAAATTGACCCATTTAATAGAGCGTGTGACATGACACAACCTTCACTCGAGGCTCAGTGAAAAGAATCCTGATATTATCCCGGCAGGATTATAAA[A/C]TAAATCGTTTCACCCAAAGACTTGATGATATGGTTCAGCTTCGTAGACATCTATATCAACCTGCACTCTGCCATTGCTCCTAAGAGTGCAACAATCAACAA |
| comp107876_c0_seq3:941-942 | comp107876 | 941 | T | C | 73 | 0.849315 | 0.150685 | 41 | 0.0243902 | 0.97561 | CTGGTGTCTGCTCAAGCCAAAGCCAAGGAGGACTTGGTTGGGAGACTTGGTCTCATCTGCGGGACTTCCACCTGCCACATGTGCGTGAGCGAACAGCCCAT[T/C]TTCGTGCCAGGAGTGTGGGGACCATACCTCAGTGCCATGGTGCCCGACTGCTGGCTCAATGAGGGAGGCCAGAGTGCCACTGGTGCTCTTGTCGACCATGT |
| comp96482_c0_seq1:275-276 | comp96482 | 275 | C | T | 224 | 0.111607 | 0.888393 | 63 | 0.936508 | 0.0634921 | AGCAATTTCTTTCATCGGAAGTGCCAAGTAAACAGTGCCATAGTTACTGTTTTATCAAACCGCCATTTTTTATTCTAGTTTGATCAGTTTTATAAAGGTGA[C/T]ATTTAGTTTTGTTTTCCGTTCATCAAAGAGGATTCTGATAATATCTGGTAATTTTAGCTCAACTGAAGAACAAGGTAATGAAGGGAGGACATAAAGAGGGA |
| comp102844_c0_seq5:411-412 | comp102844 | 411 | G | C | 201 | 0.925373 | 0.0746269 | 68 | 0.102941 | 0.897059 | AGCACAGGCCTCTCGACGCCACGGTCCGCTTGGCCCAGGGCGTCGCTTCGGCCGGCATCTACGAGTGCCTGGACGCCAAATTTGAAGAAGTTATCGACCCG[G/C]AAAACGTGCCCGTCGGCGCGGACGACGAGACGCCTCTCGCGTGGATCGGCACCATGTTCTACGGAGGGTTGGCCATGCTCATGACAGAGGAGGAGCACCGC |
| comp87028_c1_seq5:558-559 | comp87028 | 558 | A | G | 66 | 0.893939 | 0.106061 | 55 | 0.0727273 | 0.927273 | GTAAGCGTAGGCACAAGCCGGGTGACGAAGGGTCTGGGCAAGGGCGACGCCCCTGGAGGAGGCGCGTACACGCCGGCGCCCTCCTCGCGCGACCCGGCGGC[A/G]ACTGAGACTCGACCAGACTGCTCGTGTCCTTGTGCTCCGAGCGACCTCTTCGTCCGCTCCGGCGACCATGACCTTGGCTCCCACGGGGTCAAAACACCATT |
| comp103398_c0_seq1:873-874 | comp103398 | 873 | A | G | 23 | 0.956522 | 0.0434783 | 81 | 0.135802 | 0.864198 | CATATCTCATAGCTATGATAATCTCAATCATACAGTCAACAAAATGTCTCGTGCTATGTGGAGGTCGTTTAATATGCTTCTTTCTTAAGATTCGGATAAAC[A/G]AAATTCCTATATCTCCCTATCTCTCTCGAAAAAAATGTCATTCGAATCATGGCTCTGATTACAACATGCCTAAAAATATTGCACTAAAATAACAAACAAGT |
| comp106993_c2_seq1:1461-1462 | comp106993 | 1461 | C | T | 53 | 0.830189 | 0.169811 | 86 | 0.0116279 | 0.988372 | AGAATAGGATTAGGTATATTGAGGTTATTTCCTGTGCTTGATAAATACTTGGAAGGCAGAGTCCGGCCTACTTGTTAGATGTTTGAAAGTCTTCATATTAA[C/T]AGTTCACTAATCACCTTTGTATACCAGTGATTGCAAAAAACACGAATAAAAAGATTCCTAACGATAAGGGTGCCATAACAGCAGATTAGGATAAATGGTAC |
| comp105106_c0_seq3:1112-1113 | comp105106 | 1112 | A | G | 118 | 0.872881 | 0.127119 | 36 | 0.0555556 | 0.944444 | GTGGACACTGTGATGTGCAGGGAGTGGTTCCCCTCGACTGCATGTCCCTGGTGGATGAAGCCCCGGGGGAAATAGAGCAGGTCGCCCTCTTCCAGAGTCAC[A/G]TCTAGAATGGGCTCACCGATCTCGTTTTCGTCCAGGTTGCCGCTGGAGTCTACGGGGAGCACCTCCTCTTCAACTCTGGGCTTGTATACACGCCATCTCTT |
| comp105197_c0_seq1:483-484 | comp105197 | 483 | G | C | 54 | 0.0925926 | 0.907407 | 11 | 0.909091 | 0.0909091 | AAAGCTCTCGCCGGGTTCCTGATGATAAGGATGCAAGGAAGATCCGCCCTTATCACCTCGTATCGCTCCTTCATGGTGCTTCTCCTCCTGTAGAAGGCCTT[G/C]CCGTGGGTCTTCTGGACGAGGGTTCGGCCGCTGTCGGGGGCGTCGGCTTCCCCGAGGTAGCCAGATTGTCTGAGCACTTTATCGTTGTAGACAGCGCCAGT |
| comp106107_c0_seq1:3176-3177 | comp106107 | 3176 | A | C | 319 | 0.918495 | 0.0815047 | 341 | 0.102639 | 0.897361 | GAGCATTTTGAAGCAAATTCGGGACGTTTTTTCGAAGCCTCGTTCAGGAAACGAACAGACCGGAAACGAAGCACGGTCGGACAGAAATGCAGCCGAGAGCC[A/C]AGGGAACCAGACTGAGGCCGGAGGCAGCGCCAGGCGGTGGTGGGGCAGCCTCAGGAACAGAGCCATTGTCGCTGGAGTCTTGATATTGGTCAAGTCACTCG |
| comp106422_c0_seq1:966-967 | comp106422 | 966 | A | C | 331 | 0.912387 | 0.0876133 | 61 | 0.0983607 | 0.901639 | AACAGCTGTTTAGCTTGCACAAACATGCCTCCCATCAGCAAGAGGTTAAAAAGCAATGGCAGATTGGCAACTATCTGAAACCAGATATGTCCTTCAGTTGA[A/C]ATTTACCAACTCAGACACCTGTTCCACCTCTAGCTGCTTAGGAAATAATAACTGCATTTATATATATCTTTTAAATTCATGGTTTACTGTAACATGTAGGC |
| comp99880_c7_seq1:465-466 | comp99880 | 465 | T | A | 79 | 0.0632911 | 0.936709 | 65 | 0.876923 | 0.123077 | GTGTTCGTTTTTTTTTTTTTTGTAGGTATAAAAAGAGAAAGAAATCTATTGTGTGCGAAATGCCTTATATTTCGACGACGATACTTTTTTATGGAGAAGAA[T/A]TAGTTTTTTTTATTATGTCATTGTGATTTTCTTTTTTTAAGGATATTTGCTTCTAGCTTAAAATGTAATCTGTAAGTTAGGTAAAAGGTGAGATTAGTTGC |
| comp103725_c0_seq10:361-362 | comp103725 | 361 | C | A | 831 | 0.00240674 | 0.997593 | 4113 | 0.815949 | 0.184051 | TTGTAGAAGTTGTTGAGGATGGCCAGGCAGTTCTCGCCACCTGGTTCACGGTTGTCAGGTTGCCTCTTGCCAGTAAGTCCAGTGTGAGACCAGTTCAGACC[C/A]TTGAAAGGCTTTCCTGAAGGCCAAGAGAAGTCCCTTCCCTTGCGCTGCCCTCCAGTCCATACCCAGGGAAGTCCGGCATCGCCAACAATCTCATTGATGAA |
| comp105811_c0_seq1:1697-1698 | comp105811 | 1697 | T | C | 126 | 0.888889 | 0.111111 | 66 | 0.0757576 | 0.924242 | GGCATCGAGAAAGCGTGCGCCTCCACCATCGCCTCCGTCGGCTTCAACCTGTTCGCGGACATCGAACACGCGAGGAACCTGTCTTTGCCTCTCTACGACCC[T/C]GCGAAGGCCGGCCGCTTCTTCCCTTACTATGGCAGGCAATGAGGCAGGATTTGCTTCGGTTTCGTGTCACTTTATTTGACTGGAGTCTGCGGGGAGGGGGT |
| comp104433_c0_seq2:2205-2206 | comp104433 | 2205 | T | C | 74 | 0.851351 | 0.148649 | 52 | 0.0384615 | 0.961538 | AGGACTGGGCAGACATCATCAACAGCGCACGCAGAGAGACACTCAGCATGCGAGAGGTTGTGGAGAGAAGGTGCCAAGAGAGAGGCATTGTATTTGTACCA[T/C]TGCCAGAGCGCAAGCATGAAGGCAGACCTGTGTACAGGTGTGGCAAACTTAATATATCATTCAACAAAAATGTCATATATGTTCATACAGAGAGAGGATGG |
| comp105183_c3_seq1:732-733 | comp105183 | 732 | C | T | 525 | 0.826667 | 0.173333 | 71 | 0.0140845 | 0.985915 | TTTTCCGTTTGTTGTCTGTTTGTTTACTGGGGATACTAAACAACAGAGAACTATAACGGGAAGGATGAAGGTAAAAACTGATTCATTATTCGTCTTTATTC[C/T]ACGTTTTATTTGATTTTGTTTACAGGTAGAGATAAGAGAAGACAGATACACGAAAAAAAGGGAATCGAGCGTGTTTATTGCTATTTTTTTCTCCGTGAACA |
| comp104328_c0_seq1:851-852 | comp104328 | 851 | T | C | 212 | 0.108491 | 0.891509 | 114 | 0.921053 | 0.0789474 | GGTGCCAGGAGCCCAAGATATCCCTGCTGAGCTCATTATCTCTGCGGTTGAAACGAGCAATATTGCAACTCTACTTGAGGAGGAGCAAGAACCTGCAGAAA[T/C]CGCAGCTGTTGAAGAGGAAATCTCTGAGCCAGCCACAGCAGAGCAGAACATTGCAGAAACTTCAAACGTGGAAACACAGGAAACAGTCATTGCTGAAACCG |
| comp95203_c0_seq2:143-144 | comp95203 | 143 | C | A | 163 | 0.883436 | 0.116564 | 169 | 0.0710059 | 0.928994 | AGGAAAGTGTAATCAAATCGGGAAAAAAGAGAGAGAGAGAAAAGAAATAACAAAAAATCCAAGTATGATCATAAGGTTGGTACTTTTCGTCAAGCCAAAAG[C/A]GTCTAATATCAGTGCGTCCTTTAGAAACGTGGAACTTTGAGGCCAAACTTCTGCCTAGGAGGAAGAGTGGAGGTTTTAGTTACCAGAGGTGTTGCACTTTT |
| comp106393_c0_seq2:182-183 | comp106393 | 182 | A | T | 35 | 0.114286 | 0.885714 | 204 | 0.926471 | 0.0735294 | AAAATACACGTTTTAACATGTAAAATCTCAATACAATAATCTGTATATGTATGACTATTTTAAAGTTTTCTTTGTATATAAACCATATTCAGTTACAATGA[A/T]GTAAGCTGTATTGTTCCTTTCTGTTCAGTTCTCATTTATAGCTGACTTTTAAGTAGCCTTGAATTATTAAAATCAGTACTGAATTATTATAATATAAAAGT |
| comp102092_c1_seq12:659-660 | comp102092 | 659 | T | C | 243 | 0.901235 | 0.0987654 | 56 | 0.0892857 | 0.910714 | GAATAAAGATATAGAAAAACAAATTTGAAACCCTGCACACTTCCACTAGGAGTAGAGGAAAAAATATCATTGAGACACACAGTTCTCTCACACTGCCCTGG[T/C]GCACTACAAATGAAAAAATGAAAAAAAATAATAACAATTAGGTCACACTACACATGGGCAGATTAAGAGAGGACTAACAATCCTAAAAAGCCTTCAGTGCA |
| comp76861_c0_seq3:650-651 | comp76861 | 650 | C | T | 92 | 0.0978261 | 0.902174 | 806 | 0.909429 | 0.0905707 | GCCTCCAGCGCCACCGAAGCCTCCAGCGCCCCCGAAGCCTACAGAGCCGCCGCCGAAGCCTCCAGCGCCGCTGCCGAAGCCTCCAGAGCCGCTGCCGAAGC[C/T]TCCAGCGCCGCCGTGTCCTCCGAGACCAACGGAAAGGCCGCCACCAGAGGGGGATGAAAGGTCGTATTGGGGCTTGGCTGCTGCCACAGCCAGCAAGGCTG |
| comp99819_c0_seq2:1446-1447 | comp99819 | 1446 | G | A | 141 | 0.93617 | 0.0638298 | 8 | 0.125 | 0.875 | TTGTCCTCGTCGAGCAGACGGTGAACGCGAATGTCCACGCCGTCCTGGTGCCTCGGGGCTCTCACCTTCGTCTCTGAGAGGCGGCTTTCGGATCCGTAGAC[G/A]GCGGCGAGGGCGTCGCGGGCGTTCTGATCCAGGAAGCTGGTTCCGAGGGCGTCGTCCAGGTCGATCACCTGAATCTCAGCGTTGTCCAGCACGCCGGAGTC |
| comp103335_c0_seq10:150-151 | comp103335 | 150 | G | A | 210 | 0.819048 | 0.180952 | 352 | 0.00852273 | 0.991477 | CTCTTTAACTCCGATTCCAAACAACCCGGTATCATGAACCCTCTTTCGCTCCGAGGTGTCTCCATGCTGGTCCTGGTGTGCGTGGTGTCCGCCGCTCCGCA[G/A]GGATACGGTGCCGGAGGCGACGGCGGACTGGGAGTCGATCTCGGCGGCGCAGGAGGCCACGGTGGTGCAGGAGGCCACGGCGGCAGCGGCGTTGGAGGCGG |
| comp104231_c1_seq1:263-264 | comp104231 | 263 | G | C | 507 | 0.871795 | 0.128205 | 211 | 0.0616114 | 0.938389 | TCGATACTTCGCCAGAAATATCGAAAAACACACAAAGAATAAGCTCCTTTTCACGTCTTCCCATTTTCCGCGACGAAATGCCAAAAAAGGAGAAACAAAAT[G/C]GCACGGGAATGCCTATTTTAATCCTATTTTGGTAACTAAAAAAAACGGAACTTTCTCCTCTGCAAAACATATCAGATTAAATTGCAACAAGAGTATCTCTA |
| comp99612_c0_seq1:261-262 | comp99612 | 261 | T | C | 496 | 0.818548 | 0.181452 | 677 | 0.00886263 | 0.991137 | AGTGGTAAACAACCCCGGCCACGCTGTCTCCTACCGTGTGCACTAAGGACTGTTGTCCCTTGCAAACCTCCTGTGTGCTGTAAAACTCCAAGACATACGGT[T/C]AGGGTGAAACAACTGTGATTAACTCTTGATTTCGACCAAATTCCGTTGTGTCTTGAGCTGACGACATTCAGGTTGGTGACTTTCTCCTTGCAGTAGAGAGC |
| comp97533_c0_seq1:501-502 | comp97533 | 501 | C | T | 180 | 0.844444 | 0.155556 | 57 | 0.0350877 | 0.964912 | AGGCTATAAAAGGATAAAAACAAATACAAAAACAAAAACAAAACAAAAAAACTAAATGACATAGAATAACAGGTAACGATCAAAAAATAAAAACGTTTTTT[C/T]TTTCTTTCTTCTTATACTCGAGGTCCCTTCTTATCTTCCGACCATTGTTTTATAATCAGTTCGTTGCTGTGATATTTTTTTTCTTTTTTAAATGGAAAAAT |
| comp100967_c0_seq6:1193-1194 | comp100967 | 1193 | A | C | 53 | 0.90566 | 0.0943396 | 41 | 0.097561 | 0.902439 | AAGAGCTTGTCGAAGTGATTTATTGGAAAAAATATTGATTTCTAATCATAGCCATTTCATCTTCCATGAGGGTCAATAACCTCGAAAGCCTTGTGCGCCCA[A/C]AATGTGCAAACCATGGAAGTTTCAGCAATCAACTTCATTTCAGGTATCTTAATGGCTCACAAGTACTATCTCGCGTGTAACTTGCTATCCCTGAAAAACAT |
| comp97916_c1_seq1:957-958 | comp97916 | 957 | A | C | 121 | 0.884298 | 0.115702 | 13 | 0.0769231 | 0.923077 | ACCACCCGCTGCACGTGCTCCGAGGCGAAGCCCGGGCCGAAGTAGCTCTCGTCGTCTAACGTCGTCGTTTCCCCTTCGTCGGACCTGCGGACGGCAAACTC[A/C]GAGAAGTCTCTAAAATTCGAGAAGAAGTCGTCGACGTAGTTATTCGTCGGCGGAGCATTGTGGGAGTTCGGGAACACGGCGTAGCCATCGTCGAGGAAGTG |
| comp100339_c0_seq2:254-255 | comp100339 | 254 | T | C | 747 | 0.883534 | 0.116466 | 26 | 0.0769231 | 0.923077 | AGAAATGCGAGTGGATTACTCTCCTAATTTACTATTTATGATCAGTAATAGGTCAGCAAAATAGTATTCACGACATGGGTATATATGAAATATATTAATGA[T/C]GTCCCTCATTGAAGGAAAATAATGAAGTGCCAACAGAAAACCATCTTGTTTATATCTTGTCGTTTGGATCATTTTAGCCAGAAATGTATTTTCGTTGTCAG |
| comp104351_c1_seq3:926-927 | comp104351 | 926 | G | A | 92 | 0.923913 | 0.076087 | 51 | 0.117647 | 0.882353 | CTTCGGCGAATCGATTTTTTTCTTCACAATACACAACACGTCAACACCAACACGCGCACACCACAGCTCCGAGGATCCAAGGAAGCGAGAGAGAAGCCAAG[G/A]GATTATCCGAAGGCGTCCCCGCTCAGAAGGACGCGGCCGAGAGCTCCTTGCCCGGACGTCTCGGAACCCGCGCGGACGTCCCAGCTCGACCTGGTGTTGGA |
| comp97939_c0_seq2:455-456 | comp97939 | 455 | A | G | 21 | 0.952381 | 0.047619 | 123 | 0.146341 | 0.853659 | ATTCAGCAAACAACTACCCTCGTTAATAACAGAGTTCAGACCCAAACCGTCCCCAGTCCTCCCATTGTGCAGACCCAGTACGTTACATCCACCCGTGTTGT[A/G]CCACAGGTCAGCTACGTCACCCAGACACAAACTCGAACACAAGTTGTACCTGTTGAAGTGACCCGCACGCAAGTGCAGACCGTCAATCAGCCTGTTGTCAA |
| comp107319_c3_seq1:553-554 | comp107319 | 553 | A | G | 17 | 0.823529 | 0.176471 | 55 | 0.0181818 | 0.981818 | GAATAAATTGAATGAGCACTGCGCTCCGCCTGGATCAACAGTGCTTTCGCAACTCGGATGCTTCCCTGGTCACTGTTGGTACGAAGTTCAAGACGAGAATA[A/G]AATGCAAGAGAACTTTGGCATTCGAGGGAGGTGGGACAGGGGACTTTTACGTAGGAACTTTCTTTGAATCATTTTTTGAAAACGACGATGATTAAGGACAC |
| comp106989_c0_seq15:118-119 | comp106989 | 118 | G | C | 129 | 0.930233 | 0.0697674 | 8 | 0.125 | 0.875 | TCTATATATTTCAGAATATTTACTGCAAACACCGAAAACACTCAAAGAAAGAGGAAAGAACATACACAAACAACAAAAAAAGAAAAAAAAAAACTAAAGCA[G/C]ACCTGGGCAAACTACGGCCCGCGGGCCACATCCGGCCCTTTGTAATTATATATTTACTGAATTATATATTTAGTATGTAATATAGTAAATATATAATGCAC |
| comp101421_c0_seq10:4075-4076 | comp101421 | 4075 | C | G | 117 | 0.128205 | 0.871795 | 119 | 0.932773 | 0.0672269 | TTTACGTGCTTGTCTGAAGGCAGGGCATTGTCGCATCACATTTGTCCCGCATGTGTGGCGCTCGCAACCGCCTCGCACGCCCGCTCGCCCGGCCAGGTTGC[C/G]GTCGCGGTCCCCCGGGCTTATCTGTGGCTGGGCGGACCCGTAGCCCGGAACGGGTTTGGCATCCCAGGCTTTCTTTCCTCTATTGCTTCATATGCTATCCT |
| comp96175_c2_seq1:242-243 | comp96175 | 242 | G | A | 115 | 0.817391 | 0.182609 | 76 | 0.0131579 | 0.986842 | AAGTCTAAAAACGTCTATACATTGGTATCTTTTCTTTGGAAGCAAGGAAGAGAGAGTGATGGAGAGAGGAAGAGGGTGAGGGAGTGGGAGAGGGAGAGAGA[G/A]AGAGGGGGGGGGAGAAAGAGAGAGAGAGGGTGGAGTGTATATACTTTATATGTTAAGATAATGGAAACAAATTGCAACTTTTGCCTGCCATCTTGAAAATC |
| comp103049_c1_seq1:885-886 | comp103049 | 885 | A | G | 89 | 0.831461 | 0.168539 | 69 | 0.0289855 | 0.971014 | TTTATGCTGCAATGTAAACAAAATAGCAAATGTTCTCGCGCATTTTATTCTGTATGTATATTTCGTCATCCAATCGTACTAGAACAAATTGCAATAACGAA[A/G]CCTTTTTGTTTTGTTTGTTCTTTGCTAGAACTAAAAAAATAATGTTTTACGCATGTTTTCCTCTGTATATTTCGTCACCCAGTACTCGAATTCAAACAGCT |
| comp96780_c0_seq2:5092-5093 | comp96780 | 5092 | A | C | 77 | 0.883117 | 0.116883 | 74 | 0.0810811 | 0.918919 | GTTATTTTGCAGATTGTGATTTATGTCTGACAACTTAATTGGTATGGAGGAATAATTAATCTGGGTTTTATTTGTAAACCTAAATTATTTGAGAATATTTG[A/C]ATTTCTCTATCCCTGGCTGGGTAAACGCTTGTAATATTGTGTATTGATAAGAATTTTTGATGTAATCATGAAAACTCATTTTTGTAACACTCATGTTAATG |
| comp105246_c0_seq3:506-507 | comp105246 | 506 | A | G | 77 | 0.935065 | 0.0649351 | 45 | 0.133333 | 0.866667 | TTGAGACTTGACATACTCTTATCTTTCAGAATGCAGTCATAGAAAACTTTAATATATACTGAATTCCACTTTTTCTATGGTAATTAAATAAACAATCTAAA[A/G]GTACCCAAAGACACACAAAGTATCTATAGATCTGGGCAGTAAGTCCAATCCATCTCATAACCCCAACCAAGATGGTGCTCCCTGCCGATGCTTGTGTCTGT |
| comp102304_c0_seq1:294-295 | comp102304 | 294 | G | T | 339 | 0.831858 | 0.168142 | 1754 | 0.0302166 | 0.969783 | ATTTTGCAGGGAAACAGGCAGGCAGATCTTCGCTGACAGCCGCGATGCAGTCTGTCAAAGCGCTTGCGTCGACAGTTGATTCATCCCGACAGTCGTTCGCA[G/T]TGTCGTAGCAGTCGCGAAGTTGATTCTTCAGTGTGTCATCCTCGCCAGCGTGGATGATGTCCCCGACGCATTTCGGGCATTCTTTATTCTCACCACACAGA |
| comp103722_c0_seq2:858-859 | comp103722 | 858 | A | T | 110 | 0.827273 | 0.172727 | 39 | 0.025641 | 0.974359 | AAGGTGCAGTCGCATTCCACAGGATTCCCGTTGTATTTGATCGTGGTTCCATTGATAGCTCTCGGAACAAACTCGGGGTAGTAATGGTGGAGGTTATTGTT[A/T]GTAAGATCCAGCATGGTAATGTTCTCGTTAGCCATAAACACTGACGCAGGCACGGAGATGAGGGTGTTGTTCTGAAGGTACACTTCCCGGAGGGCCCTTCC |
| comp104644_c1_seq8:904-905 | comp104644 | 904 | G | C | 59 | 0.881356 | 0.118644 | 25 | 0.08 | 0.92 | GCTCTCGAGTTATTTGTTCATTTGTCTAAGTGATAACAGGCGTTCGTATTTACTATGGTTGTTTTGTTGTTCTTTGTTGTTTTGTTTAGATTTGTATATGA[G/C]TTTTTCAGTTTGGTTTATCTGCAATTTTGGTTTATTTTCACGATTGTAGCAACTGCTCATCTATAACTTCTGTGATATGTAATTCTTGTGACCTGGATATA |
| comp105509_c1_seq3:945-946 | comp105509 | 945 | T | C | 102 | 0.931373 | 0.0686275 | 23 | 0.130435 | 0.869565 | CGACAGGGACACACACGACATGAGGAACTCCACCGGGTGCGACCACTGCTGACGGCCTGTGTCCTCGCTCGTTCCATCGTCATCTTCGGGCATATCTATGG[T/C]CGTCCGGGGAAGCAGCTCCGCCCCGCTGCTGCACATCAGGAGGTCGTCGTCGAGGCTCATCTTTTCTGCGACTGGCGAAGATTTCCGTCCCGAGTCGCCAG |
| comp98471_c0_seq1:301-302 | comp98471 | 301 | T | C | 58 | 0.0689655 | 0.931035 | 46 | 0.869565 | 0.130435 | TCCAAAGGGCCGAGTAAATGAGTCCTCCCCGATTGCCTCGCTCTCGGAAATCTCAGCCAAGCGACCAGATATCTGTCGAAAAGGAGCTTTATTGCTGTGTA[T/C]CCTGAGGCACAGTTCCTAGTAACAGGATAAGTCACACGCAGTAAGAAGAGACAAATACTGTCTCTATGGTCTTATAACTCTGAATGCTGGCCAGAATGGCG |
| comp104330_c1_seq14:222-223 | comp104330 | 222 | A | T | 144 | 0.888889 | 0.111111 | 79 | 0.0886076 | 0.911392 | CAGGTGTGTGGCATATGTCCCGTTCGCCGTGAAGTCCGGTTCTCTCTGGTCGCGGAAGTCATAGCCGTCAAATTAGTCCGCTTCAGAATTATTCTTACTGG[A/T]GACAGTGTGACTGAAGTGGAATTCGAAGCCTCCATAGTAGCCATAGAAGGTGTCGAAGCCCCGTTCGAGCGGCGTGTAGGCCCAGTCGCAGAAGCCGAGGT |
| comp105300_c0_seq1:292-293 | comp105300 | 292 | C | T | 74 | 0.851351 | 0.148649 | 39 | 0.0512821 | 0.948718 | CAGGGAGGCCCACTCAGGGGATTCCTTGTAGGCAACACAAGAGGCACAAATCTAATCCTTGGATCCCTCTCGCATGCTTTTTCTACTCGCCCACTTCAGGT[C/T]CTATGCGGGAACTTTCAAGAAATACGATTACTTCTTACAGTGTGCATCTTGATAAAATCAACGTTAACATCAATTTAGAATACAAGAAAATACTTGTGTAA |
| comp99639_c2_seq1:361-362 | comp99639 | 361 | A | G | 294 | 0.911565 | 0.0884354 | 608 | 0.111842 | 0.888158 | ACTAGAAAATAAGGAAGCCAACAGTAATAGAATTCGGTGGAAGAGAACGAAAAGGTAATCGTTCTCCTTTTTCCAGTTGAGATTTCTTCGTATGAGAGTCA[A/G]AAAGTAATCATTCCCCCCTATTTTAGAAAAAAAAATGAGGGAGGGACAAAAACCTTCACTCCCAAAAATTCGGCAGAAGAGAACCAAAAAAAGATTTCTGA |
| comp99658_c5_seq1:642-643 | comp99658 | 642 | G | A | 304 | 0.799342 | 0.200658 | 2 | 0 | 0 | TAGGTGCCTCCCAGTGGCTGGATGTGGCATAGAGCTGTAATAGATGTGTAGTTTTATGTTTGTAGGTAGGCCTCTACATACACACCTGTATGTGTTTCAGG[G/A]CCGGGAAAAGTGTTAATTTATTGTTAAAAAATATAGTGCAAATAAAGATCAGGCCATAATATACATAATAAACATACAACCTTTATGTTATAGATATGAAG |
| comp106860_c0_seq1:499-500 | comp106860 | 499 | C | T | 66 | 0.924242 | 0.0757576 | 16 | 0.125 | 0.875 | ATATTTAGATTTTATTTATGTAAAGAATGTAAACTTGAACATTCCCATTATTGATATGAGAAATCAAGTATGTGAAATAATAATATTCAATGCTGAAGTAA[C/T]ATGCCTAATGAAGAGTAATGTATTTCAAACATGGTTGTTTAATTATCTGTGATTTATCGCTTGTTAAGTTATTGAGTAGATATAAATGTTTTAATTCTCAA |
| comp104291_c0_seq1:440-441 | comp104291 | 440 | C | T | 135 | 0.940741 | 0.0592593 | 7 | 0.142857 | 0.857143 | CTTTTTCTATTTCCGAAAAGAAGATAAAATGTTGATGTGTTAAAACGCCTGTGTGTTTGTACGAATCACGTAATTCTAAAACGCAAATTATATTTCATTCT[C/T]GGACAAAACTACGAACAAAGATTATTTTGAAAAACGTACTTCCTTTTAGAATGTTTTTTTCTTTGTGACACAAAATACTTATGAAGATTTAGAGTTAAAGT |
| comp103716_c0_seq2:396-397 | comp103716 | 396 | T | C | 117 | 0.897436 | 0.102564 | 10 | 0.1 | 0.9 | TACGAGGGAAATTTTCGCCGCACCTTAGTCCAAGCGAGCAAAAGTCCTCCGTTTGTCTTTGAGGAAGAGGAGGAGAAGGAAAGTCAGAGAGAGGAATAAGG[T/C]AAGGAATTAACGGAGGAAGAGAAAGATGAGGAGTGCAAGAGAAAGCGAAGGAAAAGAAAGGAAGCGAAAGAGAAGGCAGCGGGGAAATGAAGAGGCTGAGG |
| comp106108_c3_seq2:1127-1128 | comp106108 | 1127 | A | G | 330 | 0.80303 | 0.19697 | 178 | 0.00561798 | 0.994382 | TTCACTAATCTGACAGAGGATGAAACTGCATTGGCGCTGCCCGTGCGGAGACGTTCTGGGAACATTTCTGTGAGGCCCCATACTCTTTCCGCAAAGCTTTC[A/G]TCAAAATCCTCGTCCTCTTCCTCGTCCTCGTCTTCGTCCAAAGGAAGCGTGGACACCTTCTTCTCCTCAGCAGACGACACCTGAGCCGCCGCAGCTTCCAC |
| comp105884_c0_seq1:920-921 | comp105884 | 920 | T | G | 69 | 0.797101 | 0.202899 | 2 | 0 | 0 | CTCGTATAATAAAAAATCGGCCAAATCGGCTTCAAACCGGCCATGGATTTAGGTTAAGCTTCGTCTTTAGGCTAAGAAGAGTATCGAGTGGAGGAACCGTG[T/G]TTGATTTTTAATAGTCTGGTTAATTGGGAATTATCGTCTTATCAGCATTAGATGAATTGTTGATATCGATGGGGGAGCAGATTGTTTTGGAAGTGAAAGCT |
| comp107199_c1_seq1:212-213 | comp107199 | 212 | C | T | 63 | 0.825397 | 0.174603 | 35 | 0.0285714 | 0.971429 | GTTCCCCTTCACTTTATTCTCTTCTATGTCGCTGACAGAGCATGGACACACACACCCGCAAGAGCCTTTGAAGAGGATTTGCTCCAAGACGACCGACATGA[C/T]CACACGCGCCGACTGTCAGAGGAGGAATTAAATCGGCAGTGCCAAAGGGAGGCCGCAAGCAGGAGGAGCCGGCACTCTGAGGCTCAGTGCTTGCCAAGGCG |
| comp107869_c0_seq1:302-303 | comp107869 | 302 | C | G | 868 | 0.902074 | 0.0979263 | 556 | 0.106115 | 0.893885 | CGCATGTCAATCAGTGCCAAAAAGGTTAATTACTATAAACATAAACGTCATCAGGTAAAACAGATCCACGGGTGACAGTGGTGAAGATATCGTCGCCTTCT[C/G]TCATCATCTGGACTGAAGTCTGTATCGCTCTTGTACCGTCCGATAAATTGCGACGTAAAACCGCATTTTAATTCCCACGAAGTTAGAGCAAAGTCTTCCAT |
| comp105947_c0_seq5:1670-1671 | comp105947 | 1670 | T | C | 39 | 0.0769231 | 0.923077 | 55 | 0.872727 | 0.127273 | ACATGCAACCTTACTATTCAACTCCTACATTTTCCAAACTTTATTGTAAGAGATGTAGAAAAAAATGTAAATGACAACAAAGAACAGCACAAGATCTGTAT[T/C]TGAGGCATTTTGATTTAATCTTACACAGAATTACACAAAATTCTGACAATATAATTGAAGTGTGTCAAATACATAACATACAGTGTGAAGTTGTTTCAATT |
| comp104293_c0_seq3:3929-3930 | comp104293 | 3929 | A | T | 192 | 0.864583 | 0.135417 | 145 | 0.0689655 | 0.931035 | AAGCTGAGGGCAAAAAAAGGCAAAATGATGTAGAGACCACATTGCATTAAAACCAATTCAGGGATAAAATATTGGTTATCCAAAAGGATGAAGCAGCTCCA[A/T]CTGAGACGAGGCAGCTGTTAGGCCGAGTAATGATGTCCAGATTCAGATCACTGTCTCGAACCACTGGTGAATGCTGGATAAGTGAAGGTCAGCATTGATTT |
| comp107885_c0_seq1:5728-5729 | comp107885 | 5728 | T | G | 245 | 0.84898 | 0.15102 | 240 | 0.0541667 | 0.945833 | GAGCGAGTGCGGAGGGAACTCACAGGACTCCCAAGTCACTTCGGGTTCGAGGCTCTGCTGGAGGACGTGAGATCCGCCGGGAGTGGCAGTGCGGCGGCCTC[T/G]CAGCTGAAGGAGACGGTGGAGCGGTTGATATCGAGTGCGACTGAGAACATGGAGGGGAAAGTGAATGAGTTCATTGAGGCCGTTATGGAAAAGATGAAACC |
| comp107713_c0_seq1:4090-4091 | comp107713 | 4090 | G | A | 283 | 0.904594 | 0.0954064 | 363 | 0.110193 | 0.889807 | ATTAAAGTTCCAATGAAAATAGAAAAAAATGTTCATGTGTAATACTGAAAAGAAATATCAGTATTACATTTGAAAACTCCACTCGAGTGCTCTTTTAGGAT[G/A]CTGTTAAAGAAACCAATATTTTTTTTATATATGAAAAATGCATAATGTATCAGTACATCATATAAAAGAATTTAACTTTTAAATCATATGAAAAAATATGA |
| comp100606_c1_seq4:218-219 | comp100606 | 218 | C | G | 92 | 0.815217 | 0.184783 | 48 | 0.0208333 | 0.979167 | ATGGAAAAGATTGGGAATGTAGAGAACTAAAGTAAAGAAGAAAACATACGCAATATTCATATGTATTTATGTATAAACTGTATATATATCATAATCACTCA[C/G]AAATTTTTGAAAATTGCCAAGGACAAACATGCGATTTTTTGTTAGCAAGGGAAAAAGTGGGAATAAAAATAAACAATGGCTTTGATGAAAAACCAACAGCC |
| comp107782_c0_seq1:154-155 | comp107782 | 154 | T | C | 29 | 0.827586 | 0.172414 | 60 | 0.0333333 | 0.966667 | TCAATAAAGGGAATACCACATATCTTAATACAACTTCCCTGATGTATTACCCAAACTTCTCTGTGCTACACTCATACCTACCACAATTCACACCTATGGTA[T/C]TTCTAATAATCATAACCTGTTATAACGCTACTGGCTATACTGTCTCAACTTTCATCACCAAAAAATCCTGAATTCTATAAAAAATAAACTAGCATCTAGTG |
| comp104705_c0_seq2:480-481 | comp104705 | 480 | T | C | 156 | 0.903846 | 0.0961538 | 9 | 0.111111 | 0.888889 | ATCTACTGGAAGTTCTGCTGGCTCGTTTTTATTCCGTTATCCCTGAGCGGCATCCTGGTGTATGTGCTGTGCAGTATGGAACTTCCCACAATGGACGGACT[T/C]TACTATCCTATCTCTGCTTATGCATCTGGATGGATCTTGGCAGGCTTGGCTCTTCTGATGGTGCCAGTCGGGTTCCTTCACGCTCTCTACATTGCTGATGA |
| comp84269_c0_seq1:381-382 | comp84269 | 381 | A | G | 23 | 0.956522 | 0.0434783 | 67 | 0.164179 | 0.835821 | TTCTGCACAGACTACGTCCACTGCGTCGCCGGCGTCCCATATGTCAAGAAATGCCCTTCAAACCTGAACTTCAACGCTGTGAAGGGGGCGTGCGACCACCC[A/G]AGGGACGCCCACTGCACGCCCTTCAAGACGTCCTGCGAGCTGACGAGCCCTTTCGTCCCAGGAGGCGTGACAGACGACCTCGTGACGTGTGACTGCGAAGG |
| comp102001_c1_seq1:2804-2805 | comp102001 | 2804 | G | T | 77 | 0.792208 | 0.207792 | 2 | 0 | 0 | GCACACACTGATTTTCCTCGGATCAAGTTTTTTTTCTTTTTTTTTTGACGAATTTCCAGGAAAATGAGTGACGAGTGATTTACATTATTTTCCCCTCACAT[G/T]ACTAGATTTTGGTAGAGCCATATTTTCAACCATTTTACGTACTTGTATTTTTTTTTTCTCGAGAGCACCAAATATATTTAATTGGATGTTAAACCTGAAAT |
| comp96363_c0_seq4:483-484 | comp96363 | 483 | G | A | 239 | 0.790795 | 0.209205 | 2 | 0 | 0 | TTTAGTTCGTAAGGAGTAAGCTCGCCCAGCAGGCAGAGTCAGTGAGAGGAAATGTCCCTTACAGTCAACTTTGTGTTAGAGCCAAAACGTGATTATTGTGC[G/A]AGTGCGTTCGTGTAAAATGCGTGCATGCGAGCGCCGGTGCGTGTTCTTGTCACGAACGATCCAGGCTGATCGTTCTGTTCTGTCTCCTCTTTAACCTCGTT |
| comp106289_c1_seq1:1025-1026 | comp106289 | 1025 | T | A | 80 | 0.925 | 0.075 | 52 | 0.134615 | 0.865385 | AAAAAAATCATCTATCATTTTCTGCCCTTTTTTTGCATAAAGACAATCTCTGTAACACCTCTAGCCTGTATACTAGTTTCCATCTTATCTAAAGAAAGTCA[T/A]TCAGCATATACAGTCTTAAGATTACCATTACAACATCCACAGTAATTCCAGAAGAAAAACTGAAAAAGGAAAAGAACGAGTCATGAAATCGCCCCAAAGGC |
| comp101882_c1_seq18:746-747 | comp101882 | 746 | A | C | 71 | 0.943662 | 0.056338 | 39 | 0.153846 | 0.846154 | TCTCGCTTCATGGGTTGTTTATTACGATAATACACGTTGATGGATAAGTCATTATGCCATATTTGGTTACAATACCTTGTCCTTGGAAATCTTGAATAGCT[A/C]GTAGGCGGAGAGGAACAGGGAGGGAGGAGGGAGGCAGAGGGATAGACAGACGAATAAGGAGACTGTCAGAGAGACAGAGAGAAACAGAGGAATATACGAGA |
| comp104587_c3_seq5:730-731 | comp104587 | 730 | G | A | 251 | 0.840637 | 0.159363 | 59 | 0.0508475 | 0.949153 | CCAGAGGGCCAATACCACTGGTACCCGTTGCGGTATCCTCCCGTCCAGATGTACTTCACTTCGTCTTGTGCAATGATGCTAGCGATGATCCTGTCTTCCAG[G/A]TAGCTCTCGATGCTGACGCCTTGCCACCCGTACCCCAGGCTGGCGCAGTACCTGTTGGCGGCGCCCCACGGGTACTTCTGGTCTCCGTCGTGGCGCCAGGA |
| comp100599_c1_seq1:239-240 | comp100599 | 239 | C | T | 170 | 0.805882 | 0.194118 | 62 | 0.016129 | 0.983871 | AATTATGATCCCAATTATCGACTTTATCCCGAATATTTGAGCATTCGTAGTCGAGAGACAGGACCTCCTAAAGTGCAAAGGATAGCGTGTTGTCTTGATTG[C/T]GAAGGATCTTGGTACCTTTATTTGCACTGACTTCTGGCGAATCCTGTTTGACTTATTCATTTTGTTTTATTTCATATTTATTTATTTATTTATTTATTATT |
| comp99942_c0_seq1:171-172 | comp99942 | 171 | C | A | 83 | 0.819277 | 0.180723 | 33 | 0.030303 | 0.969697 | TAAATCCCCATACATATATATGTATATAATAATCCCCATACATATATATTTTTAAAATGAAAATGAAACAGAACATAAGCAGAGCATTTGTGGCTGAAGAG[C/A]ATATTGAAACGAAAATCTGACTTGAGTTCATCACACATGTCAATAAAGGCTAATTGTCTACGCACTGACCCGTTATTCTGAGCCTTAAACCTTTGGGGTCA |
| comp103549_c0_seq1:574-575 | comp103549 | 574 | A | G | 172 | 0.872093 | 0.127907 | 24 | 0.0833333 | 0.916667 | AATGGAAAAGATGCGGTGTATGTATGTTTAGATAATTCGTATATGCATGAAACATTGTACATCAACCAATTGCATTACAGTCGGTCAATGTCTTTGGAAAG[A/G]ATCACACATGCTTTGCTCCCATAAAATACATCGGGAACACATATCGAATTATAAAATCTCGTCATTTGATGACAAATATGGAAACATGGTATTCGACCCTG |
| comp107657_c0_seq15:3036-3037 | comp107657 | 3036 | G | T | 52 | 0.942308 | 0.0576923 | 39 | 0.153846 | 0.846154 | GACCAAAACCTCCCGGCGGAATTGCTCATGAAGCGCCCTCACCTCTACCAGCGGATCGCCAACAACTCAGCCATGTCGTGGCTTATGTTCTTCAAGTGGAC[G/T]CTGTTTGCCCTGTGGCATACACTCGTCATGTATTTCGGTCTGTATTTCGTGTATGCAGACGACACCCCAGGGCTTCCCGATGGCCAGACACCCGACCTCTT |
| comp89496_c0_seq2:313-314 | comp89496 | 313 | G | A | 898 | 0.809577 | 0.190423 | 519 | 0.0211946 | 0.978805 | ATTGCTTGTGAGTCCGCATCTCTAGCCATAAAAATGGGGTCTGCGGAAGCCGTAGCCGTAGCCAAAGCCGCCGCCGAAGTAGGGCCTATGGATGAAGAAAT[G/A]CGGATCTGCCTTGGCTCCGGCCTCAGCTTCGGGGAAAGGCCCAGCCACCACCAGCGAAGCCAAAGCCAGCGCCACCAGCAGGAGGACGAACACACGACTCA |
| comp106471_c0_seq1:2934-2935 | comp106471 | 2934 | A | G | 109 | 0.807339 | 0.192661 | 152 | 0.0197368 | 0.980263 | ATATAAACAAAAGGATTTGAAGGTATGTATATAAGTTTTATTGCTTGACATGGTGGATCCTGAGGGTGCTAGGGAGGTCTGGGCCTGTGCATGGAGAATAT[A/G]TCCCTTTATTATTTTTCTCTAATTTATGTCACATTCATGTTAATTTTATATTTTTGTACTAATGTTATTCAGATATGTTTTATTAACTTATTGGATTATTT |
| comp107145_c1_seq2:2009-2010 | comp107145 | 2009 | A | G | 354 | 0.89548 | 0.10452 | 203 | 0.108374 | 0.891626 | CCATGTTGAGTGTAGCTGGTAGAGGCTTCTCGAGCTGAACGAAGTAATTTGCAAACGAACGAAATTCAGAGAAGAGTGACAAGACTAAACAGAGGAGAGCT[A/G]TTGTCTCTCAGTGCATCGTCCTTTTCGTTCTTTCTCCTTCAGTTCTTTCTGCGAAACTCCGTCAATTCCGTCCGTGAACAGCGTCGTGGAGCGAGCGAGCG |
| comp104376_c0_seq1:150-151 | comp104376 | 150 | G | A | 218 | 0.90367 | 0.0963303 | 180 | 0.116667 | 0.883333 | TGATGTCTTTATCAAATCCCATGGAAACTTATACTCATTTATACCTTTTTCTACGTATGGCGCAATTTCTTGTCCTGTTTATCAAAATAGTTATAATTAAC[G/A]CAATTACTGAAAGAGTAAAGAATTATGTGGCTCAAGATAATACTCAGTTAAAAACGCAAATGCTATTTTTTTGTATTTTTGTACATATTCATCAAATTTAC |
| comp103566_c0_seq4:342-343 | comp103566 | 342 | A | T | 272 | 0.786765 | 0.213235 | 2 | 0 | 0 | GTAGGGCCAGAGACCAAGGCACTTGCCACAGGGCCGGGGGTGACTCCAGCACTAGTCGGGGAACCAGAATGATGAACAGAAATGCCGCCAGGGCTGCCACT[A/T]CCAAAGGACAGTGAGGTTCCTCCAGTCTGGTGGACACCAGAAGTAGCTAGCCCTCCTGTTTGTTGAGAAGCAAAGGCACCTCCACCAGTCTGAGTGCCAAA |
| comp107501_c0_seq4:232-233 | comp107501 | 232 | C | T | 101 | 0.881188 | 0.118812 | 21 | 0.0952381 | 0.904762 | TTAATTGATCGGTATTTCTATGTAAAGACACCTTACCACAGCTGCTGGGTCTATCCTAACGCCATAGGCCTATTCTTGTTTATGTCAGAGTATTTCTCTTC[C/T]TTTTCCATTAACAAGGACACTCCCTTACCTGTCTGTCTGCGAGATATATCTATTCTCCCTCATAATTCTCTGAAGAATAACTTGAATGATTGTGGTAACTA |
| comp101551_c0_seq3:273-274 | comp101551 | 273 | T | G | 56 | 0.785714 | 0.214286 | 1 | 0 | 0 | AAGTCGACCACGTTCGATCTGCTCGAGGGCGTGGGCGGGGATTGGGTGGGGGAATGCAGGGGCGACGGGCAGGAAGGACGACTGAGGTTGGTAACCGTTAA[T/G]ATCGGCGACGAACTGAAGATTGAAGACTTGGCCATCTGGGAAGGTGAAGCTCACGGTTCCCTGCTGGGCGCCACCTGGACCGCCAGACTCGTGCCTGACGA |
| comp82770_c1_seq1:129-130 | comp82770 | 129 | T | C | 96 | 0.833333 | 0.166667 | 84 | 0.047619 | 0.952381 | TGCCCGTCAGATTTATTCGGGCAAACAGCATTGATTTCTCCCCATTTCCCATCGCTTCGGACTGCCATTGTCAAACTACTTCCCAAAACGACGAATCACAA[T/C]GGAAGACGTGCAGCGTTAAGGATAATTATGAACTGTATTTATATTTTTATCATTATTATTTATATTTTTTGCATTACTGTTTTTTTATTCAGTGCAAACTA |
| comp98264_c0_seq1:181-182 | comp98264 | 181 | A | G | 54 | 0.851852 | 0.148148 | 30 | 0.0666667 | 0.933333 | AACACAAGGCATGGCAGGTGTCAACACTTCACCCGAGACATCCCAAATCTATGATCAAGCTTTCCGCTTCCTTTATGTGAAAGTCAAGCGAATGTTCGGGC[A/G]AGTGCTCTACACACCAGGTCCACACAAGCGACTGTGAGTGAAGGAGCTCGTGGCACTCAGTTGAGCACGCGGTAGGAGGTGGCGTGGGCGGGGTTGTGGAC |
| comp107182_c0_seq1:430-431 | comp107182 | 430 | G | C | 69 | 0.913043 | 0.0869565 | 78 | 0.128205 | 0.871795 | GTTCATGTTAGTTTATCACTCACAATCTCAATGTTATAAGTTTTAAGCATGACTAGATACTATGGAACATGTACGAATGAATAAGTATACAAGTAAGAAGT[G/C]TACATGGTATTCCCCCATAACTATATGAAATACAGCTCAAACAGAAGCTTTGTGCCATATGTAATTTCATAAGAAATGCATTACTATCCAGAATCCAGAGT |
| comp107400_c1_seq4:977-978 | comp107400 | 977 | A | G | 106 | 0.839623 | 0.160377 | 109 | 0.0550459 | 0.944954 | ATGCAGCCCACCAGGATGCCGTTGAACACCACGACGCCGTCACTTATGAGGTTCTCCGGCTGGTTGAGCACCTTACTGAACGCAACGCCCACCACGCCCGC[A/G]AGGAGACACGCCCAGAACACGTTGATATTGCCGACGAACATCCCGGCCATTATGAGGAAGCCCGTGATGGGGTTGTTGGCGAACGCGACGATCCCGACGGC |
| comp92711_c0_seq4:289-290 | comp92711 | 289 | C | T | 1231 | 0.805037 | 0.194963 | 48 | 0.0208333 | 0.979167 | GTTAGCCTGCCTGCCGTTGATGTGCCTGTGAGTAAGGTCGTCACGCCCACTGGTTGGGGCCGCCCCTCCGACAGTGTCAGCGGAACGTCCGACGTCCTCCG[C/T]CAGGTGGACGTCCCCGTCATGGCCAACGCTGACTGCGACGCCGTGTATGGCATCGTCGGCGACGGCGTTGTCTGCGTCGACGGCGCCGGCGGCAAGGGAAC |
| comp97641_c0_seq1:230-231 | comp97641 | 230 | T | C | 521 | 0.804223 | 0.195777 | 199 | 0.0201005 | 0.9799 | ACTCATTATACATGACCTTGGCCTTGAAAGTTCATGGCACCCAATAGCCATAATAAAATTGTTACCCCGAAACCACAACAGAAAACAGCTGAGCAATTAAC[T/C]CAACTCTGAAAGAATATATTTATATACGCCGATGTCATACAGTCAGCTGAAAAATATACAGTATTTGAGCTTACAAACTATTTGTATTATGTTTGTAAATT |
| comp101208_c0_seq2:415-416 | comp101208 | 415 | G | A | 683 | 0.81552 | 0.18448 | 218 | 0.0321101 | 0.96789 | GTTCTTTCCCGAAACATCACCGTTCCGTCTACGTGTTCGGCCATGGTTCGGAGTCCCTCCCTCGCGCTCGCCCTGCCTCTCCTCCTGATGGGGACCTTGGC[G/A]CGAGCGAGTTCCCAAGAGTGCAGCAAGGACCCCGCGACGTCCCTGGTCGATCAGCTGGTCGGGCTGGTGGGCGAGAGAGCGGCTCGGACCTGCGACGTCTG |
| comp104626_c1_seq1:2618-2619 | comp104626 | 2618 | C | T | 54 | 0.925926 | 0.0740741 | 14 | 0.142857 | 0.857143 | AGTCAATCGACAGACACCATAAAACCAGAAGATATTAAGGTGGAAGATGAAGATGATGATGATGACATGGACGAGGATGAAGATGAAGACGAAGATGAAGA[C/T]GAAGAGGAAGATGAAGAGGAAGAGGATGAACAGAGCAGAGTGGGGATAAAATCCAAAGTGATGACAAAAAGCAATACTGTTCAGGAGGAAGATGAAGATGA |
| comp102190_c0_seq1:568-569 | comp102190 | 568 | A | T | 55 | 0.818182 | 0.181818 | 55 | 0.0363636 | 0.963636 | TTATTCCATGTGGCTTACCCAGTGGGGAATGGCATTGTAATGTATCGTAACTAGTAACGCCGTGTATTGGCAAGTTCAAGTATATTTCATTTTAAACGAAA[A/T]TTCAAATCAGTGTACTCCTTACTGCATGCAACTATAACCATGACTCTACACGATTTGCTGGAATACTTTTTGTGTTATATTTTGTACTAATATTTTGTATT |
| comp96458_c0_seq9:191-192 | comp96458 | 191 | T | C | 24 | 0.125 | 0.875 | 85 | 0.905882 | 0.0941176 | ATATTTTGATCAATTTCTTCTGTTTCATGCTTCCAATGATATTAAATCAATATAAAAGATTTCAATTTGGTTTAAGTTGTCTGTCAAGATACCAGCTGTTC[T/C]ATGTAGTTATAAACATACCACTAAGCAAAACAAAGTACATCATACTCTGAAAAATTAACTAGTGCTTAATGAAACATCTCAGTGTCATTTGTATTACTACA |
| comp97975_c1_seq1:102-103 | comp97975 | 102 | A | G | 26 | 0.153846 | 0.846154 | 106 | 0.933962 | 0.0660377 | GTGGTGTTGGACAGCATGCAGACTGCGCGAGCTACTTTAGCGAGGTCACCGCCGGGCACCACTGTAGGAGGCTGGTAGTTAATGCCCACCTTGAATCCTGT[A/G]GGGCACCAGTCCACAAACTGGATGGTCCTCTTGGTCTTAATGGCGGCAATGGCAGCGTTCACGTCCTTGGGTACAACGTCACCTCGGTACAGGAGACAACA |
| comp104000_c3_seq2:304-305 | comp104000 | 304 | A | G | 46 | 0.826087 | 0.173913 | 130 | 0.0461538 | 0.953846 | AAGAGAAGGAATGGGAGAGAAGAAGAGACGATCAGGGAGATGGAGAGATGGAGAGAAAAGGAGAAGGAGAAAAAAAAGGAGATAGAGAAAGGAAGAGGAAA[A/G]GGGATAACGAGAAGGAGAGATGGAAGGAGATGAAGAGAAGAGAAGGAGAGACAGAGACAGAAAAGAAGGAACGTGGGAGATAGAGGGAAGAATAGAAAAAA |
| comp104514_c0_seq12:2010-2011 | comp104514 | 2010 | A | G | 117 | 0.863248 | 0.136752 | 108 | 0.0833333 | 0.916667 | ATGCAGAGAAGATCATAAACAGCTGTTGCATTGTCATAGGCATGGACTCTGGCATTCTTGAATTCTGGTGACTGACTCAGAACAGCTTCTCTGACTGCCAT[A/G]GCCTCACTAATAAGCAGAAGTAAGATGATCTCTTCATTGATGTTCTTTGGTACAAAGAGGTTGGTACCAGTGTACTTCTGGGGCTTCCAAGGACTTTCTCC |
| comp107919_c0_seq2:481-482 | comp107919 | 481 | G | C | 427 | 0.922717 | 0.0772834 | 538 | 0.143123 | 0.856877 | TTGTAATAATACAGAGTGTATATGCAGAAATGGCTGAAGCAATATGCATAATGATTTGTCAGGGATTAATGTATAGTTATAGACAAATTGTGACTGAAGTT[G/C]GGAAATTTTGGGTGTGTAAAGGGAAAATGCACTAAGAAACAAGACATTGGAAACTAATAGGATGTATGCAAAAGGGGGTTCATAGAAACCTGTTCTGTATG |
| comp101689_c1_seq4:220-221 | comp101689 | 220 | A | G | 74 | 0.810811 | 0.189189 | 32 | 0.03125 | 0.96875 | GCCCTCTCCGCCGACGCCCGAGCCGACTTCTTCCGTGATACCCGGCCCTTGCACGGGGCTGCCTGGAGAGGGCGACGGGGGGTGGTGGAGGAGCTGCTCTC[A/G]TGGGGCGCCGACCCCGATGCGCGGGATGCCAACGACGAGACTCCGCTGCACCTCGCCGCCGAGAACGGGCAGAAGGCGGTCGTGGAAGTGCTGCTGTCGGA |
| comp97157_c0_seq4:460-461 | comp97157 | 460 | C | T | 2 | 0 | 0 | 145 | 0.77931 | 0.22069 | GAAGCATCGAAGCCTTTCTAGAAGATCCCAAAATCCGCGAGACCTACAAAGAGATCGCGGCCGAGGACAAGGACGAGTGCGGCCTCCGCCTGGTGTGCGAG[C/T]TGGCGCAGAAGGACCCGCGGGAGCTGGACGACGACGAGATCGTGGTCCTGCTGCCCTACAGAGGGCGCGAGGAGAGCGACGAGGCCACCTCCTTCGGCAAA |
| comp100467_c4_seq11:755-756 | comp100467 | 755 | T | A | 32 | 0.875 | 0.125 | 52 | 0.0961538 | 0.903846 | CGCACCAGCCTTGGAGGACCTGGTGCTAGCAATATACTACATATCTATCATCTTCAAACTGGAGATGTATCTAGATAAGGAACTTTCATTAAGGTTTTTTT[T/A]ATTTTTATTGTTAACAATAATTATATTTAATAAAAAATAATAATTAAGATAGGAATATTAATATTACTCACAATATTAATAAAACTAAAAATTATGAACTT |
| comp96329_c0_seq1:142-143 | comp96329 | 142 | T | C | 69 | 0.130435 | 0.869565 | 11 | 0.909091 | 0.0909091 | TACAATCAAAAGTACATGAGTAAAATTTAAGTTTTACAAAAGGAACAAAGCCAAATAAACCTTAATTGAAATCAAGACTGGACTCTCCTCGAGCTCTGCCA[T/C]GACTCTCCAAACGAACTCTCAGCTTCTCAGGCGTAAGCTCCTCCGACGCGTCCACCCCCGATGGGAGGCAGCGTGTGCATGGCCACCTTCGGGACGCGAGT |
| comp105070_c0_seq2:531-532 | comp105070 | 531 | C | T | 53 | 0.811321 | 0.188679 | 91 | 0.032967 | 0.967033 | CAAATATTACTGACATTCAAAAAGAGGTAATTAGTAACAAGTGATTTTCAATGAATTATCTGAAATTACAATATTTGAACAGTGTGCTCTTTGAGGATCTA[C/T]TGGGACACCTTCACCAATTTAAACAATACAACGATGTGACAGATAAGCCTAAGTACACTCCAATCTATCCGATTATTATTGTTTTACCATGGAACCTACAA |
| comp104084_c1_seq1:328-329 | comp104084 | 328 | G | T | 58 | 0.155172 | 0.844828 | 30 | 0.933333 | 0.0666667 | GTTAGTAAATAAATTACACACATCTGAATCCGAAGTCGAGGCTCTATCATCGCATTCGAATGACATGCGGTAATGGTGGTAACTATGTATAGGATTTGCGT[G/T]AAATTGGACGAAAAATGGGATAATGGGGGGGGGGGTTGGTGGTGGGGGATTCACACTGGACATGCTGTGAATTAGAGAAGTGGAGGAAAGACCACGAAACT |
| comp106064_c0_seq1:1972-1973 | comp106064 | 1972 | G | A | 61 | 0.918033 | 0.0819672 | 64 | 0.140625 | 0.859375 | ACAAAACTCCTGGAATTATCATTACTTGTGCACATGTCATAGGTCCAGCAAATAGTGGTGTTGGCCTGTTGTGGTTGCAGGATATGGAGTATTCTCCCCTC[G/A]GTATCTGCCAACACCAACTCTGTCTCGAGGAATAATTTCAAAAACTATAAACTTCCCTGTTCAGTCTCTTCATCTGAGTGAACATCAGAGCTTTGCAGTGG |
| comp98206_c0_seq6:1273-1274 | comp98206 | 1273 | G | A | 132 | 0.166667 | 0.833333 | 70 | 0.942857 | 0.0571429 | TTTTTCTGAATATACAGTGGTACCTGAAGTTTTCTTGTATCCAGCAATTTTCATTTCAAGTAATTTGATGATGATCAGACTTTATCGCAAAATTATAATAA[G/A]TAACTCGTTTAGAAATCAGGACTTCAATCAAAATGTCTTTTGTAATATCAGACCATTTTGGGATTGATTGGATTCTGTTGGAACATGATATTTGATGTTTT |
| comp106517_c0_seq1:727-728 | comp106517 | 727 | T | C | 115 | 0.852174 | 0.147826 | 13 | 0.0769231 | 0.923077 | TTCTTCAGTTCCACCTTTATTCTTCTCCTTTTCATCTGGTCAACACCCTTCCTCCAACGATTTTTGCTCGACCTCCTTCCTCCTTCGCTGGCTCTCCCGCC[T/C]TTCTCTCGCCCAAAGCCGCCTAACCGAACTGGAGGATGTTGGACACCTCGGAGTGGGCTGCGCTCTGGAAGTCCTGCGAGCAGAGGTTGTCGAACCACGCC |
| comp104219_c7_seq1:1611-1612 | comp104219 | 1611 | A | T | 154 | 0.818182 | 0.181818 | 116 | 0.0431034 | 0.956897 | ATAGGTGTTTATATTCAAAGAGAAGACTTAATGTACTTTGTAAGTAAATAATATGTAAAGAATATGAGCCTGGAGATGATGAAATTAAAGGTTTATGAAAC[A/T]GAATACTGAATTATGGGTTATTAATTGTATATTTTTTAAACAAATTCTATTATTGTGATCTGTGTTATTGTGTTTGTGTGTAGACTCTCAAAAAAAGAACA |
| comp95416_c0_seq9:248-249 | comp95416 | 248 | T | C | 367 | 0.912807 | 0.0871935 | 29 | 0.137931 | 0.862069 | TCATTTGTATCCAATAAAAAGTTTGCTTTAACACGTGTAACATTTAGATTTACGATTATATTCCAGCCATGTCAATGAAAGCAAACATGAATTCCCTTAAT[T/C]ACATTTACAAAATAATATACACCAACATCCTTCTTCAGCATCGGCTTTAGGGTAAGGGTCTGCCTCGGGGTAAGGGTCGGCCACGGGGTAAGGGTCGGCAA |
| comp105159_c1_seq1:850-851 | comp105159 | 850 | T | C | 108 | 0.833333 | 0.166667 | 68 | 0.0588235 | 0.941176 | TTGGCTGCCATCTTCGCCTCCCTCTCCTCCGTGAGCATCGGCTTCATCACAGCTTACTCATCACTCACTCTGCCTCAGCTCAGAAATGACACTACTATCGA[T/C]TATGTGGATGCTCGGGACTCAGGCTGGATTGCCTCCCTCCCGAGCATCTCCTCCATCCTGGGCTCCCTCATGGGCGGCTTCGTGATGGACGCCATAGGACC |
| comp104046_c0_seq2:2549-2550 | comp104046 | 2549 | G | A | 93 | 0.774194 | 0.225806 | 1 | 0 | 0 | TGCATCCTCGCCCCATATTTTGTATATATAATTTGTTTGTGTCTATGGAGTTGTTTTTATTTATTGATATTTATTCAAAATCAAACGAGAGGCTTTTCAGT[G/A]AAGTCTTAGATTTATAAAATAAATCTACAAAACAGTTTGAGGAAAATCTTTCCCGAAGTTTATCCCTTCGCAGAGAACCCCTGCTCGCCTGCCTGTGGAGT |
| comp100372_c0_seq11:602-603 | comp100372 | 602 | C | G | 53 | 0.773585 | 0.226415 | 2 | 0 | 0 | CGGTGTGGGAGGTTCCTTCGGAGGCCCCATTGGAGGCGTGTCTTCCGGACGCGTTAGTGGAGGATACGGCCGATAACCATTATCTTATAGAAATAGGAACA[C/G]ATAACGAATAACAGAATAAAAATCATCTATGTTTTATGCATTGTGCTCTATATATCCTATCATTACTGTCTGTTTAAAGGTCCCTCACCCAAATAACACTA |
| comp101693_c1_seq2:301-302 | comp101693 | 301 | T | G | 799 | 0.813517 | 0.186483 | 443 | 0.0406321 | 0.959368 | GGAACCACGACACACGCTTGGGGGTCTCCGAGGCTGTCGAGAAGCTTAAAAGTGACCACAGTCTCGATCGCCGGGAAATCCTTCAAGACGTAGACCTCCAT[T/G]CGCCACAGCACCCGGTCCCCCTCCTGGATCGGACAGGGCGTCTCCGTGCCTTCGAGTTCCTTGCAGCCGTCCGTGTCCAGGCCGGGCCAGGGCACGGGGAT |
| comp103844_c0_seq1:392-393 | comp103844 | 392 | G | C | 391 | 0.882353 | 0.117647 | 73 | 0.109589 | 0.890411 | TCACCTGCAAGGCGCTGGGAGAGGATGGTTGGAGTCAAGTTAGCGGTCGTCCTTGTGGTGCTTGTCGGAGGAGATGTCTCGTTTTCTATGACCATCGGGAA[G/C]GATGCGAGGGGGGACTTGGCTAGGGAGGATGAAGTTGCCTTTGAGGACACAACGGAGGCTGCTGGAGAAATTCCTGCCGCCACCACCGAGGTTCTTCCCGC |
| comp106451_c0_seq7:366-367 | comp106451 | 366 | G | A | 111 | 0.0630631 | 0.936937 | 322 | 0.835404 | 0.164596 | CTGAAAGATCTTGGAAATAACGACGATAAACCACCTACAGGAGAGCTAAAGGCGGGAAGGGAAAAGGGAGTGAAAAGGTGTGATATTTGCGGGTAAAATGG[G/A]GGCCGGTTCGAGGCTAGCGCACACACGGAGGAGAATCACCAAAGCTGGAAAACTGAAAGCGGGGAAGAAGTCGAGGAGATTTGTCTCTTTCATCTTTTTTT |
| comp106690_c0_seq1:1757-1758 | comp106690 | 1757 | C | T | 121 | 0.842975 | 0.157025 | 99 | 0.0707071 | 0.929293 | TTTTATGTAAAGTTGAAATATTTTTTACAAATTCTAAATAGTGGAAGACCATTGTATATTTTGTAAAATTTCAGTTACGGAAATTGTACAGTGTAAGAATT[C/T]CTTTCTGTTAGTGTTATTTTTTTTTTGATAAACAAATGCTCACAGAATCTCAAAAAATAGGTCTACTAAGAAGTCCCATGGTACCTGGAACGTATTCCGTC |
| comp100092_c3_seq3:268-269 | comp100092 | 268 | A | G | 137 | 0.846715 | 0.153285 | 107 | 0.0747664 | 0.925234 | GAAGATGCTATTGTCAGAATGAGTGACTGTAGGGTGAATCACAAAAAAAGCAATCCCATTTCACTGGCCTATAAAGAGTACTCAAAAGGTTTGTAAAGGTG[A/G]AATTTGGAGAAGGACAAGGACAGAAGGAAGAGGGAATGTTGGAGAGAGGTAGTGCTGCAGCTAGAAGGACAATATGGAGGAGGGGGCAGAAAATAAAGAAG |
| comp105586_c0_seq5:1754-1755 | comp105586 | 1754 | T | C | 58 | 0.810345 | 0.189655 | 52 | 0.0384615 | 0.961538 | AGCCAGGGACCTTGGCTTGTGTCTCGGGCCACTGGGGTCGCTGACAGGCTTCTTAACTGGGGAATTATGCTTTGCACGGGATCACATAGCTTCAGGTTCCG[T/C]GAAGTACCCTTCAGAACACTTGTTAGTACACGTATTCTATTCATTTTGTTCTGTTGGGACAGTTTTTCTTTAAATCGAAAATGCACAGCCTAGCAGATAGC |
| comp92861_c0_seq1:1829-1830 | comp92861 | 1829 | C | T | 280 | 0.771429 | 0.228571 | 1 | 0 | 0 | TAGTCTCTGTGATTCAGAAGATTTACTTTATTGTTCTATTTTCCTTGTTTTCTAAAAAAAAGAAAAAAAAAACAGGAATACTCTGCCCCAATAGCAGTAGA[C/T]ACAGGTAAACGTTATTGCCATCTTTTAGTTATAGTCACGAAGTTCAAAACTTTTATTGCGAACGCTCCCTGGATAGACGTTAGTGGGAGCACTCATTCCCT |
| comp102534_c0_seq2:777-778 | comp102534 | 777 | T | C | 178 | 0.0786517 | 0.921348 | 300 | 0.85 | 0.15 | GCCAGCGAAGTCTTCTTCACATTCGACGAGGAAGTCCAGATCGGCAAGGATCTTCACGTGGAGAAATTCCACATGGAGAACATCAAGGAAAACATGTTCTC[T/C]TATTGCAACACGAAGACGGCTACTGGTAAATCAATCAAGTGCTGAAATGAACACAACTTTGAGTTCTTTATCCATTTTCAGCTGTACAGTTTAATTCTTGT |
| comp101378_c0_seq106:1215-1216 | comp101378 | 1215 | A | G | 1408 | 0.877131 | 0.122869 | 736 | 0.105978 | 0.894022 | TTGCCGAGGATGCTGAAGATGGTGAATCCGGCGAGCAGGGAGGTCAGCGTGTCGGCCAAGCTGATGATCCAGGCATCTCTGTAAATGTTTTGCGTGAAGCC[A/G]TTGTAAGAAGAGAACGTGATAATGGCGCCAAAACCAACCGAAAGACTGAAGAAGGACTGGGTGACGGCGGCGTACCACACATTGGGATCAGCCAGCATGTG |
| comp101811_c0_seq3:668-669 | comp101811 | 668 | C | G | 62 | 0.870968 | 0.129032 | 10 | 0.1 | 0.9 | TAATATCGTCCCATTTCACATGAAAATGTTGGTGTTTTAAACTCGACCATACGAAAATCATAGTGAAGAAGAGCAATCGCATGTCACGACAATGAACAACA[C/G]TTATATCCTAACCACGAGGTTGATCAAACACAAGGTCCAGGCTCGAAGGACAGCGATGGCCGGGGAAAGTTCTTCGGCAAAAGGGGAAACGGAGGAGAGGA |
| comp99316_c0_seq2:346-347 | comp99316 | 346 | T | C | 61 | 0.770492 | 0.229508 | 2 | 0 | 0 | GGTGGTGGATACGGCGGTGGCAGTGGCTGGTAAAACGTTATTCTCGTAAAGGAGTTTGCTTCCAAAGGCGGTACTAAATACATGAATACCATATTGGTATT[T/C]AGATTGTTGAATAAGAATAAAAGAAATCTCTACACGGTTTACTTTCAGATCCTAGTAAAAGATAGTCGTCTCTAGAATACGTCAATGATAGTAACATAAAT |
| comp105759_c4_seq2:261-262 | comp105759 | 261 | T | C | 60 | 0.85 | 0.15 | 25 | 0.08 | 0.92 | AGAGACCACCTGTCAGCCATGGTACTAAGCGCCACACTGACGTTCCTCCGGCGAGTGTACCAGTTAGCGGTGCGGGCGGTCTTGTGGGCGCTGTGGTCGTA[T/C]AAGTCGCCCGCCCGCCCGCTGCGCCCAGTCACTGACCCGCTGTTGGTGCAGCCCGCCCACGTGCTCGCGGAGTGGATTCGGGGCGGGAAGGTGACCTCCTA |
| comp103403_c1_seq1:378-379 | comp103403 | 378 | T | C | 136 | 0.779412 | 0.220588 | 102 | 0.00980392 | 0.990196 | GCTTTGGCCGCCGTCGACGCAGCGGCCACCGGAACGGCCGACTTTCCGATCATCTCGACCACGGCGGCGTTGTCCTTGTCTGCCTGATGAGCGGCCTTCCT[T/C]TCCTCGAAGAACTTGAAGAACTCCTCCTTGGCTTTCCTCACGTCCTCTGTGTCTTCGGGAAGGTTGTTGCTGATGACCCTGAACCCGCTGTCATCGGACAC |
| comp102820_c1_seq5:359-360 | comp102820 | 359 | C | T | 175 | 0.92 | 0.08 | 231 | 0.151515 | 0.848485 | CGCGACCACACTCACACACAATCACGTGATATCACCCAAGATTTGATGGTTCTTCTTCCGCGAGAGATACCAAACAGAAGAAGAATAAAGAAGTGAATAAA[C/T]AGAGAGATAAGAGTAGCTCCGACTCGACCAAAGAGGAGAAGGGGAAGCTAGAATTGACTGTGGTTCGCAAACGTGTCCGAGATAGAGGGAAGGAGAGGAGG |
| comp107564_c0_seq3:2061-2062 | comp107564 | 2061 | A | T | 81 | 0.876543 | 0.123457 | 37 | 0.108108 | 0.891892 | GTCAGATTAAAAATAAGTACGATAAGACCAGTGAAACAAATAACTGGCTGTGGCCACAAAAGAAAATGATAGAAATGGAAATGTTGTGATGAATATTCCTC[A/T]ACACACTGAAGGAAGTGGCAGGTGTAATAGGAAGTTGAGAAATTGTATAGCAAGGTGGACGAGAAAGAAACTTTAAGCAGGCAAGACCTAGGGAAGTGAAT |
| comp104253_c0_seq3:670-671 | comp104253 | 670 | A | G | 138 | 0.934783 | 0.0652174 | 24 | 0.166667 | 0.833333 | TACAGGAATTTACATCATCTTTTACACAATTACTGTCCGTCTGTGACAACAATTGGTCTGTACAGTTTGTCTACCTCACAAAAAAGGCAATTGGCAACATG[A/G]CAAAACAAGCTAATAGAGAGCAGTTAAAGGTAGTTATCAGGCACAGGGCATTGGTAAGCCTGACACGCATCGTTGTATTCTGGAACCTCTGCCTCGTCGCC |
| comp107646_c0_seq1:2550-2551 | comp107646 | 2550 | T | C | 278 | 0.928058 | 0.0719424 | 75 | 0.16 | 0.84 | TCTACCGTTACTTCATTCAGTTGCTGTCACGATCATTGTCATGGCATCTTCAATCATTAGTTATTAAAATTGTTTTTGAATCCATGTTTGCTATGCATTTC[T/C]GAGAACAATGAGGGCATGTGGTTGCCATAGTATTAGGTTGATTAAGAAGTCTTTCCCATAAACATGTAAGGTGTGTTTTGGTTTATGTAGGCGTTTGCCTG |
| comp105887_c2_seq1:2984-2985 | comp105887 | 2984 | T | A | 273 | 0.882784 | 0.117216 | 235 | 0.114894 | 0.885106 | TAGGACAGAATTTGTGTACATGTGTTCTTGTGATTTTTTTATTTAACATCATATAAACATTCATTTTTGACCAAATTATAAAGCATCTCATTGATCATACT[T/A]AAATGATGAGTTTCCAGCAGTTGTGTCCCCTAATTAAACGTGGTAAAACTACCATTGTAAGGAATTCCATTTATTGTACTATAATTTTTTTAAAGATAGTG |
| comp97823_c0_seq2:228-229 | comp97823 | 228 | G | A | 271 | 0.767528 | 0.232472 | 2 | 0 | 0 | CTTCTCTTGTTCTATCGGTGTGCAGCGTCAAGGTCAAAGTAAAATACAAGATGAGACCAGCGTTAATTCTTGCTCTGGCGGCCATCTTGGCGGTTTCGGTT[G/A]TCGATGCGAGGTTGAACATCTGTTCCTCGACTGCCGATTGCCCACCACGTTCTCAGTGCGTCCGGAAGAGGTGCTCTGACAAGTGCAGTTCTATGAGTGAC |
| comp107486_c0_seq1:458-459 | comp107486 | 458 | C | G | 102 | 0.892157 | 0.107843 | 16 | 0.125 | 0.875 | CAATGTTTGTTATTGCGTAGCATTGTATTGTATGAGAGTGTAATAGTATATATTTTATTTGATTACTGTTGATTTTTTTCTCTTTTTTGTATTTAATATAT[C/G]TTGATTAGGACAAGTACTTTTAAAGCTTTAAAAGATATTACTTAACTTTTAAAGGAGAAGAAATAATTTTTGTGTGTGACACTAGCCCAGTAAAAATATTA |
| comp102118_c0_seq2:180-181 | comp102118 | 180 | T | G | 136 | 0.816176 | 0.183824 | 119 | 0.0504202 | 0.94958 | AGATGCTGCGGTCAGTCTCACAGCTGTCTGCAGCATGCAGCAGAGCAGCCTTCACAAATGTGAGATTCGTGGCAACAAATAAGTGTAAATCATCTCTGCCC[T/G]GCATTAGATTAAATCCACCATTATTGTGTCAGGCAAACCAGCTTCACACAGTGCACTTGAGAAATGGATTGATTTCAAAGAAGATATCAAGTCTTTTTGCC |
| comp105700_c2_seq5:183-184 | comp105700 | 183 | G | T | 80 | 0.9375 | 0.0625 | 29 | 0.172414 | 0.827586 | CAGTGATGGTGAAGCAGTTGATTCATATTATGCTAACATTTTACATTTTCTTTTAATATGGGTGTGTGGAAAATAAGAATCATTGTCTGTCTAGAAAAAGA[G/T]AATTTTTTTTTATTATAATTACAATTATTTGGCATTGTCTTTCTCTCTTCTGTATTTTACTAACCTTAGAGCAAGCACAGAAGAATGTATTTTTGTACCAA |
| comp104096_c0_seq3:691-692 | comp104096 | 691 | C | T | 6 | 0.166667 | 0.833333 | 73 | 0.931507 | 0.0684932 | TTTTGTCGCTGTTTTTTGCCTGCTTGTGTCTCAAGCGCGTGAATTTGTGTCGCTGGAGATCGGTTGCTTCCAGCTCTGGTCCTTCCCTTTTTATTTCGTTA[C/T]TGTAAGTACAAGTTGACCTTTCTTGTTTCTCGATTCGTTTATTATTTTTTTGAGGAGTGTACACAGTTACACATGCAGTTATAGGTGTTTCTGTGTGTATG |
| comp90715_c0_seq1:103-104 | comp90715 | 103 | T | A | 51 | 0.882353 | 0.117647 | 34 | 0.117647 | 0.882353 | AGAGAGAGAGAGAGAGAAAGACTAAAAAAAAAGAGAAGAAATCTGCGATAGTATCATTTTTTTTTACACACACACTCGGAAAGACGTACCATAGGCTTCAT[T/A]TTCTGGGAGGGTAACGCTGTAGTAAATTTTTTTTTTATTATTCTCATACGCTTAATATTACACAAGTTTATTTAATTATAAATATATATATATAGCAAACG |
| comp101927_c1_seq3:3052-3053 | comp101927 | 3052 | G | A | 2 | 0 | 0 | 51 | 0.764706 | 0.235294 | AAGAACCGATTCGCGGGACCCCCATAACAGCGAGGAAGAATCCGCGAGAGAACATTCTAGATCGAGGTCGGTTCGGGGGGTCGATGGCTCCTCGCTGCGGA[G/A]GTGTAGCGAAGTCAAAGAGTTCTTCTATCTCTCAAAACACTGTCTCGTTATGTCGTCTTGTACAGGATTCCGGTCCAACCGCGTCAGTTAACAGGCTCCCA |
| comp106520_c1_seq1:2790-2791 | comp106520 | 2790 | G | A | 96 | 0.864583 | 0.135417 | 90 | 0.1 | 0.9 | ACCTTTGAATAAACCTGTTTTGTGTAGTGTATTTTCTGCTATTGTTGCTGTTATTAATTGTTTAACAATTATATTTTCTTTATTTATTGCAAGTGTTATGC[G/A]GTCCATAGCATAATTATGATATTGGTTAATGTAATGATTAATATCATGAAGATTCATTATTGATATTGTAATTGTTATCATATTTTTTCCAGATTTGTGAT |
| comp107387_c0_seq1:964-965 | comp107387 | 964 | T | C | 80 | 0.8375 | 0.1625 | 41 | 0.0731707 | 0.926829 | GATGTGAGCAGAGATAAAGGATATGGTCATTCTTTGACAGAAAGAACGCAATTCTATATGTTTAGGTATCAAGTAATTTGTGGAATGTGAGATATAAAGTG[T/C]AGTCATAAATGACTGCTACTTTTCATTATCTATAACACATAATATGAGGTATGAAAGACACTAAAGTGATTACTGCTAAACTATATTTTAAACTTATAAAT |
| comp100165_c0_seq1:818-819 | comp100165 | 818 | A | T | 122 | 0.795082 | 0.204918 | 64 | 0.03125 | 0.96875 | CGGATATTCGGTGTTTATGTAGTGTTGTGATTTTGTTATCTTAAAAGCTTGAATTTAGGACTTAAAGGGATAAGTAATTCATCTGTGAGTGGTGTTTTTTT[A/T]AGCTAGTTAAAATGGCAACTAGAAAGATTAACCTGCCTACAAAGGGACATTATTGGCCTACACTGGGCATAAATAATACATTAAACGGACAATACAGTGAA |
| comp106526_c2_seq1:284-285 | comp106526 | 284 | G | T | 84 | 0.869048 | 0.130952 | 19 | 0.105263 | 0.894737 | CATGCATAGGTGTTCATTGAATTATTGATTGTATTAGAGGTTAACTGACTGCCAGGCAGTTTAACAAGTGATTGTAATTAGCTTGTGTTATATGGATTATC[G/T]TAACTTGTGACTTTAATGTATGATTGATTTACTTACTGTGATTGAGAAATTTGCAGAAATATGCAACATATCTTGTTCCCAATCTCAGAATATTAAAAGCT |
| comp100545_c0_seq1:1406-1407 | comp100545 | 1406 | A | C | 526 | 0.89924 | 0.10076 | 44 | 0.136364 | 0.863636 | ATTGCTTTTCCTTTCTGCCTTTCCCTGACACCCCACAGCACCCGATCCATATCCTCCACTCCTTCTCTTGGCCTTTCAAGACACACACAAGACAAATAAAT[A/C]GAACAATCCCTCCAGCTTCACCCACAGCGTCACTCAGCACGCACAGATTCCCTCCCTACAGATCCGCCAGGTCCCCCAGCCGGTTTTTTCTCTCCTTGGCC |
| comp105311_c0_seq3:1548-1549 | comp105311 | 1548 | A | T | 59 | 0.762712 | 0.237288 | 2 | 0 | 0 | ACAGGCGCTGGTCATGGGCTGGGGTCAGACCTCGGAAGGGGATAAGGAGTCGAGTTCACCTGTGTTGCTCCAAGCCAAGGTGAAAGTGATCGGGATGAGCC[A/T]CTGCCGAGTCCTCTACGGCTTCGAGTCCGTGGACGTGACGGCCAAGATGCTCTGCGCCCTCGGGGAGAACGCGGACGGCTGCCAGGGTGACTCAGGGGGCC |
| comp91485_c0_seq1:1777-1778 | comp91485 | 1777 | C | T | 545 | 0.788991 | 0.211009 | 152 | 0.0263158 | 0.973684 | CACGTGCCATGTGATTTTTTCCTGCGTTCCACTTTCTACTGTGAATGAGTGTACTAGCGAGTTTAATTCGTCCTAGTGTTACACCAGGTATTTTATCTTTT[C/T]TTTTTTTATTAGCTGATGGACCCATAAAAAGGTTTGCGAAAAGATAAAGACAATTCGAAGTATCAAAAAGTAACCATTTTGCTGTACATAAGAATTGCAAA |
| comp107083_c0_seq2:1260-1261 | comp107083 | 1260 | G | A | 100 | 0.94 | 0.06 | 169 | 0.177515 | 0.822485 | CTGAGAGCATCCAACCTTGACACCTGCGTAGTGGCCCTCCTAATGGCACTGTCAGGCCTTGTGTTAGCACTGAAACCTCTCTTTTTCATGCCATTTCGCCC[G/A]AGTCCCGTGGATGCCCTAGGAACCCTCGCGGCGAAGGACAGAAGGAGCGAAGATGTCACGAGCGTCATAGCAGGAAGGAGAGTGACTGTGCGTGGCTCTGG |
| comp105264_c4_seq21:1467-1468 | comp105264 | 1467 | G | A | 264 | 0.867424 | 0.132576 | 750 | 0.105333 | 0.894667 | CATACGGTGGAAATCAGCTGGAAGGGCAAGTCCGGAGACTGCAGGTTGCCTTCCTGCTGTTCCTCTGCTGCCGCCTCCGGGGTCGCTGGGGGCGCGGACCG[G/A]CGGGAAATGCGGTCCAGGGACTGCGCCCCCCTGATGGCAGCCAGGGGGGGCGTCGGGATGTCGTCAGGCCCCAGCTTCTCGCGGGAGGCGTGTGATGGAAG |
| comp103253_c2_seq3:1165-1166 | comp103253 | 1165 | A | T | 74 | 0.905405 | 0.0945946 | 97 | 0.14433 | 0.85567 | ATTCGTAACGACAGTGCTTCCTGTTAGACAGCTCATAACGGGGTATTTTTCCTGTCTGTTCATAACAGGGGTGATCTCTGTTAGTCAGCCGATCCTGAACC[A/T]TTGTGTGTGAAGGGACTCTACTGAAGTTATTCAAGCTCAGAAACTGGAACATATATATATAAGTTATCTGATGAAAAACATTCAACGAAATCGCTAAGCAG |
| comp91358_c0_seq1:191-192 | comp91358 | 191 | C | A | 100 | 0.76 | 0.24 | 1 | 0 | 0 | TCCATTTCGATTTGCGCGGATCGATTATTAGAATCAGAATGCATTACTGTCTGCATCAATGGAATCCTGTCGTGAGATCCTCTAATACCGTCAGCAGTGGA[C/A]TCTGGCTGACAGTGCATAACATTAAGAATTCTTTGCCTATTTCGTACTGTTGCATTGTACAGGGTTTACGGACTGCACTGTCCATCGGGTATGGGCATGTG |
| comp100397_c0_seq1:955-956 | comp100397 | 955 | T | G | 18 | 0.166667 | 0.833333 | 81 | 0.925926 | 0.0740741 | CAAAGTGAAAGGAAGCAGACATTTGTTGTAGCCTGACTGGTCTGTTTTGAATAGGTTTATGAGGAACAATTTTTTTTTTTTTTCTTGATTTTTTCTATCCT[T/G]TTTAGATTAACAGTTATCTTTCTTCTTTTTATGAGATATGTGAAATTAATGGAGTTTATCATTAAAAAAATAGGGCATGTCTTTTCATTAGGAATATATAA |
| comp102746_c0_seq2:451-452 | comp102746 | 451 | G | A | 54 | 0.759259 | 0.240741 | 2 | 0 | 0 | TCCGGCTACGTGGCTGAGGTCAACTACGAGGGCGAGGCTCGTTACCCCGACTCCTACGAGTCCAACGAATCCAAGTAGAATCTCCAACCTTTTGTGATACA[G/A]CGACTGCGATGGACTCTGCCGGTTACAAAATGAATCTAGTTATAGCTGTTATCTATTTATTAATAAAGGCATAAAATAACGCTAGGCCTAGTAATTTTACC |
| comp98057_c0_seq2:153-154 | comp98057 | 153 | T | A | 141 | 0.758865 | 0.241135 | 2 | 0 | 0 | TTTTAACATATTTAACGTTTCAGTTAATATATTCAGAATGCAACAATCAGTTTATAATAACAATTTTATAGTTGAATGATTTAACATTCGATATTTTTATC[T/A]GACTCAAATAATCATGAGACAGTCGGGCACACAGTGACACACGGGGCATCCTCCTCGCACTGTGATGTCGCCGCGTTGAACACCTGGTTATAAGGACAACG |
| comp98389_c0_seq2:202-203 | comp98389 | 202 | G | C | 98 | 0.887755 | 0.112245 | 62 | 0.129032 | 0.870968 | TCTTTATAGATAGACATGCTTGTGTCATTTTACGGAAGCCTGATCATGTCCAACCATGTCCTCTCACAGATAGCTATAGCTTTCGTGGCGGCGCTGAGTGT[G/C]GCCGCAGCTGCGCCCGGAGGTTGCTGTGGAGGCGGCCATGGCTCCTACGGCAGCAGGGGAGGAGGAGGCCATGTAGGGGGAGGCGGTGGCCGTGGCGGCGG |
| comp102423_c0_seq2:420-421 | comp102423 | 420 | C | T | 321 | 0.819315 | 0.180685 | 33 | 0.0606061 | 0.939394 | AAAAAAACATGTTAATAAACTGTCCGTTCAGATCTATTTTGTTAATCGGTGGTTAATCACATGCTTTTTGTGTTAATTAACGGAAGTGCAAGAACCGGTCG[C/T]TTTAGAGCATCAATGTGCATAGCTAGAAGGGAGTCTGGGAAAGCTGTGACCGCTGTGTAGGTGCCGAAAAGAGCGTGGACGAGAGCGAGAACTATTAGCGA |
| comp104816_c0_seq5:558-559 | comp104816 | 558 | T | A | 145 | 0.841379 | 0.158621 | 133 | 0.0827068 | 0.917293 | TCTTTTTTGATATCTTGGATGACATTCAACAACAATGAGGAGCTGTGCAGAAGAACAAAGAAATAAAAAAAAAATAAAAGAAAATGCTGCATCGTCCTTCA[T/A]TTTTGATACAGTATTCCTCTCTGTCTTTACCTGATATCTCCTTTTACGAAAGTCTAAGTGAATTCAGACATAACTATGACTCCTGTTGATCAACTGGAAAA |
| comp99849_c1_seq4:134-135 | comp99849 | 134 | T | C | 236 | 0.758475 | 0.241525 | 1 | 0 | 0 | GTACTCCCAGCCCGCCCCTGCCCCCGCCCCCACACAGTTCTACTCTCAACCTGGAAAATGATTTCCTTCTTCGAGTGTCGGGATCTGCTTCTCTTTTCCTT[T/C]AGTCATCGCACACCTTTCATGATCTGAACTTCGATATTCACCATTCATTATCTTCTACAACCTATTTTGTTTCCTTTTCTGTAATGTTTACACGTTTCTTT |
| comp107639_c0_seq1:238-239 | comp107639 | 238 | A | G | 92 | 0.771739 | 0.228261 | 71 | 0.0140845 | 0.985915 | GTTGATGTTGAACTCCTGCATGATCTTTTGCTTCAAGAACTCCATCTTCGCATTTTCTCCATGCACCAACATTACATTTTTGGGCTCACAGTGGCGAATCA[A/G]CTGCATGATACCTTTGGCATCAGCATGGGCACTAAAGGACATGTACTCAACAGTAAGGTTCACCTCAACACTTGTTCTATTCTCAAAATCAATCTTCTTAG |
| comp100283_c3_seq1:1698-1699 | comp100283 | 1698 | G | A | 63 | 0.857143 | 0.142857 | 40 | 0.1 | 0.9 | TTCTATTTATGGCACAATCAAGCTCTTCCATTTTCTCTTTGAGGCAGAGAATTTCCTATAATGTTTAAATGACTTACATAAATTGATACAACCCAAGTTCT[G/A]CAACATAAATAATTTGGAAAAGCAATTAATCTTGTATCATTTTATAAATGTACCAATAAAATTGTAAAAAGAAATGTCATCAATCCTGATTGAACCAATAA |
| comp92476_c0_seq3:572-573 | comp92476 | 572 | T | A | 90 | 0.9 | 0.1 | 35 | 0.142857 | 0.857143 | GTGTTGTTTTCTGAAGATAGCGATCATAGAAAAATTGTTTTATCAGTCAGAGGAGATATAGTGCAAAGGAGGTGGTGATGTTTTGAAGTGATATGTGCTTC[T/A]TACGGATACCATACCCTAGTGTGATTTCACCCTGTAGTTTCATGCGCATTGTGGTCCTTATCAATGAGAATAACTTACCAAACATTGTCTCAAATTCAGTT |
| comp107839_c0_seq1:7795-7796 | comp107839 | 7795 | C | T | 452 | 0.814159 | 0.185841 | 383 | 0.0574413 | 0.942559 | TACAAAGAGTCCTTCATCGAACTTCACGACCAGCATGAGGCCACCAAGAAGGAGAAGGACCAAAGCAACGCACAGCTGAAGGAACTCCAGTACACGCACGA[C/T]ACGCTGAAGTCTGCCTACAACTCCATGTTTGCTGAGTACAATGAACTGCGGGATGTGTGCGACAGCCATAAGAGTGACGTGGGCATCCTGAAGGGGGAGAG |
| comp99175_c0_seq1:478-479 | comp99175 | 478 | T | C | 64 | 0.828125 | 0.171875 | 70 | 0.0714286 | 0.928571 | ACAGCACTTGGTAACAGTTTCCTGACTACATTGTGAAAATACTGAATATTAATTAATGGTAAAACAAAAGAAAGGAGTTCTGCAAATCCATGCCACAATAA[T/C]TCTCTTGTGAAATACACGTACGATATTTTCCGTACGTTTCTAATATTTGATGACACTGGTTTCAATCTGAGCATTCTTTCAACAACTGATGGGTATATTCC |
| comp105825_c0_seq1:922-923 | comp105825 | 922 | G | T | 1119 | 0.785523 | 0.214477 | 582 | 0.0292096 | 0.97079 | ATTCCCACGCTGTTTAAGGCGCGGGAGAAGTTGGCGCAGAGAGAGAAGTTCTAGAGGAAATTCGCTGGCCTTTCAGAAGAATTTTTGAAAAGAGAAAGGTG[G/T]CTTCAGAGGAAAGTTCAGATTTTTTTTGTGTGATTTTTTTTTTTTCTGGAGAATTTAGATAGATAGATTCAACTGAAGTATATCAGTAAAAGGAAGTTTAA |
| comp107359_c0_seq1:2237-2238 | comp107359 | 2237 | A | G | 101 | 0.881188 | 0.118812 | 56 | 0.125 | 0.875 | GAAGCTGTTACATAAACTACTAATCCATTGTTACACAAGGGCCATTTACATTTTCAAAATACGCTTTTTGCATTCCATCCCATGCTTTGGCCTAGTGTAGA[A/G]GAAATATCTTTAATCCTTTGTCTACATTTGTCTGTTCTTCCTAAGTACCTAATCAAGGCAATTCTATTCAATAAATTTGATTACTCAGTTGCACATGTGGG |
| comp106511_c1_seq10:376-377 | comp106511 | 376 | C | T | 159 | 0.169811 | 0.830189 | 675 | 0.925926 | 0.0740741 | CCAGCTCCGCTCTTGTTCTCAGATCTCCAAAAGTAGTTCTGTTCTCTTTAAAAAAAAACCGTTGAGAAATCACGTGACTGCGCGGGAAAGGCAAAACGCAT[C/T]CGAGGATCAGACTGTTGTTTTCTTTCTGACATGGTATGTAAATCAGATTTTCTAGAAGATGCTTGAGCTTTTGCCATCCAAATCTACGATTTTGAGATTAA |
| comp96514_c0_seq11:167-168 | comp96514 | 167 | T | C | 2 | 0 | 0 | 2025 | 0.756049 | 0.243951 | GCTTGAGGAGTCATCGCTGACGGCCCAGTTGAAGCTGTACTTGGCTTCGGAGGACTCGTAGGACTCCTCAGAGGACCGCTAGAACCCTGGTGGTCGGTATT[T/C]GTAGGATTCAAAGCTGCCTGCAGCTGCAAAGGCTGCAAGACCCAGGAGGATGAAGACCTTCATGTTCATATGAAGTGTTGTAGTTGTTGTAGAACTGATTC |
| comp103676_c1_seq75:1475-1476 | comp103676 | 1475 | A | G | 72 | 0.888889 | 0.111111 | 90 | 0.133333 | 0.866667 | ACCGTGCCCGTCATGTCCTTGTACGTCTCGTATTTTTCGTTTTCCTCAATCTTCTGGTCTCCCCTGAACCAGTGGACGATGGCCTTGTGGGAGTTGACGAC[A/G]GCCTCCAGAACGGCGTCCTTGCCCGTGTAGCTCGAGGACTTCTTCGGCAGAGGCTTCGTGAACTTGAAGTCGGGGTCGGGTTCCTGCACGGTGAGGATGGC |
| comp107925_c1_seq14:530-531 | comp107925 | 530 | T | A | 97 | 0.113402 | 0.886598 | 99 | 0.868687 | 0.131313 | TGTATAGTTTGTCTTGTGTCTGTCTGGCTGTCTTGTGTCCTACAGTTTTGGGGTTTTAATATCATCTAAGTTATTCTCTCCAGACGTGACAGCTACTTAGT[T/A]CAAAGTCTTATATGTCATTCAGTCTGTCTATTGTATGTCAGTCATCAATATCTGTCATAATTGAACACTTAAATATAGTAGTCTGCATTAGTCTTTATTTC |
| comp106314_c0_seq1:1241-1242 | comp106314 | 1241 | T | G | 21 | 0.190476 | 0.809524 | 54 | 0.944444 | 0.0555556 | CTTTTCTTCTATTACGTGTAGCATAGAAAGCCTAATTCATATATATTTTATTTTTCTTTACAAATTAGTTTAAGTTTGCTTTTTATCCTTTTTGTTGCCCT[T/G]TATTTAAGTTTTAAGTTTTTACTTCAATGCTTTTGCTTTATTCCAACAAAAGGCAAACATCAAAATAACCTGAAATACAGTTCTTGCATTTACTACATGCA |
| comp91707_c0_seq1:218-219 | comp91707 | 218 | A | C | 1 | 0 | 0 | 65 | 0.753846 | 0.246154 | CGGGCGTCGCATAATTTTAACAAAATGGGTGTAAACCAGGAATGATCTTTTGTAAACCATCATTTATTGAAGTTTGCACGCGGGTGTGTGGTGGTCTCTTT[A/C]CAATTATGGGTCATAGGCAGAGGTTAATGAGGCCACGCTAACCTATGTGAACTGTTAGTGGATAAAATGTTTATTGAGTATTATATATCATTTGTGAATTG |
| comp99075_c5_seq2:117-118 | comp99075 | 117 | C | T | 101 | 0.772277 | 0.227723 | 54 | 0.0185185 | 0.981481 | TTAAACTGGTTAGTCATACTACATCATGTGTGCAAGCATCACTTATTATCATTAGTATTACATTCATGTGAGTAAAGGGTTTGAAAAGCACAAGACAGTTG[C/T]TCAGACTTTACAGATTGAAAATTATATTATTAACAATGTGATGACATTCAAAACGAGCAGATGACTTAAAGTGTTGGGAAAACCCCACAAGATGCTTTGAA |
| comp104255_c1_seq1:528-529 | comp104255 | 528 | A | G | 42 | 0.833333 | 0.166667 | 100 | 0.08 | 0.92 | GAGAACCAACAGCTCAAGGAGGAAAAACAGATGCTGAGGACGGAGAACGTGCAGCTCAAGGCCGAGGTCCTCCAGACGGAAGAGGAGATGAGGAAAGTGAA[A/G]GAGGAAGTGAGAAAAGTCGAGGAGGACAAGCGCCAGTCCGACGAAGACAGACGAAGAGCGGAAGAAGACAAACGCAAGATGGAAGACGAGAGGCGAAAGGT |
| comp105990_c1_seq2:1121-1122 | comp105990 | 1121 | C | T | 59 | 0.864407 | 0.135593 | 9 | 0.111111 | 0.888889 | AAGGCAGCCAGCATTTCACCTGGGGGAGGAAATACCCAACCAGAAAACTAGCATTAGAAAGGGGAAACTTCGTATCAGTCTCTCTATGAGCTGTACATGTC[C/T]TGTATATTTTCCTATTCTCTCACAATTTGTGGAATTTTAGCTAAGAACTTCATGAGAACTTTAGAAAACTTTAATTAACAAAGCTAAACTACAAAATTCTG |
| comp102689_c0_seq4:129-130 | comp102689 | 129 | G | A | 109 | 0.12844 | 0.87156 | 59 | 0.881356 | 0.118644 | CTTCTCCGAGCCGCTGACTTGGAAGGCTTTACCCTCTAGAGTGCAAAAAGTACAAAAACCAGCAAAGACTAGTTGACTCTGGTGTCGAACTGGAGTCTACA[G/A]CAAAGGCAATGGTCTCCCGTATCCCACCTAAAGGGACATGCCCCGAAAGGGCCACATGTGGCCCTTTGACTGGAAGAAGTCGTCTTAAGCCTTGGGAAGTC |
| comp105342_c1_seq2:375-376 | comp105342 | 375 | G | A | 336 | 0.761905 | 0.238095 | 109 | 0.00917431 | 0.990826 | GAGAGGGAGAAGGAGTAGAGGAGGTTATATTTTACTCATATTACTGGTCCTTTTCTTTTTTGTTTTTGTTTTTTGTATTACTGGTATTTCTGTTTCGTCGA[G/A]GGAGGAGCGTAGAGGAGGGGGTTCAGGAGGTGGTCTTGAGGGTGGGGCAGAAGAAGGGTGAAGGAGAGGGAGGCAGAGCGGAAGGGAGAAGGAGAGAGAGG |
| comp104362_c0_seq3:318-319 | comp104362 | 318 | C | G | 11 | 0.181818 | 0.818182 | 76 | 0.934211 | 0.0657895 | GATTTTTTTCGTAAACAGGCTGATGGCAGTCTCTAGGTGTTAAGATAGGGTACGGTAGTATTGTTTTAGTATTGTTTTACTGTAAAATCACTTGCTTACAT[C/G]ATTATGAAATTGGATATTCTTTCATTACGATGTCAAATTGTTTTATAGAGCATTGTGTTATATTTCATGGTTCTAATGTTAAATTGTCTTATAGGGCATTT |
| comp105808_c0_seq2:1795-1796 | comp105808 | 1795 | T | C | 60 | 0.8 | 0.2 | 21 | 0.047619 | 0.952381 | GACGAGGAAGGAGGCTCGGCCATCCAGGTTCGCCTCCGCGAAGCCTTGGCCAAGCGCCTTGGGTCAAGCCAGAGCCAAAACACTCGTCAGGATCGTTCTCG[T/C]CTCGCCCAGGACCCTTACGTCGACGAGGAGAAGGATGAGTTTGACCTCAAACTCTCAGGATCGGATCTGCGTCCCTCCTTCGGGTTCCGAGGATCAACGCC |
| comp106021_c0_seq3:1250-1251 | comp106021 | 1250 | C | T | 6 | 0.166667 | 0.833333 | 74 | 0.918919 | 0.0810811 | AACTGCACCGACGTGATTGACCACGATGACTGCGTGAGGTACCTGAGTACTGAGTGCAGCGTCACCGATTACATTGCGAGGTTTTGCTGTCGTACCTGCAC[C/T]CTCGTGGAACACTTGCCTGCCCATGGACCACACCTTCTAAATACAGCCAAAGCACCATTGTCCGCTGATCTCCGACTTAGTGGTAGCGATTCGGTCTGGAA |
| comp104640_c0_seq10:1149-1150 | comp104640 | 1149 | C | G | 588 | 0.823129 | 0.176871 | 421 | 0.0712589 | 0.928741 | GGCCTCTGGGACTACGAGGTCGTGGAGATGTTCTTCCTCAACGACAACGACGAGTACCTCGAGGTGGAGCTCGGGCCTTGGGGGCAGCACCTCCTCCTCCT[C/G]CTCAAGGGCGCCCGCAACGCCATCAAACACTCGATGCCCCTGGACTACTTGGCCCAAGTCCCAGCGTCGACGGACGGCAAGTGGAAGGGCTCGGCCTTGAT |
| comp106895_c1_seq1:2958-2959 | comp106895 | 2958 | T | G | 58 | 0.12069 | 0.87931 | 86 | 0.872093 | 0.127907 | AAATCTATGAAAGAGTGTGTTCTATCCTGGAAAGAGGCACAGAGACAGACGGCGATGGTGTCCGAGTTGCCAAAAAAGAGATCCGTCATCTCTACAATCCC[T/G]CTTTACGTATATCAAACACATCGCAGTCAGTCGAAGGCATCATCAGATACCTCAAGGAGCTTCGTAGTCAAGGAGGGGCATCGAATACTTTTAACTAGTAT |
| comp105870_c0_seq11:157-158 | comp105870 | 157 | T | G | 1950 | 0.917436 | 0.0825641 | 18 | 0.166667 | 0.833333 | TATTTCTTTTTTTATTTTTGTAAAGAGATAGTAGAGAATAAAAGGAAATATAAAAATGAGTAAAAACAAATAAACAAAACAAAACAATACAAAAAAAAAAC[T/G]AATTTTGGACAGTCAAAATCTGGAATCTGACGCTTTACATTCCCACAGATTTTCGGGAATGGTTCAGATGTGTCATACAGTGTGGTTCGGTGTGGTTCAGT |
| comp101164_c0_seq1:178-179 | comp101164 | 178 | A | T | 52 | 0.923077 | 0.0769231 | 29 | 0.172414 | 0.827586 | CTACTCAATCTGACTGAACTTATGTTGTCTTAAGGGCCAAAGTCAATCATTAAATATACATGCCTGAAAAACAACTACACATATATATGATTTTACAGAAA[A/T]TGAATAGTGTTTCCTTCAATCTAAATGTTTCATCGTTGTTCACTTAGTCACTGAGGACCATCAGTTATCAGCTGATGATCTTCAAGTTCCGCTACAGGATG |
| comp102618_c0_seq8:1219-1220 | comp102618 | 1219 | T | C | 67 | 0.940298 | 0.0597015 | 58 | 0.189655 | 0.810345 | TTGGCGCCGCACCTGCAGAAGCCCTGGATGCACGCCGAGTGGCCGACCCGCATGCTGCACTGCGCGTCCACTTGGCACCGGAAGCCGAGGAGCACGCCTGG[T/C]AAACACGTAGTCTTATCCAGCTGAAGATAGTGCTGAGGACAGCTGCAACGTCCCTCCGAGCACACACTCTTCGCAACAACACAGTGCTTGTTGATGACGCA |
| comp103269_c1_seq3:633-634 | comp103269 | 633 | T | C | 23 | 0.173913 | 0.826087 | 197 | 0.923858 | 0.0761421 | TTCTCCCAGTGAAGTTGCCTTTACTACAATAGATGTTCAGTCAGCTGTACATTATTCAGATGTACAAATGCATTTCCAGTTGTAGAAATGGAGACAATTCG[T/C]AGTTCGTCCCCTTTTTTCCTACTGTAACAATTATAAATTTGGCTATCTCCTAAACAACTGTTGTAGTAAAGAGCAATAAGAAACAAAATTTAATACTTTTT |
| comp107148_c0_seq2:261-262 | comp107148 | 261 | A | G | 154 | 0.818182 | 0.181818 | 131 | 0.0687023 | 0.931298 | GGCGGATCACAGATAGAAGCAAAATTGGACAAACCGGCAGTAATCCACTACATTTGCTCAAAGAAGACGGAAGGATGGTTCGACCTGTGGCTGAATATGGA[A/G]CTTCTTGTTCCATATGTCATAGACTGTTGGGTGGACAATATGAAGCTGGTATATGACAATGTAACACGTACAACTACCAATGCACCAGGAGTTACAACAAG |
| comp106333_c2_seq15:761-762 | comp106333 | 761 | T | C | 100 | 0.81 | 0.19 | 66 | 0.0606061 | 0.939394 | ATCACCAGCACGTCGCTCTTGCGGAACTCGAAGTTATAGAGTTTGTCGGCGAAGTTACAATAATTCTTCGAGTAGACCCACCCGCCGGGAGAGAGCCTAAC[T/C]AAGCCATTCTTGAAACCGGTAAAGTTTTCGATTAGTTTTGCCCACTCGTTTCCCTCGAGGACAGTTGCCTTGTGACCGCTGGCTAGGGTTGTCATCTTATT |
| comp104580_c0_seq1:145-146 | comp104580 | 145 | A | C | 115 | 0.0434783 | 0.956522 | 125 | 0.792 | 0.208 | TGACAAATATTGGATATGTTAACATCAATATTCTGGATTTGTGTTCCACTTTGATGTGTAATATAATTTCATGCTTGTTGATAATTTTGTAAATAGTTCAG[A/C]CTATGTTTTTTTCTTTCTGTGGCTTTCCGGCCGGAGAATGGGTCACAGATTGACCCAACCTTTTATTTTGTGTGACCTCAATTGGACGGTAATGGTAGATT |
| comp99698_c0_seq3:367-368 | comp99698 | 367 | T | C | 375 | 0.816 | 0.184 | 44 | 0.0681818 | 0.931818 | GTTGGAGGACGAGGCGGAGTTGGACGAGGACGAGCTGGACTGGAAGGTGAAGGTCTGTCCCGGCGCGACGTGGGGCAGCTTGTTCTGGAGATCCTGGTAGG[T/C]GGGGGCGGCGGAGCACATCCCGGCCAGAGCGAGCATCACGATTCCCACACGCATTTTGAATGTCCTCGTTTGCTCCTGAAAGCTGACTATGTAGAGCAGAA |
| comp104621_c1_seq3:227-228 | comp104621 | 227 | C | G | 55 | 0.872727 | 0.127273 | 32 | 0.125 | 0.875 | AACACCTGCCTGAATACTCCCTCCCTCCCTCAGGATGGGCTGCCTGGACTGCTGCACCTCCGTCTGGGAGAAGGCCAAAGACATCTTCAAGAAGTGCGTGG[C/G]TTCCTCCGGCGACATCTCGTGCAACGGCCTCGTGCTCAAGCTCCACTACCGGTGCTCATGTCTAGTCATGTTTATTGCGTTCATCGCCGTTTGCAGGTCAT |
| comp107171_c0_seq4:412-413 | comp107171 | 412 | A | G | 129 | 0.899225 | 0.100775 | 66 | 0.151515 | 0.848485 | AAGAGAAATACACGTATATATATTCACACCTATATATATTTTCCCTTACGATGCTGACGTGTCACCACAAGCGTCACCATAGGCCCTACGAAACTTAAAAT[A/G]TCTTCTCCAGCTCTCTCCTGACGAAGACCTGACGAAGGCGCTGAAGACCTACTCCTGCTCCTTCGCTTCAGGACTACCGATGACATCCTGCAGGACCTCCG |
| comp102145_c4_seq1:1032-1033 | comp102145 | 1032 | A | G | 1 | 0 | 0 | 75 | 0.746667 | 0.253333 | GAAAAGAACATTTTAAGTTGGTGTTATACTCGGCAATGTCATGCAGAATTCTCAGCGGCGCACCATAAGTTTGCAAGATGTTACTTGCTCTTTGTTGCAAG[A/G]AATACAGTGACCACACTGTTGCAGATTTCGAAAATTGAAGGAAAAATAAAAGTTATGGTGTGTGGTGTTGCAAACTAACTGTGGCATGTCTGAGGTAGTGT |
| comp103896_c0_seq13:598-599 | comp103896 | 598 | T | G | 68 | 0.808824 | 0.191176 | 16 | 0.0625 | 0.9375 | AATGTACTATGAAGGCCCAAATCAAAAGCAGTTTTAATTTGAGCGGAACAAATTAGCAGTCATACATGATTTTGTTAAAACTGCAGTATTGGGAATTTTTT[T/G]GATCTTATTTAGTTATCGTTTCCCGTTAACTTTTTAGGGGTTAGTTGGAATACTGTTAAATATTATGCTGTCTTTTTACTTCCTCCTCCTCCTCTATCTCT |
| comp106739_c2_seq2:549-550 | comp106739 | 549 | C | A | 462 | 0.919913 | 0.0800866 | 380 | 0.173684 | 0.826316 | TCCTTGCAGGCGTACTCGCTCTCGTCCACGTCGTTGTAGCAGTCGCTCTTGTAGTCCTCCGGGAGCCCCTTCCCATTGAACTCGCACAGCTTGGCGAGCGG[C/A]CGCGACCTCACGAACATCTTCTCGAAGGAGGTCGGCTTGTCGAGCGGGCAGTAGAACACGTCGGGCGTCGCCTTGTCGAACACGATCGACTTGCGCATCAT |
| comp107389_c0_seq10:226-227 | comp107389 | 226 | T | C | 239 | 0.753138 | 0.246862 | 289 | 0.00692042 | 0.99308 | GGGAAGATTACAAATGGTCAAATAGAGTCCAGATATACAAAGGGGAGTCTTTTCTCTAAAGCACCAAATCAACCTGGGTTAGAGATCTTAAGTAGAGAAAT[T/C]AATTCTGGTCAAGATTATTTTCTTAATATTAAGGCACTTAAAACAGAATTTTGGGCATTATGTGTTCAATAATCATTTAGGTCCAGTTTGGAATGTTTTCC |
| comp102346_c0_seq2:224-225 | comp102346 | 224 | T | C | 88 | 0.886364 | 0.113636 | 64 | 0.140625 | 0.859375 | CATACACTTCAAGGAAATAATTTCCTCAAAACACACTCCTGTGCTATTATATGGGGGCACGAGTTTGGAAAAAAGTGCATGCATTGAATATGTCAGGATAG[T/C]TTTTATGCAGTGATTTTCATTAAAAATTGTGTTAATTACACATTAAGTACATCAGTTTTTCCTAAATGAAAGCCAGGGTTTGAGTTATTGAAAAAAAATCA |
| comp94820_c0_seq2:856-857 | comp94820 | 856 | A | G | 52 | 0.884615 | 0.115385 | 36 | 0.138889 | 0.861111 | CCTCTCCCGCGTCCGTTTCCTCGGCCTTTGCCATTAGTTTGAAGGTCGATTCCTAGGTCATCCGCGCATTCGAAGCCCGTTTCGCCGACTTCCCTCTCCAG[A/G]GACACGGCCACATCGCGACATTCTCGGACGGTGCTGTCAGCCGGATGTAGGAGCATGGACGCCACGAAGACCAGCAGGAGTAGGAACAGTTCCTTCATGGC |
| comp107522_c1_seq4:362-363 | comp107522 | 362 | G | C | 1299 | 0.759815 | 0.240185 | 561 | 0.0142602 | 0.98574 | CCCCTGTCGCCCGCTGTACAGAGGTAGATGTCCGTTATAACTGAACCGTAGACGTCAATGCACTGCTGGGTCGAGATGGTCTTTAGGTCCAGGTACTGCAG[G/C]GCGTGGTGGTAACCGTGGGTGTCAGCCCCCCATCCAGTGGCAGTCACGTCCATGTCAAAGAAATTAGCGGAATCAGCAGCATCCGAGTAGCTTGGTAGACA |
| comp100274_c0_seq1:590-591 | comp100274 | 590 | T | C | 110 | 0.745455 | 0.254545 | 1 | 0 | 0 | GCGGCTTCGGCTTCATGCGGCCCATCGTGTCCCGCCCCACCCGCCGGAAGGTCCAGCGCCCCAGCAACTTCTGGCGCGATCGCTTGGAGGCGCGGAGGACG[T/C]ACGCCCAGCACGCCCCGTCCTACAGGAGAACTCTACGGCGAAGAAATGGAACGCCATTGCATGACACACACAGTTTTGTCATGCGCGGCGATTCCGTGAGG |
| comp101034_c4_seq1:361-362 | comp101034 | 361 | T | A | 59 | 0.881356 | 0.118644 | 66 | 0.136364 | 0.863636 | ATATTTGAGCGATATCATGTCCCTTGTTGGATGTTAAAATATAGTTTTTGTGCGGTCAGTATATCTGGCACATAAAGACAAAATTTATGATAGTGGTTGAT[T/A]GTGGCTAGGTTTTTTATGATTGTTATTATTATTGTTATTTTTATTATTATTTTTGGTGTTAAATTTGGCCACTGTGCTCCCAGCCCCTGATTAAGGTACAA |
| comp91263_c0_seq2:113-114 | comp91263 | 113 | C | A | 84 | 0.797619 | 0.202381 | 19 | 0.0526316 | 0.947368 | AAGAGGACACAGATAAACATAAAAAGGAATTAATCAAAAAAGATATCTATGTATCCAGCCCCCCTCCCTCTCCCTTCCTCGTCCCTCGCCAAAAGAGGAAA[C/A]AGATATCTATGTATCCACCAGTTCCGAAGCTTCCCTTTGACGCTTCATCGCCCGCAGTCGGTGCTTCGTAGAACATCACACTTGACTCGCTAATAAATCCT |
| comp102817_c0_seq1:1299-1300 | comp102817 | 1299 | T | C | 67 | 0.865672 | 0.134328 | 58 | 0.12069 | 0.87931 | AATAAAAATCTTCATGATCACAATTGATTACAACAGCCAGAAGTACTGCTGTTTCATGATATGATGATGCAGCATGGTCTCATTTCTAAGTTTCAACTAAA[T/C]ATTAAAATTGAGAGATGCAGACCTTCAATTTGCAAGGCAGAGGATCACTAAAAATGCAATCAATGCCGATGACACTTGCAAACATGGAGAAGAAGCAGTAC |
| comp98660_c1_seq1:194-195 | comp98660 | 194 | A | C | 69 | 0.811594 | 0.188406 | 30 | 0.0666667 | 0.933333 | AAGACATGGATGACAGAATGGGTAAATGTGATAATAAAAAATGAAATATGAAGTGTAAGGTTTAGTACAGAAGCAGAGTAATGTAGAAATGGGAAAAGACA[A/C]GGCTAACGGAAAGTATGGATCACTTAATGGGTAGAACAAGAAGAAGAAGGAAAAGGAGAAGAAGAAGGGGTAGAGAAGGAAGAAGGAAGAAGGAGGAGAGC |
| comp105885_c0_seq2:181-182 | comp105885 | 181 | T | A | 306 | 0.866013 | 0.133987 | 140 | 0.121429 | 0.878571 | ACTCATGTATTTAGTTTTTAATTCATATTTTCAGGTATTTACTTCGCATATGTACTTAATATGCGCGCACCGAAAATACACGTATATACTAAACCTTGTTG[T/A]GTAAGCATTATTTTCATAAGCTGGCTTTGTAATTGAAAATAGGATAGGCTTCCTCGGTAGGCTTTCCTCCTATCCATTCCTCGCCCCTTCCTTGAAACTTA |
| comp106957_c0_seq23:148-149 | comp106957 | 148 | T | A | 62 | 0.112903 | 0.887097 | 7 | 0.857143 | 0.142857 | TTTTGAGGAGAGTTATTTTGGAACAAAATTTGACAGGCTGAAAGGTGATCCACGCTAGTTTTAAGTAGAAATAAAGAGTATGTAAAGTAAATCTTTCCTCT[T/A]ATACATTAACAATACTGCAATAATACTTTCTGAGGCAAATTTGCTTTAGTAGGTCAATGTTGTTACACATTTTTACCAATATAATATTTCTCAGGATTTTT |
| comp91528_c0_seq1:345-346 | comp91528 | 345 | C | T | 172 | 0.744186 | 0.255814 | 2 | 0 | 0 | CAGGCACTGGCCGTCGTGGGCGCAGGCGTTGGGCTGACTGCCTCCGAGGTGGGTCGGTGGACAGCTGGCTCGCACAGCGGGACAGGAGCCGGATCTGACGA[C/T]TGGGTAATTTCCTTGACCCTGAGAGACGTCTCGGCCGCAGCAGTACCTCTTGTTGTTCGGTCCCCGGCACCACACCTTGCACACTCCTTGGCCTCCTTGAT |
| comp107663_c0_seq11:1326-1327 | comp107663 | 1326 | G | A | 70 | 0.871429 | 0.128571 | 55 | 0.127273 | 0.872727 | TTCACCGAATCTATGGTGACTCAGCGAGATGTTCCAGCTGTATTCCACCTTGAGCGTCCAGGGATCCAGCTTCATAACCACCGTGTTGTTGTTGGAGGGCA[G/A]GCCGTAGATCGCCCACAGCCCGTTCTCGTCCGTGCTGAGGTCGACGTAGTCTCTGCTCTCCTTGTACAGGTAATTGGTCCCGTCCGTGGCCACTTCGGGGA |
| comp103127_c0_seq2:899-900 | comp103127 | 899 | C | A | 112 | 0.839286 | 0.160714 | 42 | 0.0952381 | 0.904762 | TGTAATTTCTTCACAGTCAACCAGTGTCTCAGTGTTTTTCTACAAGAAGTAGAAAAAGGATGAGGTACACGGCTAATTCTATGGTGAAGCAAACAGCCTTT[C/A]TTTAATGTGAAGTCAGAAATATATGTAGAGAATAACTGTGTCATGATTTTTGTACTCGCATTACTTGTAAAACTTTTCTTTTTTCTCTCATTCGAGAACAG |
| comp105551_c0_seq2:390-391 | comp105551 | 390 | C | A | 6 | 0.166667 | 0.833333 | 56 | 0.910714 | 0.0892857 | CCGAATACCGAAGGCTGATAGTGTTTTTTTTTAATTCGGTCGATACGACTTATTTTCGAAGGACTCGGTGAAAAAAAAGTATCGTGATTTTTAAAGTCTAG[C/A]GGGGTGTCATAAGCTCAATTTTGGAATCCGATTGCACGTTTGTCTTGCATGCAATCGAAGAAAGCTATCATACAAGCTCGTCCAGATGCACTTTAAGTCTT |
| comp92232_c1_seq1:538-539 | comp92232 | 538 | G | A | 59 | 0.79661 | 0.20339 | 76 | 0.0526316 | 0.947368 | ATTACTGATGTGGATCATAGTTTGATATTTTGATTTGATTGTATGATATATTTTTGTAAAATCAAAATCTTGCTAATGACAAATTATATTATTTAGGTATT[G/A]TCCTCCATCCAGGGACAAATGGCATTTCTAATTTTTATTAATTGCAGTTGTGCAGTTAGATATGGTAGTGAACTATGATACAGGAAATGTGCATGTGTGTT |
| comp107398_c0_seq1:140-141 | comp107398 | 140 | G | A | 64 | 0.84375 | 0.15625 | 30 | 0.1 | 0.9 | TTGTTATCCCTCCTCTCATCCTTCTTCTTTTTCTGGTTTGCCTTTCTTCTTTCCATCTCCTCCTTCTTTGCCCTCATCTTCCTCTCACACTGTGCACTATG[G/A]ACACTGAAGTTCTGTTGCAAGATGTCTTTGCCGCAGCTCTGACACCGTGCCGCTGACTTCTCATTCTCTTTGCCCATTTTATCCCCCGTTTTATCAGCCTT |
| comp106246_c1_seq7:152-153 | comp106246 | 152 | C | A | 65 | 0.923077 | 0.0769231 | 39 | 0.179487 | 0.820513 | CAGCACCATGACAAAGTGTACTCCTCTGTTAGTTATCTATTCCATTTACCACCAGAGCTGAAAATAGTGTTTTCTGTGGGACAAATAAGGTATCTTACCAA[C/A]GAATCAAGGTCACTGTTCCTTTCCTTATTCCTTTTAAGGTATAAACAATTGATAGGAAGTAATTATCACATTTCAGATAATTTACTTGTAGACTAGAATGA |
| comp107519_c0_seq1:638-639 | comp107519 | 638 | C | T | 58 | 0.0689655 | 0.931035 | 48 | 0.8125 | 0.1875 | AAATAATATCTATTAGGGTTAGACTTTACTTCTCTTTCTCTAATACAGGGAAACAAGTAATCTTTAGAAATGTCTAGATAATAGAAGAATTTCCTGACATA[C/T]TTCCTTGAGACTCAATGCCAAGACTGTAACGCCCTTTAAACCGTCTCAATTGTCTTTAGAAAAAAAGGGAATACAAAATGAAAATGATACAGGAAGAACAG |
| comp98117_c0_seq1:264-265 | comp98117 | 264 | A | T | 247 | 0.931174 | 0.0688259 | 133 | 0.18797 | 0.81203 | GGTTATCGAGAGAATATATGATTAATGGATTTGAGAGTCTCCTGTTGATCGAACAGAAATGGCTACCCACTAATGTACTGTACACTCACTCACTCACCGTG[A/T]TTGGTACCTCACTTTAGGCAAATCCCAGTGCATGAGACCTGCCTGTCATTCACTACTGAACGCACACACAAACTTACTCTTTATATGCAGATAAATATCAT |
| comp98805_c0_seq2:508-509 | comp98805 | 508 | G | A | 312 | 0.842949 | 0.157051 | 10 | 0.1 | 0.9 | TCCCGTCGACAGCGATAACCACGACGTCGTGGCTGTAGCGCACATGCTGCAGAACGCCCCCGCCCCCACGAAGCCCACCTCGGCACCCGAGCCAGCGTCCC[G/A]CGTCGACCCTGACGTATACAAGACCAGATTCTCTCAGAGATACGAAGTCGTTCCCCAGCAGAACTCCAACCCCGAGATCTCCCTTGGACCCATCACCGAAG |
| comp102182_c0_seq1:1059-1060 | comp102182 | 1059 | C | A | 65 | 0.784615 | 0.215385 | 24 | 0.0416667 | 0.958333 | GAAAGATAATAGTAAAGGTATATTATACTTAAAAAGGATAATATAAAAGAGCAGTATTATACAATACGTTTTTAGTAAATACATTTTACTTCATTGCTTCC[C/A]ATTTTTTTCCTTCTCGCTTACATTTTAATTGAAAACTGGAAGCATGAAATACATTGTGTAGTTGTACATTTAAAATTTTCGCAAATAAAATATGCCTTGTC |
| comp107196_c0_seq12:370-371 | comp107196 | 370 | T | C | 84 | 0.809524 | 0.190476 | 45 | 0.0666667 | 0.933333 | TGTGAATCCATTAGCCCTTCAGTAACTGAAGAAACAGTAATGACTGTAAGCATACAGCATGCAGGTGTTATTACATTACTAAGGACAGCTCTAAGGATAAA[T/C]ACAGTGACAACTCGACAAGACATGCTGGTTGTAGATCTTAAAAATCTTGGGTTGCCAGAGGAATTTGCTTCAGATGTCTGCAAGGTTGTGTATGGTCCTGC |
| comp106878_c1_seq18:2534-2535 | comp106878 | 2534 | T | A | 64 | 0.0625 | 0.9375 | 286 | 0.804196 | 0.195804 | TAAGAAAAATAAGAAAAAAATGTATTTAAAACATCACTATCCCTCCATAGGCTCTGTGCCTACTGGGTTTCTTGTCGCGTCCCTGGCTTTGTCGCGGAAGT[T/A]CAGGGGCGAGTCGGTATATGATCTAGAAAAAATGTTAGGACTGTTGGCTCCATCTCTCTGACACTGTATATTGTGTTTCTGTACAATGACTGGTGGGACGC |
| comp107386_c0_seq2:1338-1339 | comp107386 | 1338 | G | A | 137 | 0.832117 | 0.167883 | 11 | 0.0909091 | 0.909091 | CAAGGACTCCTTTGCAAACTTAGCTTAAGATCCTTTCAATTTTATTTTTATTTTCAATTGCCAGCTGTTAAAATGCTCAAAACAGCTTACACCGTCAGCAT[G/A]ATTTTGTCCCGTGATGTGTGCAGTCAAGAAAAATAAAGTAATGACTCACGCGTCGGTGAATGGCTTAATCATAAAATAAGTTTATAAAAAGTATTGAAAAG |
| comp107515_c0_seq3:1506-1507 | comp107515 | 1506 | T | G | 318 | 0.852201 | 0.147799 | 198 | 0.111111 | 0.888889 | GGTTTCGCGCTGTATCTCTTGGCACGCAACCCCCAAGCACAGGCAAAGTTGCATGAGGAATTAGACACAGTCCTTGGTGACCATCAGGGCCCGCTGCTCCC[T/G]AAACACATGGCGCAGTTGTCATACTTAAAGGCCGTGATTCGAGAAACCTTAAGGATCTTCCCAATTGCTATTGCCATGGTAAGGATTCTGGACCAAGACGC |
| comp107917_c1_seq1:3437-3438 | comp107917 | 3437 | G | T | 37 | 0.891892 | 0.108108 | 53 | 0.150943 | 0.849057 | TAAGTCAAAGCGTCGAAGGCTCCATCTTCAATTTCGCTCACCTGTGCATTATCAATTATCAAATGTGTTATGCGAAGTTCAGTCTTGAACAAACTCTTTTC[G/T]ATTGAAGTTAACATGTTGCCTGACAGATTTAATTTCTCTAGGTTGCTCATGTTATGGAAAGAGTTAGGAGCGATTTCCCTTATCATGTTGTTGTCCAAGTG |
| comp98677_c0_seq5:1430-1431 | comp98677 | 1430 | G | A | 361 | 0.930748 | 0.0692521 | 442 | 0.190045 | 0.809955 | AGAGGGAGCTTCATAAAGGCTAGTTGGGGTTGGCTCAGAGGGGGGTTCGTATAGATTGGTTGGCGCTGGGGCTTCATACACATTGGTTGGCGCTGGGGCAG[G/A]TTCTGAAGGAGCTTCATATACACTGGTTGGTGCAGGGGCTTCGTACAAACTGGTTGGTGCTGGCGCAGGTTCTGAAGGAGCTCCATATACACTGGTTGGTG |
| comp101541_c1_seq2:391-392 | comp101541 | 391 | C | T | 70 | 0.885714 | 0.114286 | 41 | 0.146341 | 0.853659 | ATATACTATAGATCTACGGACCAGATTCGATTTTGTTTTTTTGCTTTTTTTCTCATCATGTAGATTATAAGCAAAGAGAGAAGATCACATATAAAAGGCAT[C/T]TTTTCTATGATTTTCTTCACATCACTACTTCCTTTTCCTATGTACTCTTTCCCTAATCAACATAAATAAATAAATATAAGAGAACAGTTTCGACCTGCGAC |
| comp99473_c0_seq4:2908-2909 | comp99473 | 2908 | T | A | 98 | 0.765306 | 0.234694 | 76 | 0.0263158 | 0.973684 | CGACAGCCCAAGACCAAGAAGCTTAACCTCTCGTTCGACGACACGCTGACGCAGACGTACGAGTACCCGAGCGAGCTGTCCTTGATGGAGGAAATGGGCTC[T/A]CCCAGCGACGACCTCCTCCCGTCCAACACCACGCTCGACTCGCCCTCCTCAACGCAAGGTGGCTTGGCGAGTTACACCCCCAGTAAAATCCAGCTTGGTAG |
| comp99653_c2_seq16:185-186 | comp99653 | 185 | G | A | 395 | 0.822785 | 0.177215 | 119 | 0.0840336 | 0.915966 | ATGAGGACCTTGAGCCTTCTTCTCCTTGTGGTCGCCGTCGCCTACGCGGTAGAGGAAGCGTTCGTCTCTGGATATGATAATCTGAAAGAAATGTACGGCGG[G/A]GGAATGCACGCTCGACCTGATGGCTTGGTGATCCAGGTCCAACAGAGCGTCAACTCGGGAGGCTGTTGCGGGGATAAGAAGGGAGGAGATGACAAGGATGA |
| comp107599_c1_seq12:151-152 | comp107599 | 151 | G | A | 113 | 0.911504 | 0.0884956 | 81 | 0.17284 | 0.82716 | ATGTGAGAGACAAGGATGTGAACAATTTTATATAAAGCGAGTGATTAGTGGCATGTGAGGGAGGGGGGGGGCACATGGCCACCCTGGCACCCCCCATCACC[G/A]ATTACTGTATATCCTTATATCTCTTAAAATGAAGACATTAATAAATAAACAACTAGATAAGTAGATGACCAGATAAATAAATAAACTTTTGTCTAGATCAA |
| comp106341_c0_seq16:2027-2028 | comp106341 | 2027 | C | A | 82 | 0.829268 | 0.170732 | 22 | 0.0909091 | 0.909091 | GAATATTTCGGGTACCATGGTGGGTATCACAATGAAAACGGCGAAGGAATCAAAGAAAACGGAGACGGCGACGAAGAAATCAAAGAAAACGGAGACGGAGA[C/A]GAAAATAAAGAAAACGAAGAAGACGAAGAAAACAGCAATGAAGAAGAAGAAAACGGAGACGAAGAATCCTCAGAAGAATCCTCGGAAGATTCCTCAGAAGA |
| comp97628_c1_seq1:952-953 | comp97628 | 952 | T | C | 29 | 0.931035 | 0.0689655 | 57 | 0.192982 | 0.807018 | TTGACCTTACTGGTGCCGTTACTGGATATGCAATGCTTAACCATGATAGCGTGTACAGATGCGAGAACAGAAAGGTGACGGGATATCCTTGCAAGACCAAC[T/C]TACCCTCTAACACAGCCTTCCGAGCGTTTGGGTCTCCGCAGGCCATTATTATCACCGAGTACATGGTTTCCAGGGTGGCAGAGTTCCTCAACATGGATCCT |
| comp106875_c0_seq1:482-483 | comp106875 | 482 | A | G | 77 | 0.909091 | 0.0909091 | 35 | 0.171429 | 0.828571 | GAGCAGAGCAAGGCGTCCGAGCCCTTCCTCTCCTTGCCCTCCTCGCCCGGCTTGCGGAAGGTCTTGCTCAGGTTCTCCTTGCAGTCGGAGCGAGGGAACCC[A/G]CCGATTCGCTGGACGTCCTCGCACGACGAGAGGTTCTCTTTAGTCTCCCCTACGCTCATCTTCTCGCGTTTTCTGTCGTGGTTCCGGGGGATGAAGAAGGA |
| comp103963_c0_seq1:1805-1806 | comp103963 | 1805 | G | A | 51 | 0.882353 | 0.117647 | 138 | 0.144928 | 0.855072 | GCCAACAAAGCATTAGATTCTGTCCATTTCACAACAGAGGATGGAATAGGAGAGAGAAAATTAGAGGAAAAGAAAAAGAAACAGAAACAGATAAAGGCGTG[G/A]ATAGGAAAGATAAAAAGAGACAAAGAAAGAAACAAAGAAAGATAAAGAGAAACAGAGAAAGGCGTGAATAAGAAAGATAAAAAGAGAGAAACAAATATGGT |
| comp107397_c0_seq2:329-330 | comp107397 | 329 | G | A | 276 | 0.898551 | 0.101449 | 93 | 0.16129 | 0.83871 | ATAAACAAAAAAATTCAAACGTTCAAACAAAAATATCAGTATATCAAGTTTAATAGGCTGAGTATACTGTGACACTATGTATAAAATATTACACACCAACA[G/A]CACATATACATTTATATATAAACATGAATATATGCAATCTGAGTCCTACATAAGTGTTATGTGCTTTAGTCTTTAGTTCAAGTGCATATGACAATAAAGAA |
| comp103454_c0_seq11:283-284 | comp103454 | 283 | G | A | 86 | 0.767442 | 0.232558 | 33 | 0.030303 | 0.969697 | GACTAAGACCTCGACACGATCTTAAGGGTCCCGAGTCAGTAGGAAAGTTCTTGGTGTTTATTGGTTAAGATCTCCAGATTCTCGTGTGTTTTGTGTCTTTG[G/A]GATTTTGTGTCTGTTTGTGTGCCAATCATAGGTTAAGGGTTTTTTATTACTCGTGTATATAGGGAGCTTTATACTTTGCACAGAGCTTGTACATTTTATAC |
| comp107181_c3_seq1:410-411 | comp107181 | 410 | T | C | 122 | 0.885246 | 0.114754 | 81 | 0.148148 | 0.851852 | TTCCTCAAACCTGTTCTAACATTATATCCACTATAATGCCCATGTGCTCTTTTACAATACAAATATTCAGTACACATCAATAAATACATTATGATAATGTA[T/C]CACCTCTTTATTTCCCTTCTTCTTTTGAGCCATGAGGTGACTGGTAACAATTAACAAGTGTGTGCATGTGTTAGGGTGGAGTGGGAGGTGTGTTTGCATAT |
| comp106574_c0_seq3:524-525 | comp106574 | 524 | G | A | 56 | 0.785714 | 0.214286 | 41 | 0.0487805 | 0.951219 | CAACGAAATAAGCTCGGCATCTCAGTGTGGGGAACTGATGTTCCTGATCCAGTAGAAACTTTTGATGACATGATTGTGAAGCACAAGCTAAGCGAAGTACT[G/A]GTGAGCAATCTTTTGAACCAAGGCTACTCAGAACCCACGGCAATCCAGAAGCAGGCATGGCCACTCATGCTGCAAGGACGGGAAATCCTGGGATGTGCACC |
| comp107021_c0_seq3:410-411 | comp107021 | 410 | A | C | 209 | 0.832536 | 0.167464 | 104 | 0.0961538 | 0.903846 | TTTATCTTTGAACTTTGAAGGGAGCTTGGTTTCGTTATATCCTTCAAGTGTTAGAGAAGGGAATGCTCAGATTTGGAATGAAAACTTCCCAACCACTGTTC[A/C]AAGGTTGAATACTTGCCATAAAATATTAACATGAAGTTCCATCTGTTCCAAAATGAACATGTCAAACAAAATAAATTAAGAAAATCTCAACCCAGCAGTAT |
| comp98983_c5_seq1:1960-1961 | comp98983 | 1960 | A | G | 118 | 0.779661 | 0.220339 | 23 | 0.0434783 | 0.956522 | TTGTGTTCCATGGAGCTTTCTGTGCTGAAAAAAGTATTTACTAACATAGAATTCATGTCCATTTGAATGAAGATGATTACAATGTTATAAATGTGTTTAGT[A/G]TATATTGGCTATCCTTTTTCTTTCTTTCTTTTTTTGTATCCCCTTTTCTTCATCATGGGTTTTAATTAAGGTGCATATTTTGAAGAAGATGGTGATAAGTG |
| comp105004_c0_seq2:899-900 | comp105004 | 899 | A | T | 146 | 0.0684932 | 0.931507 | 46 | 0.804348 | 0.195652 | CTGTCTCTTCTCCCTAACGGTAGTGACTGAGGCATCAAGAAAACTATTTGTAAACTTATACATCGCATTAGTATCATCACAGGCATAGTTTATGTAGAAAA[A/T]AACGTTGGTTGTTTATGTAGAAAATAACGTTGGTTGATCTTCAGGTGAAAATGATCACAGTCAAATGGTGTTTTGTGTGTGTATTGTATGAGGCGAAATCT |
| comp90723_c0_seq1:138-139 | comp90723 | 138 | G | A | 53 | 0.735849 | 0.264151 | 1 | 0 | 0 | CTACACACCACTTATATAACTGTTCCCGGAAACATGCCTCCTTTACTATCTATGAAAAATAAAAATAAAAAATTATCCCCAATTTCTACAGCATTCGTGAT[G/A]TGGGGCTGAAGGACAGTGAATATAAACCACATTGCAAAACTCTGGTCGCCGTGGTACCGACGGCTTGCAACATCTTGCGTGCAAGGGGCAGCAGTCAACGT |
| comp106609_c2_seq2:996-997 | comp106609 | 996 | G | C | 68 | 0.823529 | 0.176471 | 34 | 0.0882353 | 0.911765 | CATGTTAATATGAACAATGTTATTATCTTGGAACGGAGGGAGAGATATTAGTGCTTTAATGAAATGCAAAGAAAAGAAAGGAAATGATAATGTTGGTGATG[G/C]TTATTATCGCTATGATGATGACAACAATGATGATATTAATGATGACGATAATGATAATGACAATGACAATGATAATGATAATAATAATAATGATAGTAATA |
| comp107483_c0_seq2:3909-3910 | comp107483 | 3909 | G | T | 82 | 0.878049 | 0.121951 | 7 | 0.142857 | 0.857143 | CAACAGAAAATTCCTAGCCCATACTTTTACAGTAATATATCATCATCTTAATGAAAAGGAAACAAAAAATACACATCTATGAACAGTGCCCAAAAAAGATT[G/T]GTCCAATGTGACTCAGTACAAGTAGTTAGAAGACCTTATGCACAGTACAGTACAACACGACCTTATGAAAATCCAAATTAATAAACTGGGCAGCACAAATT |
| comp103982_c1_seq1:1519-1520 | comp103982 | 1519 | A | G | 6849 | 0.736166 | 0.263834 | 5341 | 0.00112339 | 0.998877 | TTCTTAATGATGTGCCTTTTCACACGACGGACCTTCTTGAATGGCTGGGCGCGCTGTGACCTCAAGATAGCAGAAGCACGACGAAGAGCAGCCATCTTCAG[A/G]TCACCCCTGTAGTGGTTGAAGCGGATGAATCTCCGGATGGAGTTCAGAGCTCTGCGGGGACCAGACTTGATAGTAGTCTTGACCATGTTCTTGGCTGGCTG |
| comp103801_c1_seq2:147-148 | comp103801 | 147 | C | T | 109 | 0.770642 | 0.229358 | 28 | 0.0357143 | 0.964286 | AAAAAAAAAACGAAAACAAAATTCCCCCCAAAAAAAAACGTTTTGAAGGTGAATTTTCCTTAGCACACAGACTGTACATATGTCCTTCTCGAATAGATACA[C/T]CTCAACGCTTTGTTTTTCCCGCCTTGTCACACCAATTATTTTTTTTTTTCAGTATAAACAGAGAATCGACGTTTGTTTCATTCAGGATTTTTGTACAACCG |
| comp106286_c0_seq10:1490-1491 | comp106286 | 1490 | C | T | 37 | 0.0540541 | 0.945946 | 104 | 0.788462 | 0.211538 | GCAGAAGGGGAACGCGCTGTGGCGGGAAAAGCAGACGCGGTGGAGGAACAAGCAGCAGGTGGAGTTCGCGAGGCACCTGAGCGTGGGCGAGGACGAGGACG[C/T]GGCGCGGGAAGGGAGTGGGAAGGAGGAGCCGGAGGAGAGGGAAAATGACTCGGAAAAGGACTCGGAGGAGGGCGTTCCTGAGGACGCATCGCGGGAGGGGA |
| comp105503_c0_seq4:187-188 | comp105503 | 187 | A | C | 64 | 0.765625 | 0.234375 | 32 | 0.03125 | 0.96875 | AATCTAGTACTTAATTTTCTACCATTTTATTACAATATACAGGCAAGGGTAGTGTTCATCTACATGGAGGGGAATGCCAAAGAAAGTTGAGTAAATAAAAA[A/C]AGTTTACTAAAATCATTTATCTCCTAGTACACCTGAATTCCTAAAGTGCTCATCTCTCTCCTGAGTCTCTGGCCGTCAGAGAACATCTCCTGGTAGTGCGT |
| comp107129_c1_seq14:812-813 | comp107129 | 812 | A | G | 399 | 0.0275689 | 0.972431 | 105 | 0.761905 | 0.238095 | ATGTATTCGCTAACAGGGGGGCTGACGTGGGCGACTTCGTTTGGCCCAGTGTATTCCTCATCAACGGTTGGAACAGGGACAACGGGAGCCACGGGGGCAAC[A/G]GGAGCGCTGTATTCTTCGTCAACAGTCACAACGGGGCCAGCGTCGATACTTGGCCCAGTGTACTCCTCTTCAACGGCAACGATGGGTACAACAGGGTTAAC |
| comp107092_c3_seq8:164-165 | comp107092 | 164 | T | A | 91 | 0.802198 | 0.197802 | 58 | 0.0689655 | 0.931035 | TCATAATGCTGTATGACCTTTTCTTACCAGGGGGGCTTTGCCCCCAAGCCTACCCCTATTGCTATATTTTGTATAAGCCACCAAAGATGATATAGCAGAAA[T/A]TTTTCAATAAATATATAATCAAAAAAGTTGGACAAGATGTTTTTACAGTTTAAAACTTTCCATCTCCACACACCTTTGTAGCTGGGTGAGCAGGACAAGAT |
| comp104154_c2_seq6:194-195 | comp104154 | 194 | A | G | 207 | 0.768116 | 0.231884 | 355 | 0.0366197 | 0.96338 | TAATCAGGGGGATGTGATGGATCAAGTACTATCATGAAAGAATACAAAGCAGGAATCAACGGATTTGACAGATTTACTTACACAAGCAAACTGTTAAGCGG[A/G]AAGAGGGTCCTCACGAAACGACACAAAACAAATTTCAACGGAAAACAGATTCAAAACGTTTTTATTTCAAGTGAATAAACGAATATACATGTTAAAAAAAA |
| comp104086_c3_seq3:160-161 | comp104086 | 160 | C | T | 84 | 0.892857 | 0.107143 | 74 | 0.162162 | 0.837838 | GGGAGGTCTCCAGCCCACGTCGCGCCCACTTCCGTTTGTCCTTCAATACGTATTCTGTGATATTTATAATCTTAGGAAAATGCGATGGTTTTTCCAAGCTC[C/T]GTGTGTGTTTTGTTGTATAGTGTTTGCATCAATTATTTTTTTTTTCTCTCTCTCTCTCCATTCGTTGTGTTTCCAGTTGCCGTCACTTGCATTTCTCTTCA |
| comp100748_c0_seq3:209-210 | comp100748 | 209 | T | C | 265 | 0.822641 | 0.177358 | 228 | 0.0921053 | 0.907895 | GAGGAAAGACATTTTGGTTTTGGTTATAAGGATGGTGAGATTCTGTGGCCTCAATATTTCGGAGAGATTTGGCTGTGGCGGCATCGGACTTTTAAATCTGG[T/C]ATTCAACCTAACTAACTTCATCGGATATTTGGTCTTAGCGATAAAAGGCTATGATCACGTGACTTGGGCAGTTGCCATATCCACTGGAATCGGAATTCTTA |
| comp100911_c1_seq1:1065-1066 | comp100911 | 1065 | C | G | 101 | 0.841584 | 0.158416 | 9 | 0.111111 | 0.888889 | CAGGCCTTCAGGCCGGCCCAGCGCGTGGAGATCGACGTCACCCGCGAGGACAACTCGCGCGAGCTGTTCCTCCAGAGCGTGAGGAACGGCGGCGCCCTCGG[C/G]CGTCCGGCCATCAACCCCGGCCGCTCCTTCAGGCCGCAGGGCGCCGTGCGGCTCGAAGTGGACGTGACTCGCGAGGACCTGACGCGGGAGTTCAACTCGAG |
| comp99836_c0_seq1:330-331 | comp99836 | 330 | A | G | 462 | 0.898268 | 0.101732 | 95 | 0.168421 | 0.831579 | TATGGAGCAGGCTTAGTCATTCAGGTAAAGCAAGTTGTAAAGGGTGGTTCTTCCTGTTGCTGTGGAGGCGGTTGCTGTGGTCATTGTTGCCATCGTAATGG[A/G]CAGGATTGTGCCGAGGAAGAACCTGAAACGACTGCAATTCCATAAGAAATTATATGGAAAAGAACAGCCATATCCAGAGCCAACATCTTTATTAGATACCC |
| comp99428_c0_seq1:351-352 | comp99428 | 351 | T | C | 85 | 0.8 | 0.2 | 57 | 0.0701754 | 0.929825 | TAAATAAAAATCAAATGAAATATACTACCAATTGATGATTCTTGTTTTCTATGTTTGTGCACTGTTAATTCTTTTATCATTATTGCCACCTTCCCTAAACA[T/C]CATTCAACAGGAAATCTGTGAAACACATTGCAATTTTTATGATCATCAAATGCAACTGTACTGAACTGACATACAAACTGTCTTGCATGCGACAATATTTT |
| comp102469_c0_seq1:418-419 | comp102469 | 418 | A | G | 74 | 0.72973 | 0.27027 | 2 | 0 | 0 | GCGTCCCAGGCCCATCGGTAGTGCAGCGCCCGGCAGTACCCGCGCTGCAGAGGCTGCCCGCAGATGTACCCGGCCTCCTCGACACTGCAGTTGCAAGTGTC[A/G]CAGCCCGTCTTGGTCTTGCCTCGCGTGCAGCTGTCGGGGCAGTCGAGCACAGGACACCGCAGCTTCTGGCACGCCTCCTCGCACGCCATCCTGCTGGGGAA |
| comp103631_c3_seq2:403-404 | comp103631 | 403 | A | G | 897 | 0.80825 | 0.19175 | 227 | 0.0792952 | 0.920705 | GGCGGCTTCCCAGGGGCCGGAATCTCCCAAGGATCCAGCGGCTGCAGGTACTGGTGCAGGAACCCCGAGAACCAAGTGTACTGCTGCGAGACCGATCTTGA[A/G]CCCGAGGGCCCCGTCGGCACCAAGCCCCTCGACTGCCCCCTCGTGCGCCCCACGTGCCCGGTCAGCGTGCGCGGCCTGCGCCCCATCACCTGCTCCAACGA |
| comp102275_c2_seq14:1369-1370 | comp102275 | 1369 | A | G | 97 | 0.742268 | 0.257732 | 75 | 0.0133333 | 0.986667 | GACCACGTATGACCATGATGCTGTGTACATTGTGGGGGGTATAGTAGACACAGGCGAGAATGAGCCAGTGACTCTAGCCAAGGCCAAGAGGGAAGGCATAC[A/G]CATGCAGAAACTGCCGCTTGACAGGTATCTGGAGTGGGGTATTGGAGGCAAGTGTCTGACACTCAATCAGATAGTTAACATCTTGCTGGATATTAAGCATA |
| comp86993_c0_seq1:143-144 | comp86993 | 143 | A | T | 20 | 0.2 | 0.8 | 56 | 0.928571 | 0.0714286 | GGAAGAAAATTAGCAAGAAGTGGAGGAGGATTAGCAGGAAGACGAGAGATAAGAGAAGGAAAAATAAATGTAATGAAGCAAGCTCACCAAAGCACGTGATC[A/T]ATAACCTTCGGGGTTTCGTGACCTCGCAAATCTCCTTGGTGGTTGAGATTTTGTGGTTTATACAATAACAAAGATAACGCATTACATTATTCTAGTTAAAA |
| comp102608_c0_seq1:973-974 | comp102608 | 973 | C | T | 128 | 0.796875 | 0.203125 | 117 | 0.0683761 | 0.931624 | GCTGCTGAGGATATTATTAGTAATATATTTCGTGTTTCTAAAAATACCAACATGTCTGAGTACCTGAAACTAGAATTTATAAAGGAAATTGGTTACACCCA[C/T]TTACGTATCGTTCAGGGGAACTCCTCTCTCCTCCAGTTGTCAGGACTTCTGGCCAGATTGTGTTCCAAGTCTCTCCAGCCGGTTGCAGCATAGGACCAGAT |
| comp101888_c2_seq4:343-344 | comp101888 | 343 | A | G | 81 | 0.728395 | 0.271605 | 1 | 0 | 0 | CCCTGCAGTTCCTGACGCCGGCAGAAGGGGAGGAGATTCCCGACGACCTGCTGTGGGAGCCTGCCTACGAAGAGCAGTTGGGAAGGCTTAGTGCCTATTTT[A/G]CTCATCTCGAGCTTCCTACTATGTCCTGCCAAGAGCGACTCCTTTGCGAATTGGCTGCTGATCCAGACAGTTTTTCGCCCATCGGGGAGATCTTCATGAAG |
| comp106475_c2_seq6:1304-1305 | comp106475 | 1304 | A | C | 111 | 0.774775 | 0.225225 | 43 | 0.0465116 | 0.953488 | ATTCTACCGAATAACAAAACCTATCATTCATCAAACCAGTGCTTACCTGTGTTTTATGCAGCAGGAGTTAAATTTTCTGGTCAAGTTTACTGCTCCCATCA[A/C]AGGGATTCCTTTGATCTACGAGATTCAGCAGTATTGGTCTTCAACCCAATCGATATGCCTCCAACTTCACCTTCTTTGATCTATGAGATTCAGCAGTATTG |
| comp93479_c0_seq5:158-159 | comp93479 | 158 | C | A | 257 | 0.155642 | 0.844358 | 43 | 0.883721 | 0.116279 | CCACACCGGGAGAGACTACCACACCGGGAAGGACTACCACATCGGTACCTGAATCAACTCCCACACGGTAAACAATAACCACGCCGATTAACCCAGACGTG[C/A]CAGTGTGTGAGGGCGACCTACAGCAAGGAATCCTCCGAGGAAAGGGGAACTGTCATACACTGAACAGCCACTCTGATAAACCGGCGCTGACCACTGGCAAG |
| comp98605_c0_seq1:415-416 | comp98605 | 415 | A | G | 1094 | 0.858318 | 0.141682 | 23 | 0.130435 | 0.869565 | TGTGGTGCCCAGGATGGACAGCCATCAGAGGCCAAGCCGAGACCCGTAGCAGATCGGGCGTGGTAGGCAGAACTACGCAGGACTTCGTCAGGAAAGCTTTC[A/G]GCGCGGGTCTCATCACCGAATCAGAGGCTCAAGTTTGGCTTAATAGTTAAGGCGAAGAAGAACGACACTCAGATATAATTTATAAGAGCGCCATGGAAGGG |
| comp98406_c1_seq1:783-784 | comp98406 | 783 | C | T | 135 | 0.888889 | 0.111111 | 111 | 0.162162 | 0.837838 | TTAAGTTCTCCATATAATACCTAAAAATATTCTCCTGAAACTGTGAGCAATTTATTTCAACTGATGATTTTGTTTGAACTGGCTTTACGTAAATAACTCAA[C/T]GACAAGACTTAATAGGTAAAAGAAAAAAAAGGAAACAAAAAGAAAAAAATTCACCATTGAATATTCCCAGACCTTAATCTTTATTACTACTGCATGACGTG |
| comp102223_c1_seq2:339-340 | comp102223 | 339 | C | T | 189 | 0.920635 | 0.0793651 | 165 | 0.193939 | 0.806061 | ACACACACGCACATGCACATCGCATGCATACATACTTATATACATACATACATACATAAACACACATAAACGGCTTCACATTCTAACAGACAGCACTAGAC[C/T]TAATGTGAAACGTTTCAAAATAACGGTACTTTTCATTTACCTACGATCCATTTTTCTTAATATCATAATTTACTGAATCACAATAAATTCGAAATATCAGA |
| comp106359_c1_seq12:1103-1104 | comp106359 | 1103 | C | G | 328 | 0.896341 | 0.103659 | 276 | 0.17029 | 0.82971 | GAGCCGCCCATTTGGCCACAGAGTGTTGGAGATTTGCTCTGCAGTCAGTATGAGCAGCTATCAAAGGAACACCAAGAACTCCAGAAGTCCTACCAGTTACT[C/G]GTATCTACCTCTTCAGCAAGCTCCCCCGCCGTTGATCTTAATGAAGGTCCCAGTGAGTGGTGGTCAGCAGCTCGTGTGATGGAGCTGGAAGCACAAGTTTC |
| comp102255_c1_seq8:332-333 | comp102255 | 332 | G | C | 2 | 0 | 0 | 51 | 0.72549 | 0.27451 | TTCGGAAAGTACCCACGCATCTCCAAGATTTCCCAGATAAACATTTTTTTTCTTTTTTTCAGCGCTTCTGCAACTCACGCCTCGTAGAGTTAAGTCCTCGT[G/C]TTTGCAAAATACTGCAAATGCAAACTAGTGCACAAAGCACGGTGTTGCCCATTTATAGATCTATTTTTTTTCAAATTTATTACTATCTATATGCTTTACAT |
| comp107953_c0_seq6:410-411 | comp107953 | 410 | G | A | 125 | 0.92 | 0.08 | 41 | 0.195122 | 0.804878 | AAAAAGATGAGCCGAGCCTCTTTTCGCAGCGTCTTCAACAAGAGCCATCAGGAGAGGCTTCAGATAGATCCTCCAGGAGCCACTTTCGACCTCTTCCTCTT[G/A]TGTCTCCTTCTCGAACACGGCACCGATAAATTGGCTCCTTCCGGGGACGAGTTCTGGGTTGAACCTGGGAATCATCTCGAATACCAACTGACAGCCTTGAA |
| comp106465_c0_seq15:935-936 | comp106465 | 935 | T | G | 54 | 0.851852 | 0.148148 | 47 | 0.12766 | 0.87234 | TAACCTCTTTATCTATGGAAAACAAGAATTACAAATGTTGTTCTTTTCTTCCTCCACTCTAATAGCTGGTGCAACTTGTATTTTTTCTCTGATTTGCGCGG[T/G]AATCCTTGGCCTGCTTGACAAGAGACGCTCCAAGGTGCTGCGGCTCAATTCTGCCGAGTCTGGAGAAAAGGTGCAGCTTAGGGATATTCTAACTTTCCCAC |
| comp93932_c0_seq1:279-280 | comp93932 | 279 | T | C | 58 | 0.724138 | 0.275862 | 1 | 0 | 0 | AGATCGGGCAGAAGGTGCAGCTGCCCGCAGATACTTAGGAACTGAGATCTCCCTGCGTTTTTCATGATCTTCCTAGGCCTCGAGGCAGTGGTTGGAACACG[T/C]GGAATGCAGGGATCGAGACGCGGCCGTTCTCATCTGCGTATATCTACGTCATATATGAACGTCTATCGCTACAATAGACATTCTTCAGTCTCCGCAAGGCA |
| comp102257_c0_seq14:312-313 | comp102257 | 312 | C | G | 188 | 0.164894 | 0.835106 | 9 | 0.888889 | 0.111111 | GAAGTGCGGGAATTACCGCTGTTCTAGTGATCCTGGCGATGGCTTCTGGCGCCGAGGCTCTCGCTGTTAACGAGACGGATCCTTCGCGGCAGTCGGGCTTC[C/G]TGTACCTGACGCCGGAGCGGAGGCTGACACTGCCCCCCGAGAGCGTCCTGGTCCTCACGCCCACGCTGAGTCTTCCTATGGGGCGGAACCTTCCCTTCGGC |
| comp93760_c0_seq1:165-166 | comp93760 | 165 | C | T | 105 | 0.72381 | 0.27619 | 2 | 0 | 0 | ACATCGTACTACAGTGTACATTTGTCTTTTCTTTTCCAGTTATTGTTGATAGCTAGAGCAGATGATGAATCCTCTTCCTAGTATATACGCTATAAAGCGGG[C/T]TTCCTGTGACGAAAAGCTTCTCGTCGTTCCTTTCCTCTTCTTTCTTCGGTTGCATTTCGGGCTTCTTTTGCTTCCATATCACACATGCATATTTACACGGC |
| comp106859_c0_seq11:738-739 | comp106859 | 738 | A | C | 72 | 0.777778 | 0.222222 | 37 | 0.0540541 | 0.945946 | ACCCGACTTGAGTTGTGTTTCCGTAACAATCCAATAAGGCGATTTACCAAAAGAAAATGACCAAAGTGATTGGTGGCCATGGTGACTTCCAGGCCGTCAGC[A/C]GTCAGCTCTCGAGTGTCAGCCCCGCCGGTACATGCTACCATTACCAGTATATCTAGTCTCTCTTCCCTCCTAACGAAGTCGTCCACGAAGTTATTAACAGA |
| comp105971_c2_seq3:378-379 | comp105971 | 378 | A | G | 52 | 0.923077 | 0.0769231 | 10 | 0.2 | 0.8 | TAACGGAACATTGTTTTTAAAAAATATCAAACGAAGTGCCATTTTCTCGTTAAAGAATGACGTCACTTTATGTATAATCTACAGCACCGTAGGCCTTTAGT[A/G]TTTTCAAATAACCATACATATATATTGTTTTATATTAGCAAAGTAATTGCTTGAAATATTTTATCTTGACTGACTGCGAATACGAATGACGGAGCATCTGC |
| comp81782_c0_seq2:134-135 | comp81782 | 134 | A | C | 65 | 0.723077 | 0.276923 | 2 | 0 | 0 | GAAGTCGTATTTGGCGGGACCGTATTCCTCGGAATTTTCTCCTGAGTCCTGTGGGGCGCGATAAACGGAGGAGGGACGAGACTCGAGAGACTCAGCTGAAG[A/C]TCCTCCTCCATGGCGTGGAGATTCGTAGAAGTAGGAAGGAGGAGAATCAGCTGTGACGACAAGAGCCGTGACAGCGAGAATCCAAATCTGACACTTCATCT |
| comp104190_c1_seq3:1590-1591 | comp104190 | 1590 | A | C | 151 | 0.741722 | 0.258278 | 107 | 0.0186916 | 0.981308 | AAAAGATGCTGACTTAATATAGATGCAATTGTCTTTCTGAACTTGTACAATTTCTTCTATCATTTAATCCCTTTGTTTAACTGTGTGGCATGGTACAAATA[A/C]AGGATTTCAGAAGCAGCATTTTGATGACAAGACCTTATGTCCAACGAGGAAATACATTGTGGTCTTTTGGCAAGTTTGTATGTGGAGAGATTTCTTGCTTT |
| comp100189_c0_seq3:1341-1342 | comp100189 | 1341 | A | G | 100 | 0.76 | 0.24 | 108 | 0.037037 | 0.962963 | GCACATACCAAGGTCCCTTTCCTGTCAGTGGGTGAAGATCTAGGACACAGAGAAGAGCGCATAAGAGGCCATAGCCAGTTTTCCGGGGACTATGTGGTTGA[A/G]GACGTCCATCCCCCGGGCGCATCTAAACTGAGACGACTGATTTTCTTGGCCAACCAAAATGTTATACAGTCAGAGGCTAAATTGTTAGTTGTTAAGGACAA |
| comp104746_c0_seq1:921-922 | comp104746 | 921 | T | A | 67 | 0.910448 | 0.0895522 | 16 | 0.1875 | 0.8125 | GAAGAAGAAAAACGTAAAGAGAAGAAAGCAAGCAGAAAGGAGGCGCGTAAGAGGGTGAATCAGTATCCCGAATACTCTGGCAGCGAGTGCATCCTGGAGGC[T/A]GTGGATGGTCCTGATTCCGAGGATAGGGTTGTCAGAAAACCCCGACCAGAACCGTCCTCCATTCCTGTAATCACAGGAGGCCCCTGGACAGACGAGGAATT |
| comp98428_c0_seq13:177-178 | comp98428 | 177 | G | A | 296 | 0.837838 | 0.162162 | 624 | 0.115385 | 0.884615 | AAAGAGGCTTTCCGTATCTACGACAAAGAAGGCAACGGCTACATCACCACCGATACCCTGAAGGAGATTCTGAAGGAACTGGACAACCGGCTTACCAACGA[G/A]GAACTGGACGGGATCATCGAGGAGGTGGACGAAGACGGGTCCGGGACTTTGGACTTCGATGAATTCATGGAGATGTTGAGCGGTTAAATCATCACATTTCC |
| comp101719_c0_seq1:1165-1166 | comp101719 | 1165 | C | G | 423 | 0.898345 | 0.101655 | 697 | 0.176471 | 0.823529 | AGGCCGATCAGCTGGTGCGCGACGGCCAGGGCGATGGTGGTTTTTCTGCCGTCGATGACTTCATGTTCTTTCGGAAGAGGTGACGACGCCGTCAGTACCAC[C/G]AGCGCCAGGGAAAGGACAAACAACGCCCTCAGCCGCATCTTGGTATCTCCTTGTGCTGCAGGTCCGACGTCTTGAGAAAGAAGTGTGAGGACCGAGAAAGT |
| comp101217_c0_seq3:167-168 | comp101217 | 167 | C | T | 114 | 0.72807 | 0.27193 | 154 | 0.00649351 | 0.993506 | ATCGATGGTAAATACAAATACATTTTATACGATTTTTCTTCTTTTTTGGCTGGGAATGTGAGGTTATGTTCTTCAAGAAATCATAAACCGGTAAAAGAAGA[C/T]AAACTAAATCCAATTTACTTTCTTGAAGTTGAAGATCACTTCCACAACCCCCATTACCATTCATGACTTCCTAATCTTTTACCAAAATTTCCCCCTGTCCC |
| comp104380_c1_seq3:1377-1378 | comp104380 | 1377 | T | C | 52 | 0.942308 | 0.0576923 | 18 | 0.222222 | 0.777778 | AAATATAAAGTCATATATGAAAATACATATGGTGCTGAAAAGAAAAAAAAAAAACAATAAGAAAGGAATATTTCCACTAGGATTTTACGTCCTGATGAGGA[T/C]TCTAGTCCAACTCAAAACGTCACACTTAAGTATTCCTTTCTTATTGTGACTATGCTACATTTCGTCTTTGTACACATTGCTTTCTTTGTATTTTTTTGCGT |
| comp107033_c0_seq1:157-158 | comp107033 | 157 | A | G | 464 | 0.771552 | 0.228448 | 290 | 0.0517241 | 0.948276 | GAGACACAGCTCAGATCACTACGTAGGAAGAGGCTCGTTCGTGGCGAGAGATGAAGTTCCTGAGCGGGCACGAGGCGAAGGCGATCGAGGGCGAGGAGTGG[A/G]AACGCCAGAAGAGTCACTGGCTGGGTTACGCTGATGGCTTGGTCCGCTTCTCCCCCGGTGGCTGGGTCCTCCCGACGCCCTTCACCAAGTTTGCTGATAAA |
| comp107245_c1_seq2:473-474 | comp107245 | 473 | C | G | 80 | 0.9 | 0.1 | 61 | 0.180328 | 0.819672 | GCGCAGAGCATCGGGGTGTGGCAGGATGCAATGGAGCTGATGGGTGTCCTCGGAATCATGGTCAACTGCATGTTAATTGGCCTCTCTGGGCAGGTGCACCG[C/G]ATGTTCCCCGAGATGTCGACGGCGCATACGATATTACTCATTATTGTACTGGAGCACGCCATGATCGCCCTCAAGTACGGCATCAGCTACGTGATCCCGGA |
| comp85502_c0_seq2:258-259 | comp85502 | 258 | C | T | 87 | 0.827586 | 0.172414 | 37 | 0.108108 | 0.891892 | GCTTCAAAGAAATCGATGACACAGCAAGATTGAGTGATCACTTCTTCTCATGGCAGTATATAAGAGTCGCAGTGCAAGCACTCGTCACCACACGCGGTTCA[C/T]CAAAGCAGGAGATGAAGCTGATAGTGGCGCTGGTCGGCACGGCTTGCTTGGCTGCCTTCTGCCAGGCGGGAGGACATGGAGGCGGTGGACACGGAGGAGGT |
| comp100937_c1_seq1:413-414 | comp100937 | 413 | C | T | 15 | 0.2 | 0.8 | 74 | 0.918919 | 0.0810811 | ACACACCACGTCCACTCGACTCTTCCGCCGATGCCCTACCCTCTTCCACGCGTCCACTCGCTCTCTCAGTGGATCAGCCGGGGCCAAAGTCACCCAGAGGA[C/T]GAAAACCTCCTTCAGTCGCCGGGAAAAGGACCTTAAGCTCAACACGAACTTAGGCGAGAACTTGGACAACAAAAAATATAACAAAAACGGCGACAAAAACA |
| comp102724_c0_seq1:413-414 | comp102724 | 413 | G | A | 372 | 0.201613 | 0.798387 | 163 | 0.920245 | 0.0797546 | AGAAAAATGGCATCAGTTATTCTTTATTATCTCAGTTGTTATCTTCATCTTCGTCTCTTGTAATTCGAGTGGTTTTATTTCGGATAATCATCTTCAAAAAT[G/A]AAAAGAATATTTTCCTTGTTATAAAAAAGATGATTATTTTTCGCATCAAAACGTCATTTCGTTATCCAATGATAGGTTCCTCCTGCGAATGACGTTTTTAC |
| comp104850_c2_seq5:656-657 | comp104850 | 656 | T | C | 1 | 0 | 0 | 135 | 0.718518 | 0.281481 | GGCACCCAACCAGCTGTATGGCACTCCCGAGGCACCTCCCACAGTCCCCTCCCAATTGTACAACAGTCCCAACAGATTCTAAGTTTACTGAGGATATATTA[T/C]AACACTAAAGAATTTTCATTGCACACATTCGTTAAAGATTGATATTTATTCATTCTACATTCGTAAGAAGATATATATTACAGCTATTGTTGTATATAACC |
| comp106712_c0_seq8:387-388 | comp106712 | 387 | G | A | 78 | 0.820513 | 0.179487 | 39 | 0.102564 | 0.897436 | ACTCTAGATATGTTTAGAGATACATACTATTCCTAACTTGTTGATGCCCATAACTTCATATCAATAGACTTATAATAATAGAAATTGCATTCTCCATCTCC[G/A]CAAATGAGAAAGCTCGTGACATAACCATATATATTTTGAAAAATGATTGTGTTTTGGTGAAAGAACTTGGTGAATGTGACACGATGGCAGATGTTCCTTGT |
| comp104334_c2_seq1:720-721 | comp104334 | 720 | G | C | 133 | 0.917293 | 0.0827068 | 65 | 0.2 | 0.8 | CTTCACCTGCTCATTCCCCTGTACAACTCGGTCGGCTTCGAGGACGACCTTCAAGGACCACATCTGGACCAGTACAAGCGCGCGATGGCTCTGAGGTGGAC[G/C]TGTGGCCTCGGCTATGAGGACTGCGTGGATCGGTCGGTCCTCCAGTTTGAGGAGTGGATTAATAATGGAAGTGACGTGTCCCCGAACCTGAAGTCGACGGT |
| comp106109_c2_seq2:892-893 | comp106109 | 892 | G | A | 81 | 0.802469 | 0.197531 | 70 | 0.0857143 | 0.914286 | GGGATTTTTAAAAATGATTTGAGAAATGCATTTTATGAATATTATCAGAATTTATTTGAAAATCTGTAACCAGAATTTGTTTATTGATCTAGTTATTGCTA[G/A]GTGTTGTTACAAAGTATAAGATTTTTGTATACAGTATAGGAAAAGCTGGCAAATGTTTGTGTACTTATATACGGCGTTATAACAGTTGTGATAAGATAACC |
| comp104656_c1_seq2:433-434 | comp104656 | 433 | G | A | 212 | 0.108491 | 0.891509 | 137 | 0.824818 | 0.175182 | AGATCGTGCCAAGTCATCTGCATTTTCAATGCCGGGAATCCCGACATGGCTGGGTATCCAGCAAAAATTTGTGGCTTTCTGGCGTGTAGACAGGTAGCACA[G/A]CTGGTCTTGTATTTTATGGACTATGGGGTTGGTAGGATGGATGGATTGTATGAGTGATATTGCGTTGCGTGAGTCAGTGAAGATGGTGAAGGATGAGGAAG |
| comp100203_c0_seq28:821-822 | comp100203 | 821 | A | G | 339 | 0.929204 | 0.0707965 | 211 | 0.21327 | 0.78673 | CAAAGGAAATACAGCGCGAAGGAGGCTGAGGCGCAGAATCTGGCGAGAGAAATCAAAGACCTCAATGATAACATCACATCACTGAAGAACCAGCTGGCTCA[A/G]CAGAAAACGACGATGGAAAGGCAGATGGATAACGAGAGAGAGAAACTCGAAAAAGAGATGAAGACTTTAAAGGAGCAACACACGAAGGATCTCGAAGCCGC |
| comp107550_c1_seq16:2866-2867 | comp107550 | 2866 | C | G | 149 | 0.0201342 | 0.979866 | 197 | 0.736041 | 0.263959 | TTTGAGTCTTTATTCCCTTCATACACTTTTGAGTTTTCCTTTCCTCTTTCCGTCAAAAAGAAGAAAGTGGAATACCTCCAGAAGTTTATTCATTTCAACTC[C/G]AGATTGGTATATACATGATTATCCAGCAAACACTATTATAGTTACATGATTTTTTTTCCACTGCTTTCACTATTTAGAGAATGCGCGTGCACTGTTTTTGG |
| comp107810_c0_seq1:1361-1362 | comp107810 | 1361 | A | T | 334 | 0.922156 | 0.0778443 | 189 | 0.206349 | 0.793651 | GCGACGCATCCTTCTGAGTTCATCCTGCAAGATTTAATTAAACTTGCAAAGAAGGGTCTCAATAATGACAAGCTGTATGAGACTCTTGTCCTAACTATAGC[A/T]TCTGTCAGCCACACTTTCTGCAAAGTTTCCAACAATTGCAAAAAGCCTATTATTACTGATGTCCAGGAGTTTATAACAAGTAAGTTGGAAAACTGCAAGAG |
| comp104173_c9_seq4:299-300 | comp104173 | 299 | G | A | 118 | 0.915254 | 0.0847458 | 30 | 0.2 | 0.8 | GTATCCATAAGACTGCAAAGACGTGTGTCTAATTGTCACCCGCATCACCATTATTCACCGCATTTTCTCATCCTTTTCACGTCACCATCATCACCATCATC[G/A]TCTCATTCATTTTCCCTGTATATCTTCCTAGCGAGTGGTGACCCCGCCGTGTTATTGGGTGCTCTTGGTAGCTTGCACGTTACGGTGTCCAGCCCGGCAAC |
| comp107863_c0_seq15:2106-2107 | comp107863 | 2106 | T | G | 244 | 0.872951 | 0.127049 | 322 | 0.158385 | 0.841615 | AACACGACTGGGATAAACTCGACCTTGGCGCTGGCCTTCTTGACCTGGCCCTCGGTGCTGGTGAGGCCGCCCGCAGTGCGCGCCACGCCCACCATGCGGGA[T/G]CCGTCCCCCAGGAGCGGCGCCACGCCCTCGCAGATGGCAGCGCCCAGGCCCCGAGAGGCGCCCGTCACCAGCACCCAGCAGGCGCCCCACGACCCTTCCTT |
| comp98618_c1_seq11:126-127 | comp98618 | 126 | C | T | 41 | 0.951219 | 0.0487805 | 76 | 0.236842 | 0.763158 | AGGTGGGTGGAGATATCCAATGTAATGGTGAAAAATGAATAGAGAGGAAAAAAACATTGAAAGGGGAGGAGGTGCAGGAGAAAAAAGGACAAGATTGAAGT[C/T]GAGAGAAAAAATAGGAATTTTGGGATTAGGGAAAGGATAAAGAGGATGTTGGTGTTGTTTTGCCATATTTTTTTCTCTATGATAATAATGTTATGATACTG |
| comp102840_c0_seq5:217-218 | comp102840 | 217 | A | T | 82 | 0.768293 | 0.231707 | 37 | 0.0540541 | 0.945946 | GGCGGGGCGGCTGTTTCGAGGAGGGACACCTTGATCAAGCCCATCAAGGTGGAGGCAGAGGGATTTCCCCGCGAGGAGACGTGGACCAAGTACCTCTGCGC[A/T]GAAGAGGTGGCATCAGGCGAGGACGCTCCGGAGACGTGGGAAATAGTGCCACCTGCCAACATAGTGCCAGACTCCGCCCGCGGTTGGGTCACTGCCGTCGG |
| comp98859_c0_seq4:619-620 | comp98859 | 619 | C | G | 51 | 0.941176 | 0.0588235 | 22 | 0.227273 | 0.772727 | CCGTGTACGCCCGCATGTTCACGGACGTCCACGATCGGGCGGGGGCGGCGGTGTTGCCCCTCGTGCTGCGTCAGGAGGCCGATTCCTCCGATGAGAGCTTC[C/G]TACTTCGTGCGGAGGATCTGGAAGCGCGGGTCGAGGCCCTGCATCAGGAGGGGCGTCGCGTGAGGGCCTTCATGCTGGTGCATCCCAACAACCCTCTCGGC |
| comp101278_c1_seq1:2524-2525 | comp101278 | 2524 | G | A | 69 | 0.797101 | 0.202899 | 36 | 0.0833333 | 0.916667 | CAGATGGTTGACCTTGCCGATTGCATTCTTACTGGATACCAGCCACAACTTCGCTCTCTTGTATCTGTTAGCCACGACTTGCACCAGTCCCTTCTGCGCTC[G/A]TATGAGAGGGACCGCTACAATTTGATTCAACCTCTTGTACAAGGTGAGCAGTACGATCAAGCAGTTGGACTTGCTGAGAAGTACTGTGACTTCCGCACGCT |
| comp105357_c0_seq13:178-179 | comp105357 | 178 | A | G | 150 | 0.88 | 0.12 | 150 | 0.166667 | 0.833333 | AGTGGTGTACAAGGCATCATTGTATGTGGAGAAGCCTCAGAATCAGAAGAGGAGGAGGAATTTTGTGCGGCAACGGCAAGAGGACACAAAGGTTTGACTCC[A/G]AAATCAGGAGGATCCACAGACACAAAGGGTCAGCTCAAGCCCCCTGAACCAAAGCACACACAAAAGCAACAGCGACCTCAACAACAGCAGAAGAAAGGCCA |
| comp93856_c0_seq1:2286-2287 | comp93856 | 2286 | A | T | 102 | 0.852941 | 0.147059 | 93 | 0.139785 | 0.860215 | ATATATGCATATATATTTTTATGTAGACGAAACAGCATCGTTGAGAAATATCTTGTTTCTTTGTTGTAAAGTTCAAGATCTTAATTGTCATGTATCAAGAA[A/T]GATATGAGAATGGCAGCAAAAGATAATGGTAAAAAGGAAGGAAAAACTATCTTTTGCCATGGTGGTTGTTATTCATTTCAATAATTTTGAAGGTACGTTTG |
| comp97028_c0_seq1:198-199 | comp97028 | 198 | G | T | 15 | 0.933333 | 0.0666667 | 59 | 0.220339 | 0.779661 | GGATACAAAGAGAAAATGAGAAATAGAAAAGAAGAGAACAGAAGGTGAGGTAAACGAGAAAAGTGAATGAAAGAAAATAGAGAGAAAGAATGGGTGAACAT[G/T]GTTCATCTTTAGTATCCAATGATTTATAAATCTCTTTCTCGAACTTGACAGAAAAAAGTGAATCTCTGGACAAAAACAAGATTTTTTACTAGAATGGTTGT |
| comp96813_c0_seq1:1256-1257 | comp96813 | 1256 | C | G | 53 | 0.886792 | 0.113208 | 23 | 0.173913 | 0.826087 | GTTTTACTCAACTTTCTTTTCCCCCAGAGGAAGCTGTTTCCCTTATGATACAAAAGAAAATAACTTAAAATTTATTACTGACAAATGATTACGTTAAACAA[C/G]AGAGAGAAAAGAAAAGATAAGAAAATTAACTGGAAACCTGATAATTCATAGATATAGGACATGAAGCAAGCTGTGCAGTACATACAATTTTCCTAATACTT |
| comp96425_c1_seq3:408-409 | comp96425 | 408 | G | A | 94 | 0.712766 | 0.287234 | 2 | 0 | 0 | AAGTCGAAGCCAAAGTTTGGTCCAGTGTTGAGGATGGGTCCAAAATCTGCTCCAGTGTTGAGGGTAGGTCCAAAATCTGCCCCTGGTATGCCTACAGGATT[G/A]AAATTGCCTTGTGGGTTGGTGATGGCTAAGGGCACACCAACATTGCCAGGATGAACTGGTAACCCAACATTAGAAACACTGAAAGCATTACCGCCTGCTGT |
| comp95723_c0_seq1:146-147 | comp95723 | 146 | G,T | C | 33 | 0.121212 | 0.878788 | 908 | 0.8337 | 0.1663 | CGCGAGTTGCATCAGTTGCAGCGCAATTGCTTCAGGGTTTCAGGATGTTCCGTTTGCTTGTTCTCGCTCTGGTTGTGGCCGTGTGAGTATTTTGTTTTCAA[G,T/C]TGAATCGCTATTATTGTTATTCTTGAACATCAATTTCATTATTCCATTAGGATAAGAATATTTATTTCCTTATTCTTTCCTTGGTATAAATATCGTGTATA |
| comp94039_c0_seq5:237-238 | comp94039 | 237 | C | T | 52 | 0.711538 | 0.288462 | 1 | 0 | 0 | TTCGCCCGCCCCGACGTCCTGGACTTCGAGGGCGACGACCACGAGCACGAGCAGGAGGGAGAACCAGGGAACTCCGTCGAGGGAACGTACAGCTGGACGTC[C/T]CCCGAAGGCGAGGAGTTCTTCGTGAAGTACGTGGCTGACGAGGACGGCTACCGCGTGGTGGAGTCCAACGCCGTCCCTCGCACTCACGACGGCGTGGCAGC |
| comp97337_c0_seq1:1542-1543 | comp97337 | 1542 | G | A | 116 | 0.75 | 0.25 | 129 | 0.0387597 | 0.96124 | GGAGAAGCTTCTACCCCCCCCCCCAACCCCACTCGTGCTCGCCCAGGAATCTCTGTCTTTGAACCGCTTCGTCGTGCTGTGCGAAGCCGGTGTGGTCGTAG[G/A]CTTTGGGACTCTCTCCCGAGGAGTTCTCACTTGACGTCGCTGGCTTTTCTGTATTGAGAAATATTCTTTTTTATTCGCCTGGTAGAGATTCCAATCGCTGC |
| comp103951_c2_seq1:368-369 | comp103951 | 368 | C | T | 63 | 0.777778 | 0.222222 | 45 | 0.0666667 | 0.933333 | TCACTCACTTTACTCATTGCAAACACACGCAAGCAAACACATACTTGCTATTACATAAGTACTTATTGATTCACTCATATAGTTGTGTGTGTGTGTGTGTG[C/T]AATAGATTATTCATGGATACCAAGGCAATTGATAAGATTTGGACATACTACCTTTCATGAAAACTTGTTATTCAGGCTTTAAAATGAGGATGAAAGATGGG |
| comp100577_c0_seq8:407-408 | comp100577 | 407 | G | A | 156 | 0.935897 | 0.0641026 | 40 | 0.225 | 0.775 | AAGCAATTGCAGGAGATGAAAAGGAAAAATAACGCTAACGGGGCGGAAACACAAGAAATGGAGAAGGAAAACGGGGATTTAAAGGAATTCATCAGATCGCT[G/A]CAGGAACAACACAAAGCGGAACTAGCAGCAGCGAAAGTCGATGCTGACAAGATAAAAGATTATATTAATAGATTGAAGGGGAAACACCAGGAGGAAATACG |
| comp106363_c0_seq3:530-531 | comp106363 | 530 | A | C | 181 | 0.895028 | 0.104972 | 184 | 0.184783 | 0.815217 | AATGAAAACATTTCTGGACACAATACACCATGAAATTCCTTTCCTTAAAATTTCCTTCACGTTGCAAAATAAAAAATAAAAACACCAAGAGAAACAGAATA[A/C]ACCTGCATCTCAAAATTTGATCAAATAAAACCAAAATCAACAAAAAATGACCAATGCCTTCTTTCTTATGATAATAATTCATACACCGGGTATATCCATTC |
| comp104878_c0_seq1:3148-3149 | comp104878 | 3148 | T | G | 278 | 0.723022 | 0.276978 | 154 | 0.012987 | 0.987013 | TGGCTGCATGGGCGTTTGGAGAGGAGATGCGATAGATGCTGGATTTGGGATGGGAGTTTCAACTGGTGCTTGGACTGGAGCATATGATGTAGTGGCAATAG[T/G]GGCCTGAGAATAAGGTGTAGGAGCTGGAACAACTGGAGAAGTGACACTTGCTGGCAGCTCAGCAGGGGGGGGAATAGGAGTTGGAGTGAATGGTGAAGGAG |
| comp105607_c0_seq1:1828-1829 | comp105607 | 1828 | T | C | 57 | 0.754386 | 0.245614 | 45 | 0.0444444 | 0.955556 | TCCTTGACAAGGGAAATCGGCTCCTTCTCAAATATCCAAATGAATAACTCTTCCGGTGATCAATTAGACGAGCACGATGCGAATTCTTCGGTCGTAGAAGA[T/C]GAGGAACCAGATCGAGGAGCAGTGAATGATGATACGGAGAACAAAGACACCGAAGTTGATTTGTTAGAAAACCAAGAGCCCAGTGATCCTGATGAAGACTT |
| comp107126_c0_seq11:3465-3466 | comp107126 | 3465 | A | C | 62 | 0.806452 | 0.193548 | 176 | 0.0965909 | 0.903409 | GACTCTGAGGACCTCGTTGTCTCCTTGCAGCCCATGGAGATCAGGACCTTTATTCTCACAGTCAAGAAACTTTCATCTTAATTCCAGGGGAGTGTTCTCTC[A/C]TTTTCTCTATGTAAAATTAAAGTGTTTCCTTCCAATATCCGGTTATCACAGTGATTTGCTTTCTATTTTCATTAATATTATTATCGTTGTGATTTGATTCT |
| comp102186_c0_seq1:1187-1188 | comp102186 | 1187 | A | G | 547 | 0.753199 | 0.246801 | 296 | 0.0439189 | 0.956081 | GAGGTGCTCACTAGCATGCGGCGTGCAGGATCTGATGTGATCATTTCATACTTCACACCAAAGGTGCTGGAATGGCTCAAGAAAGAGAAAAACAAATCATA[A/G]AATAATTTCTCCTGATTTTGTTGTACTTTGTCCCAGACTTGTAAGTGGTCGTAGGCACACCAAAAATCTGAATTCCATTTACATGACAGATGATGATATTT |
| comp103224_c2_seq11:1470-1471 | comp103224 | 1470 | A | T | 2711 | 0.802656 | 0.197344 | 214 | 0.0934579 | 0.906542 | CATTTGAAGGCCATATGCCATGAGCTTTTAAAAGTCCCTTCGAAACTGAGACGTGATTGGTCGGAGATGAAAAGGTCGTTACGAAGCATTGATTCAAATGC[A/T]GATCTTGACCCTGATTGGTCCGAAGGTATCCAGACCTCACCTGATTGGTCAGAGTTCCAAAGTTCCCCTTCGCCATCCCCGTCCATTCAGCTCAAGACCAG |
| comp104179_c0_seq1:244-245 | comp104179 | 244 | G | A | 71 | 0.788732 | 0.211268 | 25 | 0.08 | 0.92 | TTTAACTGATATACGTAAAATTAAATTTTTATGATTCTATTTTTTGAGATTTATTTGTTTACAACTGTAATATAATCAAGAATTATTTCACGAAGATTCCA[G/A]TTTTCTTTCATCCTTTTTTACGAAAGTGTAGACGAGGTTTGACTATATAATGAGGTCGTTCACTTGCGGAGCCCCAGTTGTTTGTGGACCAGGTCGGTGCT |
| comp107862_c0_seq1:4000-4001 | comp107862 | 4000 | G | A | 128 | 0.851562 | 0.148438 | 77 | 0.142857 | 0.857143 | TCGCGAGGAATAAACTTGGTGTATTCCTCCATGGATGTCCACTGCCCCATGGCCCACGCTGATGCTGCTGCCACTCGGGCCATCTGTGCGCGGCACTCATC[G/A]TTCATGGAACTGCTCCACCACTCGCAGGATACACTGTACAGCTCACCCCATTCTCCAAGGGCCTCCAGACACCTCATCTGACCCAGAGTGAGTTCTAGGTC |
| comp107135_c0_seq15:409-410 | comp107135 | 409 | G | A | 50 | 0.2 | 0.8 | 120 | 0.908333 | 0.0916667 | TGTTCTTACTGAGGTCGAGGACATCGTTTTTATTCCAAAATCTGTTTATATAATGAGAGTAGTTATGACATCGACAGAAGTGTAAGGATAATGACTGTTCA[G/A]AGATTGTATTCACAGTGTTATCCTAGTTATAATATTGGCAAAGTATCATTCGGATGTGTATTGTATAAATGAATAGGGGCTCATGTAGACTGTAGAGGACT |
| comp93811_c0_seq2:195-196 | comp93811 | 195 | G | A | 642 | 0.207165 | 0.792835 | 71 | 0.915493 | 0.084507 | CAGTTTTGGGCTAATTTACTTAGAGCGTAAAAAGAATCCTCGTTTTCACCAGTGGTGGTAGTAGCGATGGTGGGGGTAGTGGTGGTAGTGTGGGTAGTAGT[G/A]CCTGCGTCTAAAGAACCAGGGGTCGGCCACGGGGTCAGGGTCGGCCACGGGGTCAGCCACTGGGTAGGGGTCAGGCATGGCACGAGCTGAACCAAGGAGGC |
| comp98486_c2_seq1:202-203 | comp98486 | 202 | G | A | 54 | 0.907407 | 0.0925926 | 25 | 0.2 | 0.8 | AAGAGTAACGCACACTTAGAAATAAACACGATACGACTCTTGGTGCTGGTGGAAGCCGAGGCCAGCGAGCCCAAGTAGGCCTATGGGTGCGGCCTCGTGGA[G/A]CGGTGATCGTCAGGTCTGTGTGAGCGAATAAGTGAGGGTGAGCGAGACCCACCCTGCTGAGGGCAGGCTCGTCGAGGGTATGAGAACTTGGGTCAAGGCCG |
| comp107454_c1_seq3:260-261 | comp107454 | 260 | C | T | 91 | 0.769231 | 0.230769 | 96 | 0.0625 | 0.9375 | GCCGAGTGATTGACCAGAGAAAAGCATTCTGATTCTCGAGCTTGGAAGAGCTTGGAAGAGCTTGGAACTGGCCTTTGGTGAGTCTCCTGCTGCTGTGTGAC[C/T]GACTGGGATTTCGAGTTCCTGCCACACCTCCCTCCCTCCGCTCCCATTACTTGGCTCCGGAGTAAATGACTGCCAGCTGTTCCATCTCTTGAAGAAGGGGC |
| comp105843_c0_seq2:2592-2593 | comp105843 | 2592 | G | A | 38 | 0.815789 | 0.184211 | 55 | 0.109091 | 0.890909 | AAACAATGAAGAATTGGTGACCCAGTGCAGGTATATGACTATTACACCAGGTCTGCATACACACTAGTAGTTCAGAGTATGTAATATGCTATTAGTGGAAG[G/A]CAAGCAAAGAAGTATATATATTGTATACCAGGGTATTTGTTTAGTTTGACATATATGGTGTTGTGCCTGGTATTGTTTAGCAGACTGTGAGAATGAGGAAG |
| comp106401_c0_seq10:3821-3822 | comp106401 | 3821 | A | G | 936 | 0.74359 | 0.25641 | 1221 | 0.037674 | 0.962326 | GAAGAAAACAGTTTTTCTACCATTTGCCTAATGGTACTGTGCACTGACATAATGTTGTTTCACAGAAATAATTGTGAAATTGTTTTCTTGCAATTTTCCAT[A/G]ATAGTGTGTGAAATGATATGCATGATGGAATGACTATAAATGTGATTTTTATTCTATAAAATTTATGCAAAATTTTTATATTTATGCTAACAGGGTGATGA |
| comp102166_c0_seq2:1235-1236 | comp102166 | 1235 | G | T | 47 | 0.12766 | 0.87234 | 186 | 0.833333 | 0.166667 | TAGACGAAAAAATATATAAATGAAGGCATGAAACTACATTTTCTTTGGAACAGACAAAAAGATATATAGATAAATGAAGGCGTGAAACTACAATTTTTTTT[G/T]GGAGTCGACTAAAACACAAATGACGGGCGTGTGAGGATAAAACCCACAGACAACCACAAACACTCGGAAAAAATAGTTTGACGAGTGATTTCGTATTGTTA |
| comp104728_c0_seq2:594-595 | comp104728 | 594 | T | C | 97 | 0.226804 | 0.773196 | 74 | 0.932432 | 0.0675676 | ACACTTAAAGTCCTTGCGAATACTTAAAATTTCTTGTTTACTTAAAATTCCTTATGCATCTTTCAAATTCCTTGTGCATACTTAAAATGCTTTGTGTATAC[T/C]TGAAATTTCTTATGTATGTTTCACATTCCTTATGCATTCTTAAAATTCCTGGTATATACTTAAAAGGCCTTGTGTATGCTTAAAATTCTTTATGCAGACTT |
| comp98612_c0_seq11:169-170 | comp98612 | 169 | T | C | 84 | 0.857143 | 0.142857 | 33 | 0.151515 | 0.848485 | CTATCCGCGTGGGGGGGAGTAGCTGTTGGACACGCCAGCACTTCCCGCTCCACTTCCGAAGGAGCTTCCAGCACCGAAGGAGCTTCCACCACCGAAGGAGC[T/C]TCCAGATGCTCCTCCAAAGCCGGAAGAGGAGACTCCGATGCCATGTCCTCCTGCAGAGCCTCCGGAAGCTCCAAAGTTAGTACTTGGGGCACTGTAGCTTG |
| comp92114_c0_seq1:217-218 | comp92114 | 217 | C | T | 117 | 0.717949 | 0.282051 | 150 | 0.0133333 | 0.986667 | AACATTGAGTACCCTTTAGAAATTTCTTGGAGATCTCGGTGTTTGCACTTGGCATCCGGGGGATACAACCTCTCTGCTTAGTAAGAACATCCTAGTCTCCT[C/T]GACTTGTATAGTGAGCACCAGGTGCTAAGGGGACATACATATACAAATACTCATATACATATAAACTATTCATATATTTATTTAATGTGTGTGTGTATTGA |
| comp99281_c0_seq1:588-589 | comp99281 | 588 | A | C | 59 | 0.881356 | 0.118644 | 96 | 0.177083 | 0.822917 | TGACTTCCTTCTGCAACACGGTCAACAGGTCGGGTGTTGCAGCGCTGCACCACGAAGTTCCTGAGGTCGAAGGCAGCGTCCGAATGGAGGCGCGCCGCCCG[A/C]CTCATCCTCACCCTCCTCATGCCTCTCCTCCCCCCCCTCCTCCTCCTTCAACCCCCGCTTCAGTCGAGGAGTCTCGCATACTCAAAGCTCTGAGGAATGTG |
| comp98155_c0_seq1:713-714 | comp98155 | 713 | T | C | 97 | 0.907216 | 0.0927835 | 54 | 0.203704 | 0.796296 | TGCTCCAAAGGGCGCTGCGCTCCTCCCCCGCAGGACATTCCCGTGGAGAGGATCATCAGGCACCCCCAGTACGGCTCCCCCTGCACGGAATGCAACGACAT[T/C]GCCCTCCTGCGCCTCAGTCGCCCCGCTGTCCTGCACCCGCGCTTCGTGGCGCCCGTGTGTCTGCCGACCAACCCCGTCCAGGACATGGGCTTCTCTGAGCA |
| comp106818_c3_seq19:2551-2552 | comp106818 | 2551 | C | A | 72 | 0.930556 | 0.0694444 | 110 | 0.227273 | 0.772727 | ACAGAAAGATAAATAAAAGTGTTAGAGAGAGAAAAATGCTCCATATTTTTGTAGAGAGGTAGAGAGAAGAAATTTATAAATAATTAATATGGTATAGCAAT[C/A]ACATCACTGCCATCAGATGTTTTGAACATTTTTATTCTTTCATTGCTAATGGCAGGAGAAAAAAACAAAGGAAATTTTAGTATTATTATTATTGTCATATA |
| comp104099_c0_seq7:1244-1245 | comp104099 | 1244 | G | A | 2633 | 0.799088 | 0.200912 | 2100 | 0.0961905 | 0.90381 | CATTTTATGGACAATCACTACAAGACTGCCCCTCTGGAGAAGAACATTCCTGTATTGCTTGCACTTCTTGGTGTGTGGTACCATAATTTCTATGGTGCTGA[G/A]ACCCATGCCTTGCTGCCTTATGACCAGTACCTGCATAGGTTTGCTGCCTATTTCCAGCAGGGTGACATGGAATCCAACGGAAAGTATGTGACTCGCAGTGG |
| comp105950_c0_seq1:169-170 | comp105950 | 169 | A | C | 117 | 0.888889 | 0.111111 | 43 | 0.186047 | 0.813953 | AAAGAGGAAGGAGGCAACTGCATTTCTTGACGAGCATTAGAACGAAGCCATCCAGCCACGATGTCGGAGGAAGTTACCGTGAAAGTCCTGAACGAAACGGG[A/C]GAAGGCAGCCATGCGCAGGACCTCGACTCCGTGGCTGTGTCCCAACGCCTGCAAAAGGCTATGTTGAAGATGACGGGGAAGTTTTTGGACGAAACGACAGG |
| comp103924_c5_seq1:1169-1170 | comp103924 | 1169 | C | T | 74 | 0.783784 | 0.216216 | 37 | 0.0810811 | 0.918919 | GACAAGACAAAGAGAAAGACAGCAAATGTAACACGAGATAGAGAAAGGTTGAGAACAATAAAGTGAGAAAGAATGGGACAAAGGAAGAAGGAGAGAGAGTC[C/T]GAGGATAAAAGACAGAGATGATTGCACCTAGATTTCAAATGGTTATGACCTCAATGCAATGGTAATAATTAACAATTGAAAGGTGATGTGCTTCCTTCACC |
| comp107007_c0_seq12:1009-1010 | comp107007 | 1009 | G | A | 44 | 0.0681818 | 0.931818 | 205 | 0.770732 | 0.229268 | GAGAGAAAGAGAAGGAGAGAAAGATAGAGACAGTTGAAAACAAGGGAATAGGGGAGGAAAGGAGAGAGAGAAAGGTTGGTTACAAGTTGGCAGACAGACAG[G/A]CAGACAGACAAACAGACAGACAGACAGACAGGCAGGCAGACAGACAGACAGACAGATAGATAGATAGATAGATGTATATATGTATATACAAACGTACATAT |
| comp99812_c4_seq1:2236-2237 | comp99812 | 2236 | T | C | 1739 | 0.768258 | 0.231742 | 2446 | 0.0658218 | 0.934178 | ACATTAGGTTTAACTGTATTAATAAGCGTCATCGTTTCCAGTCTGTAGATTCTTCCTCTCAAAGTGTGTCCTGTTCAGCGACGGACTTTGATCCTTATTGC[T/C]TGCCTCATTTTCTTATCTGGTTGATTCTGTCTATTTAATTATTCTCTGAGTTATCGCATTTATGTTTAATAACACTAACCTTATAAAATTAGGATTTCTTT |
| comp105922_c0_seq4:1335-1336 | comp105922 | 1335 | G | A | 122 | 0.868852 | 0.131148 | 18 | 0.166667 | 0.833333 | GTCTGTATGACTCGTTCCAATGATTGTCTCGTTCCAAGAGGCGTAAAAAGACCTGCGATGAATCCTACATACTGCAAATGCCTTGTTACTAGAGGAACGCA[G/A]AGAGGTGGAGATAGACCTATATAACTGTCTCGTTTCTAGAAACTTAAGAAGACGATAGGCCTGTATGACTGTCGTTCCTAGAGACGTAAATGACCTGTGAC |
| comp98508_c1_seq1:261-262 | comp98508 | 261 | A | G | 349 | 0.868195 | 0.131805 | 331 | 0.166163 | 0.833837 | GAATCCTCCCCCGTGGATCCAAACCATTACGGGAAGTCTCTCGGCAGACTCCATACTTTTCGGCGTGAATACATTGAGGTAGAGGCAGTCCTCGTCACCGA[A/G]CAGCTCCTCTGGGGTCAAAGTGATGCCCATGACGGGGAAGTCGAAGGGCACCTGGAGGCACGGCGGGGGCATCTTGGACCCATCCCTCACGCCCTCCCAGC |
| comp102593_c0_seq27:259-260 | comp102593 | 259 | A | T | 80 | 0.775 | 0.225 | 137 | 0.0729927 | 0.927007 | GATCCAGATAAGCTTATTAGGACTCTATCAAAAAGACTTTAATGTTCTTAAGTGAAGATGAGGGCCTCAAGATCATTTTATTCCATTCCATGCAAAGACAC[A/T]AAACAAAACAAATTTGAAAGTATCATGAGCTTGCTGGCATTTTATGCAGAAGTGAAAATGGGCAAATTTGTATGCCAAATGATCACATTATGCAGTTACAA |
| comp99491_c0_seq1:1383-1384 | comp99491 | 1383 | T | C | 51 | 0.901961 | 0.0980392 | 10 | 0.2 | 0.8 | GTCAAGTAGGAATTTATTCTATTTTTTATTGGCTGTGTATTAGATGAACCTGTGGAAGCATATATTCACTAAAAGATAATCCAAATTCATACAACCCTCAG[T/C]AACATAAAAATGATTATTATGTATAGTTATGTTTTGAAGTGCATGTCAACATCTCTCTTTTATATATCTATGTCTGTCTATATATGTATCATAGTTCTATT |
| comp107195_c0_seq70:1681-1682 | comp107195 | 1681 | G | A | 65 | 0.815385 | 0.184615 | 44 | 0.113636 | 0.886364 | TACAGCTAACTGATGTGGCAGGTCGTCTGTGCATCGAAGCTACCTGGTACGACCTGATAAGTGAAAATATACCCCTTGAAACGGTGGAGGAACCCTGTCAC[G/A]CGGCTGTTCTCGTGGTGTGGGTGGACTCCGTCATAGGTCTCGTTGATAAACCTAACACAATGGTGCGGCTTCGTGTCGCCGACGGACACCAGGAGACGACG |
| comp105857_c1_seq2:658-659 | comp105857 | 658 | T | A | 70 | 0.871429 | 0.128571 | 53 | 0.169811 | 0.830189 | TACTGCCTGGTGCACCTGGGGGACTTGGCCAGGTATCGGCACCAAGCTAAGCAGGCTGAGACTTATTATAGACATGCCGTTGTGGTTGCTCCCACAAGCGG[T/A]CACCCATATAATCAGCTGGCTTTATTAGAAGCTGGCAGAGGGAACCGTCTTGCTGCCGTGGCTCTCTACGTCAGGGCCATGTGTGTGCCTTGTCCCTTTCC |
| comp100865_c1_seq16:530-531 | comp100865 | 530 | A | G | 407 | 0.896806 | 0.103194 | 169 | 0.195266 | 0.804734 | ACCTTGGCCGCGGTGTCGACGTCGACCTGATTCCTCATCGTCCACACGATTTCCTGCGTCCACGTCGTCCCACTCCTCGGGAACGTCATCACAAGCACGTC[A/G]TCCTTCTGGAATTTATAATTGAAGTACCGCTCGGCAAAGCGGTCGTAGTGCGCGGGCATGGCGTGGCGCCCCTCGACGTCCACTAAGCCGCCCCTGAACCC |
| comp107511_c0_seq13:683-684 | comp107511 | 683 | C | G | 59 | 0.830508 | 0.169492 | 155 | 0.129032 | 0.870968 | TACGTGTACACCACCAGGCGGTTCTTCCCGACGTCCACGTCGACCACCACGTCCGCCCCGACATACCGCACCACGACGTGGCCGCCCTACAGGATGTACAC[C/G]ACGTCAACAACAAGTGCCCCGACTACCACGACGCAACGGCCAACCACCACAACACCGATGACGACGTCTACAACATCGACCACATATTCGCCAACATCCAC |
| comp103357_c1_seq13:4071-4072 | comp103357 | 4071 | T | A | 71 | 0.901408 | 0.0985916 | 5 | 0.2 | 0.8 | CTCCTTCCTCCCCCTCATCCTAATTTGTGCCTCAGCCTTCTCCTCTCCCCCCCTCATCTTCCCCTTCCCTTTAACTCAGATCCATCCCGACTATATTCCTA[T/A]TTTCCTAAAATTCCCTTCTCCCCTCTTCCTTCCTTCGTTTTCCCTTCCCTTCCTTCTTCTCTCTCTTCGTCCCCATCCTCTGGCATTTTCCCCTTCGTCTG |
| comp97222_c8_seq1:221-222 | comp97222 | 221 | A | G | 21 | 0.904762 | 0.0952381 | 59 | 0.20339 | 0.79661 | ACATCAGGGAGCAAAGTCTTTCTTGCTGTATATAATATTTTCTTATTTTCTTGCTCCATACCATAACACTTAAAAGATAAAAGGTTGGACGCATGTTAAAA[A/G]TAAATCCAAACACCTTTATTAATTTACTCAGTTATCAAATGGAGTAAAATTTCCCTCAAATAATGAATGAGATATGAAATTTTTGTGAACATCTGCACCAA |
| comp94910_c0_seq3:240-241 | comp94910 | 240 | A | T | 132 | 0.219697 | 0.780303 | 63 | 0.920635 | 0.0793651 | AATTGCAAAGCGAATCTCTTTTCAAAGTGTAGCTGATGCAAGTTGCAGATGAGAATGAGTTGAACAAGATCCAACGGAATTTGGAGTGAGTGACGTCACAC[A/T]AATACTCACGGCGGCGGCGACGAAGGCGAGGAGGAGGGCGACTCTGGCGACCATGGTGGAGGTGCTGCTGAGGTGCTGTTGCATCGCCACCGGTTATATAC |
| comp102550_c1_seq1:641-642 | comp102550 | 641 | G | A | 465 | 0.204301 | 0.795699 | 84 | 0.904762 | 0.0952381 | TCTACTAACGTAAATAAGCTAACCAACATTATCCTTCATGAAGATAACTTGGTACTTAATATACTAATGAGTTGTGGCTTTCATACCATAACTTAGAGTAA[G/A]AATGTATAAAAAATCATGATAATTGCTTAAAACATATCAGTGTCCACTCCTATAATTGTTATTTTTGCATTATGCTCTGTTTTTGTGTTTTAACTATTATT |
| comp103406_c1_seq1:644-645 | comp103406 | 644 | C | T | 29 | 0.241379 | 0.758621 | 51 | 0.941176 | 0.0588235 | AAGAAGAAATGGATCCGACACAGTTTTTCTCGACTTTTATCTCTTATTCCTAGCTTTTGTTTACATGTATATATATTTACACAGACACATTCTTGGACATA[C/T]GAACACAAATTCCCATTTATATCAGTCTTTTTTTTTTACATTCGTATCACATACCACTACATATATTTGTGATATTGAAATTGTTTTTTCTATTAGGTACA |
| comp104137_c1_seq12:924-925 | comp104137 | 924 | G | A | 162 | 0.845679 | 0.154321 | 178 | 0.146067 | 0.853933 | ACGTGGAGTTCGTAAAGCCTGGTGGAGGTCAGTTGAGGAACTTCCGACAAGCTGTTCCCGTACAACATCACTTTAGAGGCAGTGTCGCTCGAAGCCCCGAG[G/A]AAATCGTCCACTTGCACGAGGTTTGGACAAGAAACTATCGAAATCTGCCTGAATTGGAGTTCGCCGAGGAAGCCGACCGTCAGACGCTGAATGAGGGCGTT |
| comp106832_c0_seq1:154-155 | comp106832 | 154 | G | A | 121 | 0.867769 | 0.132231 | 101 | 0.168317 | 0.831683 | TGAAGTCCTGGCATCTTGTCGGTGTTGCTGTGTTGTGCCTGGCGGCGGCAGTGGAGCCGCGAAGTGTGGACGTAGGTCGGGTGAGGTCACGCACTCGCCCC[G/A]AGCCCCCGGGCGGGTATCGAGCCGTGCGAGTGCAGTCTGCTCCCACTGAGACTCCTGTTTCGCCGCCTCCTGCCGAAACCCTCCTCAAGCCCGGCTTCAAG |
| comp104616_c0_seq2:381-382 | comp104616 | 381 | A | C | 70 | 0.928571 | 0.0714286 | 144 | 0.229167 | 0.770833 | AGAGAGGCAGTGAAAGCTTCCCGACGCGCTCCCGCCAGCCGAGGGCGCCCGCACGACCGCCATGACCTTCTCGGGCGGCAGCGCTCTGTCTCTCCTCGAAA[A/C]TCTCGACTCCTCCGGCACAAACGGAGGTCAAGGTGGAGCTGCAGGAGGAGGAGGAATTGGAAGTGATGGGGGAGGAGGGGCCAATAATGCAGGATATAAGT |
| comp101978_c0_seq1:787-788 | comp101978 | 787 | T | C | 143 | 0.699301 | 0.300699 | 1 | 0 | 0 | AACCTGGCTCACGTTCTTGGCGTAGGGGGACCCAGCGACTTCCGAAGCGATGACAGATTCAGCAGAAATCCGTCAATTTTCTCAGAACGAAAGGGCGTCCG[T/C]GAAGACGATCTGTCAACGCTTCTGCAGAGACTGAACGCCTACCCTGATCCCACCCGCTCAGTGCGCTTCCCGCGCGGGGAGAGCACCCACGGCCTCCTGTC |
| comp101660_c0_seq1:1139-1140 | comp101660 | 1139 | A | G | 79 | 0.810127 | 0.189873 | 81 | 0.111111 | 0.888889 | ATACAATTGTTTACCCTTTCTTCTAATCTTGTTTATCTTATGAATATCTCACAACTACCTCTCCCCTAAATGACTAAAACACTTATCGAGATTTATATCCA[A/G]AGGAGATGGTACTACCTACATTCATTCTTGATAGATACACATAAGCTCTAGAACTCAAGAAACAAAGTTACAAATCCACAAACATGATGTTTATTCCTGCT |
| comp101614_c0_seq1:347-348 | comp101614 | 347 | T | C | 128 | 0.726562 | 0.273438 | 36 | 0.0277778 | 0.972222 | GTTTCCATGAGCCTCTTAAACTCGGCAGCTGCGTGACGGTCCCGTCTGCTTCGCGAATGTACGACACAAAAGTCCCACACGGTATGTCTGACATCCCGACG[T/C]CGCTGATCCATCCAGTGTTCTATCTGTTCATAGAGTGTGATGACAAAATTCCTCCAGGCGGTCGCATATCTCCCTGCAGGAAGGTCGACTCGTAATTCCCT |
| comp104979_c0_seq1:601-602 | comp104979 | 601 | C | T | 63 | 0.809524 | 0.190476 | 72 | 0.111111 | 0.888889 | TCCCGATACAGTTGAAACGCTTGACTTGATGCTCGTCCCGACAACCCGGAGGTTCAAGCCTGAGGGAATGTCTGTGATCTGGGAGGGTTCTGAACTCCCTG[C/T]AGATTCTATTTTAGAAGAGCTTGCTGACTGTGTGTTGAATGAGGATGAAGATGAAGAGAATGAATTAGATGTTCTTTCCTGAGAGCTGGCGTTTTCATCTT |
| comp107042_c3_seq6:892-893 | comp107042 | 892 | A | G | 49 | 0.897959 | 0.102041 | 60 | 0.2 | 0.8 | GCCATAGAAGCTGGAGTTGACGTCGTCGTTGTGAATGACGTAGTTGAGCTGGCAGTTGACGAAGACGTGACGTCACAATCGACCTCATCACTCCCGTCAGC[A/G]CAATCTTTGTCTCCGTCGCAGATATACCACCGACGGACACACTGATTACCCGAAGAACAACGAAATTCGCTCTGCTGGCATTCCTTGGCCGATTTCGGTGA |
| comp107612_c1_seq3:1874-1875 | comp107612 | 1874 | C | A | 54 | 0.777778 | 0.222222 | 25 | 0.08 | 0.92 | TTTTGTTTGTATTACTTCAACCATACATCCAAGCTTGGGGGAAAAAAAATAAATAAAAAAAATCAGAACATCCTTTTCTAGAGAATCACTAAACATGCTGG[C/A]CTTAACACTCATACTTGTATCAACACAGCCAGCATAGCAAGTAAAAAATAAAATCATTCATATTCAAAAATCATATCAAAACAGCTAGGCACGCACACAAA |
| comp105658_c0_seq4:695-696 | comp105658 | 695 | A | G | 59 | 0.864407 | 0.135593 | 54 | 0.166667 | 0.833333 | GCAGGATTAGAGTATTCAACAGGATGCAAGGCAGAGGCCGTGGGAAAGCCAGAGGCAACATTTTTCAAGAGTGCTCTCGAGGACTTAGGCTGCGAGCCGGC[A/G]GAAGCTGTGATGATTGGAGATGATGCTCGCGATGATGTGGGCGGAGCCATGCAGACCGGCCTTCATGGGATCCTGGTCAAAACGGGGAAGTATAGGGCTGG |
| comp107123_c0_seq3:544-545 | comp107123 | 544 | C | T | 92 | 0.923913 | 0.076087 | 84 | 0.22619 | 0.77381 | GCCGCCTGGTGCCAGCAGCGGCCGACGTGGAGGCTGTCCCGCCGCCGCCCCTCCCTCCCCTCCTACAGGGACTACCTCAGGAAATACGCGGCCAACCAGAC[C/T]GCTGTGGCCGGAGGAGCGCACTTCCGCCGCAAGAGACGAGCAGGGCGCAGACCCCGCCTGCACAGGTCCGTGAATGGCAAGAACAAGAAAGGGCGGAGGCG |
| comp104780_c0_seq5:1365-1366 | comp104780 | 1365 | C | G | 57 | 0.736842 | 0.263158 | 51 | 0.0392157 | 0.960784 | ACGCTTTCCTTCCCCTCGATCCGTGATTCACCCGCAAACGATTCTGGTACACTTAAATCTCCAGCATCTTCGTACCACTTGTCTTTACTACGAACTGGCTC[C/G]GGTTCATCTTCAAGTAATCCATTCTCACTCTGCGCTCGTGTTCCGGACAGCGGAGCAACGTCTTCATTCGTCTCAATGTTTCCCTCAGGGAGATTCTCAGG |
| comp97393_c0_seq1:734-735 | comp97393 | 734 | A | G | 62 | 0.822581 | 0.177419 | 8 | 0.125 | 0.875 | CAACGCCCCCGTGGCCGCTGTGGCTGGCTCCGAGGGCGGCGGCGCGGATAGCTACTCGGGTCGGGGCGGCGTCGAGATCACCAAGAAGATCGGCAGCAGCC[A/G]GAAGCCGGCCAAGGCCTCGCGCTTCGTGGCGGACCCCGACAAGCAGAAGCTGATCGTGGCCGTGGACGCGGAGGAGATCGAGCAGCTGAAGCAGGAGGTGA |
| comp99060_c0_seq2:903-904 | comp99060 | 903 | T | C | 133 | 0.857143 | 0.142857 | 119 | 0.159664 | 0.840336 | CTGCAGGTCCTCGACAAATACAGTTCCAGGCGCACACTGGAAGTGATACACTTGCCAATGGTCACCCACCTGATGACAGCTATAGAAGGCAGTGCAGTCTT[T/C]TGGGTCTGTATTGAAGCCTTCCCTGTGGCAGACGGTGCTTGGGGGCGGCGGAGAAGGCGTTGTGGTGGTGGTGGTATCCGTAGTGGTTTCTGGCTCTGGGG |
| comp105696_c0_seq14:1707-1708 | comp105696 | 1707 | T | A | 202 | 0.846535 | 0.153465 | 100 | 0.15 | 0.85 | GTGGAGAGGCCCACGACGACGTTCTGGGGTTGTCCGGGCACTCCGTCCTGAGTGGTGATGAAGGTGTCAAGAGATGCCCCTGCGGCGCCTGAGGGCGTCAC[T/A]CCCGTCACGGTGACCTGGTACCTCCCGCACGCCTCCAGTGCCGTCAGAGTGATCGAAGTGTCAGTAGTATTTTCGCATTTCGTTGCGGTGTCCGAATCCAG |
| comp91276_c0_seq1:165-166 | comp91276 | 165 | A | T | 8 | 0.125 | 0.875 | 56 | 0.821429 | 0.178571 | TATGACAATGACTCATATGGATAAATCGCATGCCTATTTCTCTATATTTACTACCTCTCATTATAGTCTGTACAGATCTCAACACAGATGGATATCAGTAT[A/T]AGTTGCAAAATTGCTACTAGTAGAAGCAGTAGTATTTATCTTTCAAATTACATGTAAAATTTCTAATGAAGAGAATTTTTCGTTTCTTGCAAATAACCTTT |
| comp96974_c3_seq3:315-316 | comp96974 | 315 | T | G | 77 | 0.844156 | 0.155844 | 54 | 0.148148 | 0.851852 | TTATGACTACCATTGACTCGGCGACCTCCACGAGTCAAAACCGATTCCTGAATGAACTGCTGAACACCTACGACATCAACGAATCACCTCACCTCAGCAGA[T/G]GCCGCCCCACTGCCGTCAGCGTGCAGATGTTCATCAATTCCTTTGGATCCCTCAACGCAGCCAACATGGACTTCAGCATCGACGTGTTCCTGAGGCAGTCC |
| comp95685_c0_seq1:841-842 | comp95685 | 841 | C | G | 105 | 0.857143 | 0.142857 | 31 | 0.16129 | 0.83871 | GCCACGGCGCTCTCTTGCCCGTAGTGCTTGGGCGCCGAGGGCTTCGCGTCGAAGGGATCCGGAGCCGTGATGGAGCCGTTGTAGATGTGCGAGAAGTCCTT[C/G]GTGCCCGGCGGAGCGGTCATGAGCTTCTTGTAGGTCGAGGCGGAGAAGAAGGGCTGGGGCTGCGAGTCGTCGTCGTCGGGCTGCGTGGCGTACGAGAGGTA |
| comp105751_c0_seq1:1892-1893 | comp105751 | 1892 | C | G | 71 | 0.788732 | 0.211268 | 43 | 0.0930233 | 0.906977 | GAATCGCCCAAGAGGAAGCGTGGCCCCCACAGCGGCGCCCCTCGCACAGCCGGACAGCGACCCGCACGACGATCTGCGAGCCACTCGAGCCAGCCGCGTCG[C/G]AAGAAGCCCTCTCGCGGAAGTGAGGAATCTCTCTGGAAAGCGTCCCTCGTGAACAGGACCGACCCCGCCCACGCTCTTGCAAATGACAACGAAGAAAAATA |
| comp107054_c0_seq22:235-236 | comp107054 | 235 | C | T | 62 | 0.870968 | 0.129032 | 34 | 0.176471 | 0.823529 | CGAGGGTCTCCAGTAGCGGCGCCACAGGTGATAGCGAGCGTATTCCGTGCGGGAGTTGGACACCCTGATAAGAAGTTCAGCAAGATCCTTTGGCGACATAG[C/T]CCTTGCATCAATGTAGGAGTGTGGCGGGAGGTAGTCCTCATATGTAGCCCCTCCGTAGACTACTGGCACCAGCCCGTGGAGCATCGGAAACCACACCTTCT |
| comp103913_c0_seq1:337-338 | comp103913 | 337 | G | A | 151 | 0.741722 | 0.258278 | 126 | 0.047619 | 0.952381 | AAGATTAAGTTCAGATGTCATGTATCTTCTTGGCATCAGCTGACTCCGGCCGACTTATTTTTCTCTCAAATAATGTGTATAGTTTCTTGAGGTTGGTGTTC[G/A]AAAGGAGGTTCCCGTGCCTTCTGCTCCTCCTTTGTACAGTGCTATAGTATTTATGTACATTTGTGTTGTGCTGTTCCTCGTTTTCCTGCGCTGTTGGTGAC |
| comp105801_c1_seq23:1703-1704 | comp105801 | 1703 | T | A | 289 | 0.709343 | 0.290657 | 130 | 0.0153846 | 0.984615 | ATGAAAAGCAAGCCGGTGCTGCATCTAGAAAAGTTGAATCCGCATCTCTTCAAATTTGTTGAAGAACTCAGTACAGTCAAGAAACTAAGAGAGGACTTGCT[T/A]ACCATGAAACAATACCTCAGCACTTGTCGCTCAGCTCAGGAATCTCGGATACTGCGACAACTTGAAGAGCGGCAACATTTTGTGGAGAATGCACATATGTA |
| comp93910_c2_seq2:655-656 | comp93910 | 655 | A | G | 250 | 0.856 | 0.144 | 86 | 0.162791 | 0.837209 | ACCGCGTGTTCCCAGGGGTACTTCCGGCCGTTGTCGTGGCGCCAGGAGAAGTGGTATTCGCTTCCGCCCTGCCTGTAGTCCACCACAGAATTGCTAATGAT[A/G]ACACTGGGGCGTTGTTGCACCACGAGTTGCACGGGTCTGGTCACAACGGGAGCGAAGGAGACACGTCCGACCCCCCTGCTCCCGTAGAACTGACTGGCAGC |
| comp99397_c0_seq1:909-910 | comp99397 | 909 | T | G | 123 | 0.764228 | 0.235772 | 112 | 0.0714286 | 0.928571 | AGACTTTTATTCTTTGTAAGGTGCATATCAGACTTGCATAAATTTGATGTTTGTTGTTATATTTATGTTGAAGGCAAAGCTTTATAACTAGAAACTGTTAG[T/G]AAACAAGGAAAGGTTTTAAGGATATGGAGTATGTTTTTCATACTAAATTAGAGAATAATGTTTAACATTATATCATCCATCAAAGAGCTTTTAAAATTATT |
| comp107686_c2_seq18:1340-1341 | comp107686 | 1340 | A | G | 392 | 0.716837 | 0.283163 | 277 | 0.0252708 | 0.974729 | ACATTTCCCCATCAGCTCTTTGTTTGTATTTTCATTTTGTTGTTCTTTGTCACCTTTGTTTTCTTTTTTCTTTCTGAATTCAAGTAATTTTATTTTTCTTT[A/G]TCTTTTGACCTCTTGATTTTTTCCTCTTGTTTTTCTTCTATATTTCTCAGATTCTCTTTTCATATTTAGTATAATTTGGATATCTTGTAAAGTTGAAGGCA |
| comp98453_c0_seq8:147-148 | comp98453 | 147 | G | A | 132 | 0.878788 | 0.121212 | 112 | 0.1875 | 0.8125 | ATAAACAGACTGCCATAATTCAAACCCTAAATAAGATTATTATTATCACTATATGTATGTATACAATGAAACAAATTACATCATGCTAACATTTCAATAAA[G/A]CTGTATTTCTTGTTCATCTTTTATTTCTTGTATTTTATTCAGAAGAATCCACTCTTTCCCCACGACGCAAAGTCTCCAGAATTTCCTTTACCCTTTTATCA |
| comp103564_c2_seq1:128-129 | comp103564 | 128 | A | C | 68 | 0.941176 | 0.0588235 | 8 | 0.25 | 0.75 | TGATTATAGAAAACGGGCATTAAGTAGGGTTAGCGATGACAGAAAACGTAAACTAAACAAAAATCATGAGAAATAAAAATGTTGTTTACGAGGATTATTTC[A/C]GCTCAAAATATACAAGCAACCTCTTAACGGTTGGTGTCGAGAGAAACCGAGAATTTAGAAGGATGATGACAGAATCGGGAATTACTGAAGATTAGTGATAA |
| comp107348_c0_seq1:192-193 | comp107348 | 192 | A | C | 256 | 0.835938 | 0.164062 | 172 | 0.145349 | 0.854651 | TATGATGGCCATCCGAGACAATGGCGAAACATGGCCACAGAAGAGGAGAAGACGAAGAAAAGAGGAAGAAGCAAGAACTAATTAACTCAACGGAATAAAAC[A/C]GTCAAGATAAGCAGATTAACAAGCATGAGTTTACGGATAACAAGAACAATCCGTCTCACAGGACCTGCACCACCTGAGAACGTCGAAATGCAGATAAGAAC |
| comp98638_c0_seq7:261-262 | comp98638 | 261 | A | G | 181 | 0.917127 | 0.0828729 | 251 | 0.227092 | 0.772908 | GGCATCGACATGAAGGACGTCGAAGCCGACATCTTCGCCCCAGCCACCGTCTTCTCGAACCAGGGCATCACCAGGTTCTCTGACTTCGCCATCTCCGGTGT[A/G]AACAACCAAGACGATGGACCCAACCTTAACATGGAGTTCGATGTGAACTCGGCCGCGCTTTTCGCCCAGTCCATGGCGGACGCGATGCAGAATCCCCGGTC |
| comp107600_c0_seq1:1550-1551 | comp107600 | 1550 | T | C | 50 | 0.84 | 0.16 | 60 | 0.15 | 0.85 | TTATTCACTAGAAAAACACTTCCTTAACCAAATATAACATGACATGAAACTAGAAAGTCAATGTCTACACATAGCACCTGTCTTCACAATGAATTTTCATA[T/C]ACAATGGGCATTCATCACCATCAAAACTTCCTTTGGTAACCTAGCTACGGACATTTCCTTTTCTCATGTATAAAACACAAAAATGAATACAAAAAAAAAAC |
| comp103005_c0_seq1:1683-1684 | comp103005 | 1683 | A | T | 107 | 0.850467 | 0.149533 | 112 | 0.160714 | 0.839286 | GAGTTTTTCTTTACAAATATCAGCAGGGAAAATGTGATGCGCCATGTTATGAAGCTTGTGTGGTACATTCATCCTAAACTACCCCCTGCTCGTCTCGACAC[A/T]CTGATGAAAGCACTGCAACCTGGACCTCATTCGAGCGAATCAAGCCAGAACCTGTACGACAAATTCCAAAACAGAGTACAGGAGTTCCAAGATGGACAGAA |
| comp107271_c0_seq2:2633-2634 | comp107271 | 2633 | C | T | 54 | 0.925926 | 0.0740741 | 38 | 0.236842 | 0.763158 | ACAGAGAAGTGGGAAATGCTGGTGCGGTGTAGGGGTCCGTTGGAAAAGCTCCTTAAAAGTGGGGGCTCGTCCAGATGCAATCGCATCAGCTCTAAAGAAGT[C/T]GCCACTATGGTTAATCTTATTAGTAGTTTGCCTCTTGCTCACCTCAGGGAAGGCTCACAGGCTCTGATATTGATGGCTCTCTTTGCCATCTTGGTGCAGGA |
| comp106320_c0_seq1:1143-1144 | comp106320 | 1143 | T | C | 21 | 0.0952381 | 0.904762 | 51 | 0.784314 | 0.215686 | ATGCATAAGGAATACTTGTTGCTTCTAATCATGCAGAAGATGTTTTGTGAGCTTTGTCTTTACTAAAGTTGCCATCCATAAAGCAGTTTGAAAGGCCGGAA[T/C]GGTATTGCATCTATTGTGCATTCAGTTACCATTCTAGTTTGTAGAAATTGTCCAGAACATGACTTTGTCTCCATTCAACAGTTAGTGTATACTCAACATAT |
| comp104622_c1_seq7:564-565 | comp104622 | 564 | T | C | 124 | 0.935484 | 0.0645161 | 357 | 0.246499 | 0.753501 | GGGAGCTCTAGGCCTACGCAACTCTCGAGCACTGGTCCGGCGTTGGTGTGGATGACGTCAACAAAGATGGCGTCGGAGGGGTCCAGGCGGTTCTCCTCCGG[T/C]TGGTTGTAGAATTCCGGGCCGGCGGGGTCTAGGCCCGTGATCCTCGGAAGCTTGGCAGACGCGAGGTGGCGCCCTATGCTCCCCGCGACGTGGGCGCCGAG |
| comp100187_c1_seq1:1004-1005 | comp100187 | 1004 | G | T | 398 | 0.796482 | 0.203518 | 251 | 0.10757 | 0.89243 | TTTTTTTTACCTTTGCTTATGTACAATTAGTTTTATAATGGGATGCTTAGTCATGTTATGCAGACTCAATATAGTGGCTTTGATATCCTTTAGAAATTCTT[G/T]TTTATATTGTTTTTGCTAAACTAGTAATATTGTTGTAAATTATGTATTTGATTTTAATGACTGAACTTGTTTGTATCATCTTAGTAAAATGCCAGGGGATT |
| comp103651_c1_seq2:103-104 | comp103651 | 103 | A | G | 55 | 0.8 | 0.2 | 54 | 0.111111 | 0.888889 | TCTCTTGTTTTGTTAGCATGCCTTCCCTGTATTCTTTATCTGGGTATATGAATTTCATATTTCACTGGATAACAATGAAAAGAGTTCAGGATAATTGAATT[A/G]TCTTGTTCTGTGGGTGCATGGATTTCAATTGCCAAAATGAGCTAAGACAAACTACATTGATAAAAAAAAAAGTTTTAATCAAGATTATCCTGTTTGTGCTC |
| comp106810_c1_seq4:1970-1971 | comp106810 | 1970 | G | A,T | 5 | 0.2 | 0.8 | 63 | 0.888889 | 0.111111 | ACCAGTTGCCATCACTACTTCCTCTGTATATTCTTCGATGGTACTGTTCCTCGCCCTTTGAGAATCAGATGCCCAAGAGAGAAGCCTGTGTTTGATACGGA[G/A,T]AGCAAGACCTGTCAAAGCCATGCTCCCTGTCGCACCTTGAATTGTCAGCAAACAAGCAGTGAATCAACAACAGTGGGATCCATCACTCCATCACCTGCAGG |
| comp104717_c1_seq13:1577-1578 | comp104717 | 1577 | G | C | 69 | 0.884058 | 0.115942 | 46 | 0.195652 | 0.804348 | AGCGTGCTCGTGGCGATCAGGCTGTGCCAGTACTTCCTCTCGCTCAGGTCCCGCTCGTACATGTAGGTGAGGAAGGTCTCGTAGGTGAGGTCCTCGTAGTA[G/C]GGCTCCTTCTCGTGCAGGGGGAAGGAGGCGGCGCGGAAGGACACCCTCGTCGTCAGGATGTTGGGCTCCACCACCAGGAAGGGCTGGGGGAGGTGGGGGGG |
| comp106642_c2_seq10:107-108 | comp106642 | 107 | G | A | 524 | 0.740458 | 0.259542 | 307 | 0.0521173 | 0.947883 | AGTGGACGCCAGGGGGAGAAGGCGGTCTGGAGTTGGTGGTCAGCTCCCGTTGTGAATGGTTGTGTTTCGCGTCGATGCGCCTGTCGCCCGAACACGTTGTT[G/A]AATTTACGGACAAGAGAAGAGGGAGATACGAAAAGATAAATCCCAGAGGCAGGAAATTATAATCGATTATCTCCGTGTACCCAAATGAAACCGTAAATCTC |
| comp107933_c1_seq1:2617-2618 | comp107933 | 2617 | G | T | 97 | 0.896907 | 0.103093 | 67 | 0.208955 | 0.791045 | CACATGAAGTCCAGGTTTTCGTACCCTTCTACGGGCGTGTCCATCGCCACCTGCAGCTCTCCGGCGTCGACGCGAGGGAAGCTCCCCATCACAGAGAGCTT[G/T]GTAGTGACATCAGTCTGGGAGGAGAAAGTGAGGTTGACCTCGGCCGCCGGTTCCCAGAAGATGGAGCCAACCGCCTCCAGGGTCGCGTCGCTTAGCCGTTC |
| comp105419_c2_seq1:1646-1647 | comp105419 | 1646 | A | G | 372 | 0.895161 | 0.104839 | 193 | 0.207254 | 0.792746 | TACTGAACCAGGTAAGCTCTCTGGCCTTCATAGGGAGTGATGACACCAATCTGTTCTGGCTTGACACCGGAATTGAGGAAGCGTGTGACAAGTTTTTCAAC[A/G]CTGGAGGCCTCGGTTCTGTTCAGGTAGGAAGTTCCTGACCCTGCAATTTCCTCCTGGCCCGAGGTCACATAGAAGAATTGGGGTTTGTCTTGTTGGGGCCA |
| comp107700_c0_seq2:656-657 | comp107700 | 656 | T | C | 331 | 0.782477 | 0.217523 | 243 | 0.0946502 | 0.90535 | GCCTCACCAGCTGCCTCTCGGAACACAGCGCGCAGGTGGTCCTCGATGCTGTGGTCTACCTCGTCCTCTGACTCATAAGCAATATCGAGAATCTGGGCCTC[T/C]TCGTCGTACTCCTCCATCCTGACGAGCTTCTCACAGAACACCCGCAGAAGGAACTCCGCCTCTTGGTCTGGCTCGGGTGTGCAGGGGATGACCAGGTACCT |
| comp104624_c0_seq1:1678-1679 | comp104624 | 1678 | A | G | 231 | 0.926407 | 0.0735931 | 67 | 0.238806 | 0.761194 | ATGGTACAGCTTGGCGGACACTTCATGAACATTATATCCTAATAAATAATGATGCCACATCCCTGCACCGCACTCTTTGGAATGAGGAAATAGGGGAAAGG[A/G]GGATATCCTGCCCCCTCTCCTTCCTCTCCCAAAAGAGAAGAGGAATGTGTCGAAAAAAAAATGTAGCGCGTCCCTCCCACTGGATTGCTTCGCTGCTCACA |
| comp100640_c0_seq1:899-900 | comp100640 | 899 | T | G | 43 | 0.744186 | 0.255814 | 88 | 0.0568182 | 0.943182 | TCTGTTCTTGCATATAGAAAAGCACAGAAAACTAAACATAATTTTTTTAGTGAATGTATTTGCATTTAATTACTGGGCAATTTTTTTTTTCCAATGAGAGA[T/G]AGATTATTTAGTAAGGTTTAGAAAACAAGATACAGAAAAGAAACAAACAATGGAATGGAAGTGCCTGTATATCTAGTGTTCATGTTTTCTTCAAAATTCCT |
| comp94248_c1_seq1:828-829 | comp94248 | 828 | C | G | 72 | 0.847222 | 0.152778 | 50 | 0.16 | 0.84 | ACGGTGGCCAAACACAGGAAGACGTGCCAGAGCTGCAAGTGTACCAGGGAGCTCCACGACGTCTACCACGAGGACTGGGTCAACGTGCGGGAGCGGCTGGG[C/G]CTCGGCGATTCCCTAGGACGCAGCAGCCGGGAGTTGTGCCTGCAGGAGGGCTACACGTGGGTGCCACCTGGCCTCACCAGCGAGCAGATCTATGACTACAT |
| comp103849_c2_seq2:438-439 | comp103849 | 438 | C | T | 87 | 0.758621 | 0.241379 | 14 | 0.0714286 | 0.928571 | TAATAGTAGTAAGCCTTAGCGTGGATATACTTAGTTGTTTGTTTTTAGAAATAGTTTTGTGTGATTTGAGCCATACGAGCACCATCCCTATCTTGTACTGT[C/T]CCGCTTGTCCGATTCCCTTTTGCTCGTTTTAATAACAATCCTCTTTTGTTTCGTTTCACTGCCGAAATCAGACACGATTTTCAGACGTAATAGTAATGGTT |
| comp105241_c2_seq1:288-289 | comp105241 | 288 | C | T | 53 | 0.943396 | 0.0566038 | 39 | 0.25641 | 0.74359 | TTCTTTGTCTCATCAGAAAATTCGCTAACTTTTTTAACTTCTACGTTTACACTGTCAGCTTCATATGTCACATGGTCATCAATGCCATCTTCCTCAAGTTC[C/T]TCTTCTGCTCCAGGAGCTATTTCTGCATCCATCACCTCCTCATTCTCTTTGTCAACCTCCACAAAATCAATGTCTTTGGTCAAATCCTCCAACGTTTCATT |
| comp103798_c0_seq1:2122-2123 | comp103798 | 2122 | C | G | 437 | 0.695652 | 0.304348 | 228 | 0.00877193 | 0.991228 | TATTACCATTTTAAGAGATTCATTGACACATCGTAACAGGGAAATCTTCAAACCTGTATCCAATACATTTTTGACATGAAATATCCAGGAACTAAATATAA[C/G]TTATAATATCACACTTAAAATCGGGTGAATGGTAAATTTAACTAAATCTGGAGCATGAACAATACACCAGTGCATTAAAAAGTGTTTCAAGTCATAATTTG |
| comp107099_c0_seq1:3203-3204 | comp107099 | 3203 | C | G | 93 | 0.892473 | 0.107527 | 63 | 0.206349 | 0.793651 | AAATGTAGAACTAAACCCATCATTCAGAAAAGGCTGATTAGGATTCAGTCTGCCTTCATGAATCTTCTTGGAGATAACAATGATTTGGTGCAAGATGCAGC[C/G]TCTAAAGGTTTAGGTGTTGTATATGAGTGTTGTTCTGAAGCCACAAGACAATCAATGGTGCAGGGTCTTGTACAGACTCTGACTGAAGGGAAACGCACCAT |
| comp100601_c0_seq2:1063-1064 | comp100601 | 1063 | A | C | 78 | 0.769231 | 0.230769 | 24 | 0.0833333 | 0.916667 | AATGTTTTGTATTTTCCTCATCTTTTTTTCTATTTTCTATTGGAACTTATCTCTTATCCTCCTTTTTTCTCTTTTCGATTTCCCTGTCCCCTTTCAAATGA[A/C]GTGACGCCTCATCATATATGTAATCTTTACGTTCTTCTTAAACTCCTGGAGACACATTTCACTCGAGATACGAACCACGTGATTGTTTCCTTCTTTCTTCT |
| comp106862_c0_seq1:640-641 | comp106862 | 640 | G | A | 150 | 0.873333 | 0.126667 | 176 | 0.1875 | 0.8125 | CTGCAGTCCCCTCATTTGTACCCTCCTCCTGCAGAAGTGGCTCCCCTACCTCGGCCCGCCGTGTCGCCCCAGAGGCCACAGCAGCCATTCGTCCCGGGGCA[G/A]ACAGAGCCAGAGTTCACCACGCCGGTGCCTCGTATAGTGGTTCACCAGGTGCAGATGTCTCTGCCACAGCCCGGCGACGACGACCTCTCTCACACTCCCAC |
| comp105484_c3_seq10:4945-4946 | comp105484 | 4945 | G | A | 253 | 0.818182 | 0.181818 | 166 | 0.13253 | 0.86747 | TGGCTTCCACGGCGCCCTAAAGCCACGTGCCAGGAATGAGCAGACGCGGTGACGGCAGTTCGTGTAGCGACGCAGCCAGAGTCCCGGCGGCGGAGGACACG[G/A]GAGGACGAAACAGACACGGGAAGCGCGTGGAAGATAAGGCGCGTCTCATTAGAAGATCGAGGGATAAGACAAAGGCGCGGCGACGACATGACATCCGCATT |
| comp106902_c0_seq1:1160-1161 | comp106902 | 1160 | T | C | 149 | 0.201342 | 0.798658 | 106 | 0.886792 | 0.113208 | ATGCTGCCGGCTGCACGCAACTTCGGGTATAAAGCAATAATACCACTGACCCATCAACCAAAGGAGCCACATGGTTTTTCTGAATATCACCACACAATGAA[T/C]TGGGGAGAAAATATATTCAAATATTCTTAATAGACTCCAAAAGCTGACTGTTAAGTACAAGTGAGTTTAATGGAAAACTTTAATGATCCCGACATAACAGC |
| comp96766_c0_seq3:2327-2328 | comp96766 | 2327 | T | A | 96 | 0.708333 | 0.291667 | 43 | 0.0232558 | 0.976744 | AAAGGTTGAATCTGCAAAGAATTAATATAATGTGCCATGTGTATTGAGTTGGTTTTCCAGAGCTTAAAGAGAGGATTATCTAAATTTCCCTAATTTTTCAG[T/A]AGACTTTAAAAAGAGAAATGCATTAGTTTATATTGAGATATTAGAAACCAGTGAACTTAGGTTTTAGACTTTCATAATATTTTTGTCCATGGCATAAAGAA |
| comp104977_c0_seq5:227-228 | comp104977 | 227 | C | T | 47 | 0.829787 | 0.170213 | 69 | 0.144928 | 0.855072 | GGAGGGAACAGTGAGGAAATATGGTGGAGGGAACAGTGAGGAAATATGGTGGGGTTACATGCAAGGAAACACAGAGGGGTGTGTGTGAGGTTCTGTGGAGA[C/T]GTATGCGAAAGGGTTAATGTTTGATGCACTATGAAAAAAGTTACGGTGGTATAGTCTCTCTCATTCCTTCTCCCACTTACCCTCTCCCCATCTGTCCGTCT |
| comp107560_c0_seq2:1676-1677 | comp107560 | 1676 | G | T | 56 | 0.910714 | 0.0892857 | 146 | 0.226027 | 0.773973 | AGGCCTAGCTTCCAGGCACTGGTGGTTGCTGCTCACGTCCACTGTATAAGCAAGCATGTTATGTAAAAGGATGCATAAACACTGAACACCATGTCCTGGAT[G/T]TTTTAGGTAGACTGAGGTGGTCCAGTTCATCGTGTTCTGCAAGTGTGTAGATGAGTACTTTTGAAGAATGACAGATCCATAAATGCATTTTGTATAGAATA |
| comp107778_c1_seq18:3271-3272 | comp107778 | 3271 | C | A | 64 | 0.015625 | 0.984375 | 60 | 0.7 | 0.3 | AACTTGATTCCTTCGAAGTCCTCCCAAGAAGTTATCCGACGACACGCTTCTGCCGATGACGAATTTTCGGCGGAGCGGGTCTGGCGCCGCTTGTCTCTCCG[C/A]CCTCCTCCTCCACCTTTCGATTTATTCTTTCTCCCTTTTTTACGCCCTTGCGCCGTGACATCGGTGATTGAGCGTACCGTCTGCGTTCCGTCTGCGTTCAA |
| comp101758_c2_seq2:888-889 | comp101758 | 888 | A | G | 51 | 0.784314 | 0.215686 | 20 | 0.1 | 0.9 | CAAATTTTTCACATAATCAAATTAATTAACTTATCGTATTTAAAAGCACAAACAACTTACGTAATCACAAAAGAAGGAACACGCTGATTATGTATTTGAAC[A/G]CTGGTAAATTACATTTCGCTCAAACTTCCTTACACATCCGCTACGTTTTAAACTAACCTCTTTACGCTGATCACCCATCATGACCGGGTTCGGTGTAAGGT |
| comp101111_c0_seq1:1035-1036 | comp101111 | 1035 | G | T | 58 | 0.724138 | 0.275862 | 25 | 0.04 | 0.96 | GGGAAAATGCAGACCTACGACAGATTGGTTCGCTAGAATTGCTACTCGATACATCTACGAACTAATATTTTCGTAATCAAAACTTGTTTCTCTGTTGGCTA[G/T]AGATCCCACCCTTTCCAAGCTTGCACAGTCAAGCTGCTGTTAAAAGTCGCGAAATACAAGTCCTGATAATCCCACTTCACTCCAGCTGATAAAGGACTGAT |
| comp102819_c0_seq1:1005-1006 | comp102819 | 1005 | A | G | 65 | 0.753846 | 0.246154 | 43 | 0.0697674 | 0.930233 | AAAATGCCTTCTGAAGCTGAGCTTCGGGCTGGCATCCGACGGGCTTGTTTGCAGCGTACCTTTACCCCAGTGTTTGTGGGCACTGCACTAAAGAACAAAGG[A/G]GTGCAGCCACTGTTGGATGCAGTTTTGACCTACTTACCACATCCAGGAGAAGTCAAGAATTTTGCTCTTCAGGAAAACAACAAAGGGGAGGAAGACCGCAT |
| comp97192_c0_seq1:999-1000 | comp97192 | 999 | G | A | 25 | 0.96 | 0.04 | 76 | 0.276316 | 0.723684 | ACAATAAGTTTTAATTTCTCTACATTGTATTGTAACTTTTAATTTCTTTCAAAATATTTTGAATCTGGTTTCTTCCTATAGTATATCTATGCATGTTTGGC[G/A]TTATCACGTTTACTCTGTTAACGCAATATTTTGGCAATTATGATTTCTAATTGTTCACAATTGTATTAGATTTATTCCAAATGTATCTTGATTCTGATTTC |
| comp107420_c0_seq6:659-660 | comp107420 | 659 | A | T | 33 | 0.242424 | 0.757576 | 108 | 0.925926 | 0.0740741 | GGACCGGACCGAACTAACTAGAAGCAGTGGGAAATTATTTTCTTTCATAATCAGATTCTAATGTCTACAATTTCCGAGTTTTACTACAATTCTATTCGTTG[A/T]ATAGCATCCCTGTTTACATTACAATATTTCTGAATATTCAGTTTTTCTGTTCCCAAAAAGACTAATTATCTAGCCAAATTTTCACTAACTCTTCATCCAAA |
| comp99224_c1_seq1:928-929 | comp99224 | 928 | G | C | 19 | 0.894737 | 0.105263 | 52 | 0.211538 | 0.788462 | CTCAAGAAGCTAATGAGACAAGGGAACAAGCTGGCCAGGAACCTCTTCACGCTGCTCGGGGGGAAGGTGAACGAGTGGAAGCAGGACCATCCCGAGGTGGT[G/C]ATCGTCGACATCGGGCCGGGGGCGGGGACCAAGGCGGCGCCCGACTTCGATAGCGAGACCATGCCCAACTTCTTCGAGGATCCGGAGGTCCAGGCGGTGCT |
| comp102100_c0_seq2:106-107 | comp102100 | 106 | T | C | 97 | 0.0618557 | 0.938144 | 302 | 0.745033 | 0.254967 | GGAATATTAAAAGAAAATATAGTAAAAAAAAATAAATAAATAACAGCTAAGTTTTCTGCCATTAATTAATCTGCTGTAAAAAAAAAATCATATCCAAATAT[T/C]AAAATGTTTAAACTCTTGCAATAGTCCTGATATCTGAAACCGAAATTTGCCTAAATTATCAAAATTTTAATATATACCTCATCCAAATGCGTCTCATTAGA |
| comp106768_c1_seq4:721-722 | comp106768 | 721 | C | T | 10 | 0.7 | 0.3 | 59 | 0.0169492 | 0.983051 | ATAAATTCAGATATAACAAATCTGTAAGTCAATGAAAACACTGGTCTTTCAGTGTCACATAAGGATATAAGCTGTACTGTACAGACGTAGGTAAGAGTATG[C/T]ACAGCTTCATACAGAGTCACTGGCCCACACAGCCCCTCATAAATCCACATTATACCAAGGCTGCGCACAAACTCAACAGAAAAACGACGAAGGCCATGCTG |
| comp107285_c0_seq11:1155-1156 | comp107285 | 1155 | G | A | 111 | 0.864865 | 0.135135 | 22 | 0.181818 | 0.818182 | TACTGAGAGATTCGAGTCGCTCTTTTACTTGGCATTAGCTAGAATTTTGTTGGTGGTAGTCAGTGGCTATAGAAATGTCATATGATTACAATTGGATTTCC[G/A]TATAAATACATACAATGTATTTAGAAAAGGATTTTATCTTTTGGTATTCATCCTTTGTTCATGCAATAATTCATTATGAAGGTTCAGCCTAAAAGATTAGC |
| comp96328_c0_seq1:215-216 | comp96328 | 215 | G | A | 84 | 0.77381 | 0.22619 | 11 | 0.0909091 | 0.909091 | CTCCCCCTACGTCCACAGCCACCACAAGAGGTCCGCTGAGGCTGAGCCTGGCTATGGCTACGGCTACAAGACCTACCACCCACGTCACTACCACTCCTACC[G/A]CCCCCACTCCTACCACGCCTATCCCTCTTACAGCCACGCCCACTACGGCTACCACTGAGGCACCAGGAGAATGGTAATTTAATCCCATTTTCAATTGATCA |
| comp101205_c1_seq1:1252-1253 | comp101205 | 1252 | T | C | 98 | 0.836735 | 0.163265 | 78 | 0.153846 | 0.846154 | TATACACATATGTATGTCTATAAGTGTGCAGCCCATATCAAAGCAGGATGGCAAACATGTAAATGTAGGAAGTAATGATAACTTTAAAAAAAGGAAAAAGC[T/C]AATTAAAAAAAGTCTTGGTGTTTCGGCAAGGGACATGTACTTAGACGCCTTCACTCGGGCCATTTCCATGAGGTATGCTGAGGTATGCTGCGTCTTGGCGT |
| comp103715_c0_seq1:1173-1174 | comp103715 | 1173 | T | C | 114 | 0.798246 | 0.201754 | 78 | 0.115385 | 0.884615 | ATGATAGTCGGTGACTTTGATGATATGGGTGTAGCAGAGGGGTTAATAAATGGAGAATTAGGAGAAATTAAAACATTACGTCTACATGTCCATATAGACAG[T/C]GACAATTGGGCTATACTTAGTGAGATTCACATAGAAACTCCGGAAAAGAGGTGACCAGACGCTCCAATGGCCTTCACCTTCTGCGAGTGTCCAAGCATCTC |
| comp103879_c2_seq15:299-300 | comp103879 | 299 | A | T | 146 | 0.924658 | 0.0753425 | 62 | 0.241935 | 0.758065 | TGAGACAGCAAGTCGCAGCCTTCACGTTCCTCATCATAAGATGGTAGTCCAGTGAGGAGTTCTAATATAACAATTCCAAAGCTGAAGGTGTCCATTTTCAC[A/T]GAGATGTCTCCCCTAAAAGCTTCTGGTGCCATGTAAGCAGAGGTGCCAAAGACTGTAGTGGTTTTGATGAGTGTGCGAGTGTGAGTGCCGCTGCCTCCTAG |
| comp101432_c0_seq1:384-385 | comp101432 | 384 | T | C | 118 | 0.79661 | 0.20339 | 79 | 0.113924 | 0.886076 | AGGAAGCGCTGAACGCATTATCAAGAAAATCCTTCCCGCTGTGAAAGGAGAGGGAATCCCCGGCATCTCCCCTGTAATATTTGGCTTCAAGAGCGTACTGC[T/C]TCTCCCTGTCGCCCACGTAGAAAAAATCGTACTCAGCCCAGGTCTCGTTGCCGTAGAAATCGGTCATGTCGATTCGTATCTGGCTGTGTCCTTGGTTCGTG |
| comp101472_c1_seq2:406-407 | comp101472 | 406 | T | C | 85 | 0.847059 | 0.152941 | 79 | 0.164557 | 0.835443 | ATAAATACTGTCTATTTACGACACTTTTTCAGTCTACTGCAAAATAAACTTTCCAGTTTATATTACAAAAATAATGCATTAAAACAGTGACTAAAATAAAA[T/C]AAAACAAAATTAAATGTTTCCCCTTTTCCTTCACTCCTCCTCTTTATCAACCTTTCCCTTTCTCCCAAATGCCTCGTTGTCATTTTCTTTATTTTCTTTCT |
| comp99460_c2_seq1:211-212 | comp99460 | 211 | G | A | 58 | 0.724138 | 0.275862 | 24 | 0.0416667 | 0.958333 | TTACCAATTATGTCAGCAACTTTAAGTCCCAACTCCCTTGGGTAAAGCTCTGGATTTATGATTTTATCATTGTGACTCAGAACTATCTGACTAACACCAAC[G/A]CTAAACCTTTCTGCGAGCATCTGGTATACGTGGGAAAACTTCTGCATCTTTTTAATGGGTATGCGGAGATATTCAGTCCCCCATCGTACACGCAGATTTAT |
| comp103093_c0_seq1:1503-1504 | comp103093 | 1503 | A | T | 515 | 0.768932 | 0.231068 | 299 | 0.0869565 | 0.913043 | AAACAATAACAACTCTTAAGTGAGAGGCAATGCAGAGATGTTACTTCTATATGGTCTATGATTTGTATCGCATAGACATAACTAAGATGAAAGAAAAAAAA[A/T]TCTATGGCAGCTACTGGTGAAAATATTCTGAATAGAAATATATACACAATATAAGACACTTTCCAATGAGAACAGTGAGAACCATGTATTATGATGACCAC |
| comp101077_c0_seq2:690-691 | comp101077 | 690 | G | A | 291 | 0.920962 | 0.0790378 | 205 | 0.239024 | 0.760976 | TATCCTTTTCTTATGCTCTCTATGTAGACATCTTTAATCCAAGCCATGTCCTGTACAAATTAGGAGACGGCTTAGTTTATGAATGTCATCAACCTTACTTT[G/A]CTATTTCTACCCAGTGAAGAAGTCTGTATTTATGGAAAAGTAAAAACAAACAAAGACAATCAGCTGTGTATATATGAATATATGTATGTGTATATATATAT |
| comp101780_c0_seq3:185-186 | comp101780 | 185 | T | C | 402 | 0.793532 | 0.206468 | 412 | 0.11165 | 0.88835 | AGTATGAAATTCTTGGTGCTGTTGGCGCTGGTGGGCGTGGCGTCTTGCCGCACCTCCCTCATCGGGGCGGATCGGGCTAGGGACGTCGACCTCAAATACAC[T/C]TACGTTAACGACGAAGGAAAGGAAGTGCGAGTCAAAGTCGAGGCCGGACGTCTAGGCCTGTCCCTCCGGAGGCAGCCCGACTCGGAGCTGGACGTCGAGAA |
| comp107468_c0_seq2:1760-1761 | comp107468 | 1760 | C | A | 250 | 0.8 | 0.2 | 203 | 0.118227 | 0.881773 | TAATTCACACGGAAGGAAACTGAGCAAATGTCTAGGAATCTTGATACACGACTACCAGTTTATAAGCTTCCCTGCTTCCAAGTTTACAACTCAGAACTGAT[C/A]ATATATGAACTGTCAGTTTGGAAACACATTCCCCCTTTTGATATCTGCGTATCTGTGTTTTGGATACTTGAGCTGAAAATATTCACACACTGACAACGCTG |
| comp96786_c0_seq1:195-196 | comp96786 | 195 | G | A | 126 | 0.769841 | 0.230159 | 68 | 0.0882353 | 0.911765 | TGATGTGCATCTGAATGTGTGTGACACCAAGTACTTAAGTGCAATGTCAGTTCTTTTCACTTATTTTTACACAATTACAGTTTCTTCAAATGGTCTGTACA[G/A]TTAAGTCATATTTATTTTTATGTCCATATCATTTTTCATTTCAGTTTCAACCCATAGACGCATAGGAACCATTTGCATCAAGTCCTAAAGAGAACATTTTT |
| comp103138_c0_seq2:408-409 | comp103138 | 408 | G | C | 54 | 0.814815 | 0.185185 | 15 | 0.133333 | 0.866667 | AAGAAGTGCAGAAAACCAATTTCTAGGAACAAGAAAACATCAGAACAGTTCTATTAACAGGATAATTAAATACTACAGTCAACCCAAACATTAAATGAAGG[G/C]AGATGTGACAGAATAACACCTATACACAATATATATTCAATGACTTTTGTCCATACTGCAGTTAATAAAGAATTTCTCAGTCATTTCCACATACTTTTCCC |
| comp107705_c1_seq1:228-229 | comp107705 | 228 | C | T | 343 | 0.825073 | 0.174927 | 306 | 0.143791 | 0.856209 | TTCTGTACTGGGTATGTACGTCGCGTTGCTGGATTGGCAACACTGCGATACTTCCCAAGGATATAAATCTGGCAGATTTTGAAGAAGCACTTGATGCCTAT[C/T]AGCTGTTTCCTGCCTTATTAGGGCAAGGTAATTTGATCAAGTAACGTGGGATTAAAGGTAGACGTGTTAACGTAGTATAACCTCCCGTTAATATAGACATG |
| comp106176_c1_seq2:1463-1464 | comp106176 | 1463 | T | G | 220 | 0.936364 | 0.0636364 | 235 | 0.255319 | 0.744681 | AAAATAAGCTAAATGGTGATTTCTCACTCTTAAACGGCAATACATAATCTGAAAAATATATATTCATATGAAATATTAACTTTCATATATACAAGATAAAT[T/G]AACATGAAATGAGTAGTATTCATTGCTGATATTGAAATTTCATACTTGTGTATGTTACTGTTATTACTTTCATTAGAACTGGAGAGTAATAGATCTTTTTT |
| comp105451_c2_seq18:323-324 | comp105451 | 323 | A | C | 52 | 0.730769 | 0.269231 | 20 | 0.05 | 0.95 | GCCGACTTCCCCGACGGCCCTCCTTGACATGCCGACCAGCAGATGACTGAAACTGGGGTGGCCGCGGAGGAGGCTGAGGCACGCAACTCGCCGCCGACCTG[A/C]GAGGTTCTCACGTGATTTGCGCTACAGCACTGGAAAGTCAACAGCCATCAACATCGCCACCACGCCGCTCGCCAACGCTGCCTCCTGAAGGGCGCCTCGTT |
| comp106171_c1_seq10:2082-2083 | comp106171 | 2082 | C | T | 97 | 0.824742 | 0.175258 | 111 | 0.144144 | 0.855856 | CTGGTCCTTCGGCTGCTGCGGGGGTCGCCCTTGACGCTGGCATGTGCTGCTTCTTTTCGGAGGCTGGATGCTGATTTCAACGTTGAAATTCTCTCCTCCAA[C/T]CACTTTGTACTGGCGTATTAATTCCTCTTTTGAAACCTCGACATTGAAATTCACAGGTTGAAGAGTACTATCCGTCGGCTGTCCTTCCCCAAAGTAAATTT |
| comp101707_c0_seq7:735-736 | comp101707 | 735 | A | G | 81 | 0.839506 | 0.160494 | 44 | 0.159091 | 0.840909 | AGAAAAGTTAGTGCCTGGTTTGGTGCAGACTTCCGGCTCTTCCTCCATAGAAGGGGATTCTATCCTCAAGGAAGAGGAGAGGTATCCTTAGAAGTGAAACC[A/G]GCCCGACAAGGCCTGATTGCCGCAGATTTTACTGACTTTGGTGAAGTCATATCAGTTTATGGCAAAGCTTATGTGGCTGGAGTTCTCCCTATACGAGTTGC |
| comp94290_c0_seq1:105-106 | comp94290 | 105 | A | T | 64 | 0.78125 | 0.21875 | 59 | 0.101695 | 0.898305 | TATAGAAATAACATGGTTTATTCCGTCCAGATATTAGTAATCCACACACAGGATCAATCACCACTGTGGACATTTCTAAAAGTTTAAGGCACAAGATTTGA[A/T]TGAGATCAGTTCAGGAAGAGGGCAAAGCTTATCTTATCTCTGAGAATGCTTGCAGATATGGATACAAATAACGAAATGTGACAACGTTATAACGTACGAGA |
| comp106499_c0_seq3:851-852 | comp106499 | 851 | T | C | 386 | 0.746114 | 0.253886 | 224 | 0.0669643 | 0.933036 | CCGGCCCCCACATGCATCAAGATCCGCGACGGGAAGGAGCACAGAGGGAAACGTGTCAAAATCAGGGGATGGGTGCACAGGCTAAGGAGGCAAGGCAAAAA[T/C]ATGATGTTCATCGTTCTGCGAGATGGCTCAGGATACCTACAGTCGGTGCTGACAGACCAGCTCTGCCAAACGTACGAAGCCATCATCCTCAACACAGAAAG |
| comp94538_c1_seq3:142-143 | comp94538 | 142 | A | G | 190 | 0.678947 | 0.321053 | 2 | 0 | 0 | TAGCCGTTCTCGTTGGCCACGAACTTGAAAGAGGCTACGGTGCCATCTTCCTGAGGATAACTCCAAGAGCCAACCATGTTAGAACCGCCAGCGACGCCCTC[A/G]GAACCCGAAAACTGGAACACGATGCCATTGGCAGCCTCGAAGTCAGAGCTGTGGGCGCCGAACTGGTCGGGGTTCACCTGGCTGCTTCGCACGATGGCGAT |
| comp106790_c0_seq3:1803-1804 | comp106790 | 1803 | T | G | 72 | 0.694444 | 0.305556 | 63 | 0.015873 | 0.984127 | ATGGTTTTCAAAGCATGGTATCAGCTGACCGAGACTAACCCCGGTCCAGAAAAGTCGAAAACACTTGGTGATGTGGAGGCGTCCTAAACTTTGTAAACGTC[T/G]GATGCCATTATGTCTCGGTATCAAACACCCAATCAGTGTGGTATATGTTTACCAGCAGTACGCAAGGCTCATAGTTACTGATCTTCTCCTAGTTATTTGTC |
| comp106527_c0_seq1:809-810 | comp106527 | 809 | T | C | 29 | 0.103448 | 0.896552 | 55 | 0.781818 | 0.218182 | TGCATATGCATCTATTTGTGCTTGTGCATCTATTTGAGCTTGTGTGTCTATCTGTGCTTGTGCATCTATTTGTTCTTGTGTGTCTATCTACACTTGTGCAT[T/C]TATTTGTGCTTGTGTGTGTATTTCTTCTTGTGCATATATCTGTGCTTGTGTGTCTATTTGCACTTATGTGTGCATTTGTACTTGTGTTTATCTATGCTTGT |
| comp101418_c0_seq1:2630-2631 | comp101418 | 2630 | A | G | 75 | 0.893333 | 0.106667 | 93 | 0.215054 | 0.784946 | ATGACATCAAACAAAGGGAGAAAGATAAGGATACCTGCTTGTAACTTATAGAAAAAAAATAGTCAAAAAGAGACAATTCCATATAATACTCATAACATAAA[A/G]AAGGTCAAGGCAGAGACATAAAATGAGAGAATGATTGAGAGGAGAAGAGTGGGGAGAAATATACAATAACAAAGGACTCTTGAAATATTCTTGCTTTATTT |
| comp107918_c0_seq2:5966-5967 | comp107918 | 5966 | G | A | 153 | 0.882353 | 0.117647 | 137 | 0.20438 | 0.79562 | AAATTTAGATTTCCTCTTTCTGCACTGAATGAATACATACAATCAGCAACCCAATTAATGGTTCCTAAACTGAATTTATGCAATTTTACTGTTAATATGAA[G/A]TGTTTTGCAATCTATTTCTAAACATTCTAGTAAAAGTATTCAGTCAGGTTTCACGTGGGTAATAAAATGACACCAGCACTTCTCTGTATTATTACACAGAG |
| comp99517_c0_seq5:888-889 | comp99517 | 888 | G | A | 1444 | 0.696676 | 0.303324 | 957 | 0.0188088 | 0.981191 | GGGTCGGGAACCATTCCGGTCGCGGGCGCCGGGTCGGGGCAGACCGGGGCGCAGTCGTCCAGGAGCACCGAGCCATTCCTGACGGCGGCCGCGATGAAGGC[G/A]AGACTAAGGTATGAAGGTAACATCTTTAAAAAATGACTTGCGTGGCTTGACTGACGACTTAAATAACGACTTGGGCGACTGACAGCTTCAGTGACTGACAG |
| comp106165_c0_seq2:192-193 | comp106165 | 192 | A | G | 33 | 0.818182 | 0.181818 | 128 | 0.140625 | 0.859375 | GAAAGGGGAAAAGACGAACACCAAACGCCGATAGATAGCGTAGAAAGAACACTTAAGTCACCCCTTTGACGCCTCTGACCCAGGGAACGTCAGGCCAGTTT[A/G]CTTAGAGGAAAATAATATCTCTAGTTTTTTTTCTATGTCAGATTATTTCTCGAAGAATGTCCAGAAGCTGTTCATGGAGTTATTACGCCTTATAGTTATCA |
| comp105406_c0_seq1:1364-1365 | comp105406 | 1364 | C | T | 58 | 0.758621 | 0.241379 | 37 | 0.0810811 | 0.918919 | CACATTGAGGCATAGGGGGCCAGTACTGACACAAGGGCAGCGCTAGCGAGGGCAAGGGCAGGCTGTCGGTGGGGTGCACCAAGGGCCCCACACAGAGCCCC[C/T]AGGTGGACTGCTGCTACATTCACGCCACCCTCCACACCACCAACGCCCTCACGACGCACGCCCCACACCACACCCCACACCACCTCGCTCAATAGGCCGGT |
| comp103287_c0_seq17:2232-2233 | comp103287 | 2232 | G | A | 62 | 0.822581 | 0.177419 | 62 | 0.145161 | 0.854839 | AATCCATTACTCACGGACGAATATCCGTTTCCTACACTTGAACTCAACAACCCATGTTCCGGAGATTTCTCCCTAGAAAGGCGACCCTCCTCGTCCTCGAA[G/A]AGACCAAGGCTAAACTCAAAGTCCGGCGACTCCCTCCCAGTGCCCTCCGTCCTGATCGAGTCCGCCATGGACACCCCCATCCCGAGCCCCATCGCCCCGAG |
| comp104937_c0_seq2:899-900 | comp104937 | 899 | T | C | 94 | 0.851064 | 0.148936 | 69 | 0.173913 | 0.826087 | GGTTTTGGATATTTGGCTAGATCAACTCTTTTCTTCTCAGCTTTGTTCATAAACGCCTTGGCATGATCTACAGGGATGGCAAATGAGATTCCTGAAGTTAC[T/C]GTCATTGAATTTATTCCAATGACTTCTCCGTCCAAGTTGATTAGAGGGCCTCCTGAGTTGCCAAAAGTAATGGCTGCATCGGTTTGGATGTACTGCATGTC |
| comp102765_c0_seq1:1488-1489 | comp102765 | 1488 | T | C | 112 | 0.767857 | 0.232143 | 88 | 0.0909091 | 0.909091 | GAAACAATAGAGATCTTGTTTGGAGAAGGTTTGATCAAGTGTTTATTTGCAACTGAGACCTTTGCCATGGGACTTAACATGCCTGCCAGGACTGTCCTGTT[T/C]ACCTCCATTCGCAAGTTTGATGGAAGAGATTTCAGATGGCTAACTTCGGGAGAATACATCCAGATGTCAGGTCGAGCTGGTAGAAGAGGAAAGGATGACAA |
| comp100260_c0_seq3:712-713 | comp100260 | 712 | T | G | 1629 | 0.203806 | 0.796194 | 1500 | 0.880667 | 0.119333 | CCCTTTCTTCTTACCTTGCTTCCTCGAAATAAAGTTCTCCGTCTTCTTCATAACAGGTCCTCTTCGCGCACTTCTCACTCATGGGCCACGAGGAACCCGGG[T/G]TAAAGCCGGTTCCTAGGCTTGCGACATAGCACTTACCAGGGAAGTCTTTGTGAACCTCTGCTTTCCCTCTGAAAAGCGCAGCGAAAGACACTCCGACAACA |
| comp104493_c0_seq1:821-822 | comp104493 | 821 | C | G | 87 | 0.735632 | 0.264368 | 17 | 0.0588235 | 0.941176 | ATTTCCACGTCCTGTCGCCCATGTCTTCGGGACTGTTCTGCCGAGCTATCCTGCAGTCGGGAACGTCGTTGTGTCCGTGGGCTATGGCGGAAAACCACAGA[C/G]AGGTCGCCGATAAGATTAGTCAGATGATGAACTGTTCACGTCCGTATGATCAGCAGACGATCAGCAGCTCAGGCCTTGTCGCTTGTTTGAGGAATGCCCCT |
| comp106595_c0_seq10:284-285 | comp106595 | 284 | A | G | 175 | 0.708571 | 0.291429 | 218 | 0.0321101 | 0.96789 | TTCCAATACTAACCTTCCGTCCTCTTCGGCCAGGCTTCGGAGGGGAAAAAGGTTCAGATCGACGTGACGCATCTTAGGATCCGATACTCCAACTTCCTGGC[A/G]GTGAATCCCACGACAGAGCTCTACTACCGCCGTGGCAGCGTGATCTACCTGCCGCACCGTTGGGCTCCGAGGCAGATCGTATCGGATTCTAACCTCATCAG |
| comp103220_c2_seq1:513-514 | comp103220 | 513 | T | A | 82 | 0.865854 | 0.134146 | 58 | 0.189655 | 0.810345 | TGGTCACGCTCTTGTGCAGCAGAGATCATAGGGTAGGTCACGCTGGCTCTTGACCTTTGGCTTGACGCTCCCTCTCAAAGGCCATTGAGGCAGTGCTGTAT[T/A]TTTTTTTTCGCTTTGTCTCTGTTCCTGTTATTTACTGATACATGGACTCTTGGCTCAGGATATTTATTTATGTATGATGATCACAGATTTTTATCTATGTT |
| comp107311_c1_seq4:508-509 | comp107311 | 508 | C | T | 81 | 0.925926 | 0.0740741 | 16 | 0.25 | 0.75 | AGCGACGGGGACGAGCGCTCGGCCTCCTTCGTCCCCGCAGGCGACGACCGGGGCGCCAGGAGGGTCTCGGAGCTGCCGCCGACGGCCATGTTCCCTCTCAG[C/T]GTGTCCAGGACGCGGCTCGACAAGGAGGATTCCACGGAGAGCGACGAGATGGCGGGGGAGCCGTCGCTGGCGTGTCCCCTGGAGCCGCAGCATCAGGGGAA |
| comp106486_c0_seq1:548-549 | comp106486 | 548 | C | T | 551 | 0.798548 | 0.201452 | 374 | 0.122995 | 0.877005 | CGTGCTCTGCATCACCTCATCAAAGAATTCCAGGATGCATTTTCTTCACTGGAAACGTGTCCAAAACCAGTGATTGCTGCTATACATAATGCCTGTGTTGG[C/T]GGTGGGGTGGACCTTATCTGCTCAGCGGATATTCGATACTGTACATCTGACTCATGGTTCCAAGTTAAGGAGGTAGAACTCGGCCTGGCAGCAGATGTTGG |
| comp105097_c0_seq1:104-105 | comp105097 | 104 | G | A | 589 | 0.925297 | 0.0747029 | 20 | 0.25 | 0.75 | TAATAGTAATAATAATAATAAAAAAAAAAATAATAATAATAATTTGCCTTAAAATGAATCAAATACTTTGACTAACAATTCATATTTCAAATACGATTCTA[G/A]TGGAATTGATGAAGAAAGAGCAGATCCTAAGTAGAATTGTAGCCGAGGTGAAATCATGATGAAATGGCAAACAATATGATAGAAGATGAAAAGGTATTGCA |
| comp99401_c0_seq2:421-422 | comp99401 | 421 | T | A | 107 | 0.925234 | 0.0747664 | 20 | 0.25 | 0.75 | GCCGCTCCTTTGAATCTTTCACCTTCCTCAGCTCCAGATTTCTTGCCGATTTTCTTCCCGACGCTGAGGATCGTCTCGGCGACCATCTTTCTTCCCGATTT[T/A]TTTTTCTGTCAGATTTCTTTCCCTGGTTTCGATCGTCGATCCTTTTCTTCGCCATTCTAGTGGCTTAATCGGTATTATTTGTGGTGTGTTCGCGTATATAT |
| comp102230_c1_seq1:601-602 | comp102230 | 601 | A | T | 49 | 0.795918 | 0.204082 | 58 | 0.12069 | 0.87931 | GTATCTATCCTTTACTTTGGTCTGTTGACAATTCATCCCCAATTAAAATAAACATAAAAGTTCTTTGAGCTTTTACATATCCTAGAATACTGGAATTTATT[A/T]AAAAAGTTACCATTTTCCTGATCTTATATAAGAAAATGTCATAGCAACAGAACAAAACAAAAATTCATCTTTCAGTTCTAATTTCTGATTACAATTTACAA |
| comp99009_c0_seq1:1325-1326 | comp99009 | 1325 | A | T | 125 | 0.84 | 0.16 | 103 | 0.165049 | 0.834951 | TCTTTGTGTAAGAATTGATTATAGACAGGAATTAAAGTCCTCATGAAGTGTATGGAGTTGGACCATAGTGATATCAGCTCAACCGAGAGGGATTTGCAGAA[A/T]GGGGTCACGCTGGAGATGTCCGCTTGCATGCTAGCGTGCACAGAAACATAAGCTGGAGATGTGATGAAATTCTGATTTTTTTCCAACTATGTATTTTATGA |
| comp107592_c0_seq5:814-815 | comp107592 | 814 | T | A | 89 | 0.202247 | 0.797753 | 114 | 0.877193 | 0.122807 | CAAACCACATAAAAGAGCAAGATATTGATAGTGAAAAAAAACATGCAACATACTTTATTAAGCGACTACAAATCCTTTTCCGTGTGTACATATCCGTTCGT[T/A]TATTTACGTGTATGTATATGTTCGTATGTATGAGTGTATCTATTTCCATTCACGTGTTCGCTAGCATATGTATTGTATACCCATAGAAGTGTAATTCTACG |
| comp105592_c0_seq1:3919-3920 | comp105592 | 3919 | C | G | 34 | 0.264706 | 0.735294 | 66 | 0.939394 | 0.0606061 | TTGACATGGTTATTTGTTTAAGCCGATTACTTCGCTTTCATTTCATTATTATAATTATTATTTTATTCATATTTAGTCATTTTCGTTTTATTCTATTTAAA[C/G]AGTTTTGTAAAACAACGTTGTCTACGGGAAAATGAAATGCATTAGAGCGTCGATACTATCGATGCCTTTATTATTTAGAAACTACTATCAATCATTCTTCT |
| comp106255_c0_seq12:2627-2628 | comp106255 | 2627 | T | A | 222 | 0.873874 | 0.126126 | 256 | 0.199219 | 0.800781 | TCTCTCCTCATTATCAGCCATACATTAACGGTAAGTATGATACTTATCTCAATTTTGTGCTCTACTGACATGTATTTGAGAAAAGGCAATAAAATATATAT[T/A]TTCTAGTGCACAATATTGAGCTCTTCAAAGGTCTGCAATAAAATTTGACCTAAATGAAATGAAAATCACGCCTTTTTTGTTGATGGCAGGATAGACAAGCT |
| comp101299_c0_seq4:1716-1717 | comp101299 | 1716 | G | A | 66 | 0.924242 | 0.0757576 | 8 | 0.25 | 0.75 | CAGAAAAAGATGAAGGAAAAGCAAAGAAGTAGGACACTAAGAAGTAGAGGAGCAGATGCAAAGTAGAAGTAGAAGCTAAGAGGAAGAAGAGGACAAAGACA[G/A]GGAAGAAATAGAAGTGAAAGGGATGTGAGGAGGAAGTGCAACACTAGAAAGAAGAAAATGTGTGAAGGAGAAGAGGAAAAGGAAGATGAGAAGGAAGGGAT |
| comp107629_c0_seq24:230-231 | comp107629 | 230 | A | G | 66 | 0.924242 | 0.0757576 | 60 | 0.25 | 0.75 | TGATGATATATACCCTGATTTTAATCATTACTCTTCAAAATATTCTGATTTTCTTTATTCTGCAAACTTACACAGCTACTTCAAGACAAACACATAAACAC[A/G]CATTAGCCATCCCTAACCCCTGTGGACTAAAACACCTTTTAATCTGAAAAGAATGACTCATAAATAAATCTTCCCTTTACAACTTTAGCCAGCCTTTATGT |
| comp103231_c0_seq4:221-222 | comp103231 | 221 | G | A | 73 | 0.69863 | 0.30137 | 41 | 0.0243902 | 0.97561 | CGCAGAATAAGGCTATGAGTGATTAAAAAAAAGTACATTATACATATGCTGAAATCCTTATTTTTCATTGTATTCATTCATCGGGAGACCATCTGGAGGGG[G/A]AGGACGCCAGCTCTCCGTGCCTGGGGAGGGAGCACGCGACGTCGCTGTGGCAGGACGAACGTAGGGAGGAAGAACGGGGCGCCTTTGGCATTCTGTCTGCC |
| comp105636_c0_seq6:2583-2584 | comp105636 | 2583 | G | T | 31 | 0.774194 | 0.225806 | 70 | 0.1 | 0.9 | GAACACGAAGATTCCCCAAGAACACAGAAGGCGGTGAGAAAATCCAGCCACGAGCGAGGCGCACTGAGGACCTCTAAACACCACTTCGGCAGTGACCTCTC[G/T]CACTCCTTCCTTGGAGCCTTCACTCGCGCCAGGGACACGGCCGCGCGAGATCTCGATGCTCATGTTGGCTGATGTTGTGTTGGCCATCATCGTCAGTGTCA |
| comp93183_c0_seq1:334-335 | comp93183 | 334 | T | A | 75 | 0.92 | 0.08 | 65 | 0.246154 | 0.753846 | ATATCAGACTAATTTCATTCATGTTTTATGTATATGTCCAACACCTACACAATGCTCTGACTAAAATCTTTGCAAACCTGTTTTGAAACAATGTTTTATTA[T/A]TGTGTTCAACACACAAGCAAACTTTACCTATCCTCTTCATCTGCTTAAGGTAACACAAGTTTAGCAATAACAAAATGCACATTGGCAGCCAGGGGAGGGCA |
| comp99425_c0_seq11:2080-2081 | comp99425 | 2080 | A | G | 103 | 0.776699 | 0.223301 | 68 | 0.102941 | 0.897059 | AGCAAGGGACTGCGTCGTGTAGTTCTTAGTTTCCTCCAGTGCTTGGGCTTTGTTCTCTGCCCTCGCAAAGTTCTCTATGCAATATGAAGCCACTTTCTCCA[A/G]GTTGGCATGAGAGTCCGAGAGTGACTGCCGTCCCTCCGGAATCTCCTGCTGGACCAGCGACACAAACTCCGATGACAGAGCCATGGCTGCCACGTCTTCTC |
| comp104570_c1_seq1:878-879 | comp104570 | 878 | G | A | 217 | 0.921659 | 0.078341 | 258 | 0.248062 | 0.751938 | GCCAAGAAGGCTCGTGAAGAAAACGCTGGCCTTCGTCCTGCTGCGGCTGGACCGGTGGACGAGGCCGAGAGGGAGAGGGAGAAGCTGCGACAGGATCGCTC[G/A]CGCGACAGGAAGAGGGAGAGCGCCATCGCTAACTCGGCGGCTGACAAGCGCGGCAAACTGACGAGTCGCGAGCGCGACGTGACGGAGCAGATCGCTCTGGG |
| comp95790_c0_seq1:221-222 | comp95790 | 221 | C | T | 23 | 0.782609 | 0.217391 | 64 | 0.109375 | 0.890625 | ATAGAGGACTCTCATACACAACTTTTACAAAATCCCTGTTTACCGGTTTTAAGTTCTGTAACTGCGGTACAACTGAATCTCTCACTGTAATCCTGTGACTG[C/T]AACTACCCATTCCATCATTTGGTGTTTTTCCTCGATTTTACATATATTAATTTCATTTATGTTGTTGTTTTTTAAGGTTGTTTTTTTGACGTATATTATAA |
| comp98518_c1_seq4:251-252 | comp98518 | 251 | A | G | 86 | 0.883721 | 0.116279 | 76 | 0.210526 | 0.789474 | TTGCGAGGATTTTAGCGTTCACTTGAATTAAGTTTTGACTTTTATTGGGGAACTTCCTCCACCTCGAGGCGTAAAAGGAAAACTAGCGCTCGCCTTTGTTT[A/G]ACTCTATGTCAAAGATAACATTATGAGAAAGAAAAGACTTTTGAAAAGATCTGAGATGACAGCAAAGCGGATTTTGAAAGAGCGATGACAACCGAGGGGCA |
| comp107369_c1_seq1:681-682 | comp107369 | 681 | G | A | 311 | 0.700965 | 0.299035 | 36 | 0.0277778 | 0.972222 | ATGGTGTGTTCGGTCTGGACAGTTTGGAGTCGTCACACAAGATCAGCGTGCGCGTCGATCACCCAGACGAAATCGCCGAAGTCTTTGATGGAATATCCTAC[G/A]AAAAGGGATCGTCTATCATCAGGATGATGACCCACTACCTCACTGAAGCAACATTTAGGAAGGGGTTGACCAACTACCTGAAAGCACTTTCATACAAGAAT |
| comp99145_c4_seq1:414-415 | comp99145 | 414 | T | C | 81 | 0.901235 | 0.0987654 | 57 | 0.22807 | 0.77193 | CACAAACTCGAGCAATATCTGCCTGTTGTTCCTTCGCTGCCAAAGCCATTATGAGGTTCAGTGTAATTAGAGCTCCCAGCTGTTGGTAAAGTCTGTCTTGT[T/C]CTGTCTACGTTGAGTCTGAACTCATCCTGGAGGTTCGGGTGAGACCAGGAGTCATCCGTGTCCGGGTAGGAAGGCAGGTCAGACCCGTACCTGGTCTCCGT |
| comp98731_c0_seq2:843-844 | comp98731 | 843 | A | C | 329 | 0.735562 | 0.264438 | 16 | 0.0625 | 0.9375 | TCGAGGCCCTCGCCGAGTTCTTTAACTCGTGGAATGTGTTTGCTCGCGACGGGCTGAGGCCGGGCGGCGAAGCGAATTCCGACAGCGGAAACCCCACGCAC[A/C]GGGATGGACGGCCGTCGGATAAGGGACCAGTCAACGTGAACCAAGGCAAAATTGAGTAAAGGTATACCTGTGTCTGTATACCACGCTCACTAGTAAAACTT |
| comp106037_c0_seq2:936-937 | comp106037 | 936 | A | G | 71 | 0.887324 | 0.112676 | 84 | 0.214286 | 0.785714 | GGAACAATTGGTTGTCTACAGGCCCTTGAAGCTATTAAGATTATTACTAACATTGGATCACCATTATCTCAAAGTCTCCTTCTCTTTGATGGCTTGTATGG[A/G]ACATTCAGGACTGTAAAACTGCGTGGTCACAAAAGTACCTGTGAAGTGTGTGGCGATAATCCTACCATCACTAAACTCATAGATTATGAACAGTTTTGTGG |
| comp105746_c0_seq1:109-110 | comp105746 | 109 | C | G | 63 | 0.873016 | 0.126984 | 35 | 0.2 | 0.8 | ACAAAAAAACATACATTTCCGTGTTCAATGCATTTTGAAAACCTAAAAATTGTGCATTCTACTTTCAGTGAACATTAATACCATCCATCAGTCAAAATAAA[C/G]ATTATACAGAGAAATGCCATGAAAAATTAATATACACACTAGCCAACCCTTACCTCACTTGATAGGGACAGAAAACAATGATAGATAAACATATGAGAAAT |
| comp103929_c0_seq11:449-450 | comp103929 | 449 | C | T | 96 | 0.739583 | 0.260417 | 30 | 0.0666667 | 0.933333 | AAACACAAACGCTGTTATAAGGAAAATCTGAAATCGATATCTCACAGATGTACAAATGCATTTTCAATATTTTGCAGCATCGAATTATCGCCAAGACCTTA[C/T]ACACTGTATATCGATATCAGTATCTTGTCCTTTGGAATGTATATAGACGTCACTATTCCTGAGGATCCAGCACGGAATACACCAGTCACATTTCCATTTTC |
| comp107756_c1_seq6:3025-3026 | comp107756 | 3025 | A | C | 73 | 0.849315 | 0.150685 | 34 | 0.176471 | 0.823529 | CTGGCCAGGATGGAGCCCCGAGCAGCACCGTGCGGGAGGCTGACCTTGGTGATTTTCTTGCTGACGTCGCCCTCGTACAGGTTCTTCAGTTCGGCGCTGTC[A/C]ACCGTCATGGACACTCGCGGCTTGATGGACGCAGAGTGGAAGCTGTAGGACACGCGGCGGCCGGTCGGGGGCCTGTGCTTGGGGATGATAAGGAACGTGTC |
| comp106414_c1_seq3:347-348 | comp106414 | 347 | A | T | 79 | 0.886076 | 0.113924 | 136 | 0.213235 | 0.786765 | AGCATTGGTAAGAAGGATAATATCTTTATTAATAATAATAATAACAACAACAGCAATGATAAACCTAATAATCATAATAATAACCATGATAATAACAATAT[A/T]ACAATCAAAGAGTATAAAGTTTCCCATAAAAGGATTACTGCCAATAAAGATAATGTTAAGGAAAACAGAAACAGAAGAATAAAATGAAGTTGAGTCTGTTC |
| comp98311_c0_seq5:241-242 | comp98311 | 241 | T | C | 1741 | 0.692705 | 0.307295 | 652 | 0.0199386 | 0.980061 | GGCGGCGGGTTCGGCGGACGCCCTTCTGTCGGCTTCGGAGGAAGCTCCTTTGCTCAACCTTCTTTCGGCTTTGGAGGAAGCTCCTTCGCACGCCCTTCTGT[T/C]GGATTCTCTCGGCCTTCTGTTGGCTTTTCTCGTCCTTCCGTAGGATTCAGCAGCGCCTCCTTTGGGCGTCCTTCTGTGGGCTTCGGCGGGAGCTCCTTTGG |
| comp105868_c0_seq1:2209-2210 | comp105868 | 2209 | A | G | 125 | 0.704 | 0.296 | 32 | 0.03125 | 0.96875 | AACTCTCCGACGCGGTTGATGATGGCTGTCATGCGCACAACCGGTCTGTCAGGCTCCAAAGTGCGGTTGTCATAGTAGGCGCCGTACAGGTGGAATTTCCC[A/G]TTCGACGTCTCCACGGTCTGCCAGTACATGTTGTTGTAATGAAGGTCGTACATCGATGGGAAGTGAGCACAGGAGTTGTTCATCTTCTTCTTCATGTGGCT |
| comp106811_c0_seq16:131-132 | comp106811 | 131 | C | T | 86 | 0.872093 | 0.127907 | 60 | 0.2 | 0.8 | GGAAAAACCGTTGATTTATCGTATCTTACTTTTAATTTTACCTTGTTAATAATTTATGTTGAGTGGTAAAAATTAGATTTATTATTTTTCTCTGCAAATAG[C/T]GCGGGAAAATATTTTTTATGGACCTATGTAAATAGACAGTTCAAAAGAATACATTATTGTATATAGTTCCCTTGCTTCTATGTTGTGGAGCGGTGATGGGC |
| comp95622_c0_seq14:1770-1771 | comp95622 | 1770 | T | C | 272 | 0.823529 | 0.176471 | 158 | 0.151899 | 0.848101 | CCCGTCTTGGCAATCATCGCCTCCACCGGGTCCTCCTCGTCGTCATCCTCATCACGGGGCGTCCGGGAGCGGTTGTGGGGGTCACTTGCCATTGCTAAGGA[T/C]CTTTCTGCCTTTGGCATTCATCTACTTTTCTGCTCTTTAATATCCTCCCTTCCTTCCGATTCCTTTTCTCCCTTCTCCTCCCTCTGCTTTCTTGTGTTTAC |
| comp104824_c0_seq1:1568-1569 | comp104824 | 1568 | T | C | 1299 | 0.698999 | 0.301001 | 582 | 0.0274914 | 0.972509 | TACTCGACGATATCGGTGAGGAGATTAGCATCGTCCAACTTGAGTAAGTTTCCGTTCACGAGGTAACAGAAATCCCGCATCTGATACCAGGTTCCCTCAGT[T/C]TGGGGCTCGACAAACAAACAGCGATTCCCAATGGGAGTATAGGGGCTATAACAGACTGAAATATCAAGTAAAGTTACCCAAGAAGAAGAAGCAATTAATAT |
| comp103455_c0_seq6:2336-2337 | comp103455 | 2336 | T | C | 53 | 0.830189 | 0.169811 | 63 | 0.15873 | 0.84127 | TGTGGACGAGTGTTTGATAAGAAGAAATACCTTTACACACATCACAATGTACATTCTCGCCAGAGGAACTTCCCATGCAATGTTTGTGGCAAGCAGCTAGC[T/C]TCCCGCTTGGCACTTAAGAACCACAATCTCATCCACACGGGTCAAATGCCCTTCAAGTGCCCACTGTGTGACAAGGAATTCCGAAGCTCGTCCAACCAGAA |
| comp101603_c0_seq1:338-339 | comp101603 | 338 | C | T | 290 | 0.0724138 | 0.927586 | 39 | 0.74359 | 0.25641 | TGAACACGTGATAAAGGGTGTTGTGAAATATTACGGATAATTATAGCCATCAAAAGCCACAGTCCAGGCGGTGAACAAATGTTATGAGGGAATGAAGTGCT[C/T]GAAGTAAACTGTTCAAAAATGGTTTATTAGTAGACTTGGGATGCAGGGACAAATTGATGATAACTCGGCACAGATATATACATATACAAACAAACTTTCTC |
| comp107617_c0_seq2:4025-4026 | comp107617 | 4025 | T | G | 65 | 0.861538 | 0.138462 | 21 | 0.190476 | 0.809524 | GACAGATGGGAAGGTGATCTTGACGTCATAAACATCGTCAGAGTCAGGGGTGGCCTGCCTCTTCGCCCTGACGTCGACGGCCTCGCGAGCCAGCCTGCTTC[T/G]TCTAGAGCAGTCGACAGCCACGTCAAGGCCGTTGCAGATGCCGGTGGATGTTGCAGTGTCGGTGCAGAAGTTCCAGTTGGCATTGAGCTTGTTCAGTGCGA |
| comp106876_c1_seq2:933-934 | comp106876 | 933 | A | G | 172 | 0.883721 | 0.116279 | 141 | 0.212766 | 0.787234 | GCAAGCCTCAATCAGGAGATGAGTCAAGTCTTAAAGAGAGCCAAGGATGCAGAGAGAGAAACACAACAGTTACGGACTCAAGTATCTGGACTCTTGAGCCA[A/G]CAGTCATCAAGTGACCAAATTATGCGTGAGATACAACAGAGGGAAATGGATTTGATGGAGTCTATTAAAGCTAAGGACAGCCAACTGGCAGTACTTCGAGT |
| comp106656_c0_seq11:293-294 | comp106656 | 293 | T | C | 276 | 0.880435 | 0.119565 | 253 | 0.209486 | 0.790514 | TACATAACGTACGCTGGGAAAAGGAATCATTCTCCGGGTGTTTATTTCATGATGTCATGGATGAAACTCTTTTCGGCGGCTATAGTAATAGCGGCCTTGCT[T/C]TACAAAATAAGAGAGAAGTCCATGGCAGAATCTCCCATGCCAGAACTCGATCCTGATCCATGGTGGGGACCCGGAGAGCGCGAGAAGGGAGATGATTCTGT |
| comp102718_c1_seq5:215-216 | comp102718 | 215 | T | A | 158 | 0.0759494 | 0.924051 | 300 | 0.746667 | 0.253333 | ATTATCCAAAAATCCAAGTGGAAGCAATGTGTTAAAATACTATTCTAAAATAAGAACTGGAACGGGTTATACGAAATAGTGTGAGGTAAGAATAAAAAAAA[T/A]TAAGCACGTTATTGTATTTTGACTAAACTAGAGTAGTGTAACAAATTATTCCATTCAATTGTGGTTCGTTAAACTTGAAAAATGACTGTTTCCGCTGTACG |
| comp105023_c0_seq1:4280-4281 | comp105023 | 4280 | C | T | 53 | 0.943396 | 0.0566038 | 44 | 0.272727 | 0.727273 | GTTGATCACACGGCCGCCCTGACACGCCCTTTGCCACTGCAGCCCTCTGGCTATTACACGCCTCCTGTAATCATAGGACTGGAGCCCATGTGTACCTTTGT[C/T]GGGGGGATCAATGCACCAAAAAGACTCACTCTAAAAACGTCTGATGGGCTACAGCAGTACGAACTTCTGAAGGGGAAAGACGATATCAGGCAGGATGCAGT |
| comp107836_c0_seq1:2588-2589 | comp107836 | 2588 | G | A | 362 | 0.687845 | 0.312155 | 403 | 0.0173697 | 0.98263 | CTAAGCATATGGATAGCTGGGGTGTCAGCATTTCTGGATATTATGTGAAACAGTGTACTTGAATGAATTTAGAATAAAGATATGCTTCCATAGAAGTCATT[G/A]TGTACTTTTGTATGGTATATAGATTAAACAAAACCAGCATAAAATGACCAAGAACTAGAGCCAGAAAAGATGTTTTCTTGTTGAAAAGTTCCTAATTTGTT |
| comp103694_c4_seq1:3877-3878 | comp103694 | 3877 | T | G | 87 | 0.747126 | 0.252874 | 39 | 0.0769231 | 0.923077 | ATTAGCTTAGTGCTTGTCCAAAACAGATTACTTTTTGTGCCCATATACAGGCACTCCACATTAGATAATTTTATGAATTAAGAATAGATTTTAAAATGTAA[T/G]TCGTTTATCTTTATTCAGTAGGTAACACTACAAATTATAACTGGCATTAAAGCAAACATTTTCATTACTGGAATATGGAGTATAGGTTTCTATCTGACTAA |
| comp105558_c7_seq1:1315-1316 | comp105558 | 1315 | G | T | 338 | 0.718935 | 0.281065 | 266 | 0.0488722 | 0.951128 | TGGGGATGAGAGGAAAGTAATGTTATCTAAACATATATATTAAAAAACTCAGATTAGAGTGGAGTTTGCATGCATCGCATCAGTTGTTTTAACTAGATAAT[G/T]ATTACATACTCCAGGGAAGTATACATTGACTTCTCTACCATTACCTCTAAACTCAATTAAGAAAGACAGTGTTAAGGAAATGTGTTGTTCATCCTGTATTA |
| comp107347_c2_seq11:542-543 | comp107347 | 542 | G | A | 138 | 0.268116 | 0.731884 | 209 | 0.937799 | 0.062201 | TGCTCAATTATATAGACAATGTACAAAAATTATGGTGCTTCATGTAAATTGAGTTGATATTTCACGTTGATTATTTGAAAATGCATTCACTGATATGTAAC[G/A]GGTATGTAATGGACCAGAGCTTGATTATGCCAGCGACTTAAGTTTGATATATGCACAATTCTTGTCTTTCATGTAATACAATGGTCAGACAAAATACTTAT |
| comp96662_c2_seq1:389-390 | comp96662 | 389 | A | G | 200 | 0.74 | 0.26 | 227 | 0.0704846 | 0.929515 | CCTGACGAGGGCGATGTCGTTGGCCACGGTGAAGGAGTCCCAGTCCTCGTGGGCGAAGAAGTCACTTGAACTCATCGTGACTTGGGTGTCCTCGGCCTCGA[A/G]CAGGTTGTGGGCGCCGAGGACCACCTCCACGGAGAGGTGTCTGTCGACGCAGTGAGCAGCCGTGAGGACCCACTCGCTGGAGCTGAGGGAACCGCCGCAGA |
| comp100555_c1_seq11:546-547 | comp100555 | 546 | G | A | 1332 | 0.876877 | 0.123123 | 53 | 0.207547 | 0.792453 | CCCATTATTTCTGATGAACTTGACGAGGTAGTACATGAAGTTTGCGTCATCGACCTTGGCTAGTTTGGCCCCCATGTTAGAGCACAAGTTCTTCCCCTCGT[G/A]CCACGAGACTTCCTGCGTGCGGAACTGGACGCAGCGGTCGTAAATGTTGTGAAAACCTTCATCACAAGCTGAAAGATCGAGAATACTAATTGACATCCATG |
| comp107103_c1_seq11:1725-1726 | comp107103 | 1725 | A | T | 343 | 0.804665 | 0.195335 | 369 | 0.135501 | 0.864499 | TCTCCTCTGCGCCAAATGTCCGTGAAGGAGCCATCACCTTTGCTTCAGTATTCAGTGAAAGAGGCTTCACCATTCTCCCAGGTGCCTATAAAAGAGCCTTC[A/T]CCTCTCCGACAAATGTCTGTGAAGGAGCCATCCCCTCTGCATCAGTATTCAGTGAAAGAAGCTTCACCTTTCTCCCAGGTGCCTATGAAGGAGCCATCGCC |
| comp104246_c1_seq1:1505-1506 | comp104246 | 1505 | T | A | 100 | 0.76 | 0.24 | 44 | 0.0909091 | 0.909091 | GATTAAAAAGAAATCCAAACCACTATTCTCAGGATAATGCGAAGAAAACCCTCTACCAACAATGCAGAATACTTAAACGAAAAAAAAAATATTTACAACGT[T/A]ATCAGCATGAACACTTTACAATGCCCGATGATGAATAATGAAAACAGGAGGAATAATTATGCTCTGTGAAAAGCGCAACGCTATATTAATAAACCGTACCA |
| comp106030_c0_seq4:920-921 | comp106030 | 920 | C | A | 1085 | 0.908756 | 0.0912442 | 755 | 0.239735 | 0.760265 | CCGGGGTAGCGGTAAATACCCGCCACGCGAATGCCTCCTTCGGCGCCGCCCATCACTTTTCCTCCCTTAAACCGCCCATTGTAGCCCCCAATCCGCCGCCC[C/A]TCCAGATCCAAGGCCTCGAGATGGGCGCCCTGATCCGAAGTGAAATATACGATGGTGTTGTCCTCGAGGCCCAGCTCCTTGAGGGCGGCCATCAGAGCGCC |
| comp107914_c0_seq1:5854-5855 | comp107914 | 5854 | T | C | 77 | 0.727273 | 0.272727 | 135 | 0.0592593 | 0.940741 | AGTTTATGTAGGTTTTTACATTTTGATGTGGATGTCTCTGAAGTAGGAGACCTTTTTCCCTGGAACTTTTTGTTGTTTGTAGATATTGATTTAGGGTCTTG[T/C]ATGGTTTCTTGTTATCTATAACTTACATATTAAAGGGAATTTATGTAATGACTTTCCTTGTGATAAAAGGACAAAAAGAAAACTTGACTGAAGTAGCTTGG |
| comp101013_c4_seq40:573-574 | comp101013 | 573 | A | G | 54 | 0.925926 | 0.0740741 | 31 | 0.258065 | 0.741935 | AGTTTAAACCTTAAAGTTTTCTATAAAAGTGTACCACACGAGACAAATCAACAATCACATCATCACACAGGCAAATAAGAACATTTTGTGAATTACAAGGA[A/G]AGAAAAAGAATTTCAACTTTCTTAATGTCTTTTGTCTCTTTTTGTTATTTAGATTGGGCCGCATGAATTTTATTTGTTGCTTCTCGGACTTTTTCAGAAAA |
| comp103209_c1_seq1:569-570 | comp103209 | 569 | A | C | 61 | 0.934426 | 0.0655738 | 15 | 0.266667 | 0.733333 | TCGAAATTTCTGTTGCTGAATACATCGTACACATTTGTTTGTTTTACAAGCGTTTCAATCGGTGTTTTAGATATTTTCTATTTTCTGTTTTATTCTATTTT[A/C]TTTCCCTATGGTTTAAGTCCAGTGTTTACTGCTTGTTTTACATTATGCTTTATGATTTTCGTTTGAGTTTTTGGTTCTCCTTTAAATCCGGATCCAATATT |
| comp107931_c0_seq2:3659-3660 | comp107931 | 3659 | A | G | 65 | 0.707692 | 0.292308 | 25 | 0.04 | 0.96 | CAAGAAGATTATTTACATAAGCCTTTTCTCACATTATGTATTTATTTTTATTGTTTTCCTATAATCTTAATCATTTTTTTCCCATTATTTGTTTCACATTC[A/G]GTTGATTCAAACAAGTCACTGTGAACTCAACGACAAAAATTCTTTATTGACTCATAGTTTGCCTGCGGACTCTGCTGTGGCAAAAGTTATTGTACTATGAA |
| comp104998_c1_seq1:292-293 | comp104998 | 292 | C | T | 349 | 0.885387 | 0.114613 | 179 | 0.217877 | 0.782123 | TATTGTGACTAAATGTAAAGCAGAATAAATATATAATATGACTTAATACTGCACATCTCCCTTCTGTAAATGCAAAAAAGCTTCTATTCTGATAAGATTAT[C/T]AACTTTTTTCCTTATATAACATGAGTGTTTTAAAATAAGATTTCTAATAATGCTTCCTTCTGCATTTTTAAAAGGTTCTCAGATTTCTTTCACTCAGTTTT |
| comp105749_c0_seq1:1694-1695 | comp105749 | 1694 | G | T | 384 | 0.684896 | 0.315104 | 230 | 0.0173913 | 0.982609 | ATATTGCTCATTAATTAGGGTGCGCCGTTGTCTGTGGTGCGCCTTTGCCAAAAGAATTAGGTATTGTATTGTGTTCGTTCTGCGTCTGCAAGTCAGGCCTG[G/T]GAACTGGAGTATTTATGTTTTTTGTTCTAGAAAGTGATGATGTACCTACATTTGCATTTTGAAGTGTTCTGTAGAAGTTGCAGATGAAATAGATATATATA |
| comp106663_c1_seq7:963-964 | comp106663 | 963 | A | G | 51 | 0.705882 | 0.294118 | 26 | 0.0384615 | 0.961538 | GATATGACAAAGGAGAAGCCGAAGAAAGGACATAGTGAGGGAGATAGCGAAGAAAGGACTCTCGAAGAAAGTGCGGAGTACTCCTATAGCGAAGGACTAAC[A/G]TGGAGGATGGCAGTGAAGGAGCGACTGTGGAGTTGAGGTGTTCCCATCCCCAGAATTCGAAGAGACTGTGAATTCTGTATAGGTGTATGTTACACTGAAGA |
| comp106456_c0_seq11:3619-3620 | comp106456 | 3619 | G | A | 80 | 0.725 | 0.275 | 52 | 0.0576923 | 0.942308 | ATTGAACTCCAGTTATTTGCTGATTAAGGTTATAGCAAAGCTGCTATTGAAAGTAGCCATTAGATGTAGTTATGGGTCTCTATAGCTACTGTTTTCTAAGT[G/A]AGAACTAGAGTTCTTGTTAACCACATGTTATTCAGAAATTCTACTATATATGCATTTATGATAACTCAGAATAACATCAAAAAGGAAATGAAAAATATTGA |
| comp107029_c1_seq2:2070-2071 | comp107029 | 2070 | C | T | 292 | 0.880137 | 0.119863 | 108 | 0.212963 | 0.787037 | AAATAGAGACATCTGATACAGCAAGGAAGTTTAAGAGAGAGAGAGAGAGAATTCTAAAAAAGATCTGAACAAGTCGAAGGTGATCAGGAGCAGTGCAATGA[C/T]TGATATACGGAATTACTTCCAAAATTGATAACAGGTCTAAAGCGATGAAATCTGCTAGCAGAATATTGAACACAATATTCTAGCAATGTTTACTCAACTTT |
| comp107912_c0_seq2:2257-2258 | comp107912 | 2257 | G | C | 772 | 0.923575 | 0.0764249 | 152 | 0.256579 | 0.743421 | AGTAAACCAAAGAGAAGAGCCTCCATGGAGCACTCTCAGTGGCCAGAAGGCTGCGGGAAAGGCTTCGGGTGTTCGCCCGGCCACAGCAGCTTTCGTGACGA[G/C]AAAAATGTCCTAAAGGACGCCGAGACGCTGGGCATCGGAACCTGAGAATCCTTGGTATGCTGTTATACCTCTAACTACTTTAGTCATTTGTCGAAATGTAT |
| comp106664_c0_seq1:884-885 | comp106664 | 884 | A | G | 70 | 0.742857 | 0.257143 | 79 | 0.0759494 | 0.924051 | CAGCACATCATTAAGGTTGTAGAAGCATCACGTGTCCACCTGTTTGATATTGTTACACAGTATCGCGCCATCTTTTCGGATGATGACATGCGTTTCAGCCT[A/G]GGGGACCCGGAGGTCAATGAGCAAGCCATTTTCCATGGGTGGATTCTGCATAAGGTAAACCAGTTCATGAAGATTGTGGAGAGGGACCTTGAGCGAGGTGT |
| comp103464_c0_seq3:781-782 | comp103464 | 781 | C | T | 94 | 0.87234 | 0.12766 | 107 | 0.205607 | 0.794393 | CATGATATTTACAACATAATCACACTCCTTCAATAGGGATGAAAGCTCTCCAGTATGCCAATATTTATCAACATTACTACATCTATTCTCATCACTTGGGA[C/T]TGAGCGGCTGTAACCATGCACAGTGCATCGGAATGCTTTGAGGCATCTTGCAACTTCTTTTCCAATATTTCCCATTCCAAGTATTCCAACAACCAAGTCCT |
| comp101932_c0_seq1:968-969 | comp101932 | 968 | T | C | 117 | 0.666667 | 0.333333 | 1 | 0 | 0 | TAATTATTTATTATAGTAAGGAGGAATAAGGGAACTGCGATAAGAAATAAATAGATAAATAAAATCAGCTGCATTATACCACCTTAGTAATAGATATAACA[T/C]TATAACAAGAACATTATCCATGTGAGATTGCTTTTCAAAAGAACACAATCCACTACCAGATAACATGTCACCTTGGATATTGCAGAGAAGACACGACTCTC |
| comp102725_c1_seq4:868-869 | comp102725 | 868 | G | T | 2 | 0 | 0 | 57 | 0.666667 | 0.333333 | GAGGGAGGGTTGGAACAGCGTTGCCAACACCCCCGTCGCCAAAACCAGCGTTGCCATTCTCATTCCCGCCATTTTGGTCTCTTGTTCTTCTGCGGCGGTCT[G/T]TAGAATGTCTTCTCTCTGTAGGAACAACACAAAGTGGAGTGTCCCAAAGCCCAAAGTTCAAGTCTGTGGGGGTGTTTGTGCGCCTGTAGAAGACCTGTAAA |
| comp103806_c2_seq1:101-102 | comp103806 | 101 | C | T | 159 | 0.666667 | 0.333333 | 1 | 0 | 0 | TCATCATCATCATCATCATCATTACCACCATCATCATAATCATAATTATCACCATCACTATCAACATGATCATGATCACCACCATCACCATCAACCTTCAC[C/T]ATCATCATCATCGTTATTTTCTTCATAACGAAATTAGCGAAATTATTAGGTTGCGAACGCTGGGATAGCGAAACTGACAGAAACTTCGGTGCTATATTTCT |
| comp106307_c0_seq3:380-381 | comp106307 | 380 | A | G | 66 | 0.833333 | 0.166667 | 114 | 0.166667 | 0.833333 | TTCATACAACCGTCCATGCAAAACCATTCGTCTTAGCTCTTTCATGTACTATAATGCACAAGTAGAGTCATGTTTTCTTTCATTCACAAGACGAACTGTGT[A/G]TTCATCTTTCATTTCCTCAAAATGCAAATTAACGCATTTCTTTTGAGTCATAAGAGCATAAATTCTTTCAAACTGACATGGAACGTTTTAACGTTTAGCTC |
| comp102839_c0_seq1:250-251 | comp102839 | 250 | G | A | 805 | 0.239752 | 0.760248 | 940 | 0.906383 | 0.093617 | GCAGACGCCGGATTATGAAGTGAAGAGTCCAAGCGTCCGAAGCGAACGACCAGGAAAAGGAAAGACGTCAGACGGAAGACCTCTTAGTTGTCGTCATCGTC[G/A]TCGGCTTCGGGGGCGTCGCTGCGCATCCTGGGGACAACGTTGTCGTCGACGATGCGGTACCCGTTGTGGTCGGCGATGTACTTGACGTGGTACTCGTTCCC |
| comp105056_c0_seq1:1592-1593 | comp105056 | 1592 | A | C | 148 | 0.898649 | 0.101351 | 112 | 0.232143 | 0.767857 | AAGAAGAAAAGAGAAAGTTGATTGGATTAATGTATCGTTATTGGATCAGAGGCTTTATTACTTTTTCAGTTTTAAAGGGTTCAGATGTATAGAAAGGCAAG[A/C]TTTTACTTGATGGGTTTTGTATTTTAGAAAAAGTGGAAAAAAAATTGTGGATGGGTGACCATCAGGGCTACAAGTTTCAGCAGCCGTAAAAGTTGGTAATA |
| comp107727_c1_seq1:415-416 | comp107727 | 415 | G | A | 326 | 0.852761 | 0.147239 | 295 | 0.186441 | 0.813559 | GCTATTGCATGTTTTAAGAAGTTTATCAGTCTTGTCACAGGTGAGTTCCTTTCTTGAGACAACATTCAAAACACGAGAGAAACTTTATTTCTACTCTTCTC[G/A]TTACATATCCTTATCAACATTTCAAGGGATGAGTGAAATTATTTTTTCTTCCTTTAAATAATAATACAATTTTAATCATGATTTGGTCTGCATGAGAGGGG |
| comp104296_c0_seq3:1212-1213 | comp104296 | 1212 | T | C | 293 | 0.938567 | 0.0614334 | 55 | 0.272727 | 0.727273 | ACGTAAATAGATTTCCTCGGTCTCGTGTTCGCGAGTCGTCGGGTCGTTGCCATCCGAAGAGCTCAGCGATGGAGACATTGAGTCGAATGACATTCCATTGG[T/C]TGCTACTGTCGTCACTCCTGACGGTCTGAAGATAATCCCTCCCTCGATTGCCGCCGTCGTCGGTTCTCTCTCTGACGAGGACGCCGGCGGAGGCGCTGCGG |
| comp107452_c0_seq10:1017-1018 | comp107452 | 1017 | T | C | 117 | 0.863248 | 0.136752 | 157 | 0.197452 | 0.802548 | GTTTTGAACAATCGTTGTGAGGAAATCCTAAACTCCTAAATAATCAACTCAGGATATTCTTCGAAGTCATACAGTGTTAGAGAAAATAATCTATGCATTTT[T/C]CTAAATCTTTTCCTGTTTATTTTTCTGTGGTACGATATAATGATGGACACCCTCCCACATCATCATCTGATGCAATGACTGGAGAATTACAGGAGCTTCTT |
| comp104893_c1_seq2:1703-1704 | comp104893 | 1703 | T | C | 95 | 0.852632 | 0.147368 | 107 | 0.186916 | 0.813084 | CGGCTTTGACCGAAATGTTTATTTGGATTCATGTCCTGATCCACTGCCTTCATATACATGAGTCCACTATGATAATTCCTTATGGCCTTCTTCAACTGCTG[T/C]TGCTTGAAGTGGTCATTTCCCTTTTGCTTGAAGTCCACTGCTTTTGCCAATTTATGCTGGATTCTCTCTTCTGGGGTCGCAGATGACGGCAGTTCACAAAG |
| comp107932_c0_seq13:4236-4237 | comp107932 | 4236 | T | C | 26 | 0.0384615 | 0.961538 | 169 | 0.704142 | 0.295858 | AAGGTATGGATGAAAGAAAGGGTAACGAATGGAATGGAAAATATGAATGTAATTATGAAAGATATATATGAAAAAAGGGTAAATAGGTATAAAGAGATGGT[T/C]AATTGAAAGGCATGGATGGGAGGCTACACAGGAGTGGAGTAATAATGAGAATATGAAAGGTTGATAGAGTAAGGAAAAGGAATTGAAAGGTTTAGTGAAAG |
| comp83676_c0_seq1:128-129 | comp83676 | 128 | C | T | 64 | 0.765625 | 0.234375 | 20 | 0.1 | 0.9 | TGTTCTAATTTTAATTTTTTTTTCTGCTCTTTTTCTATAAAGCTATGTTTTATTATCACAATTTAATGCAGAGCAAAAAATTTATGATTATAAAACAGGGA[C/T]AATTTTGTTGTTTTATATAAAATGAAGTGCCTGAAAAAGGATTATCTTGATAGGGTAAAACATGTAAGTAAAGCCTTACCTTCATTATTGCTCCTCCCTCC |
| comp104023_c1_seq2:685-686 | comp104023 | 685 | T | C | 20 | 0.8 | 0.2 | 52 | 0.134615 | 0.865385 | ATGTATGGTATAGGCTGGGTGATTTGGTACCTTTGGTAATGTTTAGGGAAGGGGCCTGGGATATGATGAAGAAAAGATCGAAAAATATGCTGGGAAAATGT[T/C]TTATTTCGGCAACTTTGGAAGCGAATTTAGGAAATACAAAGAAGTCAGAACAATTTTTGTCTAACCTAACACCTGCGCATCGCCCATAATGTACGCACCGC |
| comp104469_c1_seq1:154-155 | comp104469 | 154 | A | T | 596 | 0.89094 | 0.10906 | 381 | 0.225722 | 0.774278 | TCCCGACGACGAATGATGATCCACGGTCTCTTCTCTCTGCCCAACGACGACGACGATCCACGTCTAATCTCTTCTAGATAACGATGACAGATGATCCACAC[A/T]CTCTTCTCCATTCCCGAAGACTACGACAGTAACGATCCACATCCTATGATCTCTTCCTAGCGACGACGACGATCCACGTCATCTCTCATCCCAGCGGCGAC |
| comp106980_c0_seq14:293-294 | comp106980 | 293 | A | C | 164 | 0.756098 | 0.243902 | 11 | 0.0909091 | 0.909091 | TTTTTTTTTATTTTTCTAGATCATTGTGCCTATTATTACAGGCTTAATAACACACAGCAGTCTAAACCGGTGTGACTATTCTTTCAGGCGTAGTAACCCCA[A/C]ATAAACGCAGCAATACACATGACCAGTATAGATGCTCCGTCTACAACATTAGTCCACGGTCTCTTCTCCTGCAGGAACGCGGCGGCTCTGGTGGCTTTCTC |
| comp102646_c0_seq2:774-775 | comp102646 | 774 | T | G | 496 | 0.149194 | 0.850806 | 307 | 0.814332 | 0.185668 | ACGACGCAGCCACGACCAACGTCATCCTCTTCTGCTGTACCCTATAACATGTACAAGACATGTTCCGTTGTAAGGGGATTCCCCAACGACTGTTATGGTTT[T/G]GGTGAATGACTGGTCGCCAACTTCAATAAAGTTTATTGCCTCTTGGACATGAAAATAAAAAGAAAAAATGTTGAATTATCGCCCGTTTTATTAAAATGTTT |
| comp102032_c1_seq1:1137-1138 | comp102032 | 1137 | T | A | 106 | 0.669811 | 0.330189 | 211 | 0.00473934 | 0.995261 | TTACAATAGTCTCCCTTCACAAGTTTTAGTATTCATTTTCTCTCAATGAATGTAAGCCTCGGGGCTTTTAATAACTAAATTTGTTTAAAAAGAAAGCTAGC[T/A]TTTTGTTGATGAGTTATACTACATATTAAACATGTCATACTAAACTCAGCAGAATGAATTGTTAACACAAAACAATACAGCTGTATATATGAATGAATGGA |
| comp104671_c0_seq26:1133-1134 | comp104671 | 1133 | T | C | 319 | 0.717868 | 0.282132 | 283 | 0.0530035 | 0.946996 | GTGGCTGACAAAAATGTCCGAGTGTCAGTTATCAGCCCGGGATATATATCTACCAACCTGTCTGTAAATGCAGTGACTGGGGACGGCTCTTCCTATGGAGC[T/C]ATGGATAGCACCACAGCAAACGGTATGAGCCCAGAGTATGTGGCAGAGCAGATTGTTGGGTGCATCATCAGTGGCTATGAAGAGCTTCTCCTGGCCCCCTT |
| comp101350_c0_seq4:103-104 | comp101350 | 103 | C | A | 52 | 0.807692 | 0.192308 | 21 | 0.142857 | 0.857143 | GGTCCTGTCCTTCTGCAGGTCCTGTCCTTCTGCAGGTTTGGGAGGGGCCTTTCGGGTTTATGGGGGGATTTTTATTCATCTATTTATTTGCAGTGGGCTTT[C/A]TCGATACGTCAAAGACCAGTTTGCGGCCGTTTGGATTTGTTTTATTTTCGATCTATCGGTTTACAAAGAGGCGGCTGTGTTTGGTTTTATAATTGGGCCTT |
| comp105117_c0_seq29:970-971 | comp105117 | 970 | C | T | 185 | 0.72973 | 0.27027 | 92 | 0.0652174 | 0.934783 | AAGAAGGGCAGGTAGTGAAGTGCCCATGCCACAGCTAGCCATCCGCAGGCTCCTATAGCCTTTTCCCGTCGGGCTCTTGTTGCAGCATCATCCTTCAGTCC[C/T]CTTTGAGTCCTATAAGCATTGTAGATATAGACCAACACAAAAGCAGCCAACACAACTAGGTTACCCCACCAAATGATGGGATTACCCAGCAGGTAAACTTT |
| comp107338_c1_seq3:128-129 | comp107338 | 128 | C | G | 58 | 0.810345 | 0.189655 | 48 | 0.145833 | 0.854167 | GGATTTCACTCCCGTTGATTCTCTCGATCTGAACAGACGACTGAGGGCGTTGAGTATCCGTAGAGTTCCGCTGGCTATCGCTTTTCACGCTCTGCAAGGAA[C/G]GAAACGGAGGGTGGTTTTAGATCCGTGATCAACGCGTAGAACGAATGGTATTAATGAGAATAATATTGTTGATTATGAGAATAATAATGATGATGATTATG |
| comp100526_c0_seq1:1031-1032 | comp100526 | 1031 | T | C | 761 | 0.914586 | 0.0854139 | 559 | 0.250447 | 0.749553 | TCACTCTGAACCTCCTCATCATACAATAACAGACAAAGATAATATACATTGATTGCACCATTTTCTTGTTTGCATATGTCTATGTAGGTTTAATGAAATGG[T/C]AGATAAGTGTATATAAACATGGCGATGTCTCTAATAGTCCTTGTCCATTTGTGTTCCGGCTGCCTTCGCTTGCTTTCATGTCTGCTTCACACACCGCCCAC |
| comp104083_c0_seq5:131-132 | comp104083 | 131 | G | A | 118 | 0.830508 | 0.169492 | 6 | 0.166667 | 0.833333 | CCCGGGCGCTGCCTGGGGTCCCGCCCGCCGTGCTGACTGGGACGAGACTCGGTTCAGGGACCAACGCTCTTTGGCTGGATTTGATGCGCCGAAGGAGGGTT[G/A]TTTGTAGTTCCTGATGGGTGATTAGTAATGATTAGTGTCATTCGTGAATTCGTGGTTAGTGTAGCTCTTTTTTTCCAATATAATTCGGGTTTATAATGGTA |
| comp101097_c0_seq5:475-476 | comp101097 | 475 | C | G | 101 | 0.841584 | 0.158416 | 45 | 0.177778 | 0.822222 | GGCTCACATTGTGGCTGTCTGCGCGCTCGAGTGGCAGATGAAGTTCCCGAGGCCAAGCGAAGACGCCAAGTCACTGAGCTCCGCGTTGTCTCGCGGTCTCT[C/G]CGTCAGGCTGATATCCAGGCGCTGCGACACGCTGACGCCCTTCTCGTGCAGCAACTCGAAGAGCCTCCTGATGCCCGTTTGCGTGAGGCTTGTGTTTTTGA |
| comp103071_c0_seq6:222-223 | comp103071 | 222 | A | G | 114 | 0.885965 | 0.114035 | 18 | 0.222222 | 0.777778 | GAATTACCAAGTGCCTAATTACCTACTGTCAAGCACTCGAGGTTGGGAATTGACCATTAACGGCTTAATGGCTACAGAAGGACGTTGTTTTTGCCTCCCCG[A/G]ATACAGTCACGGTCAAAAGTCTTGTCAAACGTTTCGAATTCCGTTTCGAACATAGGCTCAATATCACACTCTTTTGTATAGAGTAAGACAGATTCAAAGCT |
| comp99126_c0_seq1:261-262 | comp99126 | 261 | C | T | 140 | 0.821429 | 0.178571 | 57 | 0.157895 | 0.842105 | CACCCACCATCATGGAGGCGAAACAGACCGCTGAAACCTCAAGTTCCAGCTCCTGTTGCCACGACACGGCAGCGGGAGCACGTGACCAAGATTCACCAGCA[C/T]CCTGTGGCGATCAGGACGTAACGACGATCCTACCTGACGCTGCTGCCAAGTGCTCTCTGAACCTGAGCGATGCGATGTACCTGGCGGACATGGACTCCGAG |
| comp107364_c2_seq5:143-144 | comp107364 | 143 | G | A | 67 | 0.910448 | 0.0895522 | 85 | 0.247059 | 0.752941 | ATAGCAAAATGCATGACCTAAAGTTTGCTCATCGGAGATTTAGACACTGATTCTCCATCCTTGTCGTTGTCAAACATTTCACTGAAGCCTTCATCGAACGC[G/A]GCCTGAAGCACCTCTTCCACTGTGTTCACAAGTACTACTTCGATTTCATTCAAGACTGAGTTTGGAATATCAGCGAGGTCTTTCTTATTGGCAGCAGGAAG |
| comp107276_c0_seq1:3900-3901 | comp107276 | 3900 | A | C | 132 | 0.939394 | 0.0606061 | 76 | 0.276316 | 0.723684 | TTATTCCACACGCGTTTGCAAAATTATTCTCCAAAGCTCAGGACTTCAGAAGACAGCAAAAACCTCAAATTAAAAGCCTTCAAAATCGGAAAAACCCGAAG[A/C]AATTTCCTCTCCAAGAGTCTCTTCTCACGTCCTCTTCTTCTTCCACACTAAAACTCCTTTCTAACTACTCAACGTACTCCTCCACAAGGTCTCCTCTTCCC |
| comp104814_c2_seq1:441-442 | comp104814 | 441 | A | G | 130 | 0.823077 | 0.176923 | 75 | 0.16 | 0.84 | TCTTCACCGATCTCCTCCTCCACGTACTGCAAGGGGTTGTTGCCGAAAAACGAAGACAGTTCATCATAATACTTGTCCGAGAGGAAATACATCACGAGGCC[A/G]AGCACGATGCACACGAGACCAGCCACCAGGGAGAGGTACCAGTACACGCCGAAGTGGGTCGTCAGAACGTCGTCTTCGAACGGGACTTGCAGTTCGATGGG |
| comp107077_c0_seq10:301-302 | comp107077 | 301 | C | T | 51 | 0.921569 | 0.0784314 | 58 | 0.258621 | 0.741379 | GGAATTTCTAACTCTCTATTATGATTGCGTAATGAATAAGTGGCGTTGACTCTGAAATGAAAGGTATCATCTTCATCATTGTGGAGGGACCTAAACACAAA[C/T]ACAGCAAACCGAAGAGTAGAAATATCGGGATACTTTAAGATTTGAAGGTCCTTTTTTATAGAAGTTGTATGTGATCAGTCTTTTTGAGCTTTGCCATTGTA |
| comp107830_c1_seq1:166-167 | comp107830 | 166 | T | A | 99 | 0.787879 | 0.212121 | 8 | 0.125 | 0.875 | TTTCTTGTTAATAAATCTTCACAGTAATTTGTATTACGTGCAAATACACATTTTCCCCCTTCAGACGGAATCTTTTTACTGAAATAAAATGATACAGATAA[T/A]CAAGAAATGAGTAAATATAACAGGGATTACAAATACCAAAATAATCTAGATTATATTTATAGGTCTACTGATGACAGCAGTTTTGAGCAAAACACTTCAAC |
| comp105726_c0_seq7:865-866 | comp105726 | 865 | G | A | 131 | 0.832061 | 0.167939 | 112 | 0.169643 | 0.830357 | AATGCTGCAACAAGAAGGCCACAGTTATCTAGCTGCTTCCTGCTTACGATGAAGGATGACAGCATTGAGGGCATTTACGACACACTTAAGCAGTGTGCTCT[G/A]ATCTCCAAATCAGCTGGAGGCATTGGGCTGAATGTACACTGCATCAGGTCCATGGGAAGCTACATTGCAGGAACCAATGGAACTTCTAATGGTCTTGTCCC |
| comp104245_c1_seq2:813-814 | comp104245 | 813 | G | A | 511 | 0.855186 | 0.144814 | 1068 | 0.192884 | 0.807116 | TCCGAAGAATGGAAATGTCAACCCAACAAGTTTATGCCGCCGGAGTTAGAGCGCCATGAAATTCTGCTGGGATCTTTCTCGCTGCTTTTGGGGTCCACGGT[G/A]TCCGCCATGCTCGCCTGTTACGTCATGAACGGCGGTTATTCCACGATCTACTATGACGTCAGCAAGTACGGCTGGCTTTGGTATTTCGCCTCGTGGCCGGT |
| comp97969_c3_seq2:698-699 | comp97969 | 698 | T | G | 30 | 0.7 | 0.3 | 53 | 0.0377358 | 0.962264 | CACTCGTTATCTCGTACCGTTGTGTTGTTGTTCTTTTTTTTATCTTTGTTTTAACTTTACGGAAACGTGTTACCCAATTTCTGATGTGCAATCGCGTCGTG[T/G]TTCGAGTTTTTATATCGGGAAAAGCGGCATTCAAATGATAGCGAAACATGTATGAAAGCGAAAACGAAAGAACAAAAAACGACAACAAATAAAAGCGTTAA |
| comp107514_c1_seq4:292-293 | comp107514 | 292 | C | A | 38 | 0.921053 | 0.0789474 | 85 | 0.258824 | 0.741176 | AGGCCTCAGTATATCGCCAGACACACAAGGGGAAACGGCGTATCGTACCGCAGTGCTACCATGAAGGCTCTGGGCCCTGTTTTAATTGTTGTCTTCCTCTT[C/A]GGAGCCACAGCAGCCGAGGAGGTACTAGAACACCAGGAAAATGTGCAGAGCAACGCAACGTCAGCCCCAGGCGCTGTAGAAGATGGGAATTTCGGCGTCTT |
| comp105022_c5_seq1:999-1000 | comp105022 | 999 | A | G | 57 | 0.192982 | 0.807018 | 69 | 0.855072 | 0.144928 | AAGATGTACCAGTGGTGCGTCTTGAGGTTGTTTAGGTCCAGGATATCTCTTATTTGCTCGGAGGAAAGGGCTTCTGGAAGATCCTGCTTGTTAGCAAACAC[A/G]AGCAGTGTTGCCCCGGCCAACCTCTCCTCCTCTAAAAGCTTCTTGAGTTCCCCTTGGCAATCTTTCAGGCGATCAACATCCGCACTGTCCACGACCCATAT |
| comp106823_c3_seq9:658-659 | comp106823 | 658 | G | T | 57 | 0.894737 | 0.105263 | 30 | 0.233333 | 0.766667 | TTCTTTCAGCTTTCAGTAAACAAGTGTGTTGATATTTCTGTTTTTAAAAGTGTGTACTAGTATTCTTGAGGAAAAGACGAAAATCCTCAGTACTTTTCATT[G/T]AATTAGTCCGTCTAATTTCAAGAAAAGAGTAACTGGTAACTCATTGCGGGTTCAGGGACTCGTCCGATTCGCTAATTTATATTTTGAGAGATAATAATACT |
| comp104718_c0_seq2:108-109 | comp104718 | 108 | A | G | 124 | 0.66129 | 0.33871 | 1 | 0 | 0 | GTGCGTGTGTGTGTGTCTATAAACATCCTCCGTCACATAGATTCAAACAATTAAAACATTATGTTTTGCCCATTCTCTCCTGAAATGTATGAATTTCACTT[A/G]GCAACATCAATCAATGCATCTTAATCTGATTACAAAAAATAGATGTATATCGATTGCCTTAAAATGGGCATAACCGAAAAGGCGAGAGAGTAACAGAAACA |
| comp104278_c6_seq3:769-770 | comp104278 | 769 | T | C | 93 | 0.795699 | 0.204301 | 89 | 0.134831 | 0.865169 | CACAATATAATGTATATGTCATACATCATAATAACTGGAATCTAAGTCTTGTAGTATTCAACTACTTAAAACTTAAAACATGTATTCTGCATATCAATACA[T/C]TGATGCAGTTACTTAGCATCAAAGTTTAAGCCACTAAATAACTGCCACATATACAGGCAATCACAACTACAGCGTCAGCAATCAAGCTAGTTGCCTTTTTT |
| comp99666_c2_seq1:672-673 | comp99666 | 672 | T | C | 162 | 0.882716 | 0.117284 | 72 | 0.222222 | 0.777778 | ATACGTGTATAGTTTACGGACAAGTACTAGTGTATTTGCAACACCAGGCGTAATTTTGGCTTGATATAAACTGTAATCTGAATTAGATCTTTTGTACTCTT[T/C]AGCATTTTACACGCCCTGTTAATTTCCAAAATATTTGTAAGAGCATCGTTTATATTACAAGAAATTTCATTTTATTAAAAGATCTTACAGTTAAAAAAAAA |
| comp107532_c0_seq1:3483-3484 | comp107532 | 3483 | C | T | 81 | 0.753086 | 0.246914 | 54 | 0.0925926 | 0.907407 | CGAAGGAACTCGAGCCTCAAACGAAGCCGACCCGAAGAGGCTGGGGCGACTTCGAGGACCACGGCCAAGAAGGAGAGGAAGGACTCCGAGGACGAACCGGC[C/T]GACCTCAAGAGCGAGTCTATCGTGCAGGATCCGTCAGGCTGGACCAAGACCAAGACCACGGTCAGACGGGCGAGGCTCGGATCCTTCGAAAAGACACGCCA |
| comp92679_c0_seq3:541-542 | comp92679 | 541 | A | C | 204 | 0.00490196 | 0.995098 | 254 | 0.665354 | 0.334646 | AGGAGTAGACCGAGACAAATATAAATGATCAACAGATGAAAACATAATATAAACTAAAAGTATTTACAAATTCATCAAGTGGATATGAGTATGAAAAAAAA[A/C]AAAACATTATTTTCATGACTGCCTTTGTAGGTAACAAAAAAAAAGTTTGATAATATTTGCCATTGTAAACCAATAACAGGACCACAGGAAGTGCCATATTG |
| comp106754_c1_seq29:915-916 | comp106754 | 915 | C | A | 105 | 0.238095 | 0.761905 | 49 | 0.897959 | 0.102041 | AAGCATTGGTGGTTTGGCGGTGGCACGGACCTCACCCCATACTTCCTGGAGGAGGACGATGTGCGACATTTCCACTCTGTCCTGAAGTCCTCGTGTGACCG[C/A]CACGACCCTCAGTATTATCCCAAGTACAAGAAATGGTGCGATGATTATTTCTTCATCAAATTCCGGGGTGAGCGGAGGGGTGTTGGAGGTATCTTCTTTGA |
| comp107255_c2_seq10:1111-1112 | comp107255 | 1111 | G | A | 53 | 0.283019 | 0.716981 | 35 | 0.942857 | 0.0571429 | TTGCTCTGTATTCTGGTAAATTGAGGGATTATTGCACAATACTTCTCTTACTGTACACTTGAGGCTTTACCTAATGAATGATATATCTTCTTGTTCTTTAT[G/A]ATGTTCTTCATACAGATGTAATCTAGTCAGATTTTCTCCTCTATCCTCTTAACATTATTAACACCACCATATGAATCCAGTTCATATTCATATTTCTGTTT |
| comp106900_c1_seq4:1263-1264 | comp106900 | 1263 | A | G | 390 | 0.882051 | 0.117949 | 549 | 0.222222 | 0.777778 | CGCCAACAGGTAATAGAGGGTTGAAGATCGAATTCAGAATAGCACGGTCTCCAGCTGACGCCATTGTCTATACTCAAATCAAAATAGGTGAGTAGCGCCGA[A/G]AAATCAAATTCAGGTTAACTTCGCAGTCTTATTCACTCCTCCCCACTGTGTCTCAGTCGCTACTACCGTGTTGTTTGAGAGCCACAACACCGCTGCTGACT |
| comp103400_c0_seq37:129-130 | comp103400 | 129 | T | C | 68 | 0.926471 | 0.0735294 | 45 | 0.266667 | 0.733333 | TTGATGTGCATATCTAGACATTTAAAAACAGCTCTCTAGTTTCGCCCTTTTTCAGAGGAACCCTACAATTTCACCTTGACCTACAGCCACTCGGCCAATTA[T/C]CCAGCTGATGTCTACTACATGATGGATGGATCTCAGTCCATGGCAGACGACAAGGACATGTTATATTCCCTTGGCGAACAGCTGGCAGAAGGGATGAAGGA |
| comp107755_c0_seq2:1330-1331 | comp107755 | 1330 | T | C | 124 | 0.274194 | 0.725806 | 106 | 0.933962 | 0.0660377 | ACTGGAGATCTAATATGTTATTATTTTTGGATATTTAATACTTAAAATCAAAGTTATTAGCAATTTTCTGGTTGTAATTGACTTAAGAGGGCCAAGAAGCT[T/C]TTTTATCTTTCAATCTCAGTTCAGTATTATGTACAGTTGAGTAATTCCGATAAATATCAGTAACAAAATATAAGAATAAAAATAAAAATAGTATATGTCAA |
| comp101062_c2_seq1:485-486 | comp101062 | 485 | C | G | 32 | 0.9375 | 0.0625 | 72 | 0.277778 | 0.722222 | TTTTGTTCCTCTTCCTTTCTTCCTCTTCCTCCTTTTTTTCTCCTTCCTTTTACCCTGTTCTTCCTTTTTCCTCTTCTTCCCTACTGCTCCTGCTCCCTTCC[C/G]TCTATTCACATTAAGTGTTTTTCCTTGACGTCTTCCTCTTCCTCTGTTCTTTTGTATCGTTCGCTTTATCTCCTCATTCCGTTTTTGTTTTTTTTTATTAT |
| comp102299_c0_seq2:383-384 | comp102299 | 383 | G | A | 70 | 0.742857 | 0.257143 | 12 | 0.0833333 | 0.916667 | TGTTCACACAATTTCGTGATCATTTCGTGATCGTTGATTCACTTCTTCGTCTTGTCACGTTATATATATTTTTAAAAATGCTGTCCATTTCATAGGACAAT[G/A]TGATGCTGAAAGTTGGCTTTCTTTAGCATTGGATATTAATATGTAAAAATTATGATCTTTTAATTCCATTGGTGTCAGTATTACCAATTTTCTTAAGTCGA |
| comp105756_c0_seq2:2510-2511 | comp105756 | 2510 | T | C | 64 | 0.859375 | 0.140625 | 30 | 0.2 | 0.8 | TCACAGAAATTATTGAAGCCGCCTACACCGGTTATGGCTTCATCACCAACGTAGCCATCGAAAGGCTCCGTGTGAAGCACAGGTTGCGTGTAGTGCAGACG[T/C]TGGAGGACACGGTAATGAGGAATACCCTTCGCACAGTGGGACCAGATTGTATGCTAGGGGAAGAAGATCTCAAGGAATTAGTGGTATATGTACGAAGTGAA |
| comp93388_c0_seq1:399-400 | comp93388 | 399 | A | C | 77 | 0.805195 | 0.194805 | 48 | 0.145833 | 0.854167 | TATTTTTGGCTGATAAGTTATTTTCCATACACTTCCGTTCTGCATGATTCCTAGTTTGCTCGTGCACCCGTCGGGCGCCACAGGAGAGCCTCCCCCGAGGG[A/C]GCCTACTCCTCCCGCTTGGCCGTCAGGTGGGGGATCCTGACGGGCTGCCCGATGGTGAGCGTGCCCGTCCCCAGGCCAGAGGGTCCGATGACCACGCTCTG |
| comp101444_c1_seq2:492-493 | comp101444 | 492 | T | C | 2533 | 0.273589 | 0.726411 | 2323 | 0.932845 | 0.0671545 | GAAAAAAAAAAAAAAAACATTCTGAATATAAATAAATTTCATATAAGGGATGACTAGACTCCTCTTGATATTCAATTTTATTTTTAGCAGAGCCAAATTCA[T/C]TGAGTTGTGTCAATACTGCCTCATGTTGGCATCCGTTCTATCACTTTGGTTCACCTAATACAACAGCAATCTACTCCTACTCCTCCAAAAAATGATTTCCG |
| comp102919_c0_seq1:1461-1462 | comp102919 | 1461 | C | T | 77 | 0.909091 | 0.0909091 | 24 | 0.25 | 0.75 | TCCTTTGGTGCTGACAGTAAAGAAAGGGCCCTGGAACGCTTCCTTCTGCCCTTGTTGCTTGCCTCCATCAAGAGGGAGATGTCTGGGCTGTCTTGGGCTTC[C/T]GTTTCAGCCTCTGCAATTTCATCATCTACACTTTCAAACACATCCCCTCCACCTTCCAGGACCATTTCCTCTTCTTCGTCCAGTAATTCTTTTGAATCCTC |
| comp105731_c0_seq1:1385-1386 | comp105731 | 1385 | T | G | 64 | 0.109375 | 0.890625 | 82 | 0.768293 | 0.231707 | TATACATAAATATGAACATTTTGTGCAAATACAAGTGAATCAAGGAAAAGCTGTTGTTTGCAGATATGTTTTGTTTATTGAGTCCAGCAGTAGAAAATGAT[T/G]AACTTATTCAGCATACACAAAAATACCTGCTTCAGGTATCATTCATTCATGCTGTTTTGATGAATAAGATGATGTATTAAGTGTTGTGCTAAAATTACTAC |
| comp99082_c1_seq10:1418-1419 | comp99082 | 1418 | C | G | 770 | 0.780519 | 0.219481 | 74 | 0.121622 | 0.878378 | CATTAAAAGCCGGAATTCCTTCACTGATTATCATATATATACTGTACTACATGCTTGAAGAAAATTAATAAAATTTGTCTTGTGTTAAAACAGGACGAACA[C/G]AAAGCAGAGGAATATCTCTACAAAAGAAACACTAAAATTAACAAATATTCTCCATTTTTGCACAGCCAATTCGCATCAAGAAATGGTTGCAAGTGTGTTTA |
| comp97531_c1_seq1:128-129 | comp97531 | 128 | C | T | 52 | 0.884615 | 0.115385 | 31 | 0.225806 | 0.774194 | TGGCCCAAGGACGTAAAAGACCTCGAGCTTCATTTCCTTTTCCCTCCTCTTTGTCTGCTAGGACTGATTTTTTGCCTTCGTCTCCAGGGCGTAGATTGTCA[C/T]TTTTTCCCTGGATTAGTAGCCTTTTAGTAAATTAGTCGGGCCTTCCTTTGTTTTTAGGGGTGCTTCTTTCCTGTCCTGTGTTTTATTTCGACGGCGTTGGC |
| comp97890_c0_seq1:629-630 | comp97890 | 629 | T | A | 64 | 0.8125 | 0.1875 | 13 | 0.153846 | 0.846154 | ACAAAGGACAAAATGCACCCAAAAAAATGTAAGGACACCCACAATAACACGTAATACCACATGTAACTAATATAGCATGTACAATAATTGTTATAAATACT[T/A]TTCCGAGGAGAAGCTTTAAGCTTTGACACCACTGTAATTCTATCTGTACACATTTGCCCTGTTTTGGGATTTAAAGGTATAAACCTAACATGAATTTCCTC |
| comp96397_c0_seq6:123-124 | comp96397 | 123 | T | G | 53 | 0.867925 | 0.132075 | 43 | 0.209302 | 0.790698 | TATTCTTTCTAATACCATGGATAAGGAGAAAGCAGAAGATAAGATGTATAGCCTCCAACACCATTAAAGCTACTGCTTCTACTATTAGAACCGTCATGTGT[T/G]TATTGATGTACCCGCGGGTCAAAACAAGAAACCAAGCGTAAATGAAGAGAGCCAAGATGACAACACTGTAAGTGGCTGAAATGGAGGCTATGATGTACCCG |
| comp102610_c0_seq3:759-760 | comp102610 | 759 | T | C | 70 | 0.885714 | 0.114286 | 44 | 0.227273 | 0.772727 | TCGTCTTCTCCTTTGCCATCTTTCTTCTTACCGCTCTTTTTAGTGCCAGATGATTTGCTCTTTTCAGATTTGACTTTGGATGATTTTGGAGTGGTTGGCTT[T/C]TCTGGTGAAGATTCTGGAATCTTTATCTTTAGCTTAAGTCCTTGCGAAGGCGAGTCATCCACATCGTCAACTGAGATGTCCATGTCCTCCACATCTTCTAC |
| comp106055_c2_seq6:618-619 | comp106055 | 618 | C | G | 296 | 0.875 | 0.125 | 240 | 0.216667 | 0.783333 | GTCACAGCTAAAGTCAGGTTACTGAAAGGTTCATATTTTCTGAGGAACACCCTGTTCTTCCTCTCACATATGAACGTTGAACAGTGAATGCCGTTGAACAA[C/G]TCGACTGGAAACGAAGGACGAAGATTGGGAATCGTTATATCCTTGATTTTCAAATCTTCGGTGTCCTCCTCGAGCTCCTTGCTAAGCCAAGTCTGCATCAT |
| comp107724_c0_seq5:853-854 | comp107724 | 853 | A | T | 64 | 0.84375 | 0.15625 | 151 | 0.18543 | 0.81457 | ATCATATTCTTATTTTATGAATTGAGTTGTTGTTGTTTTCTGGAATTTGATTTTTTTATCTGGAATTTTCGGACATGGTTATAATTCTAGCAACAACAATT[A/T]AAAAAAAAATCAAATTATTTTTTGTTTCTTCCAAAGTTTTTATATCACAAGGTCCGGGTGCTTGCCCCTTTTAAACGCATGACTGAATTTAAAATAAAAAT |
| comp100610_c1_seq2:108-109 | comp100610 | 108 | C | G | 346 | 0.843931 | 0.156069 | 167 | 0.185629 | 0.814371 | TTGTTTTTATATGCTAATGCATACATAAATATATATATTTGTTTGGAATCTCCTCCACATATACACAATCAGAACTACACACTAAGGGGCACGGCACACGA[C/G]GTCATGAGCGGACCTCGCCACCTACGGGTTAACACAGTTGAACTGCGGCAGCTGCACGAAGCCGGCGACGCAGAACAGCGAGATCCGTAGCGTGGTCGAGG |
| comp103518_c1_seq5:401-402 | comp103518 | 401 | T | G | 53 | 0.773585 | 0.226415 | 26 | 0.115385 | 0.884615 | GAAGAAGAAGAAGAAGAAAAACAAAGGTAGTAATTTCTGAGAAGTTTCAGATATTGGGGGACAGATGAGCCGAGGGGGTAACTTGAAAATGGTGCCGAAAT[T/G]GAACCTTTTTCACACTCAAATATATCCTAATGGAATCTTTTCCACTCTCAAAAATATACATCTACCTACGGATTACATCTCTCATAAGAAATGAGAGAGTA |
| comp101455_c0_seq1:384-385 | comp101455 | 384 | C | T | 35 | 0.714286 | 0.285714 | 53 | 0.0566038 | 0.943396 | GTATGTGTTGCGTAATGAGCAGCAGAGTTCAGAAGTTCCATTACGCCAACACATAGACGGGAAAGGGGAAAATCGAGTCTATATTACGAGTCTGGCAGTTG[C/T]CGCGGATGGGAAAGTTTGCTCTGTACGCGATCGCCCCGTGTTATCTATCACTTCACCGCTTATCCTACATAACACACGTTGCTATTCTGTGCTAACAATGG |
| comp99293_c0_seq2:274-275 | comp99293 | 274 | G | A | 91 | 0.824176 | 0.175824 | 48 | 0.166667 | 0.833333 | CACATGCCACGGGTGAGGTTCTTCCCATCAGCTGTTTGCAGTTCGTTGATGTCGAGCTCCGGTTTATCATGCATCAGCTGGGGATCGATAGTGATGCCGTC[G/A]TGGATCCAGGAGCCGATGGTGAGGGTGCAGTTGTGAGTGTCATGAGGCCAGAAGGTCAGGTCCATGACACAGGTGAAGTGGAGTCTCACGGGCGGGACGAA |
| comp105771_c2_seq1:134-135 | comp105771 | 134 | A | G | 12 | 0.0833333 | 0.916667 | 54 | 0.740741 | 0.259259 | AGACCGAAAACGAGAAGCGAGAAGCGAGAAGCGTCAGCGAACGTGTCTCTAACCCACCAGCCCCCCCCCTCACACTCGCGTGAAGAACACATTGCTCTCCA[A/G]AGTGATGTCAGACAGCGTGACAGAGGCGGGCCCCGTGTACTCTGCGCCCGTGTCCACCCTCCGCCACTGGCCAGGGGGAACCACGACCTGCAGCTCCGTCG |
| comp105212_c0_seq2:173-174 | comp105212 | 173 | T | C | 185 | 0.881081 | 0.118919 | 152 | 0.223684 | 0.776316 | CAAGTCAAATACTTGAGCAAATATGCAAGGCAAGTGAAATTTGACTGCAGTATAACTTATTTAACAGATATCCATACACTTTTTGCAGACTTTTGCGGATT[T/C]ATGTTCTACGACAATACTTTTGTAGTAAGGAATATAACACACGTGCAAATGGCTACATACTACTACACTGTTAGAAAGACATTTACCAATTATTTGTTATT |
| comp88209_c0_seq1:369-370 | comp88209 | 369 | T | G | 35 | 0.857143 | 0.142857 | 70 | 0.2 | 0.8 | AGCGTCGCCAAGCAGTGCAGTGAAGCCGAAGAGAAGGAAAGCGGGAGGGACGACAAGCCCCCCGAACTCGGCGAAACTTCAGCCTAAAGGAGGGAATACTG[T/G]GAATGGTGGAAAGCGGGTTCCTTTAGGGGGAAATCGTGACAATGCAAAGGAGCGGAAACAAAATGCAACACCAAAGAAACTGAAGGAAAGTGGAATCAAAG |
| comp96888_c0_seq1:1578-1579 | comp96888 | 1578 | A | G | 56 | 0.857143 | 0.142857 | 15 | 0.2 | 0.8 | GGCAGGAGGTTAGCAACACACTGGCACATTGCGAACTAGAGTTGTGAAGTCGCGTGGTTACTTAGCTTAATTCAATCACTTGGTAATCCGCGGCTTTGTTT[A/G]TATATAAAAAGACAACTTAGCTCGGACCGTTTGCCATGAATGCTTGTCTGTTTGAAACTTGCTTGTGTGGGTCTTCATTGACATTGGGTGTTGAATACTGT |
| comp100455_c0_seq2:595-596 | comp100455 | 595 | C | T | 8 | 0.125 | 0.875 | 64 | 0.78125 | 0.21875 | TCAGTCGTCTTTTCAAAACGTTAGATTTCGTCCTTATTGAAGACGGAAGATTCTAGAACACATTTCCATTCGCAGCGATGACAGTTGCAGCGAATGTTCCT[C/T]TAACGACGGGTTTCTCTTCGCTTTTTTTCCCTCTCGCAACATTCGTTTTTTTCTGTGGAATTCCGCGTTCGTTCGAGCAGGTCGTCAACGGTACAGTCCGG |
| comp101072_c0_seq1:303-304 | comp101072 | 303 | G | A | 286 | 0.797203 | 0.202797 | 156 | 0.141026 | 0.858974 | ACAGATACTGAGAAATACCCACACATGCTGTCCGTGAAGAATTGCTTCATCAGAGGATCAGTCGTGCGATATGTGCAACTTCCTGTAGATGAAGTAGACAC[G/A]CAGCTGCTCCAAGATGCGGCAAGGAGAGAAGCGCAGCAACAACGGCAGTAGATTGTATGGTTTTTATTTTTAGTTTGGAAAGAAGGTGGTCTCGTCACCTT |
| comp96261_c0_seq1:201-202 | comp96261 | 201 | A | G | 98 | 0.908163 | 0.0918367 | 103 | 0.252427 | 0.747573 | AAAATCTTTACTTTTCTAACATTTATTGAAAGAAGGAAACAAACAATAGAACGTTAGGCGCTGATCAGCAACTCAGATTTTTTATCATAACATTTATAATG[A/G]AAATAAATTGATTAATAAATCTGATGCAAAGATTAAAACAAAATTTAATTTGTCTTACTGCAACAACGATTGCAATGTCCAGCAAGAGCGAGAAGGATGAA |
| comp107581_c0_seq1:578-579 | comp107581 | 578 | T | C | 93 | 0.860215 | 0.139785 | 44 | 0.204545 | 0.795455 | TGCTATACGATTTGTGATGTGCAGCATTTAATTATCAACTAACCCCCTCTTTACTCTTTTCACTATTACACCTTTCTTTAGTGCTATAAAACCTTTGATGT[T/C]TTGCACATTTATTTTGGTTTGTAACTATTAACCTTATTTACCATGGAAGTTTTTTGCCATCATACTGTACAAACTCTCCATTATGTTCCTCATTCAAGCTG |
| comp107770_c0_seq3:588-589 | comp107770 | 588 | A | T | 161 | 0.913043 | 0.0869565 | 97 | 0.257732 | 0.742268 | TTTTATTCAACTGTGTTCATAATTTGTTGTTTTATGACAAATAATGGCTATATTGGAGCTAAGTTCAGAGGATATTTGATATTTGGAGCCGATATTCTACG[A/T]TAGCAATTTTGTTAATGTTTAGTCTAGGATCAAGAGTAAATGCGAAATCTAAGAACTGAATTCAACTTTTTGTCTTTTGAAAGCTGGCCAAGTTTTAAGGT |
| comp107233_c0_seq1:2499-2500 | comp107233 | 2499 | G | A | 75 | 0.893333 | 0.106667 | 84 | 0.238095 | 0.761905 | CTGTTGGCATGGGTCAGAAGAGAAGAGTCCTTAGCAATGTGAACTACCTCTTTGTCAGGGGCATCGTAACAGAAGAGCGCAACAGGAGCGATTCTCATGGC[G/A]GCTCCATTCCCATACGAGCCTCGGCCACCAAACTGAATCCCAGCGGGCGCATAGACATCCTGGAACTTTGTGGCGCGGAGAGCAGCGAAGACGTCAGTCAC |
| comp102076_c0_seq1:1087-1088 | comp102076 | 1087 | T | G | 519 | 0.807322 | 0.192678 | 210 | 0.152381 | 0.847619 | CTTCAGTAAAGAATGCTACACTTATTTATTATAAAACAGAATATAGATTTATGCATTTTGTACTCTTGTTTCAGAGATTTAGTTATTTTCCTGAATATGTC[T/G]TGTCACAGACGACTGTATGGTAGATACATGCTTAGTGGATGCATGACACTAAGCAAGTAAAAAGTGGAATACCAGAAAAGCTTGGAAGCTAATTTACATAT |
| comp107627_c0_seq1:1090-1091 | comp107627 | 1090 | G | A | 24 | 0.0416667 | 0.958333 | 56 | 0.696429 | 0.303571 | ACGCGGTGCATGTCAGCTTCTACTAATTCGATGCCTTCAGCAGAGAGAATCTTTAGAACTTCCTCCATGCGACCAGAAACGTCGTGGGAGAGATTCTGGTA[G/A]AAGTGAGTCCTTGGCACCCCCAGCCGGATGTCGCTCGGGTTTATCGTTGGAAGCTCGCTGAAGGCGGATCCAGCCAGGACAGCGTCAAGCGCCGCCACTGT |
| comp98640_c0_seq1:758-759 | comp98640 | 758 | A | G | 6474 | 0.93528 | 0.0647204 | 7439 | 0.280683 | 0.719317 | ACCAGGTCACCGTTGAGCAGCTGACATATACCCCGCATGTCAGCCCAGGTTCCTTTCGTCACGGAGTCAACCATGAGGCAGCGGCCACCGATCTCGATGTA[A/G]GGAGAATTGCAAGCAACGGCTTTTACCGGACGCACGCTATGACTCCGGAGCCAAGAGTGCTCCGCGAACACACAGGCTGCAAGCAGTAAGATGCTAATGGC |
| comp107184_c0_seq6:1007-1008 | comp107184 | 1007 | A | T | 91 | 0.912088 | 0.0879121 | 97 | 0.257732 | 0.742268 | CATTAGAATACTAATTCTCCTTGCAGCTAAACACTTATGGAGCCATCTGTTTGGAAGAAAATTCACAGTGGACAGTACACATACACATGCACAAATGGTTA[A/T]AGCTACCATTTGGGTGTCCATGGGGTAAAGCAAGAAAGTTTTGCAACTATGTGAACACAGGCCAGCAACGGTTTAAAGTTAACCATTAACTTATCTCACTC |
| comp106572_c0_seq1:2518-2519 | comp106572 | 2518 | A | G | 74 | 0.945946 | 0.0540541 | 96 | 0.291667 | 0.708333 | TCACAACTGTTTCTTTTCTCTTTTTTGAGAAGTTCTATGTCTAAATGTTTTTAACACTATGGAGGGGTGAAGAGGAAATATTTAATACAGATAATTATCAA[A/G]GAAACTGTTTACCTTCGACCGTGGAGAAGTAAAGATGAAATGCTCAACCTTTAAAGAAGGAAATAATATGACTGGCGAGTCACACACATTTGATTTGTTGC |
| comp107487_c0_seq1:1620-1621 | comp107487 | 1620 | G | A | 186 | 0.268817 | 0.731183 | 26 | 0.923077 | 0.0769231 | GCCAGCACGGTCACTATCGCCGCCACCATCCTCACCATCCCCGGCCTCACCATCCTCCTCCTCCTTGTTGAACCGCTCGGGGAATGCGTGGGAAGGGCTCG[G/A]AGAGGAGTAGGTACACAGGTGCCTAACAGAAAAGCTCCCACGACAACGACCTTTCAACGATATAACGCACTTGGAATGGGCACTGAATGTCACTCAGTGCA |
| comp106269_c0_seq11:2984-2985 | comp106269 | 2984 | T | C | 208 | 0.692308 | 0.307692 | 157 | 0.0382166 | 0.961783 | TAAATTATTACCTGATTGGTAAATATGTCCACCCCTGCCTCTTCAGCTGCTCTGGCCATAGCCTGTGTAACACCACCCATGCCTCCTTCAGGGTAACCCCA[T/C]GCACCTCGGACTCCAGCCACTTGGGCCATCACATGATGGAGTAAAACATATCCACTGCCGGGGGTGTTGGGACTGATCATGGCCCCTATGACACTGTCTGT |
| comp101226_c0_seq3:864-865 | comp101226 | 864 | T | G | 208 | 0.653846 | 0.346154 | 1 | 0 | 0 | CTCCTCATCACTCATGTCGGTGATATACTCCTTCAGGATCGTGTGGATCTGCGCGGCCTCCTTGCCCTCGTAGATGTCCTGCGCCGAGTCGAGGACGAGCC[T/G]GAGGTACAGCTGGGTCTCGATGTCGCCGTTGAAGCTGCTCAGGGAGTCGTCCAGGTAGGCGACGGCCTCGCGAAGGGCCTTGCTCTTCAGGATGGGCTTCA |
| comp107698_c1_seq1:371-372 | comp107698 | 371 | C | T | 499 | 0.731463 | 0.268537 | 425 | 0.0776471 | 0.922353 | AGCTTGGAATGTTTTTTTGCTGCAAGATTTAAATTTTCTTCATAAAATGATAATCATATGGCATTAAAAAAGAAAAGTGGAAAACATAGCATACAATTTCA[C/T]AATGCCACAATCACAAATTATTTGACCCTTGAGTATACACACAATAAGGCTTAGATTCTTCTGGTTCTACTCCACGAACACTAAATGCACAGAAATAAAAC |
| comp97879_c0_seq1:765-766 | comp97879 | 765 | G | C | 66 | 0.878788 | 0.121212 | 80 | 0.225 | 0.775 | GTGTCCCCGCCCATGTCCCCGCCCCCGCTCATGGACCTCAGCATGAAGCCCGCCCACAGCCCTGCCCCGACGCCCAAGATCCTCGCTCCCACGCCCATCCG[G/C]GAGGTGTACGTGGGCCCGCAGGACCTGTCCATGCGGCGCCCCTCGCCCCGAGACGACCCGAAGGCCCTGGAGGGGAAGAACTCCTGGAGGCCGTGGTGAGC |
| comp97575_c1_seq1:538-539 | comp97575 | 538 | T | C | 411 | 0.812652 | 0.187348 | 669 | 0.15994 | 0.84006 | CTGTGATAGGTACCTAATGTTAGCTTCCACTCTTTGTCGCATTCCCTCTAGCTCTTTCTGTCCGTCGTTTTCTTTTTTTTCTGTTTCCTCTCTTTCACTCA[T/C]AAACTTGTTTTTTTTTCTCATAGTTCATATCTTCCGAGATGTAATAATTCCATATTTGTACAAAATTCTATATTTTTTCATCTATAGACTTAATGCCGTCT |
| comp100168_c0_seq1:1889-1890 | comp100168 | 1889 | C | T | 63 | 0.793651 | 0.206349 | 85 | 0.141176 | 0.858824 | AAGGTGGATTCTCCTACCCACTTTTCTACACTTACATCACTACACCTGACATTCTTGAAGAATTCATGTATCTTGCTACCAAAGAAGGAGGAAGTTTGCAG[C/T]TGGACATCATTCCTGTCAACCATACAAATAAACAACAAAGAACAATATCAACACGTGGCATTGACAGAGATCTCAAGAATGACTTCAAGGTTGGAATGAAG |
| comp105595_c6_seq1:327-328 | comp105595 | 327 | T | G | 78 | 0.923077 | 0.0769231 | 48 | 0.270833 | 0.729167 | ATGATTAAGCATTACAGTTTCATACAGACAGCAAGATATGGAAAAAAACGGAGATTATTACATGTGTGAGTTCCATTACAAGCGGTTGTCAGTGTACAACG[T/G]AAATAAAATGTAGATCGATATTTTGGTGACAATGAGCTGTTACGTGTTGATACAGTGATCGATAGAAAGGATAAAACGAAAAGAAAAGAAAAAAAAAACTA |
| comp105860_c0_seq6:372-373 | comp105860 | 372 | A | C | 269 | 0.884758 | 0.115242 | 43 | 0.232558 | 0.767442 | ATTCTTTCCACATGGACCACACCTCTAATTCCATGCTCTATGCCTCATATCAAAGCCTGGGCATTCAGAAATGTAGGACCTAAGCCACTTGTTAGTGGTAT[A/C]ATCCAAACACTTTAGCTGCATCCTGCAAGTCCTTGTATACAGATGAACATTTTGTTCAGCTTCAGCTTCCGCTTCCAGAACCTTTCCTTCGCGGCCATCTT |
| comp102479_c0_seq1:189-190 | comp102479 | 189 | T | A | 68 | 0.838235 | 0.161765 | 43 | 0.186047 | 0.813953 | ATTTTAGGAAATGATAACCCAAAGAAATGTCCAGGTTTTTTTCCCATGTATGATCTCTCAAAGGTGTTGCCAATATCGCACAGATGGACGTCTTTTTTTTT[T/A]AATCTTTGCACATTCTTTTTGCTGATGAGATTGCTCATGAAAGTTTGATATATGTACTTTGAAAGTACCTTTACTATATACCTTTTCTTTCATTTCTCTTC |
| comp82587_c0_seq1:494-495 | comp82587 | 494 | A | G | 69 | 0.652174 | 0.347826 | 2 | 0 | 0 | TATCAGGTGCCAGAAGTCGTGAGGATGCCCTGCATAAGGAGGCTTCTGTGCCACATGGAAATGGCGGCGAGGACCAGCGAGAACTACCTGCCGAAGGAGCC[A/G]GCAGTCATCGAGGGGAGGAACCTGGAGGAGGAGGTGGCGCCCGAACCTGCAGACCCGATCACTGAGTTGCAGATTGAAGCCGTCAGGGCGCTGTTCAAGGA |
| comp104216_c0_seq7:576-577 | comp104216 | 576 | A | T | 121 | 0.752066 | 0.247934 | 30 | 0.1 | 0.9 | CATCTGTAATGGCGACCTCATGAACACTGAACACCGACCGACTGACGAAAAGGATAAGACGTTCACATTTAAGACTTCTTCATGACGACTATAACGACGGC[A/T]AGTCAACGACGATAATGTGTAAGACTATCCGCTTAACAAACCATCGAAGTGTCAGCGAGTCCACAATAACAAGCTGTTCAAGAGGATAAGGGAACAAGATT |
| comp97955_c0_seq5:283-284 | comp97955 | 283 | C | G | 84 | 0.690476 | 0.309524 | 26 | 0.0384615 | 0.961538 | AGCGTGGGCGGGGCGTGGGCGGGGCGTGGGCGGGGATGTTGCTCCGGTGGGGTCTAGCCTCGCCCGGGCCTGCATTCTCCCTCATGCCTAAGTGTCAGTTT[C/G]GTGTCATGGCACTTGGCATTCTCAAAAACGCATATATTGGGGTAGGTCACGCCGTTGCTGCCACACACCGGCATCAAGTTGAAGGGGCAACCGGTCTGGCA |
| comp93416_c0_seq1:729-730 | comp93416 | 729 | A | T | 227 | 0.876652 | 0.123348 | 209 | 0.22488 | 0.77512 | TTCTTGTGACAGTAGAATTAAACTGTAAATAGGAGATAATTGTAATTGTGCCCATTATGACACGTCTGAGGTTTTTGCTTTTGTTTCATTCCTATTGACTA[A/T]ATAGTGTGGCAACAAAAATTGCCGTGGTCATATCTCCTGTAACATTTCACATTCGTTCTGTACACTGACAATTATTGTTTACATAGGTCAGATTTTATCAA |
| comp104841_c0_seq1:654-655 | comp104841 | 654 | G | A | 210 | 0.766667 | 0.233333 | 139 | 0.115108 | 0.884892 | TCACGGGTTTGCCCTTCGAGCCGCCAGTTGTGCGTCCGCGATTGGATTCAGCTCGGTGTTGGCACTTTGAGGGCGAATCCAAGGCGCTTGAGGGTCGCGTG[G/A]ATGTCTTCATACACCTTCGGGTCCTCGATCGTCGGAGAGATCTTGATGGCCTTGTCGCGTGCGAGGTCAGCGATGCTCTTCCTTGCCGTCTTGCCCGAGCG |
| comp101984_c0_seq3:833-834 | comp101984 | 833 | G | A | 93 | 0.72043 | 0.27957 | 58 | 0.0689655 | 0.931035 | TTTGAAATTGTGGCTTTGTCCGAGGAACTGAAACGGTTAAGAGCTGGTCCAATAATTGGGGAAATGGGCAAGAACATGCGGGAGAAAGCAGCCGGCAACAC[G/A]CCACAGCAGAAGATGTTTATGTATTCAGCCCATGACACAACATTGGCACAGCTACTCCTCGGCTTGGGTGTATTCAACAATGTGGCTCCACCCTATGCCAC |
| comp97679_c0_seq1:315-316 | comp97679 | 315 | A | C | 198 | 0.732323 | 0.267677 | 37 | 0.0810811 | 0.918919 | CTCCGCTCCCCAAGGTGGACGATCCACAAACTTCCTCGTGGAATACCGCGACAGGAAACCGAGCGTTCCCGAAATCGAAGTGATCCCGGTGGACGCATCCG[A/C]ACCCGAGAATCCGTCGGCAGGACTTTACGAAATTAATGAGATCGCCCCGAAAGAGACCCCCACGACCAGCTGGTTCAGGAGGTGGCTGCGTCGCATTTTGG |
| comp92867_c1_seq1:859-860 | comp92867 | 859 | T | C | 86 | 0.651163 | 0.348837 | 1 | 0 | 0 | TGCGGCATCACCAGCTTCAGGCAGGTCCCGGCCAGGGAGTGGTACTCCGGGCGGCACCTTCCCGCAAGCACCGCAGGCAAGAGAGACGCCACGACGGCCAA[T/C]GCCCGCAAGATATTCACGCCCATCATCGTCTCTGTCACGCCCACGGTGTGAGATCCTTGCGCGGAGGAGGTTGCGTTCGAGGCGAAACGGCGCGCCCGAGT |
| comp95925_c0_seq1:1022-1023 | comp95925 | 1022 | C | T | 192 | 0.651042 | 0.348958 | 1 | 0 | 0 | GATTTTCAAGTACTAGTGGCCTTGGAAGTAGTAGCGGTCTTGGGGGCATTGGCGGTCTTGGAGGAGGTACAGGTTTTACAGGGACAGCTGATGGAGGAATC[C/T]CTGACAGTTATGCCAGCTTCGCAAGTAGTAGTAATACTATTGAAGTTGAATTGACGAATCATATAAGCACTGGAAATACCGAAGATGATAGTTCTCTTGCA |
| comp106018_c0_seq4:597-598 | comp106018 | 597 | A | G | 54 | 0.777778 | 0.222222 | 55 | 0.127273 | 0.872727 | AGAAAATTAAGAGTTTCATTCATAGTTCTTGATACGATTTTTTAATGTTGCATTTGTGTAAACCACTCTGTACTGATCTCATGGTAAGAGTATTTGGTCCC[A/G]ATTTAATGTCTTCTTAGGGAATAAGAAGGTTCATACTAAAATTTGTTGCCTTGTTATATTATTGTTCTAATATATTCAGATACTAAACATGTTCTTTCAAT |
| comp107229_c0_seq1:4073-4074 | comp107229 | 4073 | T | C | 66 | 0.30303 | 0.69697 | 43 | 0.953488 | 0.0465116 | AGATGTCAGTGCATTCTCAGGGGTTGATCAGGAGCGATGGAAGAAAGTTGCCGAGTGTTCAACGTGGCTTGAGGTTCATGAGGTTTTGCTTCGAGCAGGTT[T/C]AGATCTCGTGTCCCAGAGGTGGTGGGGGAGGCTTTACTGAAGGGGAAGGCAGACCTGAGGATGGGCCTATACTTTTTTAAAGGTTTAGTAAAGAAGTGAAT |
| comp102124_c0_seq3:127-128 | comp102124 | 127 | T | C | 417 | 0.119904 | 0.880096 | 209 | 0.770335 | 0.229665 | ATTATTATTATGCCTGAAATCAAGCAGTTTAATAAAGAGCACCAAAATTTGCATGAAATCCTTTATTTTTTTTACAAATGATTCTACAGGAGTTGTTTCCG[T/C]CAAGGCCTGCGTTTCCCTCCACTCCGGCTCCACCTGCAGTGAAACCCCACGGCATCACCGACGATCCTGGGGCTTCCTTGCACGTAAATGCCTGCGGAAAA |
| comp105035_c0_seq2:632-633 | comp105035 | 632 | C | T | 451 | 0.922395 | 0.0776053 | 66 | 0.272727 | 0.727273 | CCCCCTACCACCACACAAGATCCTTGTGTCAGTAATCCAGGTTCTCGACAATGTTGTGCAAGAAACCCCAACCACCCACGATGCGTAACAAAGGCTCCATG[C/T]TCTGGCCCCCGTGACCCTCGCCCAGAGTGCCGGCCCACCACACAGGTTCCCAGCACGACGCCCAATCTGGAAAACCATGCCTTCCACGCTTGGCAGAACCG |
| comp95004_c0_seq9:1051-1052 | comp95004 | 1051 | A | G | 116 | 0.853448 | 0.146552 | 98 | 0.204082 | 0.795918 | AAGGAAGACTGGCTACAATGGCAGAAAGATCCGACCACTCCCGCCAGTGAAGGAGTACAAGGCCAAAATCCCAGACACACCTGTCATGAAATTTACACTAA[A/G]TGGAGAGGGACCAACCAGACCTGTTATTCCAAGCACTGATAGGGTTACATGAATATTTTTGCTGTTTCAGTTTAGAAATGTATATATGTATAGTGTGAAAG |
| comp106943_c0_seq2:919-920 | comp106943 | 919 | G | A | 130 | 0.8 | 0.2 | 73 | 0.150685 | 0.849315 | TGTATAGAAATTTTTGCAGATCATTTTCTGACAATAAGATCACCTTGCTCTTGCTGAACTTTATTTCAGTGTATCACATTTCATTAAAAGTGGTTACACTT[G/A]AGCAGGCAACTTGAATAATTCATAGTATTATGTCAGTACTATATTTATCAGCCAATTATATAGGTTGATTTTGAAAACAAAAGTTAATGATGTAGAAGATT |
| comp101836_c0_seq1:708-709 | comp101836 | 708 | A | T | 140 | 0.871429 | 0.128571 | 189 | 0.222222 | 0.777778 | CGATGCACCCTCTTCCAATATCTAAATTACCGTTAATCGTCGAATGTTCCATTTACCCCTAGGACATATGCTCATGTTTGACCTAGCAATTTTCAGTTACA[A/T]TATTTTTTTTTCAGTATTTTCAGTATCAGTAACATAAAAAAATAAAAAATGATTCAGTCTGTTATCAAAATTTACAGATAATAAATGACGTTTTCAAATCT |
| comp106324_c0_seq1:934-935 | comp106324 | 934 | A | C | 16 | 0.125 | 0.875 | 62 | 0.774194 | 0.225806 | AACATATAACAATTGAACCCATAAATTACAGCAACAATACACATGAAATACATCTCTGATTTTTGGCATCCTCTTGTACACTGTGATTTATTTAATTGTTC[A/C]GAAAGTACAGTCACGATAAAGCAATTCCCTGCCAGGAACAACCCTAAAAAATTTCATCACAACAATTGCTTACATAATGCCGAAGTCACTGAAAGTATAAA |
| comp105732_c0_seq5:1300-1301 | comp105732 | 1300 | C | T | 187 | 0.106952 | 0.893048 | 246 | 0.756098 | 0.243902 | TCCTGCCAGGGGTTGGCGCCGTCGTCAAAGTGCATGGGGCATCGGTCGTGTTCTCTCTCGTACTCCACCATCTTGATCGCCACGGCCGAGCCCCCTCCCGT[C/T]CCGTGCACCGTCTGATGCAGGTTGCAGAAGGGGCCGACGCTCTGGGACATCAGGATCTTGTTCAGGTCAGCTCTCTGGTAAACCCAGCACCTGTACTTGGA |
| comp106144_c0_seq1:156-157 | comp106144 | 156 | G | A | 105 | 0.742857 | 0.257143 | 64 | 0.09375 | 0.90625 | AATGTGTATGCCATTTCCAAAATGGCTGTAGAATTAGCTAATAAAAAGATTGACAGTGGTATAGATCTTCTCCAGAGAATTTCCAAGTTAAATGACAGGTC[G/A]ATCAACGGAGCATCAAAATTGAGGAGACGTGTACAGGCAGAAATAAAATGTCTGACAAAGAAACTTGAAGATAAGAACGAACTAAAGGAAGAACATATACA |
| comp107854_c0_seq6:249-250 | comp107854 | 249 | A | G | 21 | 0.285714 | 0.714286 | 199 | 0.934673 | 0.0653266 | GAAAAGTTTAGTGTGGAGAGTTCTATTATGTGTATGGTATATTTATGTGTTTGTGATGATTTCAACGAGTTGGGCTCCAAATACTGTAAGGTAGGATTTGT[A/G]GCTTGTTTAGGAATAAATTGTGAAAGTGAGGTAGAGTAACCAAAGTTCAAAGATAGCTGAATGATAAATAGTAAGATATTGAAACATGAGAAAAATGGAAA |
| comp106968_c0_seq9:222-223 | comp106968 | 222 | T | G | 33 | 0.818182 | 0.181818 | 65 | 0.169231 | 0.830769 | GACGACGCGCCGCTCGACCTCACGCGGAAAGTGTTCGGATTTTACCTGGGAGACGCCGAGGTGGGAGTTGGCAACGCTGATCAGGTGGTTAAGATGTTCAC[T/G]GATCGGACCTTCGCCGTCTGCCACGACCTCACCACCCTCTACCACGCCCACCACGCCCGCCACGCCCGCCACCGGTCCTTCGCCTACGAGTTCGCCCACCG |
| comp107372_c0_seq13:1595-1596 | comp107372 | 1595 | A | G | 109 | 0.715596 | 0.284404 | 15 | 0.0666667 | 0.933333 | GACACTTCTTTGGGGTCCATGAGCCAAATGGGCTGAATGTTGTTGGCTTGTTCGCCGTTCACTTCGATAGGAGAGCCCACGAAGCTGCTGTATTTCTTGAT[A/G]ATGTCTTTAATTGTCTGGTCATCAGCAAACTCTCTATCTGCCACTTTCAGATGACACACGATCTTTGTTCCTGGCTCAACTTCGCTGCACTCTTCAATAGT |
| comp99343_c0_seq4:168-169 | comp99343 | 168 | C | A | 187 | 0.684492 | 0.315508 | 84 | 0.0357143 | 0.964286 | TCACACTTAACGACCAGGACGAAATGAAAAGTGACAAGTGGAATAAGAAGGAACGAAACAAGGTCAAAAGTGGGGATTGAAGGAAAATTAGAGAGAGAATC[C/A]GAGCCGCAGGACGTCAGAAGGCAAGGAACCGTGGCGCCTGCGACGCCAGCACACCCATCCCGAAGGTCCTCTCACTCGCCCCCTAGTAGTGGGTGCGAGCG |
| comp107385_c2_seq10:2374-2375 | comp107385 | 2374 | A | T | 586 | 0.856655 | 0.143345 | 125 | 0.208 | 0.792 | CCGAAGCAACTCGCAGCCGAAACATGGCGCAAAACAGAGGAAGGTTGGGAGATCCGTTGTAGGATTAGGTTAAAGTGACGATCCTCCCGGTATCCGGAGAA[A/T]AACTTTCGCCTAACAACGTTGTGAAATCATCGTACAGTAATTCATGCAGGGTACGATGAAGATAATGATCGTGTTTTGTTTTATTTTATCACCATTACAGT |
| comp107542_c0_seq11:1855-1856 | comp107542 | 1855 | G | T | 112 | 0.276786 | 0.723214 | 362 | 0.925414 | 0.0745856 | AAACCTTCCCAGGCTGATTCGTGCTTTTTGCTCATCAACGAATTAATGGCACCGGGTGATCCCTGTGGCACCAAGCTTCGCGGCCAGAACTCAGGCAGTTT[G/T]GTAGGAGAAGCGATTTCCTAAGGTTACAAAGCTTCCCGAAATCAAGGCACGGAATAGCCGCTCCTGTCCGTGGGAAATTTGTTTGGTTTATGCCCTTGCTT |
| comp103009_c0_seq5:1240-1241 | comp103009 | 1240 | T | A | 153 | 0.784314 | 0.215686 | 81 | 0.135802 | 0.864198 | TATATCGAACTACAGGGATTTTATTACAAAAAATAGAATAAAAAGAGGATAATGCAGTTGACAAAAAGTCTTACAGTATATCATTACCCCTGACAAGTTTT[T/A]AAATACAGTTTCTCATGTCATTTAGTTGATTAACTTGAAGAGGATGATGGATAAGATGAGGCCAATCAGCATTATAAAGGCGACGGCTATGCCCACGCCCC |
| comp104554_c1_seq1:588-589 | comp104554 | 588 | A | T | 55 | 0.727273 | 0.272727 | 38 | 0.0789474 | 0.921053 | TTTATAAAATTAAAATCAACATCGTCATTATTACAGACATCATGATTATCATGATGTTATTAAGAATATCAATATCAATAAAAACAATAATCTTCACAAAA[A/T]TTTAGAAAAAAGATAGACAGGTGATATAAGTATGACTAGTAACTGACTCCTTGATGATTAAGCACTTGGAACCATTTACACATAAAGACAATTAATAAAAC |
| comp106676_c0_seq10:2727-2728 | comp106676 | 2727 | C | G | 54 | 0.703704 | 0.296296 | 36 | 0.0555556 | 0.944444 | TCGCGTGGCACTCGACCAGCACGCGGGTGGCGCTGCCCAGCCGGTCCACGTCGATGCGGGGCGGCTCCGGCGTGGTGGAGGGCGCGGCGTGGAAGCGAGGC[C/G]GCGGCGGCGGCGACGGCGGCGGAGGCGTGGAGGAGGTGGTGGTGGCGGGCGGGGCGGTGGCGGCGCGCTGGAAGTAGGGCGGCAGGGTGGCCGAGAGGGTG |
| comp103811_c0_seq11:182-183 | comp103811 | 182 | A | G | 31 | 0.935484 | 0.0645161 | 80 | 0.2875 | 0.7125 | TGACTTTTTATCTGTAACACCAGATTGCTATAGGTAATAAGCATCGTCATCACTATACTTGAATTGCTTTCACTTCAGCAACCAGTATTATAATAACGACA[A/G]TGGCAATAGTAATGCTGATAATAATGATAGTGATATATTGATAATAAAAAATGCCATTAACATCATGAAGCAATGATTCTACTCCTTTTTATCTGCAGTAT |
| comp105631_c2_seq10:904-905 | comp105631 | 904 | C | A | 180 | 0.866667 | 0.133333 | 96 | 0.21875 | 0.78125 | ATACTGGATTGTATATATTCATGCTGATTTGTTTCATATTGTAGACGTTCGGAACCAAATAGTTGTATGTAATTCAATTTGATAGGAAGAATAGGTAGGTT[C/A]TTTTTCTCTTTATTTCTTGCTGCAATTTAAGTAAGCTTTTTTTATATGGATTTTTTTTTACATTGCATTAGGTCTCTCTAATGAGAAATATTTCAAGTGCG |
| comp97291_c1_seq3:153-154 | comp97291 | 153 | C | T | 7 | 0.285714 | 0.714286 | 60 | 0.933333 | 0.0666667 | TACTTAAAATAAATTCCCTGAGGAAGGTGATGACGTGATGACCCAACCGTAGAAGGGACACATCATCCAGCTCATACGCAGACTGCAATTGGGTATCCACC[C/T]TGGGGTATACCAGCGAAAATCTCACGTATAGCGATAAGAATGCGCGGCGCGGCGCGTAGATCGCCTCTTGCGATGTGTGCATCTTTGCACGTACAGAACAA |
| comp106695_c0_seq1:597-598 | comp106695 | 597 | T | A | 296 | 0.777027 | 0.222973 | 216 | 0.12963 | 0.87037 | AACCTGACCTTCATCCAGACGGACAAGTACCTGTACCAGCCAAGTCAGGAAGTCAAGTTCAGGGTCCTCACTGTGTACGGGTCCAAGGCACAGGTCGTTAC[T/A]GAGCAGTACCCGGAAATCTGGGTCACAACACCCTCCAGAACCCGCATTGCCCAGTGGAAGAACGTCACCACGTCATCCGGCCTCGTCCACCTGTCCTTCCA |
| comp106585_c0_seq2:101-102 | comp106585 | 101 | C | T | 647 | 0.00463679 | 0.995363 | 273 | 0.652015 | 0.347985 | GTCCACACTCAGCCTGGCAGCGACCAGACCACCCTGGACTGGGCAGGAGCAAGGACTCTCTGCCAAATGCTTTCGGATTCGACCTGGACTGTCGACTTGGC[C/T]GTGTTCGATTCCTCTGAGCAGTTGGCAGCGGTTTCGGAGGCGTGGGTCACGATCGGTGCCACGTACCCCTACCCCTACCCGTTCATGTGGATCGGCGTGGA |
| comp89982_c0_seq2:871-872 | comp89982 | 871 | G | A | 10 | 0.3 | 0.7 | 57 | 0.947368 | 0.0526316 | CCAGCAGGCGGAGGAGCAGGCGCCGCCAACGGGGCGCACGAGGGCCAGGCACAGGACGCCAAGCAGGAGACGAAGCCGGACGAGAAGGACAAGCCGCAGGA[G/A]AAGAAGCCCGAGGCAGGGAAGCCCGGCGAGGAGAAGGAAGCCAAGCCAGAGTCCAAGCCAGGCGAGAAGCCGGAGTCAGCGAAGAAGGAAGAGACCAAGGA |
| comp106569_c0_seq1:1832-1833 | comp106569 | 1832 | T | C | 315 | 0.292064 | 0.707937 | 165 | 0.939394 | 0.0606061 | TTAGTGCTTCAAAACAGCCTATCGTCATAGTTACCACAGGTGCTCAGTAACTTGTCCGTGAATTAAGTGAAATGGTACAAGTTTAACTCTAAGTATTTCAT[T/C]TGTTGATCTTTTTGGATATTCTAGAGGCCCATATAGGACGGACAAAATCCAGTACATGAAGCTTTCTATAACAGCCGTTGCTACTGAGTTTACCTGCAATG |
| comp106886_c0_seq10:3284-3285 | comp106886 | 3284 | A | C | 89 | 0.707865 | 0.292135 | 66 | 0.0606061 | 0.939394 | GAGCTGACGGCAACGCAGCAGATGTTCTGATTGTCGTGGGCGACGGAGGAATGGGAAAGACTACTTACCTGAAGTACCTGATGGAAATGTGGATCAAGGAC[A/C]CGTCTTCCGTGCCTGGCCTTGACCAGGTAGAACTTTTATTTTACATCGAGTGTCGTAATCCCAGCATAGCCTCGCTTGATCAGTTGATTCGGCACCTTCTG |
| comp92386_c0_seq4:393-394 | comp92386 | 393 | T | C | 51 | 0.647059 | 0.352941 | 2 | 0 | 0 | TACTTGACGGCGGTGGTGGCGCTAGCGGCATGCGCGGCTGTCGCTCATGCCGGCACTAGGGACAACTGGTCGTGGGGCGCCGAAGACCATGGAGAGGCCCA[T/C]GCCTCTGCGCCTTCATCTATCTCGTCGGCCGTGGCCTCAGCCCCGTCGTCGGGGTCGCCCAGGTCCGTCTCCTTCACCGCCAACAACGACCACCAGTTCGT |
| comp104930_c3_seq1:187-188 | comp104930 | 187 | C | T | 133 | 0.932331 | 0.0676692 | 7 | 0.285714 | 0.714286 | CGCCGAGGCTATAGAAAGTGAATCGTTTATGATGTTTAACTCAACCTTGTATCTGACTTCGAAGTGCTGCTTGTTCCAATGTTAATAATGATGATGGTGTG[C/T]GTGCTGCTTATAATCATGATAACGGCAATGAAATTTAATTAGCATTTCCCTTTTCTTTTTTATATAATTTTCATTACCATCATCATTATAATTACTGGTAT |
| comp96445_c0_seq1:289-290 | comp96445 | 289 | A | T | 163 | 0.233129 | 0.766871 | 108 | 0.87963 | 0.12037 | ATTAATTGAGAAGATGGACAAATGGATCAATGAAAAGTGCGATTTTGGAATTAGTTTTAAGTGATGATGTACTTATAAAGTGCGCTGAATTAAAAGAAAAA[A/T]ATGTGAGCACATGAGACGAGTAATAAAACAGGTAAATTAGTATTCAACCTCTGTCATGTTTTTTTCCCAGTTTTTCTTTTCAAAAACTAATTGTTGAAAAA |
| comp103090_c0_seq1:159-160 | comp103090 | 159 | G | C | 781 | 0.0576184 | 0.942382 | 1084 | 0.703875 | 0.296125 | CGGACTCAGGGAGGAGACGGGGAAGAAAATACTTAGCGCCATGTCCGAGAAGGTGTTGCCTGTGATTGACCTCCGGCTGGCCAGGGGGTCGACGCGCCCGG[G/C]TCTCGTGCAGCAGCTGAAGAAGGCGCTGACGGAGGTCGGGTTCATCTACCTCGTCGGCGTCGAAGGCTACGACGAAGAGGAGCTGCTGCGCCTCACCAAGT |
| comp98666_c2_seq3:569-570 | comp98666 | 569 | A | G | 990 | 0.190909 | 0.809091 | 614 | 0.837134 | 0.162866 | GTGGCACTGTGACGGCATCACCGACTGCTTCGGCGGCGATGACGAGCTGAACTGCGGCGCCAGAGTTGCCCCCATGGTCTCCTACAGCAAGATCAGGACCC[A/G]CACGCGCGCTCAGACCAAGCTCCTCCGCAAGCTGGCCAAGATCCGTCGCCATTAAGGAGACGTCCTGCTACACCGTCTCCATCCAGCGAAGAACCGCTACA |
| comp104992_c0_seq12:1118-1119 | comp104992 | 1118 | T | A | 73 | 0.917808 | 0.0821918 | 81 | 0.271605 | 0.728395 | ATTTCATCTCACACCTCGGATGAAACTCTACAGGGTTCTCGACATGCAGGCTCCAAAAGAAACACCCTAGTCCACAGCCAGTCGATGGGAGACGAACCAAT[T/A]GGGGAGTACCGGTACACAGTGACCCATGGCTCGGATGCCTTGAGAATCACAGGCAACAACCTGCATCTTGTGAGGACAGCAAAACTGGTGCTTGATGAATT |
| comp105635_c2_seq7:335-336 | comp105635 | 335 | T | A | 99 | 0.858586 | 0.141414 | 113 | 0.212389 | 0.787611 | ATTGATCTGAATTATTATTTTCAATCTAAAGATCACAATACTAATATATAACCATAACTGCCATTCATAAAGTCAATACTAGCTGTTGCTGGTAAAAATAA[T/A]GTACAATTTATGTTAACCACATGCTGACGGGGATGGCATGTGCCATCTCCACTGTGTGTTTAATCTAGTAATTGTTTTTACATACAGATGGATCCACAAGT |
| comp107472_c1_seq10:3690-3691 | comp107472 | 3690 | A | G | 980 | 0.929592 | 0.0704082 | 790 | 0.283544 | 0.716456 | TGTTATTCCATGTTCTGTCCCTTTGGAAAACCAGCATTGAGCCTCGGACCTTATGCAAGCGCTGCCTTATTCTCCAAACTGTTGCCTTATGAACTCGAGAT[A/G]AATACCATTGCTGGGGAATGTTGCTGAACGAAGTCGTTCATAGAAAGACAGATTATACCGTTGCCTTAAACTACAGCCAACAATAAAAATCTTGTAGAAAA |
| comp93045_c0_seq3:197-198 | comp93045 | 197 | T | G | 86 | 0.918605 | 0.0813954 | 11 | 0.272727 | 0.727273 | TTCCCCGTTCTATTGAATCTTTTTTTTTATCATATTTTCTGTTTGGTCTTGTTTTCTTCTTTTCTCTTCTCTCATCCTTGTTTTTTGGGGATGTATTTTTT[T/G]CGGATTTATTTTTTATTTTTGGATATTTGTTTTTATCGTTTATGAAATGGGCTTTATTTTTCTAACTTTTGTCTTTCTTTGATTATTCTTTTAATGTTTAT |
| comp107273_c0_seq14:206-207 | comp107273 | 206 | G | A | 73 | 0.931507 | 0.0684932 | 70 | 0.285714 | 0.714286 | TTCAAAGGCCCAAATCACCCAACAGAAGAGAGGTCTTTGGTCGTCAAATTTGCTCCGACTGCACAGGATGCAATTGAATTCATGAAGAATGGGAACCTCGC[G/A]AGGAAGGAGATTGAGTTTTACAAATTTGTCTTAACGGATGACTTCCGACTTTTCTGCGAGAAGAGCGGCCTGAAGAATCCAGTCCCAGATGTGTACTGGGC |
| comp100589_c2_seq10:2167-2168 | comp100589 | 2167 | A | G | 165 | 0.818182 | 0.181818 | 87 | 0.172414 | 0.827586 | CACACTGCACTCTCACCACTACTAGCAGACAAGGACCCAATGCTTTTGCACCGCCTTGGAGCAGCAGAAGAGCCACAGGATGACCCAACTCCACTGCTACT[A/G]CTGCTGCTGTGAGCTGGAGAAGCAGGGGGAGAAACCTGCTGTTGCTGTGGATGGAAGCTGCTACGGGAGGATAGTGAGGTTGTATTTTTGCGAGGTGGTGG |
| comp104400_c0_seq4:402-403 | comp104400 | 402 | A | C | 104 | 0.798077 | 0.201923 | 72 | 0.152778 | 0.847222 | AATCATGTGACTGCCAATATAATTATCAGAAACAAAAATGGCTCAGAAAATAATGTTCCAGGGTTTTCGCATGTGGATGATAAAAATGAAAAGAAAGCTTA[A/C]TGCCTCTCGAATGGAGTCATCTTTTTGTGATATCTCTATGTACATGTGATGGAGACTTGTTGGCATTTCTTTTTTCTTTGTCGTTCAATAATTCCAAATGC |
| comp107489_c1_seq2:392-393 | comp107489 | 392 | T | A | 54 | 0.722222 | 0.277778 | 13 | 0.0769231 | 0.923077 | GACTTAGTTGATGCTCTCACCTGCTCGAGAGAGATTCCCCTTTCTTGACCCAGTAGACTGCAGACACAACCCGGAGGAACTATAGTTATTCAGAAAACAAA[T/A]CTGTGGAGTAATATTGCTGAAGCTTTTTACACTTCTTGACGAGATATACTAGCATCTTTTAGAGGGATAGTGATTGAAACGAACAGAGAACGTCTGCAATT |
| comp103382_c0_seq1:1431-1432 | comp103382 | 1431 | T | A | 95 | 0.821053 | 0.178947 | 91 | 0.175824 | 0.824176 | CGTGATCCAATTGGAGCAGTTAACTATGTAGCAGCACGGCTAGATATTCCAAGCATTTTGAAGCTTCAGCATTCAGAGGTACAGAATGAGACTGCTTCTGA[T/A]ACGAGTTGGACGGCTTTCTATATATGTGAAGCCTGGGCTATGAAACGTGGGTATTACACACGCCGCAAGGGGGCTCCAGATGTGTCCAGATCAGCGAATGA |
| comp105402_c0_seq1:714-715 | comp105402 | 714 | G | A | 747 | 0.119143 | 0.880857 | 662 | 0.76435 | 0.23565 | TCAGTCACGGTGACAAGCACATCTCGTCCAAGGTTACTTAGCCGGTGATCACCCAGAGGCGTGACAGTGGGTGCTGCGCCTGCGGGTTAGAAAGGAGCAAG[G/A]AACATGGCTGGAAAAAGGATTGCTGTGGTGACTGGGGGCAATAAAGGAATTGGATATGGGATAATGAAAGAGTTATGTGCCAAGTTTGATGGCATTGTTTA |
| comp107861_c0_seq1:819-820 | comp107861 | 819 | A | G | 128 | 0.875 | 0.125 | 248 | 0.229839 | 0.770161 | AAGTATTCCCCTTCCCCGGCCAAGAGACCGAAGATCTTGTAATACTCATGAGCGAAGCGAGGAACTGATCCCAAAGAATTTGAACGAAGTCGAGGGAGAGG[A/G]TTTGATTGAGTGGGGTTCAATGGGGAAGGTGGGGGCGGGATGGGTAAATGGAAAAGGGGGGATGAATTTCCTCTTGCGAAGACACACGAACAGCCGCGAGC |
| comp103302_c0_seq18:1129-1130 | comp103302 | 1129 | A | G | 54 | 0.666667 | 0.333333 | 46 | 0.0217391 | 0.978261 | TGAACTTTGGAGGAAGGGAAGAAGAACTCAATTTCCCTAGTGGCTGCAACATCAGAATCCGAGCCATGGGCCGCATTGGACTGTTTATCCTTCCCAAACTT[A/G]GCGCGGATGGTGTCGGGTGCCTCTCGTCTTGCTAGTTCTGCGTCTGTAGGGCCTAAGGTCTCCCTCCATCGCTGCACAGCATTAGTTGTGATTAGCTCCAT |
| comp98623_c0_seq15:209-210 | comp98623 | 209 | A | C | 2 | 0 | 0 | 211 | 0.64455 | 0.35545 | AGTCTTTAATCTGCAATGACGCCTCCAAAGGGATTGGCCGAGAGACGGAAATCGGTTGGTCACGTGACGCGTTGACGATCGGCTTTTCGTGATTTCCTCAG[A/C]TTTTATCCTCCGAAACCGCCGCCAAAGCCTCCTCCACCTCCCAGTCCTCCACCGCCTCCGAAGCCTCCACCGATGCCTCCGCCACCATGACCTCCTCCTCC |
| comp107087_c1_seq10:879-880 | comp107087 | 879 | G | A | 34 | 0.735294 | 0.264706 | 66 | 0.0909091 | 0.909091 | TGCGCTTCGTCATATGAGTTCTTGCTTGCTTCCTCCCTAATGATGCTGTACTTGTCCACCAGCCCACTGCCGAAGGAGTCCAGGACATTGTTTGCGGCAGC[G/A]GAAGGACTCGTGGCACCATTCCCGAGGGCATTCCCTAGGGTGAGGTTGGAACTGCTGGCCGAGTTCACATCCTGAGCTATCCCAGAGTCCAGGGTGAGGCT |
| comp107566_c0_seq1:3579-3580 | comp107566 | 3579 | G | C | 53 | 0.886792 | 0.113208 | 33 | 0.242424 | 0.757576 | CACCAACCCACTTACACGAACGGCATGATGGCTTCCACGGCACGCCCTCCCGCTCACATCCTGTCCCGCCCACGCATGCCTCCCCCACAGCCGCCCACGGT[G/C]CCGCCCACAGCCAGACCGGCTAATGCCCACCAGCCGTCTGTCGTCACGATTAATGGCGTGACGGTGGCCAGTAATGGGACATAGACGCGCGAATCTCCTTT |
| comp101347_c0_seq3:637-638 | comp101347 | 637 | T | C | 1297 | 0.713184 | 0.286816 | 883 | 0.0690827 | 0.930917 | CAGGTTTGGCTGGGAGCAACCGACCAATCCTCGGAGGGAACGTGGAACTGGCTTGACGGTCGCCCCATCACCTCAGACTGGGCTAACGGGATGCCTGATAA[T/C]TACTCAGGCAATGAGAACTGCCTTGACCTCAGGATCAAGTGGCATCCCCCGCTCAACGATTCCGTGTGCGAAGTGGCTGAGCGATTTGTGTGCCAGTACAA |
| comp97599_c0_seq4:242-243 | comp97599 | 242 | T | C | 969 | 0.276574 | 0.723426 | 793 | 0.920555 | 0.0794451 | TGGCCCGAATCTGACAACACGAACAATGACCGTCGGTTTCCGTTAGTCTGAATTCTGTGAATTCCGAAGCAGTAAAGGGGACACTAGTAATGTGTGACAAA[T/C]ATGGAAGATATACCAAGATGTACTGTAATGCTTATCTGAAATAAAGACTTGATCGATGAACGTTATATAGCAGTTTGTTATTCTTTAATATTATAAGATAA |
| comp100893_c0_seq1:430-431 | comp100893 | 430 | C | A | 84 | 0.821429 | 0.178571 | 45 | 0.177778 | 0.822222 | TTACTGCCATACTATTATGCACAAAAATGAAATTGTACGTCTTAATTAATAACTTATGGAAACTTTGCAGAGAACTTTTAACTTGCTCCAAACTTACCTGT[C/A]GAACTTCTGGGTATAACTGTCTCTTTTGTCCACATAATCTACTGATTTTGTTAATATTGTAATGACCTGATTGCTAATTTATTTGTGTATACTGAAAATAA |
| comp99020_c0_seq10:280-281 | comp99020 | 280 | C | T | 55 | 0.236364 | 0.763636 | 100 | 0.88 | 0.12 | CATAATCGTTGTTGGTGGATGAGATGGGTTGATTGGAAGTGATTGTCTTAAGATAACAAATAAGTTGTCTTTATTCGAAATAGTTTAAGTGGCACAAATAG[C/T]TGTAGTGGAAAAGGCGGCTCTCAGTCGGCCGGAAGCGGAGTGAAGTGTTCGAGCCGCAGTCTCTCGTACATCGATCTGGAGAATCTGTAATCGCTGTACAA |
| comp104339_c0_seq1:3342-3343 | comp104339 | 3342 | A | C | 524 | 0.280534 | 0.719466 | 577 | 0.923743 | 0.0762565 | CATACATGTAAGTAATATATGCTTATATACCTTGTATACCTTATCAAGCATGCCAGTGTGTGCCTTTATGATCCTTTATGTGAGATAACAAACTGCTTTGT[A/C]TTCACTTTTCCATTCAGTTATAAAAGCTTAAGTGCTAAAGCAAACTAAAGGGTAAATACATTATTCAGAAGAGGGATACAGTCATTAATTAATGGCCATTC |
| comp103194_c1_seq1:1290-1291 | comp103194 | 1290 | A | G | 40 | 0.15 | 0.85 | 58 | 0.793103 | 0.206897 | CTCAATTACATAAGTGATTCCGTTGTTTGCAGTACTGCGAGAAGTACGAAACACAGAATTGAAATATGTAGATGAAGCAAAAATGAATCAGAAGGACAGAC[A/G]TTGAATACTCAAATGGAAAGTGGCAGTACAACACACAATATTACCTGAAGGTCTGCAAAGATCTCTTTACATGCATGTGGACAATCAAGCAAACATGCAGT |
| comp98913_c0_seq1:159-160 | comp98913 | 159 | T | C | 17 | 0.294118 | 0.705882 | 95 | 0.936842 | 0.0631579 | GGACCATGGAGCACCAGCAACCATCTGTAGAAGCATATTGGGAGCTTGCCACCAGATGCGCCATCAGTGCATAGGACGTTGCAGACGGCTCGAGGGCGATC[T/C]GTTTGCGTGGCCGAGCGGTATGTCAGCTATATGGAAACCGGATGGGTTGGGCTTCACACCTAGGCATTGCAGCTGCCTTACTTCCTGGCTTCGCACCTTCC |
| comp106927_c0_seq15:1641-1642 | comp106927 | 1641 | C | A | 53 | 0.830189 | 0.169811 | 16 | 0.1875 | 0.8125 | TCACCATTATTATTATCACAATCACTTCTTTTTATCATCATTTATCATCTGATTACTACTAATACTATTACTATTACTCCTATATTTTTGTTTTAGTCCTG[C/A]TATTTTCCTTCCTTTTTTTCTTGATTTTTCTACGGTAAAAACAATGCATGATATGCTGTAGCGCAATATTTTAGGAATAATTCTGAACTGATACTGTATAT |
| comp105932_c0_seq1:575-576 | comp105932 | 575 | T | C | 254 | 0.925197 | 0.0748032 | 506 | 0.282609 | 0.717391 | ACACTCAGTCTTTCCCTCAGAAGGACTTCGACACCCGACCTTACCCTCAAAATGACCTTGATTCCCGCCCTTTCCCTCAGAATAACTTCGAAACTCGACCT[T/C]ATCTTGAGAATGACCTTGATTTCCGACCATACCTTGAAAATGACCTTGATTTCCGACCTTATCCTCAGAATAACTTCGACACCCGCCCTTACATCCAAAAT |
| comp107240_c0_seq4:1271-1272 | comp107240 | 1271 | T | C | 116 | 0.655172 | 0.344828 | 79 | 0.0126582 | 0.987342 | AATATAGTTACTAATAATTATTTATTTGATGATAAGTGCCAGTGTAGTCCTGTAAAGGATAATAACTAATTGAAGCCAGTGACTCATAGACCCTTTATATC[T/C]CAGAATTATGATTGAGTGATTGGCTGTGTTGTTACAGCTCACTGAAATACTTGTTAACTGCATTAAAACAGAAGTCAAGATATTATGTATCATTATCTGAT |
| comp99738_c0_seq2:2878-2879 | comp99738 | 2878 | T | C | 77 | 0.701299 | 0.298701 | 17 | 0.0588235 | 0.941176 | ATCTCCATTTCAAAACCTCAACCAGTACGTTCCGACCGTGGTCGTTCACCGCCACCTGGTACCGGAATCCGTTGACTGTGTCCCTGGTGAACGGCATTGTC[T/C]TGGTGTTCGTTGTTAGCGGCGCCCAGCTGGGACTACGTGCTTTGAGAGCCTACCATGGCCTTCGGGGTATGAGGACGGCCTAGATAGACGTTTCCACTCCT |
| comp97950_c5_seq1:309-310 | comp97950 | 309 | T | C | 308 | 0.827922 | 0.172078 | 275 | 0.185455 | 0.814545 | AATCTCTACATGATTGGCCAAGATCGCTAAAAGAAATAGTGTTAAGAAAAACTTGTTCAATTCCTGGCACAGGAAGAAAAAAACAAAAATGAAGATTATAA[T/C]AATGATAAATACATAAAACAGTGTCAAATTCAACACCAGTACAAAGTGACGGGCTAGAAAACAATAACTGTTTGTTCCATGACAATGAAGAGGAAGCAGGT |
| comp106082_c0_seq1:2454-2455 | comp106082 | 2454 | A | G | 60 | 0.733333 | 0.266667 | 11 | 0.0909091 | 0.909091 | CGTAAATGTATGTGTAAAAACGTGTACAAAATCCAGTTATGAAGGCTGTGTATCTAGATTTCTGCTATAATTTTGGTTTATTCTTGGTGAATTTTTAAACT[A/G]CTTGTCAGACAAGGTTAGCAGAATTCAGATTTGTTTCTTATATTTAAAGAAAACTAGTGCAAGATGCAATTGTGAAGATTGTATATTAAGATGTATGTTTT |
| comp107924_c1_seq1:4643-4644 | comp107924 | 4643 | A | G | 369 | 0.815718 | 0.184282 | 242 | 0.173554 | 0.826446 | TCCGTGACGCTTTTCTCCGCGCCGGTGACGTCGAGGGTCACCACCGCGTCTTCCATGGCTTCTGTGGGCGTTTCCTCCCCGCTGGAATGCAAGGGGGGCGG[A/G]GAGGACAGCCGCTGTGGGGTGTGGACTGGGGTGGAGTCGCTCGACACCTTCGTCTTGAACGCGTCGACTGACTGCTCTAACTTCTTGAAGGCGTAAGTCAG |
| comp106332_c4_seq1:103-104 | comp106332 | 103 | G | A | 31 | 0.225806 | 0.774194 | 53 | 0.867925 | 0.132075 | ATAAAGTATGGAGATTTATATAAGAAGAAAATGAAAGGTTCTGATAAAAATGAAATGAATGAAACAAGGTGAAGAATAGGGATAATAAACAGGTATGAAAG[G/A]TTGAGTAAAAGGTATGGATGATGCACTGAATAAACGAAGGGGAAATTATAAGAACAAGATAACGAAAGAAAGAGAAGAAAGGTAAAAATTAATAAAGTAAA |
| comp105945_c0_seq3:1319-1320 | comp105945 | 1319 | A | G | 440 | 0.85 | 0.15 | 101 | 0.207921 | 0.792079 | TCAGATGTCTTACACCCTTTGTAAGGATCTACAGTCGTGAATATGCACCGATCACAGGGCTTCAGCCTCCTCAGCACCACGCTTTCTTCGCCTTCCCCTCC[A/G]ATCCTGACGAAGGCCCAGTCGTCCTCATCATATGCCGCTGAGCCCTTGACCACAACGTTGGCACGGAACCAGTCGATCGTGACAGGTTCGGCCAGGCGGGT |
| comp105707_c0_seq1:718-719 | comp105707 | 718 | T | A | 53 | 0.886792 | 0.113208 | 49 | 0.244898 | 0.755102 | GTAATCAATAGACAATGAAGATATTCAATATGTCTCCAAATATGTGTAGAAATAAAATTCTAATCCGTTTTAGTAAGCTAAAAGCACTGCTAACAAATAAA[T/A]TTTCATTCCCCCCCCCCTTCATTTGTCTAGCTAACAAAGAGGGAAAACAAGATGCTATAAATGCGGTACGGCTTTGCCATTTTCTGAATATCTGAATGGTT |
| comp99330_c0_seq1:1358-1359 | comp99330 | 1358 | T | G | 51 | 0.823529 | 0.176471 | 33 | 0.181818 | 0.818182 | GATTCTTTATATAGATTTGACTGAAGTGTGATCAATGCAGCTCATCAAAATTGATACCAGAGTTACAAATGAAATGGAAATTAGATTTGAATGTCAACATT[T/G]TTATCAAATTTTCACCATGAGAATGTAATTGGCATGTAATTTATGATTTTTTTCTTTAATATTGCTAAACCAAGTTCAGTTGTAGGTGGTTGTGACTATGA |
| comp103343_c3_seq6:165-166 | comp103343 | 165 | G | T | 66 | 0.863636 | 0.136364 | 45 | 0.222222 | 0.777778 | TGAAGTAGATTTTTTATCTGATTTATTCATATATTCTTCTTTTATAATGTGATTTTTGTGGACGACTACTTGCCAGAGGATGAATTGCTTTTGAAAAACGA[G/T]AAATTGAATAAGGAAATATGTCCCTGAAATAAGAATATGTATGGATATGCTATATTCTTTAAAGCTCAAAATAATTATAATAATAACAATGATAAACAATA |
| comp106961_c0_seq1:2246-2247 | comp106961 | 2246 | A | G | 56 | 0.75 | 0.25 | 46 | 0.108696 | 0.891304 | CCGCTTCCAAAATCGGTGCGGTTCTTGTCGCACACCAACTGGAATTCGAACCGCGAGTCCTCGACTTCGAAGCCTCGAAACCCGAACCGGCTCCACACGTC[A/G]GCGATCCACCTCGTGTAGGAGTAAGGCGCAACAGTTTTTTGGACAGCGTGATGCTTCCCTTCGACAATCCCTCTCTTGCGGGTGCCAGGAGTGACTCGACG |
| comp96405_c0_seq1:160-161 | comp96405 | 160 | A | T | 66 | 0.651515 | 0.348485 | 96 | 0.0104167 | 0.989583 | AAAAATATATATTACAGCTCCGTTATTACATGTTGCAAGACAAGGCATGCAAATTACATGTACATTACCATATGACACTTAAACTAAACCTATCTCATGAC[A/T]TATCAGCCAGAAGAAAAAACTCCTATGGGAGAAATAAGATTCACAATAAACATATATATTTCTTCTAACTTCCGGGTTTAAATAATCGCATTCACAAAAGG |
| comp101723_c0_seq3:295-296 | comp101723 | 295 | C | T | 96 | 0.916667 | 0.0833333 | 29 | 0.275862 | 0.724138 | CGGACTCTCTTCCTCTCTCTCCCCCTCCTCCACGTCCTCCTCATCTCTCCTCCATCCCTTTCATCTTTAAGACATTAATCAGAATGAAATAAAAAAAGATG[C/T]AAACAAAACACCCCACTTCTAAAAAGACAAGAAAGCAAAAGTCCTTCCCTTCAGGACCCGAAAAGAGGACGAGGAGAATCCGCCGAAGGACACCTCCCCTC |
| comp107570_c1_seq1:1352-1353 | comp107570 | 1352 | A | T | 110 | 0.854545 | 0.145455 | 131 | 0.21374 | 0.78626 | AATAGATATTATAAAAGAAATATTTGAGTTAAAAGCATATCTGTTCTAAGTAAATTTTAGGTCAGATGTAATATATTTTCATATATGCTGCAAATGGATAT[A/T]CCAGAATTTGTTTGTGTATTTCTCTGAAATGTCTTTTACTGTTGGTGAATGATTTGGCTTTCATTAAATAGGCATACATGAGTGAAAAGTTCAATAGGGAA |
| comp97558_c2_seq5:554-555 | comp97558 | 554 | G | A | 398 | 0.773869 | 0.226131 | 323 | 0.133127 | 0.866873 | GCTGCCACGGGGCCGGTCCACTTGGGCTGGTGCTGAGGGGCAGCGTGGGCGTGGTGGTAGGTGGGGGCGTAGGAGTGAACAGCGGGTGCAGCACCCACTGT[G/A]GCCCTCAGCTGAGCCTGGTAGGCGTTGTTAAAGGCAGCGCGGGCGGCAGCCACCTCGTGGGTGTCGCCCACGGGGGTGATGGTGCCGTCGACGCCGGCGGG |
| comp107551_c0_seq19:554-555 | comp107551 | 554 | G | A | 80 | 0.25 | 0.75 | 73 | 0.890411 | 0.109589 | AGAGAGGCTGACAGCATTCAGAGGGAAGCCCTTTGCCAAAGGAGTTAATGTGCTCTTCTTGATACCATCAAGGTAACCTCTCCTTTTAGGTGATGGATTGA[G/A]TTCACAAGGATTGATTTGATTTTCTATTGTTCTTACTGTGGTGGGAGATGGAATTCCTTAGCATTTTTAGCAACTGTTTTAGACCACACACTGAAGAGGAA |
| comp98197_c1_seq1:237-238 | comp98197 | 237 | C | T | 485 | 0.83299 | 0.16701 | 436 | 0.192661 | 0.807339 | AGTATCAAGACGACGTCTGAAGCTGTCCCGACTCGCTGGCAGGTTCGAACCAAAGTCCTTGCGTATACTCGTGTGGCTTTTGGTTCCGAGTGCATTTGCCC[C/T]CTGGAGTCGGAGGCGGAGGACGAAGTCGAGAAATGATAAGCAAGTCAGAGAGAAGTTGCCATATGGAGAATTTGCCAGTATCGTGCAATAAAATGAGTTGG |
| comp104543_c0_seq8:674-675 | comp104543 | 674 | A | G | 60 | 0.8 | 0.2 | 25 | 0.16 | 0.84 | TGGAGGTTTCAGCGGAAGCGGCTTCGTGTCGGGTGGATTCGGGAGCAGCGGAGGTGGAGGAGGCTACGGCCACAACAGCTGGTAAATAATTGCGAAGAGGA[A/G]AGCGTAAAGAGAAGAGCAAGAAGTGAAACAAACAAACAAAAAATAGCAGCGGCATTAGAAACATAAAACACATACAAACAAGCACAAAGACAAACATACAA |
| comp94346_c0_seq1:999-1000 | comp94346 | 999 | G | A | 108 | 0.916667 | 0.0833333 | 112 | 0.276786 | 0.723214 | ATGTTGCTAAACAGAACTGTCAGTTATCAGATTACCTTTAGTAACTCAAGAAAGCAAACCTATCTCGATGTCTCTCTCACTTTTGTTCCAATTTGTATTTG[G/A]CTTTTATCCCTTCCTTTTCCAGCTCCTTTGAACATATGGGACACACTTGTGATATCATACATCTAGGTACGACATTATACTTGTGTGTTACAATGTGAGGG |
| comp100922_c0_seq1:1992-1993 | comp100922 | 1992 | C | T | 85 | 0.917647 | 0.0823529 | 72 | 0.277778 | 0.722222 | ATATCAAGAAAAAGAGTAAATATGTGAGATGGGTTGGACTAATAACTAATAATAACTAACTCCTTGATGACTAGGCATTGGTGGGGCCATTCCTGTGTAAA[C/T]GCAATTAATAACTAAAATTGCACATAAGTTATGCCATCTGCGGCCCCTGGAAAGATAGTTACATAGAAAAAATGAGAAATGTATTAAACCAATAATTTCAG |
| comp97964_c0_seq1:441-442 | comp97964 | 441 | G | A | 87 | 0.816092 | 0.183908 | 51 | 0.176471 | 0.823529 | GAGTCCGAAAAGCGTCTCCACGAGGCCGATGCGTGACACCCACTTGGCAAGCCATGCTGCGTCACCAGGTGTCTTCAGCTCCCAAGCCTAACGGGAGTGGA[G/A]AGGCAGATACCCACTGGCAGAGGCCGAAAGTCAGTGCTAGGCCTCGACCCACAGGCCGGACAGAGCCACTTCTCGCTGTGAACTGACGTCTCTTCGTCATA |
| comp101782_c1_seq11:574-575 | comp101782 | 574 | A | T | 1456 | 0.0673077 | 0.932692 | 3175 | 0.706772 | 0.293228 | GCCCTTCGCAATAACTCATACCGCACGTGATAAATATATAAATACAGAGGACAACATAATAAGTTGATTCTTCTTGCTGTTATTATTCTTCAAAAAAAAAA[A/T]AAATCTTATTGCAAGGGGACCAGACACGAAGGACCCAAGAGTTTTGGTTTGTACTCGCACGTAAAGAAGAAACAAAACGCATAGTACTTGATTTCTTTTCG |
| comp101664_c0_seq5:914-915 | comp101664 | 914 | A | G | 434 | 0.64977 | 0.35023 | 282 | 0.0106383 | 0.989362 | GATTGAGGAAGGACTCCTGCCAGCACCGGGAGGCGAAGAGAGCGGGTCCTCTGCAGGCGACCGTCGTCAGGAAAAGGAAGGAGTGGAAGGATCTCTCAAGG[A/G]CAAACCGACAATGCTGGGCTTCAGGGACCAGACCCTCATCGTAGGACCTATGGACGCAGCCTCTAAGACGAAGGTGGAGGAGGACGTCTGGGCAGCCAGGA |
| comp100148_c0_seq2:437-438 | comp100148 | 437 | A | G | 140 | 0.792857 | 0.207143 | 13 | 0.153846 | 0.846154 | ACGAGCTTGATCATTGCTTTGCCATTTGGAATGTCAACATCTGTTGGGGCCTCATCATCACAAAACTTTTGACCTTTTTTGAATCCGCGCCCTCGCTCTCT[A/G]CCTGAGAAATATCTGGAGTACCGGAGAAATCTCTTCTCGCGGTTCAGGAGGAGCTGGCTGAGCGGTCCCCCGAGAGGGCCATTGGCCGGAGGTCCTCGCTC |
| comp102538_c0_seq3:2694-2695 | comp102538 | 2694 | T | A | 75 | 0.666667 | 0.333333 | 36 | 0.0277778 | 0.972222 | TTGGTCAGCTGCTGCATCAAGGACTGGATAGCATCAAACTGCTGTTCTGGGGGCTTGTCTAGGGGAATACCTGCAGCAGGCATTCCTGGCACAGCACCTCC[T/A]GAGACAGCCACCCCTAGTGGGCCACTAGGAGGCACAATTGGTCCCATGCCCGGTTGCTGGCCGCGCTGAAGTTGCATTAACAACTTCTGGAGAGGATTTTC |
| comp87853_c0_seq1:128-129 | comp87853 | 128 | G | C | 171 | 0.888889 | 0.111111 | 8 | 0.25 | 0.75 | GTTCGGGTTCGGCCGCCGGTTCCGGTGTCGGTTCTGCCTCGGGTTCCGCTACTGGTTCTTCGGTTTTGGCCATGTTCATCATGTGTTTCATTTCCATTTTC[G/C]TCATTTCGTCCATCATCGGAGGCATCATCGTCGTTTCAGATTTGGCTTCCATCTCCGCCAGCATCATCTCCATCTCTGGTGTCATGGTCATTTCTTCGTTC |
| comp102532_c0_seq11:351-352 | comp102532 | 351 | G | A | 359 | 0.70195 | 0.29805 | 222 | 0.0630631 | 0.936937 | CGGAGTCGATCTCCTTCTGCGCCTTGGCCTGCACTTCGGGGTACTTGGCCAGGTAGTGGATGGCCCAGCGGATGGTGGAGGACGTGGTCTCGCTGCCGGCC[G/A]CGAACAGATCTCCGAGGAGGTTCTTCAGGTCAGTAATGCTCATGGTAGACTGAGGGTCGTTCTTCTGGGCTTCCATCTCGATCAGGTAGGCGTCGATCAAG |
| comp104925_c0_seq12:1449-1450 | comp104925 | 1449 | A | G | 257 | 0.723735 | 0.276265 | 200 | 0.085 | 0.915 | TGAATGAAGTCTTTTTTGCTCTGACATCCAGCTATTTTGAATAAAACAAAACAAAGTGTGCTTATCGTCACGAGATAAAACTGGCTTGGGTGTTTCTCCCA[A/G]GTCATGTCAACAACAGCTGATGAAAGCAGGCCAAAGCAAGAAACATTGTAATCTACGTATATTCGTGCTGCCACCGAGTGAACTTGGCAGAACACAAGAAC |
| comp106544_c0_seq37:1056-1057 | comp106544 | 1056 | C | T | 53 | 0.849057 | 0.150943 | 19 | 0.210526 | 0.789474 | TCTGCACTAAGAAAATTCCTCTGGAAGGCGTTCATTGCTGCCTTCTCAAAATCTGTCATTATTGTCACTGGATTGCATGATAAACCAGATGGTATTAGCTT[C/T]AACACCTCGTACATTTTATTATATATCTCCATTCTTTCCTGACAAAAATCCATAGACGCAAGGATATGTTATACCACCAAAGTATGCATGGAGTGTGTACT |
| comp100222_c0_seq5:249-250 | comp100222 | 249 | G | T | 28 | 0.785714 | 0.214286 | 163 | 0.147239 | 0.852761 | GTTGTATATTTTATAATCTTATTCCAGCAAGAAAATTTCCATAGTTTCGAATATAAATAACCTTATAGATAAAGATATACTCTAAAAATCGAGATATCGAT[G/T]AATGGAATACCCTAGAAGCCATTGGGAGCGCCGTAGAACTGGTTGGGAGCCTGCACGGGGGCCTGGGCTGAGGCCTGAGCCTGAGGGGCGCCGTACGAGGT |
| comp99185_c8_seq1:135-136 | comp99185 | 135 | T | C | 577 | 0.861352 | 0.138648 | 251 | 0.223108 | 0.776892 | AACAGACAGACGGGAAGCTCGTGATATCGAAGTAGTCATTTCACTTCTATTCGGGGAATTACTCGCACATATAATACTCGCCTTCGAGGATGCAGTATGAA[T/C]ATTTACTCAAGAAACACGCATTTCCCGAAGACCTTTCTCGCTGCATTGCCCCTTCATGCCCCGAAGAAGTAATACAGGAAAAAGGTATTCTGGATTATCGT |
| comp106356_c1_seq2:921-922 | comp106356 | 921 | T | C | 42 | 0.261905 | 0.738095 | 60 | 0.9 | 0.1 | AAAACTATAAAAGATCTATGTACATATACATCAAAATCTTCATATCTCATTCATGAAATACTGTTTTATCTTTTATTATTGCTACTACTTTTACTATCATA[T/C]TTGCAAACAATACTTGTCCTTTAACTACTTTTACTTACAAAGGTATGCCCATTTCAAAAGTAAATACAAACCTCCCTTTTTTCACTACACACCTGCATGTT |
| comp105039_c0_seq3:341-342 | comp105039 | 341 | T | C | 306 | 0.931373 | 0.0686275 | 283 | 0.293286 | 0.706714 | AAAAAATCATTCACTGTAAAAATAATGCCACAACTCTGAACATGTCATAAAGAAAGAAAGATAGAAAGGAAGATATATATTTGTGCTGATATTTAAAAACT[T/C]TTTTGACATGTACTATTTTCAAAATGAATATAATAACAAACATTATGCATCAGTGCATTCACAAAAATCTCAAATTGCACAAACCAACAAAGAAGTTATAA |
| comp107784_c3_seq3:1732-1733 | comp107784 | 1732 | C | T | 849 | 0.126031 | 0.873969 | 831 | 0.762936 | 0.237064 | GCCTTAGCTACTGTCACGGCTATTATTGATGGCACTGGATCTATAGGTGCTGCTGTTGGACCAATGATTGCTGGTCCGATCTCAGCAGCAGGCTGGGACAA[C/T]GTCTTCTACATGCTCATGTTTTCTGATCTCTTTGCTCTCATACTTCTCCTCCGTTTGGGAATGCACGAGTTGAATAACTGGTGGATGGAGCGACGCAGGAG |
| comp93095_c0_seq11:757-758 | comp93095 | 757 | A | G | 59 | 0.220339 | 0.779661 | 14 | 0.857143 | 0.142857 | CCGATGGCTTACTTCCAAGTGTACTCGGCGTCGAAGGCCTTCGTGAGCAGCTTCAGCCGCGCCCTGCGGGAGGAGTGCGGCTCTTCGGGCGCGGCCGTCCA[A/G]TGCGTGGAGCCGGGCGCCGTGTCCACCAACATGACTTCCTTCGACAAGGACATTCACACCCCAGGGTTCTTCACCCCGACCGCCGACGCCTTCGCCGGCAG |
| comp104474_c0_seq5:177-178 | comp104474 | 177 | A | G | 12 | 0.25 | 0.75 | 53 | 0.886792 | 0.113208 | CACGTCTGGACTCTTGAGTGTCAGGACACGTTCTGTACAGGACCACTTTCTGGAGGATATAAATGCTCCTTGTAGCTCAGATATAGTTACTTGGGCTTCAG[A/G]GAGATCTACACAACAATAATGATTCGCCTGGTGGTAGTGTCAGGTTTATTGTGCCTCTTTTCTGCATCTGTGCAAGGAACTGACTGCCCTGGAGGACTTGT |
| comp105448_c2_seq1:295-296 | comp105448 | 295 | G | A | 72 | 0.722222 | 0.277778 | 35 | 0.0857143 | 0.914286 | AAAAAAATCTTGCCGTAGCGGATGTCAAATCTCGAAATAGTATAGACGTCTGCTATCAATTCCTTATCGAAACCCAATCATATATACAATGAAATACAAGT[G/A]TAAGAAATGGAACAAAAAATACTAACGTTTCGAGAAAGACTCTTCTCCTCATCAGGCAATAAAAGTGGATCCACTTTAGTTTGTCTGATGAGGAAGGTCTA |
| comp107790_c0_seq2:557-558 | comp107790 | 557 | G | T | 143 | 0.636364 | 0.363636 | 2 | 0 | 0 | TATTTATCTGTCACTCGATAGGTTATTTCTCCTGCTGTCTACTTGCTGACTGAGTCTAATCAAGTAGATTAGCTAAATGCTCCCACAAGCACAGTTAAACT[G/T]TCACGTTAATTACCTGCCAACTAGCTAATTTGGGTTATTTTCTGTCAGTATAAATAACTGATCCATTAACGGTTATTCCCACAGAACTCCTTTTCCATTGG |
| comp101409_c0_seq3:147-148 | comp101409 | 147 | A | G | 110 | 0.863636 | 0.136364 | 44 | 0.227273 | 0.772727 | GAAATGTTTTCCGATCAAATGGAGATGGTTAAGGACATGCTAAAGCGAGTTGGCGGCTACGAAAGGGCGGAGAACTGGCTCAGCGGAAATAAAGGGAGAGT[A/G]ATAGAGATAATGGAAAACAACATCAGAGGAGTCCCCCCCTTCGACGTCTTGTGCCACGGCGACATCTGGACCAACAACGTACTCTTTAGATACAACGAAAA |
| comp107676_c0_seq3:1339-1340 | comp107676 | 1339 | A | G | 108 | 0.731481 | 0.268519 | 63 | 0.0952381 | 0.904762 | CCAAACACACCAAGAATAATTGGAACCTTATGAAGCAGCATTTGCAGTTTAAAAGGAGTATTCAGACTATTAGCTTCACACCATCATTAAATGGTTAAAAT[A/G]ATGTAATATAAGTAGTAAGGGTATGTATGGCAGAATAAATATATAAAATAAAAATTTTCAGCTGGGTTTCCTTTATGCAACTATTTGCATATCACTTTTTA |
| comp102041_c0_seq11:390-391 | comp102041 | 390 | G | A | 93 | 0.860215 | 0.139785 | 125 | 0.224 | 0.776 | AAGTCAGCATCCGCAGTCGGCCTCACAGAGCGCTTTATTTCCATCCCGTTGACCCATAAACCCAAGGATAAAGTATGGGCTCCACACCGCACACGTTCCCC[G/A]GCATCACCTTCCTCTTGCTGACTCTGGCCATCGCGGCGGCAGATCAGCGGAGACACCAGGCAGGGTTCCACAGCCAACCGGGGGTGAGCTTCGGCGAGGTT |
| comp103123_c8_seq1:459-460 | comp103123 | 459 | A | T | 80 | 0.9125 | 0.0875 | 76 | 0.276316 | 0.723684 | TTTCTGTCCCTCTTTCTCTCGAATTCCACGATTCCTGCCGCACAAATAGTGATGATTGATGGTGAAGGTGATAGACTGAAGATGGTGAGGTAATTGAACAT[A/T]GCTCTGACACACCTCTTGGTCCTGCGTATGTGATTCCTTATCTTATTTTGATTTTCAGAAAATAAAATCCCATTTATAGTTTGTTTTTTGTATCTCCTTTC |
| comp100626_c5_seq1:485-486 | comp100626 | 485 | G | A | 881 | 0.666288 | 0.333712 | 958 | 0.0302714 | 0.969729 | CCAAAGCCATCTTTTACTTGAATCATCATGAAAAGGTTGTTAAGTATTCTTTAGCTTTTATAATAGTGAGAGGAACTGTCTAACTTGTTTCCTGATTTTTT[G/A]TTCTTTTTCTTTATTTCTTCTTTTCTTGCAAAACATGTTACCATAGACTTTAATGCTGATACAAGATGTCTAGTTCCCCTTCTTTTCCGTCATTTTGGCAG |
| comp105372_c0_seq2:117-118 | comp105372 | 117 | G | A | 104 | 0.0384615 | 0.961538 | 132 | 0.674242 | 0.325758 | AATGGAGGTTAAATGAAATGTGTGGGTGCAACAGTCTATAGAAAGGTATGAATGAAAAAAATAAAGGGTTAATTGCAAAGCGTGATCAACATGATGGTTAG[G/A]GTAATGTAAAGAAAATAAGTAAGTCAAATAAGTAAATAAGTAAATGGACGGTTGAATAAGAGGCGTGGTTGATGCACTGGTTGAAGAGATATTTTAAGAAA |
| comp90026_c0_seq1:115-116 | comp90026 | 115 | C | G | 168 | 0.702381 | 0.297619 | 15 | 0.0666667 | 0.933333 | GTCAAATCTTTTTATTCATGTGTTTAAACAATATATAAAAACTATATAAATGTATTGGAACGGTACATAAAACAAAAGTCTGCATGAACTGTGTACAAGCA[C/G]AAAGAACAAACGAAAACAAGATCTATTTTATGAAACGACAAAGCACCAACAAAATAGAATCAGCAGAGGAAACACCAGCCCTGCTGAAGGGACCTGCAACG |
| comp107555_c0_seq2:6194-6195 | comp107555 | 6194 | G | A | 69 | 0.826087 | 0.173913 | 21 | 0.190476 | 0.809524 | TGTGCGAGTTCAGGGATCTGACAATAAGAATGGTCTCGAAATGATACGGGAAAGTGCAAAATCAACTGGGAAAGTATTTAACGTCCTCTAATTGGTACCAT[G/A]CGTAATTTATGCCTCCCGTTGTTAAATTATATTGTTGAAATATTAGGAATTAGGAATTTTAACAGAAGGGGAATTTGCCAAATATTTTCATTCCGGTATAA |
| comp106964_c0_seq1:533-534 | comp106964 | 533 | G | C | 112 | 0.0267857 | 0.973214 | 154 | 0.662338 | 0.337662 | CGAATGAACTTTGTAAACATATGGGTTAAGTTCCTTATTATTAAATGTAATATGCTTGTGCTACAAAATAAATAAACATAGTTTATCAATATACTGTTCCT[G/C]TTACGTACCAATCTTCTATCACATTTCTGTCTGCCAAGAGGTATATAGTCATTATGTCTACAATGAAAAGTGAAGGACCTGATAAATTTCCAGAAAATGCT |
| comp100179_c2_seq1:849-850 | comp100179 | 849 | T | C | 92 | 0.902174 | 0.0978261 | 15 | 0.266667 | 0.733333 | TGAGAATTGGGTGCAATTAAGAGTACTTTTGTTCTTATCTTTGATGTTGAAGATATGTCACACAGGCTGTGATAGAAGATTAGAGATTTAAGAAGAAAATG[T/C]AGAGTATTATGTTACACCAATGTGATATAGAGCAGCACAAGATTTCATTTTCCCTTGGCAAGGATAAGGTGCCACTATCGTCTATTACTATCTGGGTATAA |
| comp100043_c0_seq2:602-603 | comp100043 | 602 | A | G | 71 | 0.746479 | 0.253521 | 27 | 0.111111 | 0.888889 | GGTGGCGGTGGCTCGATGCCCTTCTTGCCCTCAGCAATGTAAGACTTTTGGATAAACGAAAACCCCCCGTTTCCGCCTGGCTTAACTTTCCCCTTTTCTAA[A/G]ACGTTAAGCAAGTTTACAAATCCATGTATTCTAATCAGTAGGGTTTAAGGAGTGGAGATCAAGGGACAGCCTGAAGTGCATATCGTCTGTAGGTATGTGCA |
| comp105055_c0_seq4:881-882 | comp105055 | 881 | T | C | 1705 | 0.849853 | 0.150147 | 275 | 0.214545 | 0.785455 | CCTCCAATAATGGCACCAATGTTGCGCATTGCCGAGTCGTTGAGGGCCGTGTAGCAACCATCAGTGAAGATGGTTTCGCTACCATCAGTGAAGATGGTTTC[T/C]GTCTTGTTGACGGAGAACACATTATGGCCACATCCTGTTGAAATTATCTTGCAGCACGTGTCAGGTACTCCAGTGACATTCTTTCCAAAGGTTGTCCCTTC |
| comp107113_c0_seq10:2743-2744 | comp107113 | 2743 | T | A | 418 | 0.935407 | 0.0645933 | 593 | 0.300169 | 0.699831 | CTCACATTTTGACGATTCCTACTAGCTGTGGAGATCATAGAGAGTGCCTCCTCCATCATGGCAAGCCTTGCCTTCGATTGGCTGATTTCTCTGTATTTGGT[T/A]GCAATTTCAGGATTCTTTCTCTCCGCTTCAGATAGCTGGATCTTGCGTCCGTCCACACCCACAATCTCTCCCCCAACTGCTCCTTCCATCTGCTCATCCCG |
| comp103180_c1_seq12:2023-2024 | comp103180 | 2023 | T | A | 70 | 0.857143 | 0.142857 | 45 | 0.222222 | 0.777778 | CGCACCACAAGTGGCAAGCTCCTAACCTACACTGTGCTGCAGATCTTCCCCTTCACTTCTGAGACCAAGAGAATGGGCATTATTGTCAGGGAGGAGCTGAC[T/A]GGTGAGATTGTCCTTTATATCAAAGGTGCAGACACAGTGATGGCATCAATGGTTGAGTATAATGACTGGCTTGAGGAAGAGTGTACCAACTTGGCTCAGAA |
| comp106880_c0_seq5:1346-1347 | comp106880 | 1346 | T | C | 52 | 0.865385 | 0.134615 | 26 | 0.230769 | 0.769231 | GACTGACTGCTACCAGGAATGTTTCTTGTTACAGGTTGGACTCGATTCCACCTCTGAAGCATTTTTGACGATACAACCTGAGCATACACTGTGCCTTCTGA[T/C]GGTACCAAAAGGGCATCCTCTGTCAGAAGGTAATCCCGGGCATGTCTGTAGGTGTCGATGGCCCCTTCCCCAATCAGCTCTGTGTCAAACACCTCTGCCAC |
| comp107373_c1_seq3:399-400 | comp107373 | 399 | A | C | 211 | 0.886256 | 0.113744 | 143 | 0.251748 | 0.748252 | TCATAAAGAATCTAAAGATAAGCCTATTCATAACAAGCACTTGACATACAATCCTCGCAGGTCGTTCATCGTAAGCCACAGAGATAGTTGTTTTATAAATC[A/C]CTATCCCCAAGCCACAGTTTGAACGACCCGGAATATAGTTAACTGTTGCTTCCAAATTTTCTAGAAAAAAGGATATATATATTTTTTTACTATACTTATTT |
| comp102607_c2_seq1:455-456 | comp102607 | 455 | T | C | 55 | 0.254545 | 0.745455 | 54 | 0.888889 | 0.111111 | TTTCCAAAGCAGTTACATCATACTTCTCGCTTTGGCTTTAAACGCATCAGAGTTGTGTTGCAAAGATTTGAATCCCTTTTACAAACAAAAGAAATCAGAAC[T/C]GAAGGGAATTTAATTAACACATTCGATCATTTTCTTTTGTTGTGGAGATAAAAACAAAGATACATTTATGAGGGAAAAAGAGAAAGAGAGGGAGAGAGAGG |
| comp96489_c1_seq1:526-527 | comp96489 | 526 | C | T | 77 | 0.727273 | 0.272727 | 43 | 0.0930233 | 0.906977 | ACCTTGACCCATTTTCCTTTGCCTTCGGATGTACCTTTTTCCTTTTTCTTCCCCTCCTTATTCTCCTTCTTTGCAATCTTCAATTCCTTGTTCTTTTTCTC[C/T]TCTTTCTTCTCTTGCTTTCGCCTTTTCTTCAGCTTTTGCCTGTCTGCTTTTCTTGCTTCCTTTCTCTCTTCCTTCCTTTCTTTTGCCTCCTCCTTTCTGAC |
| comp106334_c1_seq1:773-774 | comp106334 | 773 | G | A | 101 | 0.653465 | 0.346535 | 52 | 0.0192308 | 0.980769 | GGATTTTCTGTAGTGTTTAGGCCCAAATCCTCATCAATATTACGCTTGATCAAATGCTCTGTCGATCTGCTTGATGGAGTCATTACCCAGTATCTGAGGCA[G/A]CAACAAACAACGATGAAAGAGATGAAGCCAATGAAGAGGACGATCATAAGTGCTATAAGGCCTGCTAGACGTGCATCCACCTCATCCCTCTGGGCTTCCAC |
| comp99772_c0_seq2:712-713 | comp99772 | 712 | T | G | 164 | 0.634146 | 0.365854 | 2 | 0 | 0 | TCTGCGTCGTTGGCTACGTAGGTGAACAGCGGAGCATCGGAACCTGTTTTGAGAAATTTCACCCAAAAACTCAGCGTGAATGCCTCCAGATCCGGTATATC[T/G]GTTATATACTCGACATATTGGCTGTCGGTGGCTGTTCGGTTGAGGAGAACCTGGGAGACTTTAACGGGCGGGCAGGAGGAAGCCAGTGAGAATAACTGATT |
| comp105495_c0_seq4:328-329 | comp105495 | 328 | G | A | 51 | 0.745098 | 0.254902 | 18 | 0.111111 | 0.888889 | GATGGTGTAAATATATAAAGTGAAGACGACATATTGGAGAAAGGAAGAAGAGAAATATAAGCGACAAGAAACGAGAGAAAGGAAGAAGAGAGATATAAGCG[G/A]CAAGAAACGAGAAGAAGGAAGAGTAAAAGACCAGGAAGGAGAAGTATATAGAATAGACCCCAAGAAACGAAAAGAAAAAATGAAGATGGATAAATGGAAAC |
| comp100077_c0_seq8:420-421 | comp100077 | 420 | A | T | 3621 | 0.85805 | 0.14195 | 1709 | 0.224108 | 0.775892 | ACGGAACAGCCAATTGGATAAGGCGAAAATGAAGAACGTAAAGATGGCTGGAAGAGAGAAAAGTAGATAAACTAAAATCATCTGAGGAAAAAAGTAAAAAA[A/T]ATATATATACGCTTGTTTAAAATCTCCGGATCAGATCCAGGAGATATTGATTTAATTTTCTATCGGCGTGAATATTCGAATATACTTCCGTATGTACCAAT |
| comp105666_c0_seq3:232-233 | comp105666 | 232 | G | A | 77 | 0.935065 | 0.0649351 | 73 | 0.30137 | 0.69863 | CATATCAGCATTTTTCTTCAACAATTTACATTCTATCTTAAAATAAATTACACATCATCTTAATTCAATAATTTACATTAAAAATTCGATCATTAATGCCC[G/A]ATTTTTTAATTATTCCTCTTTAATTAGAGCCAGTGATCAAGGCGCGACGTTTTGATCAAAGAAGCTCATGTTAGTATAACTTACTAAATAAAGTAGGAATA |
| comp102925_c0_seq1:113-114 | comp102925 | 113 | T | C | 176 | 0.130682 | 0.869318 | 174 | 0.764368 | 0.235632 | TATATCTATATGCTGCATTTTTTCAATACATTTTTTTTTTAACTCGCCAATACAATATCGGTTTCCAAGTGCCGTGACTCACCCATCCAGTCGCCAAAGCG[T/C]CCCTCCGTTGACAAGGCATATCCGGTGTCATGTCCGGCCGGCGTGCTGCAGTAGAGCTTGACAGCGTTGACGGCAGTGTCATCTGTGGAGCTGAAGATCTC |
| comp96081_c0_seq3:343-344 | comp96081 | 343 | C | T | 82 | 0.658537 | 0.341463 | 80 | 0.025 | 0.975 | CTCGGCAAGAAGAAGTGTTCCCCGGAGGAGGAGAAGATGAGAGTGCGTGCCCTGGCTACCATGAGGAGCTTCGGCACGTGCCCCCCCAGCATGTGCACCCC[C/T]GAGGAAGCCGCCGAGATGAGCACTGCAATGGGTCTTCTGGCGGAGAAGTACCCCGACCTGTGGGCCAAGCTGATCGCGGCCATGTTCGGCATCGACCTCGG |
| comp95224_c0_seq1:239-240 | comp95224 | 239 | G | A | 132 | 0.204545 | 0.795455 | 111 | 0.837838 | 0.162162 | CATTGCTGAGGACAAGTAAAGTGGGTGACATCACGGGGGTACGAAGGAGGAGCAAAAGAGGAGAGGAGGAAGAAAGGAAGAAAGGAGGAGGATTAGGAAAT[G/A]AGGAGGACGAGGAAGAGGAAATGAGGATGAGGAAATGAGGAGGAAGCGGAGGAAAGGAGGAGCAAAGGAGGAGGAAGAGGAAAGGAGGAAGAAGAGGAAAT |
| comp98800_c0_seq10:2218-2219 | comp98800 | 2218 | T | A | 23 | 0.304348 | 0.695652 | 112 | 0.9375 | 0.0625 | ATTTCTTCATCAGTGCATGGTAAGCACCAAATAATTCAAAATCATTAAAGTTCCATCTCTATGAACCAGTAATTTAAGCTTTTCCTTCTGATTCAAAATCA[T/A]CATTTGTTTTTTATCGAACTTGTACCAGATAACGAGAACTGTAAGGCTTCATTTGGTAAGGAAGTGATAATTAAACTTGGTACTATACAAGACAAGTGGGG |
| comp98219_c1_seq1:206-207 | comp98219 | 206 | C | T | 63 | 0.84127 | 0.15873 | 72 | 0.208333 | 0.791667 | ATAATTGTCTTCTTTTCGATGTGGTTCCTTTTATATATTTATACTGGAAAAATTTCGTAAACATATTTCTATCAAAGGGTTATAACAGAGAACATTTTATA[C/T]TTATAATTAGAATTATCACATTATAATGCAGTGATCGAGGACACGAAATCACAGTCACCAACATTGCCAAATAAATCATTAAAATTGTCACGAAAAGATTT |
| comp93756_c0_seq1:938-939 | comp93756 | 938 | T | C | 32 | 0.875 | 0.125 | 95 | 0.242105 | 0.757895 | GTAAAACCACCTCCGGGGCACAGGAGATCGCCTGCCGGTCGCTAGACTTCGGGAGATCTCCATTGTCATTTGAAATTGCTTTTGGGATGATGTGTGTACCG[T/C]TTTGCTGAATTTTGGGGTAAAACGCCCGAATTGTCTTGTATATAATGAGGTGGTGATGGTGTAAATGGCGTAAAGAATGTGGCGTCAATTCAATTACACCT |
| comp102845_c0_seq3:1257-1258 | comp102845 | 1257 | A | T | 219 | 0.799087 | 0.200913 | 66 | 0.166667 | 0.833333 | GGCTGTCACAAAAGAAGGGGCAAAGGGGATAGGAGGGGAAGGGAAGGATGGGCACAAGAGGGGAAAGAAGGGCTTTGAAAGTTAACAGCACTGTTTTTTTT[A/T]TTTTTTTTAACTGTTTTAGAATTTGCAAATATAGATGCCTCATTTACAAGCACTAGCAAAAATCCCAAAAAGCAAAATTCTACATCAAGCAAAATGATAAA |
| comp104953_c0_seq10:127-128 | comp104953 | 127 | A | G | 84 | 0.75 | 0.25 | 17 | 0.117647 | 0.882353 | TGTTTGAAGGTAACAAGAACTAAAAAGAAATTATGACTGTTTTGTTATTTTATATCTAAAATCAGAGATTTACTTAGTCTTAAGTCATAAGCAAAATTTTG[A/G]ATTTTTTTTTTATAGCCATGTACAGGTAATTAATGTCACTCATGGAAGTGTGACTTTTTACCACAAAAATATGAACATTCATATACACATTTTCCACCATT |
| comp104106_c0_seq1:363-364 | comp104106 | 363 | A | G | 79 | 0.822785 | 0.177215 | 21 | 0.190476 | 0.809524 | CCTCGGAAATTCCCCGGTGAGAGGCGCCTGGGAACCATTAACTACGGGCAATATCGCTTCGGCAAGAGAGGATACCAGAGGAAATGCCACCCGAGAAAAAA[A/G]CACTGCAGGTTACCTTAGAGAGAGAGAAAACAAGCAAACAAAGTGCTCAAAAATGGACATCATGAATCCAAACTTGCCAAGGCTATCCGTCTGGAAAGTAT |
| comp104107_c0_seq1:463-464 | comp104107 | 463 | G | A | 113 | 0.902655 | 0.0973451 | 270 | 0.27037 | 0.72963 | TGGTAGCTGGGTCTTTCGCCCTGATAGCGAACCCAGATTTCCTAAGATGGAGGAACCAGCTGGGAACGCAAGAGCTCGACCAGCTGACAAGCCGTCTCTAC[G/A]ATGTGCAGGGAAATGATCTAGACAACCAGCTGGAAAACGATCTTATTAACCAGTTGCCCGGTTTTCCTGAGAACCAAATGGGAGACCTGAAAGACGTCCGT |
| comp100643_c0_seq1:1457-1458 | comp100643 | 1457 | G | C | 65 | 0.276923 | 0.723077 | 66 | 0.909091 | 0.0909091 | GAATCTCATGAGACAAACGCTCGTAACTACACAAAATTTTCTTTCAACCCAGTATCACCGTTAAAAAGCAGTATACCATTTTAAACCAAAAATATGATATG[G/C]CAGACAAATAATTTCTGCCATGTCTTTCTGTTATAAAAATATTTACGTCCCCAATTAATGATATCTAATTATTAAAAAATGATGGGTTAATCTGTTGCTAA |
| comp95520_c0_seq1:815-816 | comp95520 | 815 | C | T | 1220 | 0.636066 | 0.363934 | 759 | 0.00395257 | 0.996047 | CTGGCCACACTGGTAAGGAAGGATGACAAGACAGTGATCACAGTGTTGCCGGTTGAAGAAGTGACCAAGCTTATCAAGGAACATGAGGTGCGGGAGGCTGA[C/T]GCTGAGGCAGCCAAGCAAGAGGGCTCCAGCAAGAAGTCCGACTCGTGAAGGACAAGAGACGTCACCTAGTCCTAAGGTCTAAGTAAAATGAGATGAAGGAG |
| comp105667_c0_seq6:101-102 | comp105667 | 101 | A | G | 807 | 0.915737 | 0.0842627 | 1550 | 0.283871 | 0.716129 | GGAAAGGTACCGATGAATGAAAGATTAAAAAAGAAAAAAAAAAGGAAAAAGAAGAAAGTAGGGGAGAAGGTAAAATGAAAGAGAAGACGGTGAAACAGAAG[A/G]AAAAATCAAGAGAAGACACTGAACGAGTAAGAAGTGGAAGAGAAAAAGGTGGAAGATTAGAAAGTGGAGGTCGTGTGGAGTCTCCTTATGCGCTGAGAATC |
| comp103228_c1_seq7:498-499 | comp103228 | 498 | A | G | 82 | 0.719512 | 0.280488 | 57 | 0.0877193 | 0.912281 | AGTGTCTCAGCATATCACCTTTGGCAGCCTGTCATGATGGTGAATGTGCAGGACATCTGTGAAAACATGAGGCTGGCCAGAGAGATGGCCCTTACCAGCCA[A/G]TATGAGACAGCTGCTGTGTACTACCAGGGAGTTGTACAGCAAATCCATAGACTGCTTGCAACCATACAAGAGCCCAACCGGAAGATAAAATGGCAGCAGGT |
| comp106990_c0_seq4:647-648 | comp106990 | 647 | G | T | 221 | 0.714932 | 0.285068 | 120 | 0.0833333 | 0.916667 | GAACATAAGAGCTGGAAACCGTGCTTTCGTGCTTGTTAAACTTGAAGAGCTTCTCATCTTTCTCCTTTTCTAAGCTTCCAAGAAGCTGTTTAGATCAGCAC[G/T]TGAGTGAGACTCAACCCCGAGTGCGACGAGATGCAGCAATCCGGCAAGAGCGAGCCCGTCCCCGTGCTGACTCGCGTCCGCAGCAAGACGTTCATCGTCAC |
| comp104122_c1_seq1:558-559 | comp104122 | 558 | G | A | 44 | 0.818182 | 0.181818 | 75 | 0.186667 | 0.813333 | ACAGCGCCATCCCCCACACGCCCCAGGCAATAGGCGTAGGGCAGTTCTTCGCTAATTTCTTTGCTAATCAAGCTCGCCAGAACAGCCAGAAGTTCCCGACC[G/A]AAGAAGAGGAAACAGCTGCTCTCATCTACAATTACGAACCTTTCTTCACCCAAGAAACCTCTGGCTTCGTGCAGGTATATCTCTTCCGCTGACAGCCCGGA |
| comp105535_c0_seq1:453-454 | comp105535 | 453 | T | C | 15 | 0.933333 | 0.0666667 | 53 | 0.301887 | 0.698113 | CCCAGGCACACACTTCAGACTTCTGAAGCTGTGGGGTTAAGGGTGAAACCACAATGGCTCGTCTTGTATTCTTAGTTCACGTTACGTCGCTCGTTATCGCT[T/C]GTTATCGCTCGTTATCTCTTCCATCCTTTAGTCCCGTTGCTTGTCTTAACTGCTGGTGGCGACATAGGAGCGAAGTTGCTGTGGGTTCGGCGGCGGTGACC |
| comp98226_c0_seq10:1472-1473 | comp98226 | 1472 | C | T | 74 | 0.837838 | 0.162162 | 92 | 0.206522 | 0.793478 | TGTAACAAGGCTTCAAGAAATCCATGACTGGGGAAGTATTACTTATAAATAATAGTAATTATAACAAAAATAACAATAAATTTCTAACAAAAGAAAAGATG[C/T]AAACGTAAATTTAGTTATGAGTTTGATGGAGGACTGCTTTCACCACTGCTTTTAAAATCATCATCACTATCTTCATCTGATGAGTCTGCAGTGTCTGTGGA |
| comp98921_c2_seq1:2436-2437 | comp98921 | 2436 | T | A | 66 | 0.909091 | 0.0909091 | 72 | 0.277778 | 0.722222 | CATCTTCTTCTTCTTCTTCATCTTCTCCTCCTCCTCCTCCTCCTTTTTCTCCTTCTTGATCTCTGTCTTCACCTCCACCCTCATATCTTTTTATCTGCACT[T/A]AAGGTTATTAGATTTTGAAAATTTCTTCTTTTGGTCATATGCGGTTTTAAAAAAAGGATTGATTCCCTTCTTCCCTTCCAAGATAAGAAAAAGACAATATA |
| comp91755_c0_seq1:103-104 | comp91755 | 103 | C | A | 105 | 0.27619 | 0.72381 | 54 | 0.907407 | 0.0925926 | CGGCGCAAAGTACACCATATTGTGATAACCAATGACATCGACTTCACTGTTTATTGTAAGAAAAAAAAATTAAGTAATCGGAAAAAGACAAACGGCTAATC[C/A]AAACAATTTCTCAGACTGAGAGAAGCAGATACTCAAGATCTCAAACACGATGGATGACGCTTCTTTGTTGTAATTGTTCTACAGACACTTTGAGGATGGCT |
| comp100115_c0_seq2:840-841 | comp100115 | 840 | C | A | 23 | 0.304348 | 0.695652 | 155 | 0.935484 | 0.0645161 | AGATCCCTGGACAGTAAAGCGGCGGTCGCCTGCCTGAAGAAGGTTCCTGCAGACAAACTTACGTCTGTACAACTTGCCTTTAATATATGGAACGGGAATCC[C/A]ACCGTAATGCTACCCAGGGTCGACGGAGAGTTCTTGCCTGACCATCCGGCTGTTTTATTAAGGGAAGGGAAATATAACAAGGTCGACCTCATCTCCGGCAT |
| comp102901_c0_seq1:4070-4071 | comp102901 | 4070 | T | A | 52 | 0.134615 | 0.865385 | 226 | 0.765487 | 0.234513 | GGTAAAATTCATGAATACAATTAGGTTCCAGTGTCATGAAATGCATTGGCTGAACACCAATAATTTATATTTTACAATTGATTGTAAATAGTATACTCAAC[T/A]TGATAAAAGTTTGCCAACTTTTGCTTAGCTTTTTATGTTATTTTCTTCACGGCAATACAACGACTACCATGATCTTGGGATCCTAAAGTCTTCCAGCATCT |
| comp113138_c0_seq1:188-189 | comp113138 | 188 | A | C | 111 | 0.918919 | 0.0810811 | 59 | 0.288136 | 0.711864 | ATATTATTTTCTATTTTTGTTGATACTGTGTGGTAACAATCAAATAAATCTAACAATAGTTTTGCTTTGTATAATGACCTAGATAAGCTCAGTAATATTGA[A/C]GTGCTATAATAAAATACAAACTTGTAAATATACCATAGTAATTCTAACATAGGTGCTGTTCTTGCCTGAAAAGAAACAAAAGCAACTGTGGAATATCAACT |
| comp103628_c2_seq6:819-820 | comp103628 | 819 | T | A | 108 | 0.777778 | 0.222222 | 68 | 0.147059 | 0.852941 | ATAAATATGGACAAATGAAACCTAAAATACTCAAAACTTCCACACTCATAAACCAAACTTTGTATAAATACTTAAAAAAGCAACGGTTACAGAAATCCAAT[T/A]ATCTACCCAACATTCCATTTCCTGTTAATCAAAAGCATTATTAACAGGCTTAAAGAAAAAAGATGTCTATGAAGTGCCATCAAGACTTAAAATACTCATAA |
| comp105590_c0_seq1:1055-1056 | comp105590 | 1055 | T | C | 60 | 0.8 | 0.2 | 59 | 0.169492 | 0.830508 | TCCTGTGTTAGGCATACTACGGAATAGTCGTAAATTTTTGTAATGCCGTCTATGTTCAAGTATTCTAACTGGCGACATTGTTTAGCTATTGCAACAAGGCC[T/C]AAGTTATCCAAAAACTGACAGTGGGACAAATTCAAGTGCCTCAGGTTATGAAATTCTGCAATTGCAAAATAGCACTCGCTGCAGCTGATTCGAGAGCCTTC |
| comp107688_c2_seq1:5273-5274 | comp107688 | 5273 | G | A | 51 | 0.784314 | 0.215686 | 13 | 0.153846 | 0.846154 | GATCTGAGTTATGGCATAGATGAGGGTCTTGTTAGAAAAAGTGGAAAACCACAGAGTATTGGTTTAGAGGATGCATTTAAGAAGGTAAGCTCAATTGCAGA[G/A]GTACGTATCAAATATTACACTTTGCTAGAACGGCTCTTAAAACTCTTAGCAGAGGATGGCCGTGTACCTGGAGCCCTCAAGGTATCAGTTCGTAAATTTGA |
| comp106793_c2_seq1:1661-1662 | comp106793 | 1661 | T | A | 226 | 0.738938 | 0.261062 | 276 | 0.108696 | 0.891304 | AGTATACTGTGCAATTGGCAAATAACTCTTTCTGCTTTCTCTTTCCAAAAAATTCTTTAAACTATGGCTGTAAACATTGGCTCTTTAGTACACAGTCCTAT[T/A]ATCTAAATACCAATAAACAAGGCACGCGTCTAGAGTAGCATTTGATTTTGCGAAACATGAGGATACAAGGAAAACACAGCTGCTCTTTCAGTAAGTGCTAC |
| comp101613_c0_seq20:516-517 | comp101613 | 516 | T | G | 203 | 0.881773 | 0.118227 | 163 | 0.251534 | 0.748466 | AACCGGCCGCGGGGGGGGCAGAAAGTTGCAGATCTTCTCGATGTAGTCTCTGTCGCTGTACTGGATGTGCGTGCGGAGCGCGACGATCTCGTAGAGTTCCT[T/G]TTTCAGCGCCCTGGGAAGCGTTTCTGTGACCAAAGCCATAATCCTGAATCCCTCTCGGTCCATTTCCTCGTAGCGCCCGCGACGAATCTCTTGGAGAGAAC |
| comp106632_c1_seq1:315-316 | comp106632 | 315 | T | A | 127 | 0.141732 | 0.858268 | 57 | 0.77193 | 0.22807 | ATGTCATATAAAATTGCCCGCTAGAAATGAGCGAAACGCATTAGAAATCACACGGTTTGCGTCGTCAAACGTTTTTGTCTGAACAAAGAAAATGGAAATCC[T/A]AAGCACCAACACGGCAGAAAGAGAACTTTTGGAAAATAAACAAAAAAAGCAAACGTTTTAGGGTTCATTTTAATTTCATTTGTTGCAGTGGATATTCTCTT |
| comp105123_c0_seq17:555-556 | comp105123 | 555 | G | C | 337 | 0.792285 | 0.207715 | 37 | 0.162162 | 0.837838 | CCCTCCACAAGAGGTCGGCCGAACCAGAAGCTGACGCCTCCGTTGTTCACCCGCACACTTACTCCTACACCCACGCCGCTCCTGTCGTCTCCTACGGCTAT[G/C]GCTACCGCAGGGGCTATGGATATTAACGGAAAGCACTTGGTGACGTAACCGCTATCAGTATGGATCTAGAGTTCGTTCTTGCATCACTTACCAGGATGCAA |
| comp104452_c0_seq3:722-723 | comp104452 | 722 | A | G | 50 | 0.74 | 0.26 | 91 | 0.10989 | 0.89011 | ATGCAGCTGACGGAGAGCTTCCTGAAGGAGAGAGGTTTCCTCACCGGGAATCTGAGGGACAAACTGGACGAGCTGTGGTTCACCTTGTACAAGAGAAAGAA[A/G]ATCCTCAGCTCCTCGGGGTTCGAGCACGTCTTCGCCGGGGAAGTGAGAGGACAGAAGGTCTTGGGTTTGCACAACTGGGTGTACTTCTTTCACGAGGAACA |
| comp101940_c0_seq2:938-939 | comp101940 | 938 | T | C | 364 | 0.901099 | 0.0989011 | 616 | 0.271104 | 0.728896 | GTATGGTACATGGATGATGAGCAAAGGCTGAGAATAGGAGAATATGCAATCGACGTACCCCTGACAGCCCCAAGAACAAGAGTTGGGTTGTTCAAAGCAAG[T/C]GACGCCATTCATCAAAAATGGTGGAGGTTCTCTGAGTTAAAAGCTTCACTCGAGGGAGTAGAACCACCTGTGTATCCCTTCGATGGCAGCGACGAAACTAC |
| comp107236_c1_seq7:1904-1905 | comp107236 | 1904 | T | G | 136 | 0.852941 | 0.147059 | 148 | 0.222973 | 0.777027 | AATTGGGACTGTAGACTCACTGCCATCATCAACATCACTGTCTGCATCAAGGCCACGGTGAAGGGTTCTTGTGGTTGTGTAAGTTGAAGACGTTATTGTTC[T/G]GCTCATTGCTGTGCCATTTGACTTTGGCATGACATCAATACTTCGACTCCGACTGGATCTTCTCCTGGGTATGATAAGCTGTTCCTGCGTATCTTCAGCAT |
| comp104765_c0_seq1:4861-4862 | comp104765 | 4861 | A | G | 126 | 0.849206 | 0.150794 | 82 | 0.219512 | 0.780488 | ACGCCCTGTTCGTGTTTATGGTCACGTGGGGCAACGACACGAACACGAGGAAACGGGCTAACAAAGGGGGAGCCTTCCCGAGACGTCTTACCATCGCCTCA[A/G]CGGAAGAAAAGGAAAAGCTTTGGAAACTTAGGAACTAGAGGCCCTAAAGCTAAAACTAAAGGAGCAACCTCTCCTACATTACCTTGCGACCGCCGAAACCA |
| comp105186_c1_seq5:704-705 | comp105186 | 704 | G | A | 350 | 0.76 | 0.24 | 276 | 0.130435 | 0.869565 | TTCCGCTTGCAGCAGGACAGGACATCCGAGGTCAGCAGACAGCGACGAATCCCCAAAGCAAGCAGCAGGCAGGAGGAGCAGGTCCAAAGCAGCGGCAGACA[G/A]CGATAGGGCCCAGAGGGATTTACCCAAGTACGAAAATCTGAAAGCAACGGACAGAAATGACTCTTCACAAGCAGGAAGCAGGGAAGAGTTTCCAGAGAAAG |
| comp103613_c0_seq10:1777-1778 | comp103613 | 1777 | G | A | 145 | 0.77931 | 0.22069 | 207 | 0.149758 | 0.850242 | GACGGCTCCCGACGCGGCCACCTCCCCCCCTGGCTCGAGGAAGTTCCCCCGCGAACCTCATATTCGTAGCTCGACGCCCTGTACTGCCAGTCCCTCTCCTC[G/A]GACTCCTGTGTCCCGCCGTACTTCTCCAGCCAGTCGCGATAATGGTACCACCACTGCTCCCACTTCAGGTAGTCGTCCAGCGTGATCGATCTGTCGCCGAA |
| comp99001_c0_seq2:934-935 | comp99001 | 934 | T | A | 101 | 0.821782 | 0.178218 | 52 | 0.192308 | 0.807692 | TACAGTGACAGAAAGAAAGAAGGAAAAATCACAGATTGTGACGATTACATTCCATGGATGATGATGATGATGATGTTATTGTGATGATTGTGACGAGGTAT[T/A]TCTTCAGTAGATGTGATGTTACGTCACTCACTATAAACAATGGGGAGAATCCTTAGAAATCCTCAGATTCTCACGATGGACATGAACAACTGAACAACAGA |
| comp97732_c0_seq1:210-211 | comp97732 | 210 | A | G | 98 | 0.785714 | 0.214286 | 96 | 0.15625 | 0.84375 | GTACATGACACACTGGTGAAATTATAACGATCACTACCAATCCTGCAGTGATAAGCGTTATCTTTGAACTGATGATAACGCTGCAATATTCGTGGCTTCAT[A/G]TGAAAGTTCCCTTCGTTTAAAACTCAAAATATATTCATATACATCAGAGTATATGGTATATATATGTGTGTGTATTTTTGTGTATATATGTTTGTTAGTTT |
| comp104258_c0_seq43:1217-1218 | comp104258 | 1217 | T | C | 276 | 0.648551 | 0.351449 | 157 | 0.0191083 | 0.980892 | TGGGACATGGCAGCTGGTACCCTGATACTTACAGAAGCTGGGGGTTATGTGTGCGACACGGAAGGCGGTCCATTTGACATCATGAGGAGGAGAGTGCTGTG[T/C]GCTTCTTCACCAGAACTGGCCGAAAAGATTTCCAAGTTGCTGAAGCAGTACCAGCCCGAAAGAGATGGCGTGACTAAGTAATTGAATTTTTCGACGGTGAA |
| comp102779_c0_seq2:1223-1224 | comp102779 | 1223 | C | A | 85 | 0.776471 | 0.223529 | 68 | 0.147059 | 0.852941 | TTAGCAGTGTGTATAATGCAGCACTCTCCCTCAAAAGAAGTACTACATTTTAATGACATTATGAAGTTAGCTTACCTTTCAGGGTCTTGCATAGAAGAGAA[C/A]AATGTAGAAATATGATATTTATAATGTGTAGTGGTGCTTAAACATGATTTAAAAGATGTTCGTAACAAAGGTATCATTTCAACAGTAAGATAACTTTATCT |
| comp104446_c0_seq1:413-414 | comp104446 | 413 | C | T | 164 | 0.682927 | 0.317073 | 149 | 0.0536913 | 0.946309 | GAGACTCTGTCTCGGGATACTGTAACTCACGTTGAGGTTGATGAGACTCAATAAAGCCTAAACTTGTTATGCAGGCAAGAAATCCGTCCATGCATTTTCTG[C/T]GTGTATTTTGTAAGTCTTGTACTTTTCTGGAATGTGTTCCGTCCATCCAAACACTTATTGATCACTGTACAAACTTGATATTCCAGTGTCATATCAAGAAA |
| comp105750_c1_seq7:1596-1597 | comp105750 | 1596 | A | C | 200 | 0.775 | 0.225 | 96 | 0.145833 | 0.854167 | GTCTCGAACTGCGAAGAACGAGCTGTCCTTTGTCTGGAGGTTTCCGGGCTTGCGGGCCTCAGCAAGCTTCTGACCGGGGTTCCAGATGAGTTTCCGGAAGA[A/C]GCCCGGTCTCCTTCGCAGGCACCGGGGATTTTCGGCCCGGGAGAAGCCCTGCTGAAGCCTACCGGGCCCTCTGAGGAGACCTTTCCCGAACCACCTTCGGG |
| comp106586_c0_seq1:1676-1677 | comp106586 | 1676 | A | G | 324 | 0.768519 | 0.231481 | 208 | 0.139423 | 0.860577 | GGCTTCATTGCAATGCGCATTCCCTTTCCTCCTCCTCCTCGGACAGCCTTGATCATAACAGGGAAGCCAATCTTGTCTGCTTCTTGTCTCAAACGTGCATC[A/G]CTCTGATCCTCTCCATGGTAACCCCCTATTACAGGCACACCGGCTTCTGACATTATGATCTTTGATGTACTCTTAATACCCATATCTCTGATGGCCTGAGC |
| comp106840_c1_seq12:352-353 | comp106840 | 352 | G | T | 93 | 0.795699 | 0.204301 | 12 | 0.166667 | 0.833333 | GATTGGCAAGAAGCTGCATGTTTGGGCTTCAGATTCGCGAGATTGAGAAACAAGGATGATAAAGAGAGAGAGAAAAAAACATTTAGATATTCGAGGTTGAT[G/T]GAGAGATGGCGAAGACGTCGATGGCCTGAAGTCACTTAAGTGTTGGTTATATTTTATTTTATTCTGACAAGGAATGGACGCGATGTGGAGTGGAGCCTATA |
| comp94407_c1_seq3:1565-1566 | comp94407 | 1565 | C | T | 71 | 0.802817 | 0.197183 | 23 | 0.173913 | 0.826087 | GAGAGGACCCCGCGCGCGAAGAAGTCCGGGTGGAGAGTGACTTCCTCAACGGCGAACCTCTGCACGGGGGGCGCGCAGGCGTGTCTTCTTCTGCTGCCGTT[C/T]GTGCAATCGGGGTCCGTCGAGGTGTCCCATTCCCCGACCAGAACCTCGACTAACTCTAGTCCTCTGTCCACTGTCAAGTTTTTAAAGGCACATTGAGCAGG |
| comp106050_c2_seq1:1356-1357 | comp106050 | 1356 | A | G | 165 | 0.8 | 0.2 | 187 | 0.171123 | 0.828877 | TTCATCATATGGCAGTTCGCATGCAATCTTCAAATCTTCGTCACAGAACAGAGCAGATGAACAAAATCGTATGAAATCACCGAAGCTTTTAAATAAACCTT[A/G]ATTAAGAAGAGAGAATACGCACATGGCAGTGATAAATCGGAGGAGTGTGTAGCCCCTTTTTGTAACATGATTCTAAATGCTTATGTTTAAGCTTTTATGAT |
| comp104186_c0_seq3:685-686 | comp104186 | 685 | A | G | 33 | 0.878788 | 0.121212 | 92 | 0.25 | 0.75 | TCCCTTTAAACCGCCTCTCATTTTGTCTCGAGATTCGTGTAACCCGTTTCGAAGCGATTCGAGGCGTCAACCGTGCGAAAGGGAGTTATCACCGTGACTGC[A/G]CACATCACGCAATGCAGACTCGGTGAAATGTGCCACCTGACGCGTTTGTCAAGGTATGCGCTGCATCCACGACAGGTTTAGTTTGTAGATTCGTAAGGTGC |
| comp105392_c0_seq1:2212-2213 | comp105392 | 2212 | T | C | 51 | 0.941176 | 0.0588235 | 16 | 0.3125 | 0.6875 | AGGACTGGTACACGGCCACGTACGCCCTGGGTAGCGTGGCCTTGGTGCTTCTGCTGCCGTGCCTCCTGCTGCCGCCCGTCGAGGCCAACGTCCATCAGAAC[T/C]TGGCATGGCGACGAGTGCTAAAATCCCGCGCAGTATGGGCGTGTTTGGGCGTGCACGTTGCCAACACGTGGGTCATGCATTCCCTACTAGTGGGCGTCCCC |
| comp105748_c0_seq2:2619-2620 | comp105748 | 2619 | G | C | 128 | 0.90625 | 0.09375 | 18 | 0.277778 | 0.722222 | AACAGTCGTCCTAATTGCCTGATGGTATATATCCCCAGCAGAGTAGCCATAGTCCTTGCTAAAGTGGACTAAGAAAGGGAGGCTAAATCAGCCAACTGAAA[G/C]TGTGTTGTAACAGTATATATGATATTGTGTGATAGAAGATTAAAAGAACTGGAAATAACCTGAGGGTGTTTGTTGATATTTCATTTTGATTTTTTATATAT |
| comp104542_c0_seq1:3507-3508 | comp104542 | 3507 | T | C | 55 | 0.872727 | 0.127273 | 45 | 0.244444 | 0.755556 | GAAAGTTGTTAGTAATCCTATAGTAAATATTTTTCTTAGGGAGAGTGAAAGAAAACCTGTATGGAAATTAGTTTTATTTAACAGTCGATTCCACTGTCTCT[T/C]TTGAGGCTGTTCTGTGCATTTAGAGAAACGAAATGAAAAAATTTGCATGTTATCAACTGGTCTTTCTCTGTAGTTCCCTTTATTGTACCACAGTCTTAAAA |
| comp101869_c0_seq1:1811-1812 | comp101869 | 1811 | A | G | 72 | 0.819444 | 0.180556 | 47 | 0.191489 | 0.808511 | TCTTCCGAAGCTTCGTACGTGGCGCGATCTCCGAAGTAGTTGCTGTCTGACCACTCTCGGGGCGTCCGTGATCTGGCGTCGTAGCTGTACATCGGCTTCTT[A/G]CTGTTGTTCTTAAACCAGAGAGTGAGAGTCACTTGGCTTGCAGGCGCTGGTTCTGACACGTTGCAATATATCCTTGTGCCTTTTCCTATCACGCCCACTGT |
| comp103474_c0_seq2:874-875 | comp103474 | 874 | T | C | 941 | 0.768332 | 0.231668 | 598 | 0.140468 | 0.859532 | AGATAAGGCAGAAAGCGGCGGCATTCTGTAAGGATCGTGGTCATGAAAGTGCCATACTTGTAGCCATAGAAGTCCAGCTCGTCCTGTGTACATGGTCGATA[T/C]TCCTCCAATATCATCTTCAGGAATGGATTGAATACGATGTTGGAGTACTGGGAAGAGAAGTGGAAGCCCGAGACCGTCTTGACGCCTGTCACCTCTGGTTC |
| comp103863_c3_seq19:996-997 | comp103863 | 996 | T | C | 373 | 0.745308 | 0.254692 | 170 | 0.117647 | 0.882353 | CAGGTCGTGACGGGTCAGCTGGTCTACCTCACCCTAAGAGTAGGAGAGACAATCTGCCCCATTGGAACTCCCAGTCTCAGTGCCTGCGCATTCGACCCCTC[T/C]GAAGACGCCCATATCTGTGAGATCGTGGTTTGGGAACGCCCATGGCTCAACAGTACAAAGGTCATCGACGAGAAGTCCAGGTGTGCTGAAACAGAAGATGA |
| comp82183_c0_seq1:131-132 | comp82183 | 131 | C | T | 223 | 0.852018 | 0.147982 | 147 | 0.22449 | 0.77551 | CATCAATCAGGTTTTCCTATCATGTCTTGCGTGGCATCAGCAAAGCGCCTCAGAGCATTCGAAGAGTAAAACCGGCCCCAGCTCGCAGTCGATCGCTCCTC[C/T]GTGCAGTCGTGAAGCATGTCTATTTTCCAGGATGAGGTGCGCGATCTTCAGAACGGGCGTGACCAAAAATTTCATAACAAATGCATTCACCATTCTCATAG |
| comp95594_c0_seq1:723-724 | comp95594 | 723 | A | T | 1497 | 0.778223 | 0.221777 | 411 | 0.150852 | 0.849148 | CTCATGCCTTTTTAAATATATTTATCGGTACTTGGATTTATTGCTAGTCACAATAAGATGCCAATGACAAGAATCTATGACCCTATGCATGAATCAGGAAC[A/T]CTGGGTCATTTGTTGAACGTGTGTATATAACCTTTTGAAGACCAACTTTATTGTGATTACCAATAAACGAATTTCAAGGGAGTTTCTGCACAGCATATCCA |
| comp96805_c1_seq1:405-406 | comp96805 | 405 | C | T | 75 | 0.626667 | 0.373333 | 2 | 0 | 0 | CTAACAATTTTTGGCGTGAGCTTTATGATGGAGGATGGCGTGCGGCGCGGAATGGTGTGGATGATTGGCTCCAGACTGTATGACGGGTCATCACTTTGGAC[C/T]GTGACGTGAGAGGCTGATATAGTCTGGATGGTCTGGCCACGGGTTGAACTTCTAGCAGGTTGGATGATAAGTATGGGAGGGTTGTCCGAATCTGAGGATAT |
| comp107268_c3_seq2:251-252 | comp107268 | 251 | A | G | 119 | 0.638655 | 0.361345 | 83 | 0.0120482 | 0.987952 | GACTTTGCGCCTCTCGTGTCGCCCCAGCTGAGCCCAAACCCAGAGAACCTCGACTTGGAAGAGCAGGACTACAATCTCTTGTACCAGTTCCGGTTACTGAC[A/G]GCTCTAGAAATAATTGAAGAAGTGAAGAAGCTCCAAAATGTGGCTTACCAGCTTGGCTTAGAAGAGGCAAAGGAAATGACGCGTGGAAAATACCTTCACAT |
| comp95106_c0_seq1:666-667 | comp95106 | 666 | A | G | 87 | 0.724138 | 0.275862 | 41 | 0.097561 | 0.902439 | TTACGCTACAGGTAAATTGTACCTCTTGAAGTTCTGGTGCAAATATCACGCGTAAAGTAGTTGCCAGATACACGTGCGTTATGGAGTAATACGTTCGCTGG[A/G]TTTGCTATATGGTTTGTAGCACCAGATAAAACGATGTGGTCTTGGCACGGCTAAATGCGTCAGGTGGATGGAGTTGATAGGCACGGAGTTCTGCTAAAACC |
| comp107934_c0_seq3:4936-4937 | comp107934 | 4936 | T | G | 94 | 0.925532 | 0.0744681 | 97 | 0.298969 | 0.701031 | AATCAGAAAACTGAAGTAGTAAGGGAAAGGGATATGACTTTTTTACATATTCTCTCAAACCACTTCTGCATAGCATAATCAACCAAACCCCACAAAAAGGG[T/G]TTTCATTTAAAGAACACTAAAATTATGCTTGTATGATAAAGTAGTTGACTGGGTTCATCTACTTTGGAAAAATAATGTGCTTTAAAATATTTCCTGAAGCC |
| comp107797_c0_seq2:1087-1088 | comp107797 | 1087 | T | C | 63 | 0.0793651 | 0.920635 | 17 | 0.705882 | 0.294118 | GAGATGAGTGCGAGTACATATCGTGGAAGGAAGCGATGGACCCGACCTCGCGCCTCGGCCAAGGCGTGCCCTGGGAGTTCGTGTTCAGCAGATGCCAAGAC[T/C]GGACTTCCCACTCCTCAGACCCGCCCCCTCGAGCGGAATGGGGTGCCCGGATGCCACTCCCCTCGCAACGCCTCCCAGACACTACATTTACTCGTAAAGTG |
| comp107403_c0_seq1:2089-2090 | comp107403 | 2089 | A | G | 102 | 0.715686 | 0.284314 | 190 | 0.0894737 | 0.910526 | AAAACAATGGAGTCTTGTGAAGGTAACTTGAATCTGGTTGACCTGGCTGGATCAGAGAGACTGAAGGAGTCAGGCTCAGAAGGAGCAAGACTAACAGAAAC[A/G]CAGAACATCAACAGATCTCTGTCCAATCTTGGCAATGTGATTATGGCTCTGGGTCAGAAGCAAAGTCACATCCCCTATCGCAACTCCAAACTGACCCACCT |
| comp105827_c0_seq9:965-966 | comp105827 | 965 | C | G | 132 | 0.689394 | 0.310606 | 79 | 0.0632911 | 0.936709 | ACCGCCAAATCATCTGCATTCTGCCACTTACAGGACCAGTCCCCATCACTGTTCCTCGCACCTCATCTGCCATGTGACTTCGTGCTGGACTCTGCGAGGTG[C/G]TACTTGGAAGAGATACAGAGAGCAGAGTGCACACCAGGTCGCAAGGTGGAACGCCCTTGTGATATTTCATCCGATTACGCCAGTGGCCCATGCTCCTGCCC |
| comp98658_c1_seq20:176-177 | comp98658 | 176 | T | C | 541 | 0.922366 | 0.077634 | 388 | 0.296392 | 0.703608 | GATTCAAGTTTGGGCTTTTACAAAGAGCTCATGGTGACACTGCAAAATCCGAAAAGTGAGTCTTTCCTCTGCTGGAATAGTTGGGTCCGTCATAAAATTTG[T/C]ACGTCCAAAATTAATCATTTTCTCTACCATGACTACTCAAGGAAGTTGATGTCACTCCGGCCGAAGTAGCCGTTGGAGGTCCTGTAGCCTTCGCCGCCTGC |
| comp104768_c2_seq1:2239-2240 | comp104768 | 2239 | T | C | 154 | 0.668831 | 0.331169 | 70 | 0.0428571 | 0.957143 | CCAAACCGGACGCCATTTGGGTTGAGTGTTCCATCACTGCGTCCCAGCATTGTTATTCCGCCCGTCTTGGAATTGATGGAAACAAAGTCGCCGTGAGTCCA[T/C]GTGCGGGGGTACTTTGCAAAGTAAGCCTTGGTGTACTTGATCTCGCCATCATCATTCCAGAAGTGTGTGGGCATGGAGGGGAAAGGCTTTGTGCACACCAG |
| comp103391_c0_seq1:433-434 | comp103391 | 433 | T | A | 64 | 0.734375 | 0.265625 | 83 | 0.108434 | 0.891566 | ATCTTTTTTTTTCAGTAAGAAAATACTTGTTTTTTCTTTTGTCAGTTTTTTCTTTTGTCAATGTACTAATCTTGCACTTGGGTTACATCAGAATTATCTTT[T/A]AAAAATTTCTGTACCTACATACCACACATCTAGCAGATAAGATCACAAATCTCTAGAAAGACCACCAACTTCACTATGCTTCTTGTTGAACGTGGTGGTGA |
| comp102981_c1_seq11:1459-1460 | comp102981 | 1459 | T | C | 5364 | 0.9327 | 0.0673005 | 3506 | 0.306902 | 0.693098 | AGTGAGTCCCTTGACGGTGGCGACGAGGAACTCCTCCCGTTCTTCCGTCAACCAGGCCTTAGCAGGGCAGACTTTTGATCCGCAAGGACTCTCAGGCATCT[T/C]TCGAAGTTTCTCTGTGTCACTAATCAGAACAGCCGGAAGGGGCATAGCTGTTCCGTTGACCTGGAATGACCAGAATTCAACGGTCTCCTGGGTGGTCAGTT |
| comp102786_c0_seq1:121-122 | comp102786 | 121 | G | T | 162 | 0.901235 | 0.0987654 | 145 | 0.275862 | 0.724138 | TCTGTACGTTGGAAAGCTTAAGAATAACCTTTGATATCAAAGACATTGAACATGAGTATTGAATATTCATAAAAACACATCCTGATATTTTGGCCGAAGCT[G/T]TACCTGCATCATGAAGTTGAAAACGACTGAACGACAAACAATAGGATGATGCTTACTGGTCGCATCTAAGCACCGTTAACTTGCCACACGTATCAAAGGGA |
| comp87637_c1_seq1:721-722 | comp87637 | 721 | T | A | 27 | 0.740741 | 0.259259 | 52 | 0.115385 | 0.884615 | ATACCTTGAAAGGTGTTCATTGTTCACATAGCGGTTTACTGTTTTGATAAGTTCATGAAGCTTACTGAGAGTGGGGCGCCTTTAGTAATGATGAAGAAAAG[T/A]CAAGGATAAGAGAGAGATAAAAGAGAACAAAGAAAGAGGCTGATAGATAGATGGAGGGAGAAAGTTATCAGTCATATAAACACAGCAAAAGACAGGTATGT |
| comp105993_c0_seq9:539-540 | comp105993 | 539 | T | C | 56 | 0.767857 | 0.232143 | 21 | 0.142857 | 0.857143 | TATAGTATGGAATGCATACGTTTTATTTTAATATACACTGTACATGTATATATGTCTTTGATATGCAATTCACAATTAAAACGTTTCAAAAGTTTGTTTTC[T/C]CACAAATTTACCATTCCATACGAAGTGTTATTTTTTCTAAATGTAATTTCTTAAGAATATAGATCTTACAAGAACTAATCAGTCCTTCCCGTTTTCTTCGA |
| comp107098_c0_seq1:756-757 | comp107098 | 756 | T | A | 52 | 0.75 | 0.25 | 8 | 0.125 | 0.875 | TATTCCTTTTCCACAGCAAACTTCACTTTTTCATTTGAACTTTTGAATTCCTGTTTATGCCGTAAAATCAGAATATAAATTAAGAAATTGTCACCAAAAAA[T/A]AAAATTACATATATATGAATAAATATCTTAAGAAAGATCTTTTCATACCTAATCATTAATACCTAGTAAAGGCATATGTATTGTGTAATGCATGTACATTC |
| comp107743_c1_seq1:2021-2022 | comp107743 | 2021 | T | G | 3874 | 0.90475 | 0.0952504 | 2994 | 0.279893 | 0.720107 | GCTCGCAGACCATCAGCGGACCTCTGTTTTACGAAGTAGAGACGACCATTACGGGCCTGGCCGCCTGCACGCCCTACGACCTCTCGGTAACAGCCGTGTCC[T/G]CTGTGGGCGAGAGTCTGGCGACGACGCTGACCGCCTCCACGCTCTGTTAACTCTAGTTTTAAGTGAGGGTTTAGGGCTTCTGGATTCGTGTTAGATCCTCA |
| comp99498_c0_seq5:136-137 | comp99498 | 136 | C | T | 65 | 0.753846 | 0.246154 | 31 | 0.129032 | 0.870968 | TGTGTACTGTTGATGAATCTAGCATTTACAACCTTACTTTTCACATGATTTCAACTTATAAATGAAAAAGTGTTGAAAGAACTACTTAAGATTGTGAAATT[C/T]CAGATGATTACTGTATGAAAAGTTATGGCAAACTGTTTTTTAATTGGTTTCTGATCTTTTATTCATTTTTTTATTTCCCTTTAGAACTATCATGTAAATTA |
| comp100645_c0_seq4:373-374 | comp100645 | 373 | A | G | 53 | 0.660377 | 0.339623 | 28 | 0.0357143 | 0.964286 | CGCCCATCCTGTCTTGGTGACTCTTTACCTTGTGGGAACTGTCCTGTGCGTGGTCATGGCTTACTTTCTCGACACGTCCCGCAGGTCAACCAAGGGTCAGA[A/G]ACAAGCCACGCCCAGCGATAACCTTAATCCAGACTCCAGTGCTATAAAGGAGCTGATCCGCGAGCCAGGCGAGTGGGTTCGTCCCCTCGGCAGCCTGGAGC |
| comp104557_c0_seq1:877-878 | comp104557 | 877 | A | G | 75 | 0.92 | 0.08 | 115 | 0.295652 | 0.704348 | CTTTCAAAAAATACACTCACACATGAAAAAGTACATTATTTTTAAAGGATATTAAAGACGAAACAATTACAGACAGACGCAACGTACTTGCAGAGTCCTAT[A/G]CACGCAGAACATAAAATACATAATAAGTTTTCCTTTTGAAAACGCCTCTCGATCCTCTTTCGTTCTCACTTGAACACAAAATGGCCGAGTTTTATCTACCT |
| comp105979_c1_seq4:3256-3257 | comp105979 | 3256 | G | C | 74 | 0.824324 | 0.175676 | 5 | 0.2 | 0.8 | CATCGTTCAAGACTTTGCTGTGTTTTCTTTCCAGTTTTCTCGCTCGCTTTTCAATCATGGTGCCTCTCTTGCAGTTGCGATGGCGGAAGGGGCGGGAAAGC[G/C]AAGATGAATGATAGATAGATGCGGAGAAAAGAGGGACGAGTGGTGTTCAGTCGTTCTTTCTCTGTCTTCTTTTCTCTTTCTATTTGTCCATCTCCCTTCTG |
| comp107402_c0_seq2:2654-2655 | comp107402 | 2654 | A | G | 362 | 0.917127 | 0.0828729 | 362 | 0.292818 | 0.707182 | GGCCTTGATCCACTCCTGCTGGTTAATAAAGTCCACCGCCTTGGCACTCTCCTGCTTGAAGTAGATGAGGTCCAAGTCATCGATGAGGCCGACCTGGTACA[A/G]GAAGTCCCCGTAATCGACCATTGACACAGGGTCGCAGAGGCCGTCGCCAATAGCCATACCTTTCAAGTTAATTTTGATTTTTGCCGTTGGGTTCTCCTTAT |
| comp99181_c1_seq4:457-458 | comp99181 | 457 | A | T | 7 | 0.285714 | 0.714286 | 100 | 0.91 | 0.09 | GTCAATTAGTAGGCCCAGGGGACATCAACTGATTTTCCTATTCCTTGAAAAAAGAAAAATAAGAAAGAAAGAAAATCTAATGGTATCAAATATCAACATTT[A/T]TAAGCATTATTGACATCATGATTATCATATTATTATTAAATTGCTTACATTAAAAAAGAAAAAGAAGAAAGGAAGAAAAAAAGAATAATCATGGAAGAGGC |
| comp97514_c0_seq1:1051-1052 | comp97514 | 1051 | G | A | 104 | 0.826923 | 0.173077 | 74 | 0.202703 | 0.797297 | CATAATGAGTAATCGATAATGAGCAAGAAGAAGAAAAACCTGTGCTACTGTCTGTTCATTCTATATCAGACATCACACGTAAAATACCTCATGCCAAGTCA[G/A]TCTAATCTATTTATCACTTTGACAGAATTCCAAGAGAATCGTAAGCATTGTACTTTGGTTAGCAAATAGATATAAACATATGTGACATTCATAATTTATCT |
| comp99636_c0_seq1:558-559 | comp99636 | 558 | G | A | 65 | 0.153846 | 0.846154 | 18 | 0.777778 | 0.222222 | CTGCCTGAACTGCAACGTTAGAATATGTTCCGATTGTTTACGAAAAGAAGGGGCCACGTGCAGGAGGACATGCCAGTGGAACGAGTACCCGATCTCAGTAC[G/A]GTGTCACAGTCCATATTGTCAGTGCTGCAGAAAGTGTGAAATGGAATCGTCTTGCCAAGCGTCCGGGGGCCGCTGCGTGGGTCACCCTTCTTTCTGCAGAA |
| comp102020_c1_seq3:1685-1686 | comp102020 | 1685 | A | C | 79 | 0.696203 | 0.303797 | 55 | 0.0727273 | 0.927273 | TTGGGCGGGTCGGAGGCGTTGAGAGCCGGGCCCAGGGACACCTCCGATAGCCCCGACGACCCTGCCGCGACTTCCGTCTCCCAGAAGAGATCGGGCGAGTA[A/C]CCGTGCCAGTCCTCGCCCTCGAAGGGGTCCACCACGTGAGCTTGAGGGAAAGAAGCGGTGAGGCTGTCATTCACCGAAGCCTGCTTGTTCCTCTTTCTCTC |
| comp97433_c1_seq3:2127-2128 | comp97433 | 2127 | C | T | 60 | 0.9 | 0.1 | 47 | 0.276596 | 0.723404 | CTTCTGTTGTCAGGGGTGAGGGGGAGCATGCGGCGCTTGCACTCTGCCGTTGACCACCGTTGCAATGCCTGGAACACCTTCAGTTCATTGGGTACGTTCAG[C/T]GTGGATCTCTTCAGGATGAGCTGAAGGGTGGTGGAGTCGAGTTCCTCTAAGAATTCTGTAGCGAGGACAGACTGGGTTCCTCTGTCAACTACGTCTAGACA |
| comp106274_c0_seq5:718-719 | comp106274 | 718 | T | C | 63 | 0.714286 | 0.285714 | 22 | 0.0909091 | 0.909091 | GGGCGGCTGAAGCAAGGTCGCTCGTCCCTCATTTTCCTCATCTTTATCTTTTGGTTGCTCTATTTCCCTTTCGTGATATCCATTTTCCTCTTTTGTACTAT[T/C]GGCCTTCTTCTCTGAGTCTGCTGTGTCCGTAGCTTTATCCTTGTTTTTCTCATTCTCCTCATTCGCTTTCTTCGTTTTCTGATATTCTCTTGCTTCACTCT |
| comp104224_c3_seq24:1380-1381 | comp104224 | 1380 | G | A | 178 | 0.910112 | 0.0898876 | 129 | 0.286822 | 0.713178 | TTGTAGAAACTGTAAATTCTTTTGCATTCTGTCCACCATTTTTGAGTATATCTTTGAAGTCGCGCATCTTGTCAAGAGTAATAAGGGAAACAGCTGTTCCT[G/A]GTTTTCCAGCTCTTGCAGTTCTTCCAATACGATGAATATATGTAGCACTAGATGTGACGTCATAGCTGACTACATTGTCAACATTAGGTATATCCAGTCCA |
| comp105716_c0_seq1:432-433 | comp105716 | 432 | G | A | 26 | 0.961538 | 0.0384615 | 65 | 0.338462 | 0.661538 | TGACATAAAGCAGTGCCGTACTTGGCGAGGTCAACGAGCAACGAAACTTCCTCTTATTGACCTTTTAAAGGACGTCACACGATAGTACATTATCCAGTTAG[G/A]TTGTACCTTATTACATCATCCAGCAGAGTATAATACTTTTTTTAAAATATACTCGAAATTCGTGTGACTCTCGTGACCGCATTTCTTTCGTGAGCAGCTGA |
| comp104234_c1_seq3:3696-3697 | comp104234 | 3696 | G | A | 47 | 0.787234 | 0.212766 | 67 | 0.164179 | 0.835821 | TTCTCCTTGTCCAAGGGGGTGTTATCGCCAAAGATCAGGTTAGGGTGGTCTAAAGTCTGGTCCATGGCAGATGCATCCCTCATGATATCTGGGGGTTCCTC[G/A]AAGCCCATGTCTCCAAAGCCATCATCACCTGTCACCAAGGAGATGTTGCCATAGTCTTCCCTCATTGTGATCTCTTCAGCACGTGACTGGTTGAGAGTGAA |
| comp104987_c1_seq1:700-701 | comp104987 | 700 | C | A | 87 | 0.758621 | 0.241379 | 118 | 0.135593 | 0.864407 | AGCTAAATACACATACCCTATGAACACCTGCACTTTCCTTCCCTATCAAAATGTTTGCTGCAAATTGCACGTCAGTGACCATCATTGTCTGAATATTCACA[C/A]AAAAGTTGACAACACATTCAAATTTCTGATTAAAAAAAAGTGAAGAGAGGTAACATCCCAAACAGAGAAATTGAAACTTATTTCTTTATAATAACTTTCAA |
| comp105040_c2_seq6:1754-1755 | comp105040 | 1754 | A | G | 339 | 0.879056 | 0.120944 | 566 | 0.256184 | 0.743816 | TTTTTGGCTGCTACAGGAGGAAGCGCGCAGGGTAGGAGGAGTTCAGACGGCGAAGGAGGAACCGAGAGGTCCCTAGAGTTCGGGGGATGGCGGCGTTCGTG[A/G]TGGCTCTGGAGAAGCAGTTTCGCAATCAGGATATCGCTTGCGTTGTCAAGTGTTGATTCTCACACAGGTGTTTGTGATTAGAATGTATATAAAACGTTCTG |
| comp104966_c0_seq6:382-383 | comp104966 | 382 | C | G | 52 | 0.846154 | 0.153846 | 197 | 0.22335 | 0.77665 | TCCTTCACACTGACTTCAGCTGATCTCATACTTGAAGTCCCAACTCCAGCTGATCTCATACTTGAAGGACGAAGTACCAATCGATCAGCACAGAAGACCAG[C/G]CACATATGAGCTCGTCCTTTTAACACCTTTTCAGGACCCTGGAGACACACTCGGCAGCAGAGATCATTCTAGAAGGTTATGATAGTCACACTCGCACCCTT |
| comp104232_c2_seq1:780-781 | comp104232 | 780 | T | C | 237 | 0.805907 | 0.194093 | 229 | 0.183406 | 0.816594 | ATCCCACGACGCTCGAGACCATCAGCAAGGTGGACCTTCACAAAGAGTTGGGCATCAACTCCCACTGCCCCAACCCAAAGCCCCTGCCCGACGGGTCCACC[T/C]TGAACATCCTGCACGCCGTGGGAGCTACGGGACCCAAATATGATATCGTCTCCTTCCCGTCCAAGCCTCTCGATGGCAAAGCCACTGTGTTTGCCAAGCCC |
| comp105219_c1_seq1:426-427 | comp105219 | 426 | G | A | 40 | 0.775 | 0.225 | 59 | 0.152542 | 0.847458 | TACATGTACATCTTGCAACGCTGCTCTCTAAACTTAAAGGGATCGTGTGGCTTATGAAAATTAGCAGTATCATGGGGAGAAGATTTACCATAGTTTAGAAA[G/A]AAAATGATAAGGATAATAATGAGTATATATAATAAAGTCAACACTGATATGATAAAATCAGAATGATTAATTTGCTTCTTTTGTAGTCATTCTCAGTGCTT |
| comp104982_c0_seq2:1329-1330 | comp104982 | 1329 | C | A | 71 | 0.859155 | 0.140845 | 76 | 0.236842 | 0.763158 | CGATCTGTTAACAATAAATGGGCATTTATAATTCTTTGTTATGAAAGTAAGACGGATCTGTCTTACTTAATATCATGAGATTTGTATGTCAAGTAAATTAT[C/A]TTTACATGTCAACAGATAAGAGCAAATCATTGTATGCAAAAGTCTTTAATTGATACACAGATTGTAATTGTTTTTCGTATGTATATAAATTGTCTTTATAC |
| comp100219_c0_seq1:963-964 | comp100219 | 963 | T | C | 1170 | 0.865812 | 0.134188 | 825 | 0.243636 | 0.756364 | TGCACGGTGTTGCTCACGAAGCCATTTACGGGGTCAGCAGAGTACGTGGTCACGTGACGGACGCCGCCGGCATCGACCCACGCGTACTGGCCCTGGACCCT[T/C]CCGTTCTCGTCCTGCGACTCGACTCTCTCCTGGTGGTTGCCGAGCTCGTCGTCGGCGACCAGCAAGGTGAATTCGTAGGGTTCGGGGGTGTCCTCGTCCTC |
| comp106683_c2_seq3:453-454 | comp106683 | 453 | T | A | 55 | 0.836364 | 0.163636 | 14 | 0.214286 | 0.785714 | TTATTCATTTTTATATTATCCTAATTATTAGATATTGAAATGGCTTGTAGGAAAATTATCACTTTGCTGTACAGAATTGAGCTTTTTGCAAAGGCACTACA[T/A]AAACGTCACTGAATTTTAACACAAGAATAAATTAAATTTTGTAAGGTTGAGATGAAAATGATTTTTAATTTTTATGCCTTATTTATTCTTATTCATGTGAA |
| comp100567_c0_seq1:1086-1087 | comp100567 | 1086 | G | A | 1870 | 0.704813 | 0.295187 | 1450 | 0.0827586 | 0.917241 | GCCCTCCGAACAATCATGATAGCATCATGTAAAGATCTTTCAGTCTCTTCCAAGAACTGTTCAGCACCACCTCGCAAAACAATAGTGCAGGTCTTTGCCTG[G/A]GGACATCCTTTGAACAAGTTGAACCTTTCTGCACCGATCTGCTTCTCCTCGAAGATCTCGCAGGTACCAAGAACTGATTCATTAAGGTCATGGGCTGTGGT |
| comp97493_c0_seq2:172-173 | comp97493 | 172 | G | A | 106 | 0.867925 | 0.132075 | 61 | 0.245902 | 0.754098 | TCTTACCAAGGAGAAGCCCAATACCCAGCCCAGGAGCCTTCCAGGTCCTACCAACCTGCTCCTGCTCCATCCTACCAGCCTGCCCCCTCCCCATCCTACCA[G/A]CCTGCTCCCACCCCTGCCTATGGTTAATATATTGTACTAATGTGCAAAAACGTAAAATTACTTTGAACGAAGATTGTGTTTGTAAATAAATATTGTATTGA |
| comp92227_c0_seq1:1294-1295 | comp92227 | 1294 | G | A | 167 | 0.826347 | 0.173653 | 44 | 0.204545 | 0.795455 | TCATCATAAAAACCATATATCCTGTTTATGGAGGCGCATTCATGGTTGCCACGAAGAATGAAGAAATTGTTGGGGTATTTGACCTTGTAGGCGAGAACGAG[G/A]CAGATGGTCTCCAGGGATTGCTTCCCTCTGTCCACGTAGTCGCCCAGGAAGAGGTAGTTCTGGTCGGGAGGATACCCCAGCTTGTCGAAGTGGCGCAGGAG |
| comp106439_c0_seq10:1035-1036 | comp106439 | 1035 | A | G | 81 | 0.246914 | 0.753086 | 99 | 0.868687 | 0.131313 | GGGCAGGTGAAGCTATGTGCCACAATGCCAAGTCCCGAGGCACCGGAATCCAGGCACCAGTAGTATTTGTGGCAGTTGTCAGGGTTGGGGTAGAAGCCTTC[A/G]CGCTTGCAGGAGAAGCCAGTGGATGGACTTGGTGTCGTGGGCGGGGCGGGCGTGTTGAGGGGGTCGATGGTGGTGGCTTGCTGTTGACGCCTGTTCTGTGG |
| comp106689_c0_seq1:404-405 | comp106689 | 404 | T | C | 73 | 0.931507 | 0.0684932 | 113 | 0.309735 | 0.690265 | AACTGCGTCAACACCGACGTTGTGGTCGGTTACACCACAGCGAGATCTGCGTCCCATGGCTAGGAGGAGTTGTGGCCGCGACAGAAATGCACTTGCTAGAA[T/C]AGTCTTTGGGTTATCTTAATTTCACATACGTAACAGCACGTTTTCTCATTTAAGTATGTTGTGTTTACCGGTGCATTGGCCTATGTATCTATCTCAGTGGT |
| comp107872_c0_seq19:1415-1416 | comp107872 | 1415 | A | G | 76 | 0.710526 | 0.289474 | 45 | 0.0888889 | 0.911111 | TGGCTTGTATAGGCATCGTCGGACTGACTTGGGTGGTCACTGTAGCCTCCCGCGGAGGCCCCAGGACCATCAGAGCGACAAATTTCTCTTGACATATGATT[A/G]CCGGCATCGTCATGAATAATGAGGGTGTCTCTGGATGGCAGGTTGACAGTCGGCGACCCAAGGGGGTTGTGGCCGGGCCCTCCCACCGCCACAGACTCCCT |
| comp107868_c0_seq3:2363-2364 | comp107868 | 2363 | C | T | 166 | 0.716867 | 0.283133 | 63 | 0.0952381 | 0.904762 | GACGCTTCTTTCCTCGGCTCGCACAAACCCTTACGCTAAAGGATTTGTGGTCCTGGCACGAGTACTAGCAGCAAGCGCCACGTGTCGGAAACCGAAACCAG[C/T]GGGATGTCAGGGTAAGGATAAGGATGAGGGTCAACGCTATGGCAGGGATATGGTATGAATGAGGGTGAGGGGTGAGGGGTGAGGTGGTGTCACAAGCGTCA |
| comp105016_c0_seq1:3836-3837 | comp105016 | 3836 | A | G | 167 | 0.748503 | 0.251497 | 126 | 0.126984 | 0.873016 | ACACAACCTTTCACATCAACATTGGAAGCCTTAACGAAAGCACCGTGGGGGTGAACATGGTCGTACAGAATGATCAGGGCAACCATCACGCGGAGGATGAA[A/G]AGTTGTGTTTCCTCTCGCCTGAATCTTGCTATTAAGTCTGGATTCTCCAACATTCTCTGGCAGACCTTGGCCATGGTGCCAAGGGTTTCTGTGGTATTCTC |
| comp96166_c0_seq2:1674-1675 | comp96166 | 1674 | C | T | 158 | 0.632911 | 0.367089 | 87 | 0.0114943 | 0.988506 | GACCCATCCCAGGAACCAGTGTGCGGCATCACACTCTCCCCGAGGCCCTTCAAGCTGGTGTTCGAGCCGCGGGAGTCGAAATGATGGCGATGCTGCGGGCA[C/T]CGTGGCCAGGTCCACACTGCATGGCTGTGACACTCCCTTGGAATATGTTACTGGAACATGTTTCGAATAAAGATGTATTTCACAATTGTTTAGATATGTTA |
| comp107654_c0_seq1:3451-3452 | comp107654 | 3451 | C | T | 128 | 0.875 | 0.125 | 67 | 0.253731 | 0.746269 | AGGGCATGTTCGTGCTCTGTGAATGTGATGAAACCATATCCCTTGCTCGCAGGAGTACCATCATCAGATAAAGTCTTCATGTCTTTCATAATGCGAGCCTC[C/T]GTAATCTTAGCCCCCTTTGGTGAATTGTCAATAAATATCTTCTTGAACTCTTTATCAGTCAGCTCCTCTGGCAGGTTGTTCACACACAAGCGATTCGGAGA |
| comp100281_c0_seq2:2029-2030 | comp100281 | 2029 | C | T | 65 | 0.661538 | 0.338462 | 98 | 0.0408163 | 0.959184 | CATGAAACTGTTCCACTTGGGCCTCGGGCAAGTCCACTCCGAGCAGGGCACGTCCTCCTCCCTCATCTGCGTGACGACGTTGATCTCACTGTACTCGAACT[C/T]GATGCCGAGTTCCTCGAGGCGCTCGAGGACGGCCTCCAAGTCCTCGGGGTTGACCAGCGCCTCGCCCCAGCCGGCCTCGAAGGGGCTCACCGGCACCAGCA |
| comp105629_c1_seq1:2674-2675 | comp105629 | 2674 | T | C | 96 | 0.802083 | 0.197917 | 11 | 0.181818 | 0.818182 | GGGAATTCCAACCCCAGCCTCTCTCTGTCTGTTGTTTGTTATGAATTGCGCCTGGCTGGGGGGTAAAAAAGGGAAGGAACTTGAGGGTTCATCGTGTGATG[T/C]GAAAGTCATTAGCCTCAATAAGTAGCAGAAATTACATTTTGTGCAAATGTTATGTGATAAGTTATTTATATAGTAACTTTAAGTTACTTATAAATGTTATG |
| comp107020_c0_seq1:2616-2617 | comp107020 | 2616 | A | C | 51 | 0.72549 | 0.27451 | 38 | 0.105263 | 0.894737 | ATTAACTACTGAATTACTGACAATTAATTCTCCTACACAACATAGCCTACAGAAATTTTATGAATCCTGTCTCTTCTGGTACTTTCTACTAGCAATGAAGC[A/C]TTGTTTCTTACCACTGGGTTAGACATGTAGCCCTGGTCACAAGATGCAAGATCACTGACACATTACTTTCTGGCTATGCAGAGAGGTCATAGCTGAGCTCC |
| comp90332_c0_seq1:399-400 | comp90332 | 399 | C | T | 66 | 0.69697 | 0.30303 | 26 | 0.0769231 | 0.923077 | CAGTCTTCTAGCTCTGATGACTGCAAAAGAGGGTCAGAAAGTGTAAAAGATAATCTATTGGATACTTGCTGTTCCTCCTCAGGCCGTTCCAGAATAACAAC[C/T]TGAGGCTTTGCTTCTATACTTGGCGCAAGGTCCTGGAAATAATTAGGTTCCTCCTCCTCTTGAGCCTCTGTTTCTTGTATTTTTCTTTGCTGGTTTAAGCG |
| comp100701_c1_seq2:219-220 | comp100701 | 219 | G | C | 64 | 0.015625 | 0.984375 | 118 | 0.635593 | 0.364407 | GAAGAGGGAGAAAGGCTGAAAGGAGAGGGAAAGGAGAGGGGAAGGAGAAAGAAGGATAAGAAGAGGCGAATGGAGGACAGGATGTGGGGAAGAGGGAGATG[G/C]GCAGAAGGGAAGGGGGAGAGAAGCGGAGGAGAAGAATGGGGCAAGGGGAAGAGGAGGGAGAATGGGGTAAAGGAAGAGGAGGGAAAATGTGGAGGGAAAAA |
| comp95176_c1_seq1:667-668 | comp95176 | 667 | G | A | 84 | 0.0833333 | 0.916667 | 64 | 0.703125 | 0.296875 | ATGATGGAACTTCGACACTAATGGCAGAGAAGTCTTCCATTTCTCAACCTTTATGATTCAAGACATCAATTAATCACCCAGTGCTCTTCTTTAATGTCTTA[G/A]GGGAATAAATTGATGGAAAGTCTTTGCCAAACTTTAAAAGAAAATAAATGTTTAATGCTATAATGTCCATTTCATGATCTTAAAAGGTTTTTATGCTCTTG |
| comp107035_c0_seq2:1837-1838 | comp107035 | 1837 | T | A | 29 | 0.0344828 | 0.965517 | 101 | 0.653465 | 0.346535 | CGCCGAAGGTCGGGAGAGTGAACTGGGGCTCCTGCAGGAAGATGCTCTTGGTCCCCCGGTGGTCGTGCTTCGTCATCGCGTACTCGGTCACGGGGTCGTCG[T/A]ACGAGTAGGCCCAGATCACGCGAATGGTGTCGCTCGTGAGGTGAAAATCGTGTTCCTTGTCGCACGTGTTCCAGGGCCTCGTGAACCTGAGCACGGTGTGT |
| comp84358_c0_seq1:120-121 | comp84358 | 120 | C | G | 76 | 0.197368 | 0.802632 | 98 | 0.816327 | 0.183673 | AGGGGATGGTACCTCAAGAACACAATATTTTCCATCACAAAAGACCATTGTGTTTAAAAAGTTAATAATGAACAAAGACAGTTTGTTTGTCGAGACTGACC[C/G]GCCTGTGATAAAGAAGGCTGTAGTGATAAAGATGGTAAAAATAATAATGGCAACAATAACAATGAATAACAATAACAACAATAACAACAACAACAATCATA |
| comp100456_c0_seq10:1315-1316 | comp100456 | 1315 | T | G | 57 | 0.701754 | 0.298246 | 12 | 0.0833333 | 0.916667 | CAGAGTAGTATGTCGGGGTTTTCTAGTCATTACTGCTATTGTTATGATTTTTTATATTATTGTGTTTTGTATCATTATTAAGTATGTTTTAATGCATTTAT[T/G]GTATGATTATTTGCTTATATATTTTTGAAATGTGTTCCTTCATTCATTGATTTTTTTAAAAAGTGATTTACTGTATATATATGTTTTTGATTTTGTATGAC |
| comp107696_c0_seq1:651-652 | comp107696 | 651 | C | T | 53 | 0.849057 | 0.150943 | 26 | 0.230769 | 0.769231 | TTGGGATCAAATTGCTCGACTTGTCTGGTATTCCTGTTTTGTCTTTGTGTTTCTAAAGCTTTGTTTAAGTGTTACTTAAGGAGAAGCTTCAACCTTGGTCT[C/T]CTTGGACGTGTTGGTTTTGAGTCTCATGGCAAAAGGGCTCATAGTTGACATGGGCGAGGCGGGGGAGTGTGTAGGGGCGGGGTCTGTTTTCTGGGAGCGCG |
| comp96808_c0_seq1:803-804 | comp96808 | 803 | C | T | 125 | 0.704 | 0.296 | 105 | 0.0857143 | 0.914286 | GACTATTTGCACTTAACTTGGCAAGGTTATCGCCGTGCTTTTGAGCCAGTCTTCGAGTTGTTAACACAGCTGCTGCATGAGAACGACAAGGTGATTTTGAA[C/T]GATGCAACGCTGGCATCGCCTGCATCCGACAACCCCCCTTCGGACAACCCAAGTCCCTGTGCTGAGTAGCCTCCCTGAACTTCGAAAAAATATGCACAGCT |
| comp99928_c0_seq2:665-666 | comp99928 | 665 | T | C | 152 | 0.730263 | 0.269737 | 107 | 0.11215 | 0.88785 | AATATTGATGTACTACATCTATGCCTTTTCACAGATCGTCTTATATTTTGCCATTTGGTGTTATAGCAGCACAGCCTTATTATCATTAGAAATGGAAAAAA[T/C]GAAATGTATTTTTCTCAAAGAGATTTCTATTTTGGCTTAATAAGACACTTTGGTAACTAAGACTTAGCACAGTGGATACTAAAAGATATGCTAAAGATTAA |
| comp102301_c0_seq2:136-137 | comp102301 | 136 | A | C | 379 | 0.720317 | 0.279683 | 635 | 0.102362 | 0.897638 | CTTCAAATCAAAAGTAGTTTCGATAGAGTAATTCCCGTCCAGACGCCCAAGAGCAGAAGAGCACCCAAGGAACACCAGTGGAAGCTTCAGCCCCGAAAGGC[A/C]CTATATTTGCTCCGCGTTGCCATGCATGTGCTGCAAGACCCAGTGCAGGAACGCCGAGACCTCCGTGTAGACGCCGGGGTAGAAGGAGTGGCCGCAGCCCT |
| comp100474_c4_seq1:1616-1617 | comp100474 | 1616 | T | A | 60 | 0.833333 | 0.166667 | 65 | 0.215385 | 0.784615 | TTGCAAGTAAATATTTTGTGAATATACTTTCAATAATAATTTCTTGGGTATATGTTTGGCCTAAAGAAAAGTTCTATATATGAAGGTGTTATCCTTTTTTT[T/A]AAATTGTGCAAAGGAGTTTTCCCAAATAGCATACGGACATGCTGCAAAGTTTAAAATGGTTCTCTTCCATAGCACACATTCATAATAGATTTAAGTATTTG |
| comp107391_c1_seq3:362-363 | comp107391 | 362 | G | A | 83 | 0.903614 | 0.0963855 | 28 | 0.285714 | 0.714286 | AAGGGCGTGGAGGTGATGCAGAAAATTAGTGCTGATCCGCACATTGTTCTAGGCTCTGCAACTGTCGACTCTCTTCAGACCCTTCTTGATGCGGCAATTGG[G/A]AACGATGAAGCCGACACAGGGGTGATGGTAGTAGTCTATGCTGTCGATGCAGGACATCAAGAGGCCGCGGACATGGCTAAAAAACTTGGGTCAAGTCTTAC |
| comp106442_c0_seq1:441-442 | comp106442 | 441 | A | G | 64 | 0.890625 | 0.109375 | 11 | 0.272727 | 0.727273 | AATTCCCCTTGCCCTCTGTATCCCACTTTATTCGACTATTGTTTTATTCTCACTTTACATAGTGTTCTCTATAATATAATGAAATTAGCGATGTTATGGTA[A/G]AACAGTTCATCCTTAAATCTCAGAAAGTACCAGCGCTAGCAACAGAGTCGTTTACAAAATGCAAAATTCCTCTCTGACTCTGGCCAGCCTCTGTGCCTCTC |
| comp94970_c0_seq1:274-275 | comp94970 | 274 | A | T | 53 | 0.943396 | 0.0566038 | 86 | 0.325581 | 0.674419 | TTTAACTTGCACGGCTGAAGGAAGACACTCCGCATTCGTCTGTTGACAGAAGCTACCCTCTGGAAGGCATTTCTTCAGGACCACGTACTGGGGTTCGTAAT[A/T]CAAGTCGTGCAGTTGGCTGTCCTTTGGTAGAAGATCCGAAACCTTAAAATGTAGCTCCTTGGGTTCGCATCCCATGGACATGAGTTCAGACAATAATTCTT |
| comp107544_c0_seq4:6982-6983 | comp107544 | 6982 | A | G | 63 | 0.777778 | 0.222222 | 75 | 0.16 | 0.84 | AAGGATGTCAAGTACGAATACCGCAGCCACTTCACTAAGCCCGAGAAGGTCACTCACCCGAGCGGAAGGGCCTACGGCCTGGTCTACGATGACGCGGGGTC[A/G]CTTCGGGAGGTCGTGACGCCCCGCGGCTCCGCTTACACCCTTCAGCTCTCGACCTCTCTCGGCTTCTACAGGCTGAGGCTGGCGCTGCCCATCGACAGCAT |
| comp102569_c0_seq5:386-387 | comp102569 | 386 | T | A | 64 | 0.828125 | 0.171875 | 19 | 0.210526 | 0.789474 | CCTTTTTTCCATTAAAAAGAATCATGAAACATTTCGAAAACTGTATGATTGCGAAATTCGTCTAGTCACTGGTATTGTTTTTGTTAACCTTTTTTGTTATT[T/A]CTGGCTTAAATTTTCTGGAAAATATCTAGCAAAATATATTCTCATAAATTGAAGTTACTGAAAAAATAGAATAGTACCATCAATCAGATGGTAACAAGGGT |
| comp107523_c1_seq2:2249-2250 | comp107523 | 2249 | A | C | 524 | 0.835878 | 0.164122 | 426 | 0.21831 | 0.78169 | GAAGGGTCGATGTGCATAGGGTTGCGATCCCCACTGAGCCGATACAAGGCAGCTTGATCCACATCTGTCTTGAATTCCTCCGTGGCATCAGGCTTCCTGCT[A/C]GGGGGGTCACAGAGGGGTACTAAGCGGTTGCTCGATCTCGGGCCACCAAAGTTCCCATCTCCTACATGGAAGGTTGACCACTGGGCTTTCGCCACAGTTTC |
| comp99101_c1_seq1:493-494 | comp99101 | 493 | C | G | 53 | 0.886792 | 0.113208 | 78 | 0.269231 | 0.730769 | TCAATTGGAGTCACTTCAGTACAGCCTCGGCTTTCTAAAACTGGTGGCTGTCTTCCTGACTATTTTGCTATGTCTGTGTGGCTTCTTTCCACTCAATTCTC[C/G]TTCTATACCCCATCCAAATCAACAGAGACTATTAAACCCACCAGCTCGAGTAAGGTAGGGTAATGTGTACTGTGGTATTTTGGGACAGACATAATTCTAAT |
| comp100735_c2_seq4:1119-1120 | comp100735 | 1119 | C | T | 139 | 0.848921 | 0.151079 | 302 | 0.231788 | 0.768212 | TCACTTCCTTGCGTCGAGAGCCAGCTGGGGCTCGGGTCTCCTGAGAGATTTTTCCGAGAGACTTTTCCTTGGCCCGGGAGGGAGGACTTCCGTGGCTTCCA[C/T]CCGTCGCCAGCTAGCTAATATTTGAATCAAATATTTGATCTAGTCGGCGGAAATATCTTCTTTTTGTTCGTGTGAATCTCTTCCTTGCATGCTTCGTGGCT |
| comp107922_c1_seq2:446-447 | comp107922 | 446 | G | A | 147 | 0.884354 | 0.115646 | 157 | 0.267516 | 0.732484 | GAATGGATGATCACTTTGCTCAATTTCAAGGTCATTCCTTCCGCCAGCTTCTCCGCCATGATCTTCCTCGTCGTCATCCTCTACGGCGCCGTGACCTTCAT[G/A]TGGGAGCACTGGTGGTTATACGGAGTGATCCAAGAGCGCCTGATGCCCTGGCTGGCGAGGACGTGCGGGCCGCGATCTCCTCACGCCAAGCTCGAGGCCGA |
| comp107150_c0_seq2:732-733 | comp107150 | 732 | G | T | 105 | 0.761905 | 0.238095 | 124 | 0.145161 | 0.854839 | TTTGTTAGAGTACTATCTGTTGATATGGTGCGAGTTCTAAATCTTTCAGAATCATCCTGACGACGCTGCTTTGGTGTTCGAGTAACTGAAGGTGATGAGGG[G/T]AGAACTTCTACCAGTGTAGGATCTGTATCCTGTGATGGTGTTGCATCTGACATTTCTCCATCACCTCTTGTGAAGGTCCGGAACCTGTCCTTTGCCTGTTG |
| comp101467_c1_seq8:716-717 | comp101467 | 716 | G | A | 143 | 0.699301 | 0.300699 | 109 | 0.0825688 | 0.917431 | GTCCTGGTGGGCGGAGACCGAGAGACCACGCGATTCCCGCCCTGGCTCATAAGGACCATAAGGGCGACGACCAACTTCACGGCCACGATCGGTCCGACAGG[G/A]GGTGATCGCGGGTACAAGGCCATTACGGGGTTGCCGTCTTGGGGTATCGCCTGGTGGATAAACGACATGCTTATCCCCGAGATATACGTGGAAAGAGGACA |
| comp99561_c1_seq20:179-180 | comp99561 | 179 | C | G | 20 | 0.3 | 0.7 | 60 | 0.916667 | 0.0833333 | GTGTGGCGCGTTATTGATATGACGGCTGAGGATGGTAGGCCTGGGGCTTGAAGGTGATGGGGGGACCGTACTCGTGGGGGTACTGAGCCTCGCCGTAGTAG[C/G]TGACCTCAGCCTGGTAGCCGTTGTAGTGGTCAGCCACGTAGTTCACCGTCTGCTTGCGGCCGTCGGGGAGCTGGACGGTGTAGGATCCCTTGACTGCCTTG |
| comp100736_c2_seq21:3809-3810 | comp100736 | 3809 | A | C | 52 | 0.75 | 0.25 | 15 | 0.133333 | 0.866667 | ACTGAGTGTTGATGAATAACTCTCTCACTGAGAAACTGAATGCAACATGACCATTTAACTGGTTAAATGAACAAAACTCAAAAAGTTTTCTGTTTCAGTAT[A/C]ATTGGGAGAGCAGGCAAGCAACTAGTGGGAATTTTGCTGGCCGTAAATGAGTATGATAATTCTCTTGATGTGATGCCTGCTGCATTACACTGTGGATAGAA |
| comp106436_c0_seq2:802-803 | comp106436 | 802 | A | C | 94 | 0.893617 | 0.106383 | 83 | 0.277108 | 0.722892 | ATATACAACTATGTCAGCATTTCTAACATCTTACTGTCTACTATGTCATCTATTCACACTGTTTCAGCAAAATTAGGCAATTATCCAACTCTTATACTGCA[A/C]AATACATACTTAGCCTTCTTAAACTTAATAATGCCTTATATTCCTAAATATAGAAATAACATCTTATACTTCAAACTGTGATAAATGTTATTCTCTCTTTA |
| comp107591_c0_seq8:2046-2047 | comp107591 | 2046 | A | G | 1012 | 0.323123 | 0.676877 | 975 | 0.939487 | 0.0605128 | TCATTGCCTTGTCATATTGCACCGTCTTGTCAGTTCACGCCCATCTGTCTCTGTCACGTGACTGTGATGTCGTATCTATCTGTCATGTCAAATCACTGTCA[A/G]TCTGATATTCTGTCAAGTGTCAGTCTGAAGTTTTAGTTCTGTCAAATGTCAGTCATAAGGTTTATTTCTGTCAAATGTCTGCGGTTATCTGAAGGCTTGTC |
| comp97557_c2_seq1:163-164 | comp97557 | 163 | T | C | 158 | 0.639241 | 0.360759 | 87 | 0.0229885 | 0.977012 | ATCTCTTCCTTTCTGCTACTTCTCGTATGCTCTTTAAGAATGCCTCCTGCAAGAACCTTCCTGTCCTCCGTCCCCTAGTCATTACTAAAGCATTTTTTTTA[T/C]TGTCTTCTATTCTCTCTTCCTTTCTCTTCCTGCTCCTGCTCCTTCTTCTTAATCTTCTTCTCTCTTTCGGTCAGTGCTGGAGTCGGTCATTTATTTTGTAT |
| comp99108_c4_seq2:1346-1347 | comp99108 | 1346 | C | T | 62 | 0.870968 | 0.129032 | 51 | 0.254902 | 0.745098 | GCGGTGGCGCAGGTGCCCACGCGGCCCACGTACACGACCCACGTGGCCAGCGGGAAGGCCAGCAGGAGCCAGCACAGAGCGGACGGCAGGAGCGACAGCAG[C/T]AGGAGGCGCCGGAGACCCAGGTGCTCCATCAGGGGGCCCGAGACGAAGCTCGAGAACATCCACACGAGGCCCAGCGACGACACCAGCCACACGACATCCAT |
| comp107013_c0_seq3:1662-1663 | comp107013 | 1662 | T | C | 456 | 0.734649 | 0.265351 | 236 | 0.118644 | 0.881356 | TCGTTGTCCAGCCAAACTAGTCCTGTCACTTGCGACTGTGGGTGCGAGTCATTGTTCTAGAACTTGACGCTCCATTTTTTCACTACTCCATCGTGGCCTCC[T/C]GAGATTAGTTGTTCGTTGTTCAACCAAACCATGCCTGTGACCTGGGCTTGTTGGTGCGAGTTCTTAATAATGATGTGCTTGTTTGTCTGTGTCATGCTCCA |
| comp94214_c0_seq3:2462-2463 | comp94214 | 2462 | C | T | 705 | 0.770213 | 0.229787 | 415 | 0.154217 | 0.845783 | GAAAGGTGCGCAGAAAAGATGAAACACCTGTAACTGCGTGACAGCTCTTTGGTTTTACTTCAGGCAGGCTCTTTAACATTTTATCTTCTGGTACATCAACA[C/T]GCCCATATAAATACGAGGTACTTTTTAGACTTCCGGCTAAGAAGAGCTTAAAGCAGTTTACTCCCAAGCTATGCAGTGCTGTTTAAAGTACACAATGTACA |
| comp105609_c0_seq20:260-261 | comp105609 | 260 | A | G | 43 | 0.953488 | 0.0465116 | 80 | 0.3375 | 0.6625 | AACGGATCATCCATGAAGTCCATCAAATATTTTATGCCGTTAAATACTTGTAAATACATGGTGTAGAAATAAATAGTACAGTTTAAATAATCATGGAGCAA[A/G]TGCATGATATATAACTTGTTGATAATAAAACTGCATATAGATTTTTAGTTCGTAATAGATGAACAGTACGACCAGTGCATTACACCAGTTTCACACAATAT |
| comp105315_c1_seq7:1138-1139 | comp105315 | 1138 | A | G | 277 | 0.299639 | 0.700361 | 391 | 0.915601 | 0.084399 | TGGACTGCTCTCTTCTCTTTCTCTTAGGTGTGTGTCTTTCTCCCCCTTTCTTTTATTTTATTCTTTCTCTCTGTGTTTCTTTCTCAGTTTATTTTTTCTTT[A/G]ATTCTTCTGCCTCTCTCTCTTGTTCGGTCTCTCTCACTCGGTCACAGCGGATAATCTGACACGACCTTGGACCAGTGAGTGGGTTATAAATAAGGCTCTAT |
| comp107718_c1_seq2:955-956 | comp107718 | 955 | T | C | 120 | 0.308333 | 0.691667 | 581 | 0.924268 | 0.0757315 | ACCTACCAGCCGGGAGAAGTCTTGGCCGAGACTTTCATCAAGATCTACGAGACTGACTACCAGGGCGCCAGCAGAGTCTTCACGGAGCAGTTCACTGTAGA[T/C]GCCTCTCTGGGTCGTGACCTGACCTTCAAGCTGGAGACCAACACGGCCTCGCACGTGACCAGCCGCCCTCATCTCCTGCGTCCGGATGGCACTCAGATCGA |
| comp105158_c1_seq2:828-829 | comp105158 | 828 | G | A | 848 | 0.71934 | 0.28066 | 135 | 0.103704 | 0.896296 | GCTGTTTCTTCGGCGAGCACGAGGACGAGCAGGTCATCCTTCAGAGGATCTCGTCCCACGCCTTCCGAGGACCTGGCACTCGAGCGGAGGATCCACCCCAA[G/A]TTCCGGCTGCTTCCAGGAGGTTCGAGTGCCAGCGGAATCGAGTGCCACGGCGTCGGGGAGTGGGAGGGCGTGCCTGGGGTGGCGGTGTGGTGTGCCAAGAA |
| comp107942_c0_seq3:6336-6337 | comp107942 | 6336 | T | A | 294 | 0.632653 | 0.367347 | 58 | 0.0172414 | 0.982759 | CACGTAAACACAGAAGCTAGGAGGACCGCGAGAAAGAGTCCTCGACAGGGACTCGCATGTGCAGAGGCCATGGCGGGGTAATCCTATGCAACAGCTACGAC[T/A]GCCCGTGTTCTCCCGTCTGCGTCCGGGCACACACTGAACGTACGAGGCGAGTGCGCTCCTACTTAACGCCGGCGAGTGGCATCCCCAACGGTGAACTCAGG |
| comp96063_c0_seq1:817-818 | comp96063 | 817 | T | A | 96 | 0.854167 | 0.145833 | 67 | 0.238806 | 0.761194 | GAGCTGAACTGCTTTCCAGTCACATTACTAACTCCAATAAAATAGTGTGTATATATATAAGAAAAATGAGGAAAAATAAGTGACATTATCAGGTGAAAATT[T/A]ATCTTTTCCACATGAGGAATTTTTATATTTTATATCATACTTTGGATAAATTCTAAGTATATCATATTATTTCATAGTTCAGCACTTATCAATGAAATTAC |
| comp102258_c0_seq42:4382-4383 | comp102258 | 4382 | C | G | 86 | 0.860465 | 0.139535 | 53 | 0.245283 | 0.754717 | AGTCTTCCTCGCTACTCCTTTTCCTTCCCTCTCCTTGCCTCTCTAGTTATTCCTTTTTCTTTCTCTCACTTCACTGCCGCGCACAACACGGGAAGGAACGA[C/G]CGCAAAGAGTCCAACGCGAAGCCTCAGTGGCGCCTCCCCCTCCGAGAGCGGCCGTGCTTCTCCCCGACCTGGCTGCCACAACACTGGCACCCCTCGTCGCC |
| comp105664_c1_seq2:170-171 | comp105664 | 170 | C | A | 194 | 0.737113 | 0.262887 | 41 | 0.121951 | 0.878049 | TTTAGGCCTGGCAGATGTAAGCAGGGGACTGACTAGAAGGACCACCTACGTTGAGGTACCTCCTGGGCGTCGTGGGATCAGCAGGAACCAAGAGGGTGTAC[C/A]AGGTGGCGTCTGAGTTCTTGGGAGGGAGGTTGGGCACCCACAAATTGGACTTGACACTCAGCTTGTCGCCATTGAGCCACTGCCATCCTGACCCGACAGTT |
| comp106242_c0_seq2:1534-1535 | comp106242 | 1534 | T | C | 120 | 0.641667 | 0.358333 | 113 | 0.0265487 | 0.973451 | ACACACGATTTGACATCTTCTCCGAGGGCATTCTTCAACCGCGATATGATGTCAGGGTACTTGGTTGTGTTGTTTTCTTGGTTCCTACTGTATATGTTAAC[T/C]GTACCATCTTCCTTCAAATGAATCTGTGCTCTCTCTCCATCATACTTGAACTCACAGGTGAATTTGGCATTTTCAAACCTCTTCAGGACTTCAGACACCCC |
| comp96747_c0_seq1:307-308 | comp96747 | 307 | A | G | 274 | 0.729927 | 0.270073 | 313 | 0.115016 | 0.884984 | TCAATTGCATTTATGTCTTGAAGATTAGGATACCCATGTTTCTTTATCTTATTTATGAAAAGTTTAAATACATTTTATAATATTTTCATAAGATGCACTTT[A/G]AGTCACCAATAAAAAATGTGCTTTCAAAAGGAAAAGGTTGTTACAAATCATTAAATTTTGTTACCATATTTTTATAAGTAGTATAGTTATTTTGGTTTAAA |
| comp102972_c0_seq1:2482-2483 | comp102972 | 2482 | A | G | 81 | 0.814815 | 0.185185 | 25 | 0.2 | 0.8 | TTTGTTTTTTGGTTTACGTAGTCATGTACATATATACATATATATCGCTGTTAAACTACACTTCTCAACCTAGCGCGTGTTGGACAGATAGTTAAAAGCAA[A/G]GAAAACGGACCCAAGTTCTTCCTTCTTGCTCCACGTACAAGGTGCTTTCGAGCAGGAGAATCGGAAGAACTTTAACAATAAAAGACAAAAAAAAAAGAAAT |
| comp107366_c0_seq1:435-436 | comp107366 | 435 | A | G | 712 | 0.644663 | 0.355337 | 736 | 0.0298913 | 0.970109 | AACATTGGACTCACGAAGTCATGCGGGGTGTCTTTGTGAACGACTTTGAGAGTAGTCTCTAAACGAATGCCGAATTCACCGTCTTCGTAATACCCGGGTTC[A/G]TCAGAACCGAAGAAGCCGATCTCATAAGCATTATTGGCAGATTCATGCACAAAGAGACACATTCCGATGCCGTGGCTGGTCCCATGTCGATAATCCAAACC |
| comp104283_c0_seq3:560-561 | comp104283 | 560 | A | T | 65 | 0.830769 | 0.169231 | 37 | 0.216216 | 0.783784 | ATGAGAGCATACCACTCTCCTAGTCAGAATACCATGTTACTATACTTTTGTAAGAATTGTACTGTCGAAACCTACCCATTGCAGCTGTCTGTGCGATAGGT[A/T]ACATGTGCAAGGAGCGATGGTGGCGAAGGCCCATTGAGAAGGGGCATAATGATAAGGCCCTCACCTGTTTGCTTACAAAATGTTATCATAGCATATTGTAT |
| comp101672_c0_seq1:309-310 | comp101672 | 309 | G | A | 70 | 0.9 | 0.1 | 21 | 0.285714 | 0.714286 | ATCGAGCGCACTGTTGTCTCTTCTTTTTCTTGACATATACATAGAGGAACTCCTAGAACGAGGAAATATAAGAAAGTTTAGAAATGATTTGAAGTGACAGA[G/A]CAATCAGAAAGAGACATTTCGGAGGGACCTATAGTGGAAAGGAGTGATATTGACCAACCGACGAATTAAAAAAAAAGGAAAAAAAGTCACCAAAGAACAAG |
| comp96846_c0_seq1:1215-1216 | comp96846 | 1215 | G | C | 103 | 0.864078 | 0.135922 | 104 | 0.25 | 0.75 | TGGGGCATACATTTGAAAAAGGGATATGGTCTGAAGTAAAAGACAGAATGCTGTGACAATATTCAGAATCACAGATGTGGTCCACAAGATGAAAAGGAAAA[G/C]TGGAAAAGTAATAATAAAAGGGGGAAAAGGACAAGCACTTAAAATGCATAACATCTTTACAAGCTTATATAACCCTTAAAGAATTTACAGAGTAGGAAATT |
| comp106701_c0_seq10:379-380 | comp106701 | 379 | G | T | 143 | 0.832168 | 0.167832 | 110 | 0.218182 | 0.781818 | TCGGGAAGCAAAGTCACCGGCCCCGTGAAGCTCTTACCCCAGACCAGAACAGAGCTGAGTGCCTTAGTAGACGAGGTGTTCAAACGGTGTACCAAAGTACC[G/T]TCGGCAACGGCACCAAACGCAGCTCAGGAATGGACAGAATACACAGAAATCTTCCAGCTAATTCAGAACATAAGAAATTTAGAGAAAGATGTCCTGCTTCC |
| comp107334_c0_seq1:4454-4455 | comp107334 | 4454 | A | G | 101 | 0.782178 | 0.217822 | 214 | 0.168224 | 0.831776 | ACATATCCAATTCCTCCTACCCATATTTCAACTTTTTCTTTTTTTGTTTTGATTTACAAACGTACCTCTCAATGAATTTGATTTTTCCCTACAGAGAAAGA[A/G]TTATACATTACCATTTACAATTTGACCAACCGTCTGACTATGAAACAGAGAGGTAAAACTAGACATATATTATATACCGAGGGAAAGCTATTATATACATA |
| comp95246_c0_seq2:116-117 | comp95246 | 116 | A | G | 55 | 0.327273 | 0.672727 | 17 | 0.941176 | 0.0588235 | TATATATGGCAATTGTGCATAAGTCTGTCTATTCATTTAAAGAGATAGTGATATTAGATCTATTGGGACTAAGAAAAAGATAAAATATTGATTGTATTTTC[A/G]GATGGATCTAATTTAACTGAATAAACAAATTCATAAGAATGGCAAATGAAAATACACATATTTTTATTAAATGCAATCACTTTTTCCTTGTTTATGATTCT |
| comp105389_c0_seq3:1272-1273 | comp105389 | 1272 | G | A | 34 | 0.0882353 | 0.911765 | 94 | 0.702128 | 0.297872 | GTCTGTGGTTGTGGTGGCGGGGGGCAGTGTATTCGTGGTGTCTGTGCTTGTAGCCCCGTATGTTGTGTCCTTACTTGGCTGTTCGCTCGTGTCGGGATGCG[G/A]AGTGCTTGCGTACTCCAGCACGTCGTGGGTGAAAAGAGGCGCTTGCGAAGGGAGGTAATAGAATTGCTTTTGCTCCCGCCGCTTCAGGATCGAGATGCTCT |
| comp103786_c0_seq1:1139-1140 | comp103786 | 1139 | A | G | 380 | 0.763158 | 0.236842 | 261 | 0.149425 | 0.850575 | TCCATGTAGTTGCCACCACCCACAACAAACACAACTGACTCCTGGAATGGCGTTCTTGTTCGAGCTCCAGCCACACTGCCATCTCCTGGTCGTAACATCTT[A/G]GGGTCAAAGTACTTGTACTCTTTCTCTTCTTCACTGTTTTTCAACTCCATCAAATGGTCTACAATTCTGGTAATTGGCAATTTATTTTCCTTCAGCACGAA |
| comp99236_c4_seq12:306-307 | comp99236 | 306 | G | A | 272 | 0.783088 | 0.216912 | 490 | 0.169388 | 0.830612 | ATACATAAAAGTGACGTGATCTGCATCATTGTCTAGCGTAGAATTCGTCCAGTTTATTTGTTGGGAGCACCATAGGAGCGGGAGACTTCTCCACGGGCGTT[G/A]CGGGCGTCCTCCTCGGCGGCGAAGGCGATCTGGTCGAGGACGAACTGAGGGATCGGGTGGGGGAACTCGGGAGCCACGGGCAGGTGGGCGCCCTGGGGCTG |
| comp97003_c0_seq23:1819-1820 | comp97003 | 1819 | T | A | 62 | 0.693548 | 0.306452 | 25 | 0.08 | 0.92 | TGACACAGAAATTCCCGAAAACCTTACATGAGATGCCGTGCTGGTAAGGCAGATGCTCAGGAGATACACGATCGGCTGAAGTTTGGGAAACTGCTAAAATA[T/A]ATGAGCGGAAAAAATGAGGTACTCACGTTTGGCTCCATCGATGTACGCAAGGAATTCCCTGGAGGGAATGGCGGGACCGCTGCAGAAGTCGGTGAAGAAGA |
| comp105195_c0_seq6:428-429 | comp105195 | 428 | C | T | 130 | 0.738462 | 0.261538 | 216 | 0.125 | 0.875 | AAGCCGGTCCCCGAAAATCCGGCGGCGAATACGATGTTCTTGTATTTCGGATGACGGTCCAGGATAAATTCCTCGTCCGGCGTGGTCGTGTACATGCAAGA[C/T]TCCTCTATCGCAGGCCGAGGTTCGAGGCAAGGGAACTTCTTCCGGACGTACTCCATCACTCGCTCCCTTATCTCCGTGGAATCCCCTTTGTCTCGCCACTC |
| comp93862_c0_seq1:109-110 | comp93862 | 109 | C | T | 91 | 0.791209 | 0.208791 | 101 | 0.178218 | 0.821782 | TGCCAGCTCCTCCGAAACCTCCGCTGGTCCCCCTTCCAAACCCACTTCCCGAGGAACCTCCAAACCCGCTGCCACCGAAGCCTCCTCCTCCAGATCCCACA[C/T]TGGGAGTCCCGTAGGACGTAGACGGGCTTTGGGGCAGTGATGCCACGGCGGCGATCACGCCACAGAGTACCAATTTCGAAATCATGACCGTTGGAAGATGG |
| comp103601_c0_seq1:1273-1274 | comp103601 | 1273 | G | A | 13 | 0.769231 | 0.230769 | 128 | 0.15625 | 0.84375 | TTGATGATAAATAATAATATACAAGAGCTATATGAAAACACGGGCTTTAAATACATACATGCGAATATATGGTCATACCGTCCGTGAATATACACATATCT[G/A]ATATGACTCCTGTAAGAGAGGTCAGGTCATGAGTAGACAGACGAAATGACATCAATGCCAGAGTCACAATTCCTGTCATGAATTCTACAAATCGGAAACAC |
| comp95324_c0_seq1:222-223 | comp95324 | 222 | A | T | 117 | 0.692308 | 0.307692 | 88 | 0.0795455 | 0.920455 | CTCATGAAAATAAAAACAGGGCCACAATCAGAACTGGAAAATCTTACGTTTCGAATTCATACAGAGTTCCTCATCGGACGTAAAAAAGTCTGAAATGGATC[A/T]ATTTTGGGGTTTTACTTCGTCTGATGAGGAACTTTCGGCGAACTCGAAACGCTGTCCGTTGTCAGTTCATGGTGCAACAGCGTTTCTTATCATCGTTGTGT |
| comp105127_c0_seq1:3923-3924 | comp105127 | 3923 | A | T | 46 | 0.76087 | 0.23913 | 54 | 0.148148 | 0.851852 | CGTAGTGGAAGTAGAACACCAACTCCCTCCTCATCCAATAGTTCCACCACACGTGCCACCACCACCAAACTGACCTCAACATCAAGGTCTTCCACCCGCAT[A/T]CATGGCGACATCATCAGGGATCCATGGCGATCATCCTTGAGGGAATCAGCCTTGCCTTCCCCTCAAGAATACGCACGCCTTCCATCACTCAGTCCCTCCGT |
| comp94359_c1_seq1:1058-1059 | comp94359 | 1058 | C | T | 72 | 0.819444 | 0.180556 | 29 | 0.206897 | 0.793103 | TCCAGCCAAGGGGAAGGACCAGCTTATTGTAAGCATCATCGTAGATGACAAGTTTGGAGCACTGTGAAGAAAACAAAGTTCTACTCCCAATATCATCATGT[C/T]ACTCCTTAAAACAGGGTTACTCTGGTACTATACATATGTGATGTACCAATCAAAGACACATTTTTTCATGCAGAACTGTAAAGGAGTTGACTGTTTTTGTT |
| comp104646_c5_seq1:1670-1671 | comp104646 | 1670 | T | G | 88 | 0.625 | 0.375 | 80 | 0.0125 | 0.9875 | GCATTAAACAAGTCTATCAAATTTCTCGAGGTAAGGTTGAGAGGCTGAGCAGGCTCCTCTTGCTCAACGACATTTCTGTGTGGACTTGGGGTGTTAGCCAT[T/G]GAGGAGTTGAAGCTTGTGCTCTCATTCAATCGCTGGGTGAAAGGTCTTCCTGTCCTGGGCAGGGTGATACGGCGGCGGATGAAGGTTGGACTATCGTCTAT |
| comp99251_c0_seq1:177-178 | comp99251 | 177 | C | A | 57 | 0.877193 | 0.122807 | 34 | 0.264706 | 0.735294 | TTGAGCGTAGTGTCTCGAAGCGTCCTGGAGGCCTGGTCGCTGACGGAACACCTCTCCAGCACACTGCTCTCGGCGCCTTTCGTCAGCAGCCAGATTTTTCT[C/A]GAAGATTCGTCCCGCACTGCCACCGACATGCATTTCCGGTCTGAGTCGAACTCGAGGATCTGGAGCCTGGTGAAGGTCCTCCTCGACCCCCTGACGCTGAG |
| comp102722_c0_seq6:1284-1285 | comp102722 | 1284 | C | G | 56 | 0.910714 | 0.0892857 | 57 | 0.298246 | 0.701754 | GTGGTTAATGCCATCTTCACCACGTTCCTGCAGGCTGCCATTGCACCGGTCTTCGCTGTGAATCATCTGCCGGTGTTCACGTACCCGTTCTGCATGAGTTC[C/G]CTGATGTTCCTGGCCATGGCGAGCAACGGGGGGATGGACAGCGAGAGGGTCGCCAATCTTACCTTTCCTGAACAGCATTTTATACTCCATCTAAAGGAATC |
| comp96658_c0_seq1:322-323 | comp96658 | 322 | C | T | 93 | 0.741935 | 0.258065 | 54 | 0.12963 | 0.87037 | TACATGTACATTTTTTCGTGAACAAACGAGCCTTTGGCAAAATCAAGACATTTTATGATCTGCGACAGTAAAAGCTCTATTTTTTTAAGACATTTTAAGAC[C/T]TTCTAAGGATGCATGGGAACCCGTGATGTAGAGGAACTATTATATCGGTGGTTAAGGCACAATTCGAAATGGGTGAAAACATAACTATAAGATTTGTACAT |
| comp106724_c1_seq2:238-239 | comp106724 | 238 | A | G | 204 | 0.931373 | 0.0686275 | 94 | 0.319149 | 0.680851 | ATATTCGCAGAGCACACGATATTTACACCCCATCCATATTTTCTTTTTACATTTGTCACAATGAATACTGTTCGTTCCTCTATCAGTGACGTCACAGGGTA[A/G]TTGGCCCGGCCTTCGGTCCCAAATAATTTTACAATTGTTAGCTTCACGCACAGATAAAGAACAATATCTCACATTCTGATTATCCTTCGAAATAATCGAAC |
| comp104775_c3_seq1:760-761 | comp104775 | 760 | A | G | 109 | 0.293578 | 0.706422 | 138 | 0.905797 | 0.0942029 | CTACCCCCAGGCATAAAAGTAATACATTTGAGCATCCTGGGAAAGCAATGAATTCAAAGGCCAGTAGCACTAAAGTTTATCTGGAACACTGGAAAGACCTC[A/G]TGAAAATGTAACTAAAATGAATTGAATTTCTTAATGGGCATTATTACCAAAGAATATCCATTGCAATGTTGGGGAGGACTGAATAAAAATGCACAAGTTAA |
| comp107408_c0_seq5:2451-2452 | comp107408 | 2451 | A | C | 43 | 0.790698 | 0.209302 | 84 | 0.178571 | 0.821429 | GAGTCCGAAAAGTATTTTGTAGTTTGTTTACTCTTAATGAAGGGGTTGCATGTGACGTGTATTTTGGGCCTACATCAGCGACAGTAAGAAGGCATACAACT[A/C]AAACCATACACAATCAGTTCATTATCTTAGATCGTTGTGCAAAATATGAAAAAAATGCATCCTTATTATTTGAATATTAAATTGATCATTATTTTGTTTTC |
| comp96475_c1_seq1:626-627 | comp96475 | 626 | G | A | 63 | 0.68254 | 0.31746 | 71 | 0.0704225 | 0.929577 | AGGCAACCCTTCTTACCCCACAACTGTTGTCCAGTCTTTAAATTGCCCTTTAGTGCGTTTGAGCCCTCCTTTATGTCACATTATTAAATATTTATCTACCC[G/A]TTTTTGCACTGTTTTGAAATGGTTCTCATACTCCAGTACTGTCACTATACTATGCCAATGCGAAATGCTGAACATCTCATTTTACAATTTAATATATGCTC |
| comp100351_c0_seq7:420-421 | comp100351 | 420 | A | T | 215 | 0.623256 | 0.376744 | 88 | 0.0113636 | 0.988636 | TGGATTTCAATATCATCAGCCTCTCTGAAAACCTTCTCTGCCTCTTCTTTGGATTTGTATATCATGTGAGCTAAACATCTGTTAGGCATGTAAACAACCTC[A/T]GATGCTGTTGTAAACACCTTCTCCAAGTCTTCAAAAGTAGTCTGAACTGGTACATTTTCCATAAACAATCGAAAAGGGTTTATACAAATGTTATCAATTTC |
| comp107132_c0_seq2:717-718 | comp107132 | 717 | T | A | 54 | 0.333333 | 0.666667 | 73 | 0.945205 | 0.0547945 | TGTAGTCATACCTGTAACATCATATATCGTATATGATTATAATATAATTCAAAAGTCAAAATGTACAACATGTGCATACTTATAACAAAAAGTAACAAAAT[T/A]ACATTCTATATAATAGTAGTATATAATTGTGCATAGTCTTCATGATTTTTCACCTTCTTTTACAGAAAAGAACAACAAAAATGGAAATGTAATACACAATC |
| comp90582_c0_seq1:429-430 | comp90582 | 429 | G | A | 328 | 0.307927 | 0.692073 | 348 | 0.91954 | 0.0804598 | GTCTTGTGGCATGGTGATCGGGGGAGGTACTGTCCTAACAAGATTGTTCTGCAGTTTAAGATCATCGTTACTTGGCGCGTGTGGTTTCATTATGAGTCCAG[G/A]TTTCTTTTACACGTACGCCTCCTGTGTATAAGAGCGATTAAAAGGGTTTCCAAACATAAATGTTAGGTCTGTTTCTCCTGATTTTGATGTTATGTTGCAGT |
| comp103305_c0_seq23:2423-2424 | comp103305 | 2423 | T | A | 50 | 0.8 | 0.2 | 53 | 0.188679 | 0.811321 | CTCTGCAGGTCTTTTCTCCGATATTCACCTTTGGCAGGAGAGATGAAAGGTTTTCGCAAAACTCTTCCGAGACAAACTGGTGTGTATTTTATTATTTTTTT[T/A]AAAGGGATGTCGACGTATTGTCACAGTCCGGGTCTAACGCCCCTTTCGGTCCCCCTCGACAGCGGTGACCAGGTTACGTTTTTTTGTAATACATTTAAAAC |
| comp100580_c1_seq1:653-654 | comp100580 | 653 | A | C | 62 | 0.790323 | 0.209677 | 67 | 0.179104 | 0.820895 | AAATGTATTAAATGCATGAGTAATAGTTTTCCCTTCAGTAATCCAAAACAATCCAAGAATAACTTGAATCACATAGACAATTGCTTAAATACACATTTTCA[A/C]ACAACTTCAAAAAAAAATTATTTATAACACAGCCAAATGTTAACTCCATACAACTATGCATGATAGCTATTACCATCAAATCTTTTAATGAAATTGGCTAA |
| comp101868_c0_seq1:960-961 | comp101868 | 960 | G | A | 30 | 0.833333 | 0.166667 | 54 | 0.222222 | 0.777778 | TATAACAATAATGAAATGCTAATGATTTAGTAAAATCCTTCTTGGCATGTTGACAAATCATGCGAACTTTTCTCTTTGTCCACTGAGATAAACTTCAAATC[G/A]GTAATACAGATCACAAACATAAATCTTTAATTCTCTCTTTCACTCACTCTGCATTTCGCACACATGCAATTTTTTATATTTTCTCTTTTTTTTTTAAATTC |
| comp107178_c1_seq2:1557-1558 | comp107178 | 1557 | T | G | 105 | 0.819048 | 0.180952 | 24 | 0.208333 | 0.791667 | TTGAAAAAAAGTCAGGAATGCTGAACTTTTGAAGATATACACACCGTGGGGACAGCTTTAAGATTCATAAAAGTTATAGAGTGTATTTTGTGTTTCTTATT[T/G]TTTTCTAGCATATTTACTCAGTAATAGTTTTGGCTTGTTATGAGATCCCTGTAACAAGAATTTGAAATGGATATTTTATCTAGATTTATCTAGATGTGTAG |
| comp107947_c2_seq1:2754-2755 | comp107947 | 2754 | A | G | 71 | 0.633803 | 0.366197 | 86 | 0.0232558 | 0.976744 | GAGGGCTACACCGTCGACGTCCTCGGGAACTCCTACATCCTCTACGTGGCCGAGGCGGACGTCAGGCACGCCGGCTCGTACTTCTGCCAGATCGTGGACGA[A/G]GTGGAGGTCTACACCAAGAACTACACCATCAGCATCGATGATATGACACAGACGTACATGGGTCAAAACGGCAGCATCAACTGCTACGCACAGCCGGGAGA |
| comp99931_c0_seq1:306-307 | comp99931 | 306 | C | T | 54 | 0.333333 | 0.666667 | 89 | 0.94382 | 0.0561798 | CGCTTGCTTTAGGTTGCTTTCGCTAAGAACTTATTTTTGTCTGTCTTTCGTTCTTCGCGTTTCGTTTGGGACAGGGGGTAGGGTTCTTTAGCAAAGCAAGA[C/T]CGGGCAAGCGAGTCCTTCGAAGAGCAACCTAAAGCAATGGATCTTGGTTCACTTGATCGCCAAAATGTTCTCTCTTCAAGACCGCATTCAAATCGAGTGTT |
| comp98570_c0_seq1:520-521 | comp98570 | 520 | G | A | 140 | 0.857143 | 0.142857 | 158 | 0.246835 | 0.753165 | TTTATATGGTCTTCTAGTGTAACATTTGTGTTAATGCAATTACAAAATTGGATAAAAATAAAGATCACTGAATATCTAAAATCACATTCCTTTTCTGTGGC[G/A]AGAAGCTATGCAAGTTAATACCAGCTTATATAAACTTTTTTTTTTCTAAACCTAACTGCATAAATAACCAGCTCAACCGTAACAAAATATGAATTGAGACT |
| comp107638_c0_seq11:8101-8102 | comp107638 | 8101 | G | A | 236 | 0.75 | 0.25 | 207 | 0.140097 | 0.859903 | GGGCCTGCAAGTCTTCTTCTGTAACTGATTTTGTCATTTGGCTGCAGCTTTTCTTGCATTCTTGAAATCAGCATCAACTTCTCCTCAGGCGTCCATAAGTC[G/A]AGTGGCTCAGCTTCATTAGCATTAATTGGCTCTTTTTCCTCATCATCTTCATCCACAACAGCCTCCTTCCTTTCTTTCTTAATCTTCTTTGGTGAAGATTT |
| comp107121_c1_seq5:691-692 | comp107121 | 691 | C | G | 279 | 0.874552 | 0.125448 | 185 | 0.264865 | 0.735135 | TGCGAGGAGGCACACGAGAGGTCGGATGAGATAGTTGTATTTCAAACAATTATTCTCAACAACCTGTCAGTTAGTAATTAATAATTAATTCAGCAATCTAT[C/G]AACATGCCGTTACCAACAATTGAGAAAGAACAGAGTATACATACATCCTTATTACTATTTTTCCACGATACTTTTTTCAAACTATTTGTTCTTTCTGTCTC |
| comp103796_c2_seq1:1847-1848 | comp103796 | 1847 | A | T | 15 | 0.866667 | 0.133333 | 70 | 0.257143 | 0.742857 | ACGATAAACGAATTGATAAATGGTTCTTGATAAGTAGAAACAGTAAGTAGGTGAAGCAAAATGCATCATATATTCGCCGAGATCAGTACAATGCCAGCAGA[A/T]AATGAAGAAATGATGCATATAAGGTTAGATTAAACAAATGCATTCGTGTAATCGGATCTAATGTTTTTGTTTTTGTTTTTTTGGCGGCAAATCACAAGGAA |
| comp107382_c0_seq28:710-711 | comp107382 | 710 | C | T | 144 | 0.930556 | 0.0694444 | 109 | 0.321101 | 0.678899 | CCTAAGAAGAAGCTGGAGCCCCCGCCCCCCTCCCAGCGTGACGTGCCCATCAAGAAGAGCGATGACAAGGCATAAAGACCGCCCGTGTGATGAGTTGGTGA[C/T]GTGGTGGTGATGACTTGTCTTTGGGGATTATTAATGAGCTATTTGTTTTGTTTCTGGTGTGTTTTGGTTTCGAAAAAAGGGGGTTTGTGCTTGTCTTTTTT |
| comp105449_c3_seq2:522-523 | comp105449 | 522 | T | C | 116 | 0.181034 | 0.818965 | 377 | 0.790451 | 0.209549 | TTGTTCGTCCGGCATCGTTTTCTCGCTGTAGAATTTCGTATTCGTTATAACCAATAGACATTTCGTTTTAGCCTGACTCTTTGCATGTATGTATATATATA[T/C]TAACCTTTCTCTCTCCATCGGAAAGTCGTTCTAGTAATCAGCAATAACAGGTAGTATCGTATAGACTTGAATTAACTTTATAAGATTTTTTTTTCTTCTGA |
| comp107929_c1_seq12:2095-2096 | comp107929 | 2095 | G | C | 271 | 0.852399 | 0.147601 | 662 | 0.243202 | 0.756798 | CCGACCGACGTGGCTGCCGTGTACGAGGCCACGGCGCAGTACCTGGTGGCCGCTTTCCTGCCGGACAGCACGGCGGAGGCGGTGGCGAGGCTGGCCGAGTC[G/C]CTCTACTACACGCCGAAGGCTAGGCGGTCCCTCAACGCCCTGGCGGAGGAGATGACGGAGGTTCTGACGGACTATCTCTTCTTGTCCTGCGTGTGGGACAC |
| comp107792_c0_seq2:583-584 | comp107792 | 583 | T | A | 57 | 0.736842 | 0.263158 | 47 | 0.12766 | 0.87234 | CGAGGACTTCTGCTGATGGCTCGGCCTGGCGTCGCCGCCCACTGTAATGCGAATGTTGTCTGGGAATATAGAATAAATGTTTTAGCCTGCGTTTGTACACG[T/A]CATTGCCCTGTAATATATACACTTGATCACTAAGTTGGACTTTTTTCTGTTGGACATTTCCCTTTCCCTTTCCCTGCTGCCGGGAAATTAAGAATGAAAAC |
| comp107136_c0_seq2:723-724 | comp107136 | 723 | C | T | 304 | 0.842105 | 0.157895 | 249 | 0.232932 | 0.767068 | CTTGGCTCCACCGCAGGGCCTTCAACAGCAGCAGTAGAAACCAGCGCCTCGGGGACATTAAACTGAAAAAGATCCTCTGGGATGCAGAAAAAATGAAAGAC[C/T]TGCCTCAGGCCAACTTCTCCGACCTCCTGGCAAACGACAAGGCCCTCCTCAGGTTCCTGCAGGAGGTGGAGGAGCTCGGGCTGTGCATCGTGAAGGAAGCT |
| comp107470_c0_seq1:600-601 | comp107470 | 600 | T | C | 113 | 0.327434 | 0.672566 | 63 | 0.936508 | 0.0634921 | CCGGAGGCCAAAGTGGTCAAGGGCTTCAACGTGCTGTCGGCCTATGCGCTCGAGAACGGCGGACTCCAGGGCAGTAAAGAGGTCTTCATCGCCGGCGACAA[T/C]GGCGAGGCGAAGGCGAGCGTGGCGGAGGTGGCTCGCGTGATGGGCTTCCACCCGGTCGACTGGGGCAGCCTGCAGGCCTCCAGGGACATCGAGGACGTGCC |
| comp106223_c0_seq2:422-423 | comp106223 | 422 | C | T | 56 | 0.714286 | 0.285714 | 19 | 0.105263 | 0.894737 | AACATTCTCAGTCGCCGCAACTCGAGGACGTCCACGACCGAATCCCCCGAAGAACCGACGACCACGACCCCAGCCCCCGCTGCCGCCGAAGCCGCCTCCGA[C/T]GCCCCCTCCCACTCCACCAGGCGGCTGCCGCCTCTGCGCCGCCCGGGCATTCGCTTCGGCCTCCGCCCCGGCTCGTCGCTCAAGAACAAGCTAGAGCAGCA |
| comp107146_c1_seq2:1854-1855 | comp107146 | 1854 | G | A | 52 | 0.942308 | 0.0576923 | 12 | 0.333333 | 0.666667 | CTGCACCTCTGTTGTTCTTCTTGGGGTGGAGAAGATGGCTGAGGTCCAGGGCATCGGCCTTCTGCTTGTTCATCCTGTTCTTGTTGTTCTTGTTCTCCAGC[G/A]CGACGGCTTTCACGAAGGACGACGGTCCGCCTTTCCTGGAGGAGGAGGAGGTCTTCCCGGAGGATCCAGAGTTCTTGACCCCTGGCTGGACCTTGTTGGCT |
| comp106322_c1_seq2:177-178 | comp106322 | 177 | T | C | 57 | 0.912281 | 0.0877193 | 89 | 0.303371 | 0.696629 | TTTCTGTCTCCTCTCAACTTTGTTCGTCTTTTCCCTTGATTCATTTCACTTCTCCGATGGTTAAGGATCGCTATGATTCTCAAAATCCACAATTGGAAACT[T/C]ATCGCTCAAAGAAGACTAGATTCTATTTGCAGCGTAGGTGAAAAGGAGGAAATATTCCCTTCATCTATGTATTTACGTACTTTTCCTAGATTTCCAATCGC |
| comp101001_c2_seq6:1786-1787 | comp101001 | 1786 | G | A | 29 | 0.827586 | 0.172414 | 64 | 0.21875 | 0.78125 | TATATCTTCAAGTGCATTTTAGTTTCCAATTAAAATAGCATATTTGATTACCATTACTCTCTAGTCTCTTTAAAGACTCGTTACTGCTGATCTGGGGACTA[G/A]TTTACTACTTTAATTTCAAGGGAGATTTTTAGGGTTGTCGTTTTCTCTAGTTCAAGTAAACATCACGGGAGACTAAAGGAAGTTAGCTACGTTTCAGCAAG |
| comp106959_c0_seq1:335-336 | comp106959 | 335 | A | T | 565 | 0.893805 | 0.106195 | 256 | 0.285156 | 0.714844 | CACCCTTTTTTGTAACATTTCAGAATATTTGTAAGATGGACGCGTCGCCAAGGAAGCCCGTGGCAATCCTAGCAGTAGATGGCATTGGGTCGACCTCCGTG[A/T]TCCGGCGCCTTCGGCCTTCAGCCACGGCTTCTTGATCTCGACCTCGTACTTGCAGCCTCCTCAGCGCCTGTGTCGGATCTCGCCCTTCCTCGAGAGCCTCT |
| comp107193_c0_seq1:774-775 | comp107193 | 774 | T | C | 175 | 0.668571 | 0.331429 | 100 | 0.06 | 0.94 | GCACCTACTTGTACGAGAGAAGAGCTCGATATAACGATGGATTTTTGGACAGTCTTTCGTATGTTCTTCCTGGTTTCCAAGATGGCGCAGGGGGTCACCTC[T/C]TATCTGGTTGCAGTGAAGAGGTTCCAGAGGTTGCAATACCCGGAGCATTACGAGCACCTGCGGGGGCGACACTCCCGGCAGGGCAGACTCAACATCGTGGC |
| comp104160_c0_seq2:1517-1518 | comp104160 | 1517 | G | A | 88 | 0.875 | 0.125 | 45 | 0.266667 | 0.733333 | TGGATGGATTCGAGAATATGAAGAGGCTCATTATTTCCATGGCCATCAAAAATGTAAATCTTGGGGCTCTCACTATCCGAAACAGCTAATGTGTGTAGCGG[G/A]TCAGATGAAGGATGGATCCACTCGCAAGTGTTGGGCACAAAACTCAATTTGATCATGTTGATCATATCAAAATTCTCTACATCGAAAACTTTCAGAGTCTT |
| comp107475_c0_seq1:1263-1264 | comp107475 | 1263 | T | C | 73 | 0.191781 | 0.808219 | 25 | 0.8 | 0.2 | ATTCACTTTATAATATTCAATTGCATACTTTTCTATTTCCCTTATCTACTTATAAAGTTGCTTTAATCATTGCCATATATAACACAGAGCAAGGAAAAAGC[T/C]CACCTAAACACTGGTTCATCACTCCTTCATTCCATTATTTCTTCACAAAAACAAAGAAAATCTAAGATGATTTAACAAAACTTCAAATTTCAGTTAGTCTG |
| comp104149_c0_seq1:2961-2962 | comp104149 | 2961 | C | T | 125 | 0.656 | 0.344 | 125 | 0.048 | 0.952 | TATTTCAGTCCTCTCCCAGAGAGCATACCTGGATCTCAAGCTCTTCTGACAAGTGTGGGAGAGTTCCTTGCTCTACACTTCTGTGACTCACGCATTGTGTA[C/T]GCAGACAGCAAAGACTCGCTCATTCAAGCTCTGGCAGGATTTGTATGCCACCAGACAACCCTTACAGCCTTAGAGATGATGCCTCTGGAAAGCCGACGACA |
| comp105944_c0_seq3:330-331 | comp105944 | 330 | G | A | 58 | 0.758621 | 0.241379 | 73 | 0.150685 | 0.849315 | ATAATTTTAATTATATATATTTACAGTAATTTGGATTAGATGTAGTCTAGAGTTATGCATGCGTCATGGAAAAATGTAGTTTAAAGTCAAGTTGTTTTTCA[G/A]TTACAAATAAGTTTGTCTTTCTTCAATCCATACTAAATATGGTTTAGTTTAGTACATAATAGTAAAATGCAGATAAATGTTAATTATTTTTTATAGATTTT |
| comp104092_c0_seq1:1298-1299 | comp104092 | 1298 | A | G | 83 | 0.710843 | 0.289157 | 68 | 0.102941 | 0.897059 | CAAAGAGGAGTAAGGCATCATCAGAAGCCTTGAAAATGCCCCCAATTACTAAATTTACATCAGAAACAACAAGAGAAAAGAGTTGGGACAGTCTTGCAGCA[A/G]TTCACCGTGGCACACCAATTGTTACAACATGGTCAGTAGACAAACAGAAAATGGGAGAGCACAAGTTGTTGCATGAACGCTTTAAGGACAAGACCATACGT |
| comp106753_c2_seq2:1289-1290 | comp106753 | 1289 | C | T | 370 | 0.843243 | 0.156757 | 582 | 0.235395 | 0.764605 | GGCGGGATGGTGTAACTGGCTCTGCCACTAATTATATACATCTTTAAGGGCATCATTGTTATTTTAGCACAATTATTAACACTGCATTCAGTAGCTCAACA[C/T]CATCAGCTCTAAAAAGTCTGACGCACTAGCCAGAAGTTCAAGGATTTGATTTGATAAGTTCATTACGTCAAGGTTTGGAGAACTTCACAAAGGAGGAGATG |
| comp104783_c0_seq1:520-521 | comp104783 | 520 | T | C | 51 | 0.941176 | 0.0588235 | 6 | 0.333333 | 0.666667 | TTAATACATAAAAGAAAAGAAAATGAAATATTTACAATACCAAAGCTATAATTGGAGTTCTAAGAGAATAATATTCTGATTCTTGCATAATGCAGTCCTTT[T/C]GGCAATGGCAACGCGATATCACTGCCATCTACGTGCTGAAGTTGACAACAATCTGTATATTCAATAAAGAAAAAAAAAATTCAACAGGCTAGGAACTAAAC |
| comp105019_c2_seq1:1233-1234 | comp105019 | 1233 | A | T | 63 | 0.333333 | 0.666667 | 68 | 0.941176 | 0.0588235 | ACAGCAGTTTAAACAAACGGGATCTGGAAACGCCATCAAAAAGACCCGGATGGAAGTAAATGAAACGAAGCACGTACAGTTAAAGGCGTGTTTCCAAGGAC[A/T]CAATCTCGAACACAAGGAAAAAATGCTATAGACAAGGCCAAGGACATATTCGTCACGAAATCACATAATACAAGAGGCGGCCGGATGTTGGCGGCTTGATT |
| comp102059_c5_seq1:2934-2935 | comp102059 | 2934 | T | C | 185 | 0.67027 | 0.32973 | 112 | 0.0625 | 0.9375 | GATACTTTGAGGGAGATGTTCGGAATCCATGCGTACACAGGCTTCACCATGGATAAAGTGGTTACTAGTGCTGTAAGGCAGTTACAACACCTGGTGTGTGA[T/C]GACCCCCCAGTCCAGTGCACCGCCATGTACCTGAGTGAGGCAAAGAAAGGTGCTGCTGGCGGACCTTTAGCCTCTGCACACAGACGGCTCGCAGCAGAACA |
| comp105085_c0_seq1:1076-1077 | comp105085 | 1076 | A | G | 101 | 0.70297 | 0.29703 | 105 | 0.0952381 | 0.904762 | TCCACAGACAGCTCATTACTTCTCTCTTCCAGAACTCTTAGGGTCTGGTCCTTGTCGATGAACACCAGATTGCTGTGCACGTTCCACTCTTCCTCTGAATC[A/G]TGTCTTGTCTGGCGGGGAATGAAGGCCGCGCATGTGACTTTTGCAGGGTTGATGTTCCGTTTCCAACTGATGGGCTCACTGCTCTTGGGTTCGAAGATAAA |
| comp103361_c2_seq17:2943-2944 | comp103361 | 2943 | C | A | 101 | 0.920792 | 0.0792079 | 99 | 0.313131 | 0.686869 | ACCCAGTTAAGCTCGATCCCTTGATGACTGCCAAAGACTTACTGACAAGATTCATCGAGGAACTGACCGTCCTTCCCGAGAACCCCCGTCGCGTGGAGGGC[C/A]GGAGAGAACAGCAGTACAAGCGAGGGAGCGGAGGCAACGGGTCCCCTCCTTCGACCGCCCCCTCTAGCGTCTCCTCGCCCCAGTTCTACGCCTGCCTCCTG |
| comp107162_c0_seq9:328-329 | comp107162 | 328 | C | T | 102 | 0.852941 | 0.147059 | 106 | 0.245283 | 0.754717 | GTCATCTGACAAATATTAAATTTTGGTTTATTCCAATTTAAATATATGTTATAAACTCATGTGTGGAAATTAAATAAGTGAAGCAGTAATTTAAAATATGA[C/T]CGAAGGATTGGAGAACATGTGTCCTTACCATGTTAGTATATGTTATTTTACACAAGAAATAGTAGGGTTAGGTTGTGTTAAGTGTTCATGTAGAAATGTAG |
| comp96498_c2_seq1:491-492 | comp96498 | 491 | A | T | 55 | 0.745455 | 0.254545 | 29 | 0.137931 | 0.862069 | CAAATTGCGGCTCATCCTCCAGCACTTTCTTAGACATTCCTTATTCATCTTGTATTAAGTATTCTTTAGACCTTGCAAGTGTATCCTCACAATGCTTAATA[A/T]TGTTTGAAAATCACTTTGAAACCAATATCAGGATCCACTTTGTACTGAGACAAAAAGTAGTTAAAATTATCCATTATTCATCACTAAATAGAATTCTACTC |
| comp106513_c0_seq4:167-168 | comp106513 | 167 | A | T | 64 | 0.3125 | 0.6875 | 25 | 0.92 | 0.08 | ATAAACTGGGCCGAGCGACATAAACCTTCCGTGTTAGGCAAATGTTGAAGGAAATGCATTTTCTGCCTTATCAGTGTTATCAATTACTGATAACAGACGAA[A/T]AGTCTTCTACGAACTGTTGCTAGAGCGAATGCGTCGACGTGGAACCGAGAGACATTTCTTAGATTCCGATCCATTTAGTAAAATTTCCGCTTCTTCCTTCG |
| comp107892_c0_seq1:525-526 | comp107892 | 525 | G | A | 54 | 0.907407 | 0.0925926 | 130 | 0.3 | 0.7 | ACAAAAAAATCCTCCAATTTCCCATACACGAAAACATACAGAACCACCCATACCCACCAGACCGACGACCCGAACATAAAACAAACACCCACATTAATAAC[G/A]GACGAAGACTTACATGCGCAACACATTTATTTAACTTCTGAGAACCCAAAACGCGAAACGAACCTGTTCAGAACCTCCCGAGCGGAAAGGAGTAAACACAG |
| comp103241_c1_seq3:686-687 | comp103241 | 686 | G | A | 158 | 0.721519 | 0.278481 | 175 | 0.114286 | 0.885714 | CCCAAATTCAACTAATGGTGTCCTACATCACCTAAGAATGTTTATTTAAGTCCAAATAATTTCATGCTGTGACAAGTAAAACAGTCTTTGTGATTGTAGAT[G/A]TTAGTTAACTATGACATCTCAGACAGGACCAGTAAGGAAACTTTCAGGACAGACAATACTAGAGTGAATTATGGTTCACTTGCAGGAATATATCAGTATTT |
| comp107115_c1_seq2:135-136 | comp107115 | 135 | A | C | 164 | 0.859756 | 0.140244 | 198 | 0.252525 | 0.747475 | TCGCGACGCAAGTACAGGAGCCACTTCTCCAAGACGTCTCTGGGCACCAAGTCGGCCTTGTTCAACACCAGCACCAGCTTCTTATTGGCATGGCAAGTGAC[A/C]GAGGCCTCCATTTGCGGCACTCTGGAGCCCAGGGGGTCACGGGCATCAAGAACCTCTAGGATGACATCTGCTGCCTCCACCACCTTCTTGAACTCCTTGTA |
| comp106995_c0_seq1:1173-1174 | comp106995 | 1173 | C | T | 63 | 0.904762 | 0.0952381 | 84 | 0.297619 | 0.702381 | CACAGATCCCTTGACGGGCCATCTGAGCAGATTCCGGCGATGGACCAGACGAAGGACTTGGTCAAGAGCGCGAATTTCTCAGATTTCGCAGCCCCAATCGC[C/T]ATGATGTACAGCAGTTGGGAGACGGATAAAATCATCGCATTTGAACTTGTCCGAAACCTTTCTCTGGCGCTGGTGGCCGTGTTCGTCATGACGCTCATCCT |
| comp105501_c0_seq1:903-904 | comp105501 | 903 | T | C | 164 | 0.658537 | 0.341463 | 136 | 0.0514706 | 0.948529 | GTCTGGTCATAGCCTTTTGGCTGGAAGATGGGATCCCATCCAAACTTGTGCTCCCCTCGAGGCTCCACAATGGTACCTTGGGTGCGACCATGGAACAAAAG[T/C]ACCTCGTCATTGGGTTCTCCAGATGAGTAAGCAAATGTGCACACAGCTTCAGCAGACTTATCTTCAAAACCTGCTAAGAGCTTTGTCAGGCCACCTGGCCC |
| comp107794_c0_seq2:549-550 | comp107794 | 549 | A | C | 867 | 0.612457 | 0.387543 | 553 | 0.00542495 | 0.994575 | ACTTTGAAGCCCAAGGGACTGTAACGCCTACATTCCATGGTCAATTTGTCAGTTCATTCGACCAGGTTCATGGTTATTAAATCACTTACGATGTAGCATAT[A/C]CATAGTCATTAAATTAATTGCAGTGTGGCAGATCCATGGTTATTGAATTACAGTGTAGCAAATCCATGGTCGTTAGAATGCTTTGCGTGGCAAATCCATTT |
| comp107558_c0_seq2:1200-1201 | comp107558 | 1200 | C | T | 157 | 0.707006 | 0.292994 | 10 | 0.1 | 0.9 | TATTTCCCGCTCTTTTCCTTCAGTCTATGCAGTTCAATCGCATTATTTTATGTCTCCTTACGTTCATATACGATGTTTCTCTAACTGTTTGTAGGCCTACC[C/T]GTGTGCACAAATACCTTTTCACACATAGCATTAACGCACTATTATACCAGCGGGAATAAAGGTATATATATTGTTTATTTAGACACGATACACGTACTGCA |
| comp96843_c0_seq1:454-455 | comp96843 | 454 | T | C | 269 | 0.657993 | 0.342007 | 254 | 0.0511811 | 0.948819 | ATGGTGAGGTTCCTGGTGGAGCATGGTGCTTGCATCCTCGCGACCACGCTGTCTGACCACGAGACTGCAGCCGAGAAGTGCGAGCAGGATGAGGAGGGATA[T/C]GACGGATGTTCCCAATATCTATACAGCGTTCAGGAGAAGCTCGGCATAATGAACGGCGGCGTCGTCTTCGGTGTCTACGACTACGAAGGGCAGGCGGGAGA |
| comp107898_c0_seq1:5532-5533 | comp107898 | 5532 | C | G | 138 | 0.702899 | 0.297101 | 52 | 0.0961538 | 0.903846 | GTTGAGAAAGGGTTGGAAGGGTGTTGAGAAGGAGGAGGAGGAGGAAGGAAGAAGGATGGATGACAATAAAGATGAAGAGAACGATGGAAATAGACGAAAAA[C/G]AGAGAGAGAGAGGTTTGAAGCAGGTTCGAATCTCGGATTGAGAAGGTGCGAATAACTCTGAGGGGCGAATAACGGTCCCTTTGAGGTGCGAATAAGACGAG |
| comp107771_c1_seq1:2091-2092 | comp107771 | 2091 | G | T | 509 | 0.856582 | 0.143418 | 140 | 0.25 | 0.75 | AAAAGGTTCGGTTTTGTGGGCGTACCCCTAGTGGCAGTCAGGCGAAAGATGTTGTTCAGTGCCCCTTCGACGTCTTGAGTCCTGTAGTAATCTAGAAATTC[G/T]TTGGCCACCTTGAGTTTGTCTTTGATCTCTATAGCATCTGTGATCTGAGAGAGTGTACTCTTCAGTTTCATGTCTACGTTTTCGATGGAATTTTTATAGTC |
| comp105365_c0_seq2:1086-1087 | comp105365 | 1086 | G | A | 73 | 0.821918 | 0.178082 | 65 | 0.215385 | 0.784615 | AGATTAAAGAGAGTCCCAACAAAGGTAACTTGAGAATAGAGCAAGTCATTGGTAATGATAAATCTAAACTTGATTTCAGTGTGAGTGCCAATCAAGTTAAT[G/A]TTTGTCATGATCAGAGTGATGATCAGGTAAGTGAAAATGAAGAAGAGGGAAATGAAGAGGACTCGTCTGATCAAGATGAGAATAGTGATGTTGATGAGGAT |
| comp107900_c0_seq2:654-655 | comp107900 | 654 | G | A | 50 | 0.66 | 0.34 | 56 | 0.0535714 | 0.946429 | ACTGCAATGTACATTTTTTGCCCTTTGTGTTTCTGTGCAGCTGTTAATGTGGCCGTTCACTGTGAAATGGTTTGAAATTATAGATTAGCTTCAAGTATTTT[G/A]ATTTTATACTATTAAGTCAAGGAAAAGTTTTAGCACTTGATGACCAGTGCTTATTCTTTGGAATCTTATTTATGCATCAAGTTGGAACAATGATATCAAGA |
| comp107495_c0_seq2:2021-2022 | comp107495 | 2021 | A | G | 77 | 0.922078 | 0.0779221 | 38 | 0.315789 | 0.684211 | AACAAATCAGCTGTTGAAACGATCGTGGAGAGCATGTGGTGGGAAGCGGGTTTCGGTTCCACGAAGAGGCACGAAGAGCAGCCTTGTAGTAACTTCAGGGA[A/G]CTTATCAAACTTTACCCTTCAATAGCCGAGAAAGTGATGAACAAGTGCACAACCACTTGCCCAACAACGAGGTTCAAGTCCTACGATTTCAGAATATTCGA |
| comp105764_c2_seq8:445-446 | comp105764 | 445 | G | T | 149 | 0.939597 | 0.0604027 | 36 | 0.333333 | 0.666667 | GATATCCGAGTGGAGTGACTTGGATAACAAAATGACTGACGGACAAGAAAATGCCAGTGACGATGAGAGAACGAAAGAGAGAAAGAAGAAAGAAGAAAAAT[G/T]ACACAAAATAAGACGAAAAAAGCGACACAGATTGGAGGACAAGAAAATAGATAAACAGAAGGAGAGCGCAAAGCAAACAGAAAAATAAATACATTAAATAT |
| comp107911_c4_seq1:105-106 | comp107911 | 105 | A | T | 32 | 0.78125 | 0.21875 | 80 | 0.175 | 0.825 | TTTTGTGAGATGCAGTATTTATTCAGTAGAGGGGAAATCATTTATTCATGAGAAAGAGAGAAAAAAAACTGTTGTAACATTTTCTTTAATTTCAATCTGAT[A/T]ACTGTACGCATCCACAATGGATTTCTTTATATATGGTAATGGAAAATAAATAAATAGATAAAATAATGGGATCAGAAAGCAGATGTGGATACAGAAAAACG |
| comp93629_c0_seq1:530-531 | comp93629 | 530 | G | C | 52 | 0.865385 | 0.134615 | 27 | 0.259259 | 0.740741 | ATTTCTCACAATACTCAGGAGGGACGACGGCGCGCCCTTCGAGCAGCAGGAGGAGACGCCGCGGCCGCAGAAGATCCGCGTGCAGAGCTTCAGCCACGTGA[G/C]CGAGTTCGGATGGGGCTTCCCGGAGGTGGACGAGGCCGCGGCCAACAGGAGGAAGGTCGACCTGAGCTGGATCCAGGGCTACGACCGGGAGATCGGACGCC |
| comp106667_c0_seq1:431-432 | comp106667 | 431 | T | G | 131 | 0.709924 | 0.290076 | 144 | 0.104167 | 0.895833 | ACAGTGGCTGTGGTAAGTCCTCCAGTGCTCGTCGTCGTGTTTGCTGTCGATCACGTATCTGACGAACTCGAGGAAGGTCGGACCTACAGGACTCGCGAACG[T/G]ATTCCCCTTCAGGACCACCGGGAGTCCGGCCATCACCTTGTTCCGGACTTGCTCTTGGAGGAGGCCGAGCTGTCCCTCGGAGATGTACTTGGGGAACTCCC |
| comp101775_c0_seq4:425-426 | comp101775 | 425 | C | A | 106 | 0.613208 | 0.386792 | 133 | 0.0075188 | 0.992481 | GGGAAGCACTGACGAGGATGATGGCATTGACTTCGACGAACCAAAAGATGATGGGATCGTCATCATGATCGAGCAGGTGGTGGGCGGCCCCCGTGCTGCCG[C/A]ACAAACAGCTGAGACTGCCCTTGGAGAGGGAAAACGAAGCGCCAAGTCGGATGGAAGAAAATAGCGAAGAAGAAATGGAAGAGAATGAGATTACTTAATAT |
| comp106630_c0_seq9:172-173 | comp106630 | 172 | G | T | 113 | 0.920354 | 0.079646 | 108 | 0.314815 | 0.685185 | CTTGAACAGATGATCGTAACAAGCAGTTAGGTTGAGTAATATACACCTATCTAGGTAAATGGATAATAATTCTATTTACTGGTTAACTATGTTGTAAAGGA[G/T]AATAGGTAGTAGAGAATCGATTAAATAATTCGAATAACTTAAATTATAACGATATGAAGACCGGTAAGTAATTAACCACATTGGCAGCACACAGATAAAAT |
| comp101564_c1_seq9:337-338 | comp101564 | 337 | C | A | 599 | 0.75626 | 0.24374 | 378 | 0.150794 | 0.849206 | TTCTTATCCCCTTTCTTTACAATCTCTTCCTCCTCCTCTTCCTCTTCGTCCTCCTCCGACAGATCGTCCTCTTCCTCCTCGTCGAACTTTTTCTTCTTTTT[C/A]TCCTTCTTCTGTTTCTTCCCCAGCTTCGCCTTCATGCTCATCGTCATCATCATCTCGTCTCCCTCGTCCTCGCTCTCCTCGTCACGAACCCCGAGCCCGAT |
| comp103828_c2_seq5:2137-2138 | comp103828 | 2137 | T | A | 282 | 0.79078 | 0.20922 | 232 | 0.185345 | 0.814655 | GTAGCTGTGTGTTCCATCTCCTTCAGCTGACTGTCCAGGAATTCCTCCAAGGTCTCCATTTCCTTCTTTGCAATTGACAACTTGTTCTCTGCCTTGTCCAA[T/A]GCTTCCATCAGTGACGACAAAGAGGTTCCCAGACGCTGCTCCTCTTCCTCAAGGTCATTGTCATCCATATCAAGAAGTGTGTCCTTTTTCAATAGATTCTT |
| comp98792_c2_seq2:663-664 | comp98792 | 663 | G | C | 63 | 0.777778 | 0.222222 | 58 | 0.172414 | 0.827586 | CCGCGCCTTGCAGCGTCTCGGAGAAGTGGAAGAGGCGTTTCTTGCGGGCAAACCGACGGGGAGACGAGGGTGATGAGACGTGCGCCGCGGGGAGGAGCGTC[G/C]AGTGGCGCCGGCGTGGTGGGCCTCGGAACAGGGCGAGGTGGGTCAGATGAGGGTGGGTCGGCCGAGGGTGGCGAGGGCCCCACGACCACGACGCCGGAGTG |
| comp99598_c1_seq10:435-436 | comp99598 | 435 | G | A | 56 | 0.339286 | 0.660714 | 54 | 0.944444 | 0.0555556 | ATGACTCTGTCTGTGTCTCTGGCAAGAGAGGCTGTTAATGAGTACTGTAGGGAGAATCAGAAGGAAGGAGAGAAGCCACTAGTTGTTGGTTCAGTTGGACC[G/A]TATGGTGCTTGCCAGGCTGATGGGTCAGAGTACACTGGAGATTACGTATCAAAAATGACAGAAGAACAGCTGAAGGTGTGGCACAGACCTCGCATGACTGC |
| comp94799_c1_seq1:634-635 | comp94799 | 634 | C | G | 119 | 0.605042 | 0.394958 | 1 | 0 | 0 | TTGTCCTTACGGCAATGCGCAGTACACGAAGCGTAGGCTGAGAAGACAAACCGTTGTTGAGTGTTGTGGGTTGTTTAGGTTCTAGTCATGAAGTTTGGTCT[C/G]GCGATGAGGCTCCTCCTCGCCGCCTGCGCTCTGGCGCTGCCCCGTCTCTCTACCTCGACCCCCTTGGCCTCCCCCGCCGCTGAGCCCCTGCCCGGCCCTGC |
| comp97713_c1_seq1:1461-1462 | comp97713 | 1461 | G | A | 195 | 0.68718 | 0.312821 | 146 | 0.0821918 | 0.917808 | GCTTCCCGTCTTGTAGCCACTCATTTCTCTGAGGGTCTCCCAGAACCTGCACTTGCCCTAATAGTCAGGGAGATACTGCAAGGACTCAGCTATTTGCATTC[G/A]AAGAATATTATCCACAGAGCTGTACGAGGAAGCCACATTCTGATCGACAGTAATGGTCGTGTTATACTTACTGGTTTGCGTCACAGTGTCATGGTGGGAGA |
| comp92406_c0_seq3:179-180 | comp92406 | 179 | T | G | 69 | 0.695652 | 0.304348 | 44 | 0.0909091 | 0.909091 | TGGGCGAGCATTCTAACCCTGTCCATAAGAGCCACACAGGAGTAACTGCTTCTGTTGTTGGCTGCAGACAGCCCGCCTCCGCCCGCGAGCGAGAATGACGT[T/G]TTTGTTCAATGTTTTCCTCCTCCAGCCAAGTTCCGAAGTGCTCTCGCCTGGCTTTCCTAGTTTGGCGCCAGCTGGTCGCCCTCGCTTCTTCAGGTCCTTGG |
| comp103147_c1_seq5:1480-1481 | comp103147 | 1480 | T | G | 53 | 0.735849 | 0.264151 | 61 | 0.131148 | 0.868852 | GCATTCTGTTTTTGTAAGTTTTCCAAATCTGCTGATGATCTGATTTTTGCTGCGGTACACTCAATACCACTATACTTGTTAATACTCAAAACATTTTCTTT[T/G]ACTTTATCATAATTTGCAGTATAAAGAACGCTCATGTCAGGCAGCTTGGTCTTTTGCACACTGTAGACATGTACTGACATCACATCTTTTAATTTGGACTT |
| comp103742_c0_seq2:246-247 | comp103742 | 246 | T | C | 38 | 0.710526 | 0.289474 | 85 | 0.105882 | 0.894118 | TGCAAATAGAACATTGGCTAGCAGAAGAGACTGAGTAACGAGGGAGTTAATAATGCATAAGTAAATAAAGAAGTGTATTTGCATTTTTTGTCAGAATTAAT[T/C]TAAATCTCTACCATTAGGGGGCCTTTTGTGAATGCCCAAAGTCTCATAAATGATCAAATATACATAGAGGAATAAATGTTATATAATAATTATCATTAAAA |
| comp100181_c0_seq2:1695-1696 | comp100181 | 1695 | G | C | 470 | 0.814894 | 0.185106 | 390 | 0.210256 | 0.789744 | TTGCTCCTGCCTGACCGTGGGAAAGACGGTGGTCGGGAGTACGAGCCCCCAAGGCTGGAGAATTCCCTCGGCAAGTTGCAGGTTGTTAGCGTTAATGCTCC[G/C]AGAAAGATCATTGACCTGCAAGTTGTGCACCTTGACCCGTCCCACCCTACGACCCCACTACAGCGTGAAATGAGGAAACATAGACATCTCCTTCTACACAT |
| comp105570_c0_seq14:274-275 | comp105570 | 274 | A | G | 84 | 0.678571 | 0.321429 | 54 | 0.0740741 | 0.925926 | TTGGCGACCATCAGGTTGACGGCCAGGCCCTCGCTCCAGCTCTGCCACACCTGAGGAGCGGTAAGAAACGGCAGTTCATCCACCCAGTAACTGCTCATTAT[A/G]GCATCTTTCACTCCTCGTTCGACCACATTGCTCGTTCCTGGCCCCGGGTAGAGGACGTCAAAGCACACCTGAAGCGTAAACGTGACCCCGAAATCGGTCGT |
| comp107673_c2_seq1:495-496 | comp107673 | 495 | A | C | 2564 | 0.162246 | 0.837754 | 420 | 0.766667 | 0.233333 | ATGATGACCAGATTCTGCTGATTCAGCACCGACAGGTAGTTGTCCTCGTTGGTCTCGGATGTGGCCATCATCTGGCCGACGGCGTCGGCCTTCTCGCTGGC[A/C]TGCTGGACGCCCAGGGCGGCCACCTTCAGGTTCTCCTCCTCGTCCTTGATCTGAAGGATGAGCTGCGCGAAGGACACCACCTTCTCGCTGAACCTCTTCCC |
| comp85895_c0_seq1:623-624 | comp85895 | 623 | G | A | 74 | 0.864865 | 0.135135 | 215 | 0.260465 | 0.739535 | TAAGCTGCAGTTGCGGATCCACATTCGATCGTCTTGGGGGCGTTGACGAAGGACCCGATGGGCTGCCCTTCGGCGTTGAAAGCCTCGATGAAGAATCCTTC[G/A]AACGTCACGTTGGGGTCGAAGCCGGTCAAGATCACGTTGATCTGCGAGCCGGACGGGATCTCCTCGTTGGGCACCATCAGGAGGTAGGGAGCCTCCGACGT |
| comp104451_c0_seq5:969-970 | comp104451 | 969 | G | A | 115 | 0.704348 | 0.295652 | 10 | 0.1 | 0.9 | GAAACCGAGATTCTATTTCCTTTATTTGAGCTTGAATCAAGTCCTGGTGCCATCTTTCAACGTAGGCGAGCTAGATCCTGCGATTTTTCGAGCCAATGAAG[G/A]GGTATCGTTTGTGATCGCCTTTGAATTTTGTTAAACGTTTGTATATCACCAATAATAACGTTTTGAGTAAACATGGATACGCCTTTCCAAGAAAGTTGAAT |
| comp106958_c1_seq1:1676-1677 | comp106958 | 1676 | T | C | 62 | 0.33871 | 0.66129 | 35 | 0.942857 | 0.0571429 | TCATGCAACACCGTGTACTCAAAACCCAAAGAACACACAAAATAACAGGCCTCAGGCAAAACGATCACTGAAGTATTTCAGATATCAAATGTCTAGGCATC[T/C]TATAGCACCGCAACCAGGCAGATTTTTTTAAAGGTCTGTGCGTTATACGGCTGGCATATTCTAAACTATGCAACAGGGTATATGTAAAAAAGGGGGAAAAT |
| comp106412_c0_seq3:1377-1378 | comp106412 | 1377 | A | G | 58 | 0.844828 | 0.155172 | 54 | 0.240741 | 0.759259 | ATTCATCCGGCGCATATAAGCGGGTACCATCTGATGAACTGCCCACAAGAGTCAGGGTTCCCTCATACGTCTTGTCTTGCAACGCTACTCCATTGGCAATA[A/G]TCTGACACTCCTTTTGGACTGCTGTGCTCACTTCCCTTGCCTCGCCCTCACTGAAGTCAACACTGAAGTCACTACAGTCATCCACGATGTCACTCAAGCCC |
| comp107015_c0_seq1:1300-1301 | comp107015 | 1300 | T | C | 89 | 0.640449 | 0.359551 | 55 | 0.0363636 | 0.963636 | TTTGAGAAAACGTTTTTCCTGATTAAACGCACTGTTTCCTTAAGAATTATGAGACAATTATTTTTTTTCTAGATAAACATTAATAAAATTAATACTGTTTT[T/C]TCGTTATAATTGGCTATACTGCGTCCAGTTTTTGGGAAAAAAATAATAACAAGCACCTCACCCCTGTCTGGTGTTCGGATCCATGAATTCTCTTTTTGGTC |
| comp105673_c2_seq2:290-291 | comp105673 | 290 | G | A | 73 | 0.917808 | 0.0821918 | 51 | 0.313726 | 0.686275 | TATGTAAAAGCTAAAAAAGAAATTCGTGGCCAAGTTAAGCCTTTGTATCATGTAGTCTTTGAGTAATAGGGAATCGTGGCTGCTGTGTTTTGTTTTTTCTT[G/A]TGAGGCTTTCGAGAAGGAAATGGCGAATCATGTCTACTACAAGTTTTTTTTTTCTGCAGCCCTCTGTAGATAAAGAATGGATAGATAGAACACTATGTTCA |
| comp96428_c0_seq2:191-192 | comp96428 | 191 | A | C | 134 | 0.865672 | 0.134328 | 126 | 0.261905 | 0.738095 | TCTTACCTATACAACTCATTTAAGGCAATTACAAACGAAAAGAGGCCGTTACACAATCCGGATAGATGACCCGGCTACCGACTACGTAACTCGCCAGAGAC[A/C]TATTCAATCCCTCTAATTTGTCCTGAAATACGTATTTGTAAAGTGACATAAATGATCGAGAAAATTATATAGCCTAAGAAATGCCAATACGTTTGTATAAC |
| comp107368_c0_seq1:1966-1967 | comp107368 | 1966 | C | T | 56 | 0.803571 | 0.196429 | 10 | 0.2 | 0.8 | TGGCCCCTCGCCCCCTATCATGAGGAAGACAGGACCTCCAGGCCTGTAGAAGGAGCCATTGCTGAAGTATTGCTGCTTCCATGTCCTGCCTTCGGTGGGGT[C/T]GAAATGGTCCAGCTTCTGCAGGAACCACTGGGCCTCCGGGAGCTCGGTGCCCTCAGGACGTCGAGGGGGCGTCAGAAGTCCCCCTCCTCGTCGACGTCTTC |
| comp107445_c2_seq2:179-180 | comp107445 | 179 | G | T | 63 | 0.825397 | 0.174603 | 9 | 0.222222 | 0.777778 | CATCTATATTCCCCTTTTTTTCTATATCTTGAGAAAAGAAAAAAAAATCCGCTACATCTACCCTTCCTGTAATCTAAGGATCCCAATAAATGACACAATCT[G/T]CACAGCGTTCAGGTTATGGCACCGCTACTTGACTAGAATCCGAACGGCTGTGGTGTTGTGTGCGTAGAGTTTAATACCACTTCACTAACACAATGAAAAAA |
| comp106202_c0_seq1:1049-1050 | comp106202 | 1049 | A | G | 17 | 0.941176 | 0.0588235 | 71 | 0.338028 | 0.661972 | TCTGCATATACTTGATATGCCGTTCTTCCACTGTGTCTAGCTGGTGCCATAGACGTGGGAAAACCAAGCTGCGTGTATATAAGGGATATGGAACGATAATT[A/G]TACACTACTGGTACATTTCTCAGGATGGATGTCTTACGCCTCGCGCTCATTCTTGTACTTGCGGCTGCCCCAGGGACTCTCGCCTGCTCCTTCGGCTTCAC |
| comp106449_c1_seq5:465-466 | comp106449 | 465 | T | C | 224 | 0.84375 | 0.15625 | 187 | 0.240642 | 0.759358 | GAGGAGAAGAGAGTTCGCACAGAAGAAAACTCCGCAATGGAAGAATATAGGAAAAAAGTATCAGAAATGCAGAAACATGCTGCAGAGGAAGAGATCAGAGC[T/C]GAGATGAAGGCTGCAGAAGCACGTAAAAATGCCGGGGGATCAAATAAGAAGGCCTCACACCTTTCTCTCTTAGCTGGGGCAATCAAGAGGAAGTCCAGCGA |
| comp107414_c1_seq1:1495-1496 | comp107414 | 1495 | C | A | 81 | 0.925926 | 0.0740741 | 65 | 0.323077 | 0.676923 | AGCTCAAAGACAAGATTTATTATCGTCTCACACACAAAGAAGAGGTCTGTGGTGTCCAGTGGAAGAGATACAGCGAACTAAACGAACTGTTACTAGGTCAC[C/A]GGCCGGGAGAACTGACGGTACTCACAGGTCCCACAGGCTCAGGGAAAACCACATTCATGGCCGAATATTCTTTGGACTTGTGTTTGCAAGGGGTGAAAACC |
| comp98673_c0_seq4:223-224 | comp98673 | 223 | A | G | 75 | 0.106667 | 0.893333 | 55 | 0.709091 | 0.290909 | TTCTCAAGATGTTTAAGAAAACTTCTTTGATCAACATATGTGCAGGTATGATAGTCCTAAGATTAACAAATCTCGGATTTTGCTTTTTCTATTTCTTAGCC[A/G]CATGTGGATATTCACCTTCTTGGGTATGTTGTGGATATTCGCCTTGGGTATGTTGCGGATATACCCCTGGTTGAGGATGCTGTGGGTATGCTTCTAGCTGG |
| comp102007_c0_seq1:610-611 | comp102007 | 610 | C | T | 47 | 0.914894 | 0.0851064 | 96 | 0.3125 | 0.6875 | TCACATAAACGAAGATTTTATGGGCATCACACTTAGCAAATACCCACATGCAAACATATAAAACCCCACAGTAGTAACACACACATTATAATTTACATCAC[C/T]GTGAACCTACACAAAGACAACTTTCTCTTGTACTAATGGCACATATTAACAATTATAAAAAATAGCAGGCATATGACTTGTAGCATATATGCACCCTGGAT |
| comp102915_c0_seq2:458-459 | comp102915 | 458 | G | A | 45 | 0.644444 | 0.355556 | 71 | 0.0422535 | 0.957747 | CCGGCCGCGGCCTCGAGGGCCGCCGAGGACGCCACCAAGGTGACCGAGCCGCAGGGAGGCCTGCAGAACTACGAGTACCTCACCTTCGAGGAGGCCGACGA[G/A]CTTCAGAAGCCGGAGGTGGACATCGTTCCGAACGCGGAGGCCGAGGAGGGGGCCCCCGCGGCCGCCAGCGTCCCCGCCCCTGGCCTCGCCGCCATGACCAC |
| comp103897_c0_seq11:139-140 | comp103897 | 139 | A | G | 60 | 0.333333 | 0.666667 | 62 | 0.935484 | 0.0645161 | AGATTTGAGTTTACATATAAATACCATTAACATTTATTCTTACAACAAACCTCTGAAAATCTTCTACACGTTTTGAATCACCAAACCACTCGTCAAGTTTC[A/G]TAATGCATTTAATAATCAGTTATTTTTTGTCATGGAGTGTGCATTTTTCCTTAATTGTATATATACACGTATTCTCATCATATGCCGACGTTTCACATTTT |
| comp99161_c0_seq2:1565-1566 | comp99161 | 1565 | C | T | 74 | 0.837838 | 0.162162 | 89 | 0.235955 | 0.764045 | CAAAGTTCTAACAGTCACGTTTCACAGTGATTCAGAAAAATGTCTTTCTGAGAGTCAATGGCTTATAATGACCTTGCCTAAGCACTATCAGGCTCTACACA[C/T]GGAAGTCCATTTGGCATCAGAGGTACCAGAGGCGGGTACAGAACTGCAGTCCTGTGCTGTCCTGACACATTGGCAGTTGATGAGAACGTTTTAGCAATTAA |
| comp104655_c0_seq14:1226-1227 | comp104655 | 1226 | C | A | 63 | 0.873016 | 0.126984 | 59 | 0.271186 | 0.728814 | ACGGAATCGGTCTTGACCTGGACCACGTTATCTAGGCCTAAGCCCATGGTGACGGCGGCTTTCCTGATCGAGTAGTGGCTCTGGTCTGAAGTGAAGGCGAC[C/A]AGAGGCTTCAGGCTAAATATTCCAGACTGCTTGATTTCAGGGTGAAGTTTGTATCTCGCCAACAGCATGGCGTGTAAGTTGCTTATGCTGCCACCCGGAGC |
| comp107541_c2_seq3:244-245 | comp107541 | 244 | T | G | 227 | 0.903084 | 0.0969163 | 312 | 0.301282 | 0.698718 | GTTTTTACATTTTTTTACAACTCTCGGGAAATATACTGGTTAAAATGTACACAAATGTAGGTGTGGTTTGTATTAATCTGCCTATATCTGTCCTGAATTTT[T/G]AACTAATGTAACACAGAATAACTATTGTAGTTTCCCCTTAATATACGCCAAGAATGTAATAGGTACCTTTGTTTTTTTTCTTATCTGATTATCTTACATCA |
| comp107653_c0_seq11:3404-3405 | comp107653 | 3404 | G | A | 72 | 0.944444 | 0.0555556 | 70 | 0.342857 | 0.657143 | GGAAACAAATAATTTAACTTTTGTGAAGTTGAAAACAAGTCTGTTTGAAAATCAAAACTTCACAGTAACAGTATAAACCTACCTGAATGTAACCAGTGCAC[G/A]AGATAAGAAAAATAATTTTGCATCCATTCATAGCAAAGCAGAAATACCATTGTCCAAAGTAAGAGGTCTACAAAAGGTGGGAAAGTAAAAGCAATTTAGTA |
| comp104227_c0_seq7:217-218 | comp104227 | 217 | A | G | 215 | 0.934884 | 0.0651163 | 6 | 0.333333 | 0.666667 | GTTTAAGTGAAAGGTTATTGAGCTTGGGATTACGAATCTAAAATTGGAGTGAAATTTGGCTTACGGTTGGGTAAATTTTGGGTTTAAATGGTTTTGTTTCT[A/G]TGGAAGTTTAAGGGAAAAGTTTTTGTGCGTGGGGTTACGAGCCTAAAATCGGAATGAAAATTGGTGAAGTAAGACAAATCTTAAGTTCTTTTCCTGGTCGT |
| comp107329_c0_seq2:1315-1316 | comp107329 | 1315 | C | T | 105 | 0.92381 | 0.0761905 | 93 | 0.322581 | 0.677419 | AACAGACTATTAAACTGGATGTGCTTTTGTTTCTTTGGCATACATTTTAGTAACTCCAAAGGAGTGAGGAAACGATTTAACCTTCTTGTACTGCTTATGAA[C/T]AGTACCTTTGGCTTGAAATGTCCAAAGGTCAATTCAGATATAACAAGAATTCATAGCTTAAGTAAGTCCACAAACTTAAAATTCACTAGTTTCTGATTTGA |
| comp106036_c0_seq13:1329-1330 | comp106036 | 1329 | T | A | 51 | 0.803922 | 0.196078 | 69 | 0.202899 | 0.797101 | TATTTAATTTCCTTGTGGATGGAACCAAAGAGGATTTACATTTGCATAAACATCGGATTTCATCAAGAATCAAAACACATTTTCTAATATTCAAAAAACAT[T/A]TTTTTGTTCGTATAACAAAGTGCAAATACAACTCCCTTATTTTATTACACATGTAATAAGTCTGAAAACACAACAGAAGGAAAGCGAAGATATGGTCGATA |
| comp105181_c0_seq1:1013-1014 | comp105181 | 1013 | A | G | 115 | 0.669565 | 0.330435 | 102 | 0.0686275 | 0.931373 | CATATTATATATAAATATATTATCCTTTACACATGATTGCCCTAGTCTTAAATCAACATAACCATCAAGGTTTGAGTAATTCTCCACAAACCAATAGATGC[A/G]TTTATTGTGTGTACAGACTGGGAGTGAAGACAATGTCTCTCTTGATGGCTGTTGATGCTTCTTCCATTTTCTGCATGAGACTCGGGTCACCAATGATCCTG |
| comp102850_c1_seq6:246-247 | comp102850 | 246 | T | C | 203 | 0.788177 | 0.211823 | 64 | 0.1875 | 0.8125 | GAAGTAGGTGGTTGACATGTGTCAAATTTGGAATATGAGAAATGCGAATGGCACCTAAGAATGAAAAGTAAGAAAAAGATAAGAATGAATGATAACCTCGA[T/C]AGAGCAGTAGAAAAGAAGGAATTGAAAGTCTAAATAAGAACCTGGCTGAAAATGGATGAAGGGTGAATGAGTGAAAGCATGGATAATGGAATACAGAGATT |
| comp106490_c0_seq8:1940-1941 | comp106490 | 1940 | C | A | 229 | 0.80786 | 0.19214 | 164 | 0.207317 | 0.792683 | GATATGAGCAGAATTGTGGATGATGATAATGATGACAAGAAGAAAAACAAAAATAAGAACAAGAACAAGAACAAGAATAAGAATAAGGGAGAAAGTGACAC[C/A]GTGGGAACTGCTAAGATTGATGATAATGATGACTTCGATATTTTGGACAATGCTGGTTTGGAAGAAGTGCGACTCATGAAATTCTCCGACTCTGAAGATGA |
| comp101797_c0_seq3:937-938 | comp101797 | 937 | T | C | 247 | 0.765182 | 0.234818 | 170 | 0.164706 | 0.835294 | TTCATCACATCATATTTGTCTGCTACATTCTCAAAAACTTCATGTACTTTTTGGGCCTTCTGGTTCTCAGGCACGTTTTCAAAGCCAAAATGTGTTTCCTT[T/C]TCCGTAGAATATTCATCCTGAGCGTGACCCGATGCACTAGACTTAAACCTGTTTCCCCGAGAGAATAATATTCGCGAAACCGACGGTATTCTTCTGCACAG |
| comp80161_c0_seq1:352-353 | comp80161 | 352 | G | C | 91 | 0.725275 | 0.274725 | 24 | 0.125 | 0.875 | AAATCCTGATATAATTATTAACGTTATTAATTATTATCTTTACACTGCCCTTCGCTAATTGGGCAAGAGACATAACACTGTTGAACATATATTCACATGTG[G/C]AACTGGAATATACACAGGTAATTGTTGAAAATACATCGCATGAGAACTATCATACCATACAGCCTGAGAAATGTGTAACAGGATATGAGATCTGGCTTGAT |
| comp102936_c0_seq15:1225-1226 | comp102936 | 1225 | A | G | 2270 | 0.6 | 0.4 | 1 | 0 | 0 | CGAACCAGTGACGAAGAGCTGGAACGCATAGCGGAGGGTATGAGGGACAGGATCCAGGCTACCAGTGGTGCAGAATACACCGTTACCAGAACTCCAGAATC[A/G]ATCATCGTCGGAGGAACCTCGGAGAACTGGGTGGCGAGCCTCGGCGTCCCTTACGTGTACACCATGGAGCTCCGAGACAGGGGCGAGACCGTCTTCCACCT |
| comp104912_c0_seq1:2718-2719 | comp104912 | 2718 | A | T | 60 | 0.6 | 0.4 | 2 | 0 | 0 | GAGACCCCCACATGCAATGGCTTGGCTATGTCTGGTGTAGTCTATGCCCCTGTTCTGGGAACACATGACATCCACTCACGAATGTGCAAACCTGCTTTTGT[A/T]GAGGTGACCACCAACAAGCAAGGTGCCGATAGAAAATACATCGCCAGGATCGGTCTTCAGGCACCAGCTAATGCGGAAATTAGTCTGAGTGAGGGAACAAC |
| comp96032_c3_seq3:105-106 | comp96032 | 105 | A | G | 120 | 0.6 | 0.4 | 1 | 0 | 0 | GAAGGAGAGAGGTGGAGAGATATGAAGGGAAGAAGAGGAAGAGAAAAAAAGAGAGATAGTGGGAACAAGAGGGAAGGAAGAAGGAGAGTGATGGAGGGAAG[A/G]AAACGGAGACAAAGAGAGACGGATAGAGGAAGAGACATGAAAGGAAATATAGATACACAGAAAGTTTGATAGGTATGTATGTGTATATACATACCTATATA |
| comp98749_c0_seq5:786-787 | comp98749 | 786 | G | T | 55 | 0.6 | 0.4 | 1 | 0 | 0 | GACCAGCAGGACGTTCTGCTGCTCCTGTCGTGGCTGCTCGGGGAGGCTAGCGACGACCCCTCCTGTCTGGAACGCGTCGTCTGCCTCACGCCGGCCAAGTC[G/T]TCCCGATACATCTACGTGTCGTCCATGATTTTCAAGGTCCTTCACTTCTTCCAGAGCTGGCTTCCGTACAGCCGCCGAGTCGAGGACCTGCTGCTGCGGCT |
| comp104269_c3_seq66:2309-2310 | comp104269 | 2309 | C | A | 71 | 0.788732 | 0.211268 | 90 | 0.188889 | 0.811111 | CTGGTCCCCTGAACCACGGCTTCGATACCTTCTTCGGAATCCCAGTGACTCTCTTCTTTGAGTTCCGAGGACCTTACGCCTTCTGGAAGTTCGACTTCAGC[C/A]AGCCCTCCTACCAGGTCAGGCTCCCTGGCCTCTCGCTTTTGCCATGGGGACTGTGGGAGTGATTCATCACGTTTTGCTGATGGCGGGATATTTACAGGAAT |
| comp96417_c0_seq2:551-552 | comp96417 | 551 | G | A | 956 | 0.933054 | 0.0669456 | 147 | 0.333333 | 0.666667 | GGAGGAAGGGCGGGAGGACTCAGCCAGCAAGGAGCAACGGCAGGAGGACGCGGGGGAAGAGCAGGAGGAGGACTCGGCCAGCAGGGAACGACAGCAGGACT[G/A]GACCAGCAGGGAACAACATCAGGACTCGGCCAGCAGGGAACAACAGCAGGACTCGGCCAACAGGGAGCAACAGCAGGATTCGGGCAGCAAGGAGCAATGGG |
| comp90303_c0_seq1:621-622 | comp90303 | 621 | C | T | 1149 | 0.896432 | 0.103568 | 1431 | 0.296995 | 0.703005 | AGTTCAATCTGAGCCCCAGTTGCTCCACCAGCCTCGTCCAGGCGCTCATTAAGATCAGTAAGTTCGCGGTGCATGTTATTCTTGGCTTTCTCGACTTTGGA[C/T]CGAGCCTGGCGTTCATGCTCAGCTTCAGTTTCCAATTCTTCAATACGAGCTTGTGCCTCTTTGATAGTCTTCTGCACCCTCGAGACTACCCCCTGTTCCTC |
| comp100479_c0_seq3:233-234 | comp100479 | 233 | C | T | 242 | 0.619835 | 0.380165 | 49 | 0.0204082 | 0.979592 | ATATTTTTTTTTAGTAATTATATTGGATATTCCTCTCTATTTTGACTTTTCATCTAAATGGAAAAAATGATCAAACACTTGTATGTATTTAGTTTTTTTTG[C/T]AGTTTAATATAATATGGGAATTGGAAGTTTAACTTAGCTTTTTAGAGAGAAAGCAAGAAAGGTGCTGTTTTACAAAGGTGTTTTATTCAAGCACGCATTAT |
| comp99364_c0_seq1:340-341 | comp99364 | 340 | T | C | 945 | 0.753439 | 0.246561 | 597 | 0.154104 | 0.845896 | CCCAATTTCCTTCAGTTTTTAAGCATGACAATGGACGATTACAACGAGCACCACACAGGATTCCAGTTCGGCAACTTCGCCAGAAATGAGAAGCTAGCAGC[T/C]AAGGGCTTCCCCATGCCCCGGGCCCGCAAGACTGGTACCACCATTGCAGGGATTGTGTTCAAGGATGGCGTGGTGTTGGGGGCAGATACAAGAGCCACAGA |
| comp106939_c0_seq2:1322-1323 | comp106939 | 1322 | G | C | 142 | 0.633803 | 0.366197 | 29 | 0.0344828 | 0.965517 | TTGAGGGAGACGCTGTAGTTCCCGGGGGAGTTGGAGGGGGCGGGGACCACCTCGTAGACGTCGGGGGCGAGGCGGACGGCGCGCCCCTCGAACACTTCAAA[G/C]AGCGTGGCTGAGGACACGTTCACGTACCCCTCCTCGTCCAACCCAACAGCTGTGATGTAGACATGACCCACACTCGCAGGCACGTCAACGGGCTGTACACA |
| comp104118_c1_seq4:403-404 | comp104118 | 403 | T | A | 303 | 0.874587 | 0.125413 | 316 | 0.275316 | 0.724684 | ACTAGAATTAGAGATACTGGGATATTTAGGAAGAATATTGCCAACTTACATGACATACTTAAGAATCTCAGTTTGAAGTGATTATTATTTTCCAAATTAAA[T/A]ACAATTTTGTAATCAATTTTTCTTTCTTAAGGAAATAACCAAATGAGAGAGATATTCACAGTGAAAGTATAAACATCATGTAACACATTCTAATTCCGATG |
| comp94488_c0_seq2:1034-1035 | comp94488 | 1034 | G | T | 381 | 0.83727 | 0.16273 | 21 | 0.238095 | 0.761905 | TAGTAATGCTTTTTTACAAATAGCCAAAGAAGTAAATCCAGCAAATAAAAGGATTGACACGCAGCTCTGTTGAATATAGCATTCAAGGGATGGTGAATATA[G/T]GATTATTTCTGAGCCCCAGAGAACCATAGATCACCAGCACGCCAAACGCTGCTGTTGCCACCTGCTCACGAACAGCTAAGATGTTTTAAAGCTGCGCAGTT |
| comp102941_c3_seq12:315-316 | comp102941 | 315 | A | T | 366 | 0.748634 | 0.251366 | 622 | 0.149518 | 0.850482 | TACTTGTTATACAAATCAACGACTGCGTTCACATAAGTCTCGTGCAACTTCACGATCTGTTCCTTTGTGGGATTTTTGACGAGGGGAACGTCAATGGGGGC[A/T]CCAACCACTGTATATATGGGCTTGCGGAATGGCACAATACCAAAGCTGTACTGGAACACTCCTCTGCCAATGAACATGCATGGGGCCAGACCTATCATCTT |
| comp103529_c0_seq1:693-694 | comp103529 | 693 | G | A | 1018 | 0.82613 | 0.17387 | 740 | 0.227027 | 0.772973 | GTCTACACGCAGAATGCTGAGATGCGCCCCTTAGGATGCTGCATGATACTTATAGGCTATGATGATGAGCTTGGACCCTGTGTTTACAAGACAGATCCTGC[G/A]GGCTACTACTGTGGGAACCGTGCATGCTCTGCTGGGGTCAAACAGACAGAGGCTAACAGCTTTTTAGAAAAGAAGTACCGCAAAAAGACAAATTACTCCCA |
| comp102283_c0_seq3:119-120 | comp102283 | 119 | T | C | 173 | 0.705202 | 0.294798 | 179 | 0.106145 | 0.893855 | TTGAGTTTGTAATAAATACAGGTAAACATAGAGGGACTTACAGTACTTGGTGTATGATTTTTTGTGGTTTTCAATCTCTTATATGGGATCTGACTGTTACT[T/C]TGGAAAATACCAAGGATACGCGTCAAGGTTTTCCAAAGTGCCACTGAAAACTCTAAAGACGAAAGTTCTCAGGCTTTATTTTTGTCAATTTTGTAACAGGA |
| comp106665_c1_seq1:1138-1139 | comp106665 | 1138 | G | A | 22 | 0.909091 | 0.0909091 | 58 | 0.310345 | 0.689655 | GAGGAGGAATGCGGCGTGAATGAGTGCGAGAGAAACAACGGCGGCTGCACCCACTTGTGCGTCGACTCCAGGGAGAGTTACCACTGCGAGTGTAGGACGGG[G/A]TATCGCCTGGTCGGAAAGTACACGTGTGAAGATATTGACGAGTGCCTCGAGGTCCCAGGAACGTGCTCCCAGCAGTGCACCAACACCAACGGAGGTTACCA |
| comp100003_c0_seq1:940-941 | comp100003 | 940 | A | C | 28 | 0.892857 | 0.107143 | 51 | 0.294118 | 0.705882 | GGTGGCAGGCGGTGTGATCTTCAACCTTTTGGCTTGAAGCACGAACTAATCATCTCCGACGACCTTTCAACTATACCTTATCGTGATATGAAGAACAACTG[A/C]ACAAAACCATGAGTACACGTATGGGCCAATCTTTTCTCTTTCCTTTCTTCTTCCCCCATATATGTGTTAAGCAAACCGTTCTTACGTTGGTTTTGCTGGTT |
| comp97082_c0_seq3:380-381 | comp97082 | 380 | C | A | 86 | 0.77907 | 0.22093 | 72 | 0.180556 | 0.819444 | CATGCCTTGGTGTCATCGAATCTTTTCTCAGCACCAACAACATGATTGGCTAAACCAATTTGTGAAGCTTTAATGCCATTCAATTTACTACAGGTGGTTAG[C/A]AGATCAAGAGCAATAGTAGGGCCTACAAGCTTCACCAGCCTCGTTCCACCACCCCAACCTGTCACCACCCCCATACGACCCTGGACGAAACTTACTTCCCC |
| comp105917_c0_seq3:2069-2070 | comp105917 | 2069 | A | G | 79 | 0.78481 | 0.21519 | 59 | 0.186441 | 0.813559 | CATGTCAAAGAGCAGGAAGTCTCCCGTCTCACATGCTGTGACCATCATCTTGTTGCGCTTGTGGAATGCTGTTGCCGTCACTGAGGTACTCATGCCCTTTA[A/G]GTGATTGTTCACATTGAGCTTATAGCGACGAGTGTAGAATACTCTATCTTTACTTTGGTCTTCTTTCCCTTCTTCCTTCTTCACTTTCTTCTCTGCCTTCA |
| comp107889_c1_seq2:1984-1985 | comp107889 | 1984 | C | T | 68 | 0.926471 | 0.0735294 | 67 | 0.328358 | 0.671642 | AAAGTTCTAAAAGCTAAATGGCTCAGGTCAGGAAGCACAAAACTTTCAGTCCTTCTGGCAATGACTTTCATATCCACCTGGAACTTTTCTGACTCAGAATA[C/T]CCACAGAATACGCAGTGTTCATCAGCTTCAGGGAAGCACTGGCAAAGAGAGATTCCCTCCTTGGTGGCTGCAAATTTCTCACAGTGTTCATTGAGCATTGT |
| comp93238_c0_seq1:600-601 | comp93238 | 600 | G | T | 1500 | 0.825333 | 0.174667 | 22 | 0.227273 | 0.772727 | GGAGCAGGGCGGCTGTACACGGCCTCGTACGTCACCTTCGGCACGGTCACCAGCTTCCATTCCTGCACGTGGTGCACGACCGGCTTGTGGTGCACGATGAT[G/T]GGCTTCTGCACGACGACGGGCCGGTGCTCGACGAAGGGCTTGTGCACCAGGACGGGCTTGTGCACGACGTGGGGCACGTGCTCGAGGAAGGGCTTGTGGAT |
| comp102234_c1_seq1:675-676 | comp102234 | 675 | T | G | 193 | 0.756477 | 0.243523 | 82 | 0.158537 | 0.841463 | GTTTTCCAGCACGCTGTCAACGGAGCCGCCAGAGGGATCGCCGGCTGTGGCAGATCCGGTTAAAAAATGATGTAGTTTCATAAGGAATGTGATTTTTTGTC[T/G]TTTTTGTTTTCTTTCTTTCTGTTTTTTATTGTTTCCTATTCTTGTTTTCTTTTTCTTTTTATTCCTTTTTCTCTTTATATTTCCTTGGCTTTCGTTGCGTT |
| comp102781_c0_seq1:200-201 | comp102781 | 200 | T | A | 51 | 0.0392157 | 0.960784 | 157 | 0.636943 | 0.363057 | TACGAAATTAAATATTAATGCTTCATCACGAAGGTTTGACCTCAATGTGGAAAAAAGAAAAGAAAAAAAACAGGTCATTAAAAAAAAAAAAAAGGTTTTCA[T/A]GTCTGCAACTCACTGACACTCATATACTACACACTTTCCATTGTGATAGTCTATATAGTAGTTACGAAGAACAAGTTATGGAGAACAAACGCTAGCGTGTT |
| comp104413_c0_seq6:371-372 | comp104413 | 371 | C | A | 1252 | 0.748403 | 0.251597 | 2979 | 0.150722 | 0.849278 | GCCGGTCGGTACTGCTGACGGCCAAGGCAGTGGGCGTCGATCTCAACCTAAAGCTGCTCGACCTCATGACGAAAGAGCAGATGAAGCCCGAGTTCGTGGCC[C/A]TCAATCCCCAGCACAGCGTTCCTACCATGGTCGACGGGGACCTGAAGCTGTGGGAGAGCCGCGCCATCTGCACCTACCTGGCATCCAAATACGGCAAGGAC |
| comp101755_c1_seq5:754-755 | comp101755 | 754 | A | G | 422 | 0.829384 | 0.170616 | 164 | 0.231707 | 0.768293 | GCTCCAGTTGTTGTAGTTGAGTGGCACTGTGCCATAGGTTCTGGAGGGCCACAGGAACGCGCCGTTGATGTCTTTCGTGCCAGCTGTCCAGATCCAGGCCA[A/G]GTTATGGCCTCCGACGATCTGCGACAGAAGCTGATCCTCCTGCGGCGTCTCAATGCTGACGCCGTCCCATCCGGGTCCGAGGCTGGTGCAGTAGAGGATGG |
| comp100823_c1_seq2:116-117 | comp100823 | 116 | C | T | 79 | 0.696203 | 0.303797 | 81 | 0.0987654 | 0.901235 | GCCGGGAAAGGCGACGTGTGTGAAGGGAAACCCTCCTTTCTCAACACTGGATTTACTTTCTCTGATCGTTGTATTTCCACAGTGTTCATCTGCTGACATAT[C/T]TGAATCGTTGTTGATTCGTTCCACATCGCCAGCATGAATGACGACGACGATCCTAAGGAATATCGGTGGCAGAGTGGCTACGAGAAGACATGGGAGGCGAT |
| comp100218_c0_seq2:1544-1545 | comp100218 | 1544 | C | T | 101 | 0.930693 | 0.0693069 | 93 | 0.333333 | 0.666667 | ACCTGCGCCCTCATCTTCCAGGTCGCCTTCGCCCTGGGCATCTCCCCGATCTCCTGGATCTACATCGGCGAGCTCTTCCCGCTCAAGCACCGCGGCCTCGG[C/T]GCCATCGCCAACTCCGTCAGCTACGCCTGCTCCTTCGCGAGCGTCAAGACCTTCGTGGACTTCCACCTGCTGCTGGGGCTCCACGGGGCCTTCTGGCTGTA |
| comp106159_c0_seq4:411-412 | comp106159 | 411 | A | G | 51 | 0.333333 | 0.666667 | 101 | 0.930693 | 0.0693069 | TTACTCTACTTAAACGGGTGATTTTACGTGATTTTCCTGTTAAAAGTTATATAGCTATTTTTAGATGCATACCACATATCGTTCGAAATCAGAATTGTGAT[A/G]GAATGACCGGAGGATAAACGAATATTTAATTAGTCCAATAATCTTAGATGGTTTGTGTCTGAGCTGCTCAATCCTAGTTCATTATATTACAATAGATTTTT |
| comp106389_c0_seq13:292-293 | comp106389 | 292 | C | T | 61 | 0.901639 | 0.0983607 | 23 | 0.304348 | 0.695652 | TGTGTATTATACAAATGAACATATCAATCAAATGTTCTAATGTCCAAGAAAGTATTGGCAAAATTCACACAATAATTATGGCAAAATCATGTTAAATCTAA[C/T]GAGATTCCTCATTCATACAAAGTTAGCACACAGAATAAGAAAAAAAAAATAGATTCTACAGGGTGAGTATTCTACAAGCTATGCTTTTTAAATAACAAAAT |
| comp101991_c1_seq2:403-404 | comp101991 | 403 | G | A | 67 | 0.910448 | 0.0895522 | 83 | 0.313253 | 0.686747 | AAGTATGTAAATATCAATCTTTTTCTCTCATTTTTAAATTTCATTTTAAGGGGAGGAGGGGGTAAAGAATAAGAGAAAAAATAATAAACCTCCAAAATATA[G/A]GTATGCAAATTAAACGTGTTTAAACATATCAGTGATTGGAATGTGTTATTTACCCCATGAATTTACAAAAATGGATTATGTGAAAAATCAGACCCTATATC |
| comp106910_c0_seq3:497-498 | comp106910 | 497 | C | A | 29 | 0.310345 | 0.689655 | 54 | 0.907407 | 0.0925926 | GGATTCAAAACTGGAACTTAAATACTTTAAAATATATCATAGGGAAAAACAATAACTTTCATTGTTATACATAGATTCAATTACCAAATTTCTAAAAATAT[C/A]ATTTCTGCATGAAAATGTTGTAAGGTGAAATGTGTAAAAAGATCATGCATTTTTTCATAATTACAGAGACCTTCTGAAGCTGCAATTAAGACATCATGTTC |
| comp106546_c0_seq1:199-200 | comp106546 | 199 | A | T | 135 | 0.933333 | 0.0666667 | 113 | 0.336283 | 0.663717 | CAAAATAATGTCTCAAAATAATAGGGAGCAATATAAAACAACAGCACACTGAAAAATCTCCATATATAAAACATTTTCCTGACAAATACAGACAAAAACAT[A/T]TGCAACCTTTGCAACCATTTAGTTTGCAACCAAACTAAATATCCTCTACCAAAACATAAAACCATCACTAAGATTATATACATACTAAACTGACTATTACG |
| comp106655_c0_seq2:948-949 | comp106655 | 948 | G | T | 86 | 0.930233 | 0.0697674 | 18 | 0.333333 | 0.666667 | ACTTGGATTACGTGCAGAAAGTCCGTCAGATGGTTCCCCTGGGTTCCCAAAGGAGGGGCGATATGTACGAAGTTGTAACCAAGAAGTAACTACTCTTCAAT[G/T]TAAAAGGAATGAATGCTATATAATGTCTTACTAATCCAGTGCTCAATGCAATGATTATTTTTTCATTTTGACATCATTTATATAGAGATACAATGCTGAAT |
| comp104864_c0_seq2:876-877 | comp104864 | 876 | G | A | 137 | 0.846715 | 0.153285 | 88 | 0.25 | 0.75 | ATACTAATTTAAGAAAATCTTGTTTTCCTTTTATTTTCCTCTCTCGCGCTGCAAGATTTTAATAGAATCTGGTGTTTCCACAGGATGAAAAATATTAATTA[G/A]TCAATTTTTATTTTACTGTCCGGATCAATTGTAAGAGAATGCAGCTAGTACAGTATTCTTTTGGAATGTTTCAACACTGACAGTAGTTCTATGATAATCTG |
| comp97443_c0_seq1:342-343 | comp97443 | 342 | G | C | 752 | 0.867021 | 0.132979 | 355 | 0.270423 | 0.729577 | ACGGCGTCCCTGCTCTCGCCGGTGATGTAGTAGATCTGCTTCTGGTCCTCCTTCATGCGGGACACGTAGTCCTTGAGGGAGGACATCTCGTCACCGGAGGC[G/C]GACGTGTAGAAGCGCAGGAGTTCGGCGAGCTTCTTGCGGTTGGTCGAGTCCTCGTGGATACCCAGCTTGATGTTCTTGGAGAAGTTGTCATAGAACTTCTT |
| comp105783_c0_seq1:263-264 | comp105783 | 263 | C | T | 296 | 0.939189 | 0.0608108 | 280 | 0.342857 | 0.657143 | GTAATTGAAAATTTAGTTGTTATCCAAGTAATATAGAACACAAGACTTAAAATAAATTTGTTAAACATATGGAGAAGTTTATAACAGGGACACTGTTTATA[C/T]TTTTTAAAAAAGAATTCCCACACCTCTACATCAATTACTCACAAAATACATTTTGGACACTTGGTATGCTCTTGTATATCTCCAAATTATTAATTCAACAC |
| comp104192_c0_seq9:1045-1046 | comp104192 | 1045 | G | C | 52 | 0.846154 | 0.153846 | 8 | 0.25 | 0.75 | TGGTGCAAGACGAACTGCCTCATGTACCCCCCCAACTGCGACCCGACGATCTGTGTCTGTGTGAACGAATGCGAAGCTATTGGGGAGTTCGCCAAGCAACC[G/C]GGGGCAGATATTTACTGCCACCAGAACTGCCTGAAGAACCCCCCCGTTTGCCCCAAAGATCGGTGCAAATGCTTCTAAGGGAGATTCGATCGGTTATTGTT |
| comp105096_c0_seq2:2593-2594 | comp105096 | 2593 | G | C | 76 | 0.75 | 0.25 | 39 | 0.153846 | 0.846154 | CATGCACTTGAGGACGACGCGCTCCCCCTCCGGCACCGTGACGTTGGAGGGCTGGACGATGAACTCGGACGGGGGCGAGGGGCCGCCGTGACGAAGGGCGT[G/C]TCCGCTCCAGGGATGCCACGTCGCTAGGCACGCGACTAGTGTAATTTGGATGCTGTTGCTGACCCTCATCTTCACTCCGGTTCCTTGTACTTGTACTGCGC |
| comp103082_c0_seq1:2856-2857 | comp103082 | 2856 | C | T | 699 | 0.0686695 | 0.93133 | 969 | 0.664603 | 0.335397 | AAAAAAAGAGAAAATAATAAAAGATAAAATAACGAGCTATGATAAATCCTTTCGGAATTTGAGAAGTCACGCAGAGATAGAGGAAATGATACTTTGATGTA[C/T]CTTTTGATTTCGTAATTAATTACAGGGAAAAAGAGTGAAGAGAAGATGGCGAAAAAGAAGGGAAGAGAAAAGAAATGTAGTAATAATAACCTTTGAGCCTT |
| comp100936_c0_seq21:188-189 | comp100936 | 188 | T | C | 328 | 0.60061 | 0.39939 | 202 | 0.00495049 | 0.995049 | ATATGTGTGCATGTGTATGTATCCTATTATGCAGGAAGAGCAGACGGGATATGAACATGACATTCTAGAAGGTAACATTATATTGCATCTGTATATACACA[T/C]GCGGACACACATGTATACATGCATAAAGACACAAACTTAACATTGCGTTACATTGTGCAGTAATATTAAATCAAGATACAAATAATACATAGGAAGTTTGA |
| comp98330_c2_seq1:417-418 | comp98330 | 417 | C | T | 75 | 0.706667 | 0.293333 | 18 | 0.111111 | 0.888889 | CGAAGCAGCACTTGGAACAGAAGCGAACCGCACTTACCACTCAAAGAGCAGAATTGAGCGCCAGTGTAGACCAAGTGACACGAGAAAAGGAGCAACTTCTA[C/T]TAAGCATTGAAACACTCAACATAGATATGGACCAAATTGAATTTAATCTCAGGAAAGTAAGACAGGATCTGGCTACCTGTGATCGCTTGAGTGATATGGAA |
| comp96450_c0_seq2:395-396 | comp96450 | 395 | T | A | 140 | 0.878571 | 0.121429 | 106 | 0.283019 | 0.716981 | TTTACGGCTCCAATTTCCTGACACACTGCCATGCGAGACAATGTGGTGACGTAGGAATGGCCGGGGCTAAACTCAAGTACTGTGTGGCACTTTGTAACTTT[T/A]GTGAATGTTTGTTGTACAGTTGTGGAAGGTAAAGTGGCATAGCCATTTGACGGTGAATCCTTGTCGAAACTAAATAACGTTATTATATAAGAATTAGTATA |
| comp105494_c2_seq1:669-670 | comp105494 | 669 | A | G | 291 | 0.721649 | 0.278351 | 222 | 0.126126 | 0.873874 | AAGAGGCAATAACCTTTTATTAGTTGTGTATATATAGCTTCTAAATAATTCTCTGATGAAAAGTATCTTTAGGTGATTGTAATGCGAAATGGGTCTGTTAT[A/G]TACTTGTCAATCTAAGTTGAACTCATACACTGAGGAAAGTTCCACAATAAAATATGAAGAAGTGAATTCACTTGTATAACCAGTAGTCAACATGTAGAGGA |
| comp106735_c1_seq12:4287-4288 | comp106735 | 4287 | T | A | 346 | 0.887283 | 0.112717 | 353 | 0.291785 | 0.708215 | ATCAGTTTATCATTTTGTACTTATTTGTTTTTAAGTGTCTTCAAGGCTTTGGATGCATAAATTCCAGATGATAAAATCTGAATCTGAAGACGCCATTCCTG[T/A]CCTCTACTTGTGGTTGTCGAGGTTCTGGCATCCCTAAGTCCAGGCTCACTGCCCTGCAGCTGCACTATCATCAGCAGCACTCATCTTGAGCACACTGCACA |
| comp96289_c1_seq4:301-302 | comp96289 | 301 | A | G | 101 | 0.881188 | 0.118812 | 84 | 0.285714 | 0.714286 | AGTAGAGTAGCATCCATAGGTGGCATGAGTGGACACTTCCGCTTATTTGTTCTTGATGTACCCAGAGCTTGTCGATATTCTGTACTTGCTGATTCTGGATT[A/G]GCAACACTAGCTAATACTTGTTGTCCTCTTACACGCATAAATACATTCTTCTTTAAGGCAGGCCTTGTTGATACGACTGGAACATGTCTCTCCCTCAGGAA |
| comp106049_c0_seq2:232-233 | comp106049 | 232 | A | G | 118 | 0.779661 | 0.220339 | 114 | 0.184211 | 0.815789 | AAAGGATACAAGCCGAAACGAAGCAAGGACTGTTACGAGCAAATAACAACAAGCAAAGTGTGCTGAACAGGACGAGATCGACTTGGAACAACAGCATGAGG[A/G]TCAGTTAGCTCAAGGACCTACTTACCTGTGTTTCTCCACCTGCAATTATACCAGGAGGACTTAATGCAACTCGACTGATGACCTTGACGTCTTCTTTCTGG |
| comp104647_c3_seq4:1081-1082 | comp104647 | 1081 | C | T | 102 | 0.931373 | 0.0686275 | 119 | 0.336134 | 0.663866 | CAAGTGTCAGCCACGCAGCTGCCGACGGGCCCGCACTTCGACAGATCGCTCCCTCGCAGCATCACGGTGCAGGCGGGGAAGACGGCGAGGCTCGTGTGTCG[C/T]GTCTTCGACATCGGGGAGACATCGGTGTCGTGGATCCGGGTGGGGGACCTGCACATCCTGTCGGTCGGGAAGTACAAGTACAGCACGGACAGCCGCCTCTC |
| comp97083_c0_seq1:904-905 | comp97083 | 904 | T | G | 75 | 0.8 | 0.2 | 200 | 0.205 | 0.795 | TCCTTTCATCATATAATACACGTCAGTATCCTTTTTTCTATCTGATCATTGAATGTCTTCACTGTGTCCACTAATCACACGATATACCAAGAAGTTTTACT[T/G]TTTATCTCTTTTACACGCATGCAGTATCACCCGTTTCCACGAACAGAACTCTACGGCGAATATCCATACGTTTTCACTTACTGGACGAGTGGCCTGGCATG |
| comp106441_c0_seq1:1785-1786 | comp106441 | 1785 | C | T | 128 | 0.140625 | 0.859375 | 219 | 0.73516 | 0.26484 | TATTTGTAAGATTTCTCTCTGTGTTTTCCTTCATTTTTATTTTTTTTCTCAGTCTCGTTGTTTCTCTCCTGGTTCTTTTTGTCTCTTGTTTTGGTTACACC[C/T]TTTTCTCTCTGTTTTATCTAGTTATTCTAATCTTTATTGTCTTCTCCAACGCCTGAGGACTTTTGGTGTAGATCTCCCCCACACTATAAACTCCCCAGGGA |
| comp100298_c1_seq1:140-141 | comp100298 | 140 | T | C | 53 | 0.716981 | 0.283019 | 49 | 0.122449 | 0.877551 | TGTTTCTGCCGGGAGTGCATTTTTTTATTGACGTGTGATTATAAGTTCGATTTTTCTTTTTTCTCTTCTAAATTATTCGTTTTAATTACCCGATGTGTTAT[T/C]AACCGAGAAGTCAAGTCTAGCGATATCCTTAGTTCAGATTGTATACTTGACTGTATTTGCTATCGAGTTCGCTGCTGTTGTCTGTCGTGTCAAATTCCGAA |
| comp107170_c1_seq2:1312-1313 | comp107170 | 1312 | T | C | 619 | 0.240711 | 0.759289 | 704 | 0.835227 | 0.164773 | GAACTCTGGTCTTTAACGCTTACAATTCACAAAGAATGCATGAATATTTAACGAAAACAACATAGGAATGGACTGATTTTGTGAACAATATGTACCACAGA[T/C]ATTACTCGCGGGAAATTGGTGGTGAGGATAACTGAGTTTGTACGAACATTAAGGGAAATTGATATACTAAGAAACAAGTAGATGTAAATTGCTGTATATAC |
| comp98962_c0_seq1:4193-4194 | comp98962 | 4193 | G | A | 61 | 0.622951 | 0.377049 | 35 | 0.0285714 | 0.971429 | GTAGGTGTCTTCTCTCCATTTTCTTTCTTTGAGTCAGAGGAATCTTTGGCCTCTGGACTGTCTGTTGTCTTGGTGTCACTGTCATTCTCACCAGTTTTAAT[G/A]ATCTTCTTGGGCAATCCAGGTACCTTCTTGGGAGATTCAGAGCTCTTTTTAAAGACAATCTTCTTTGGAGGTGAAACCTCCTCCTCCGACTTTTTGTTGAA |
| comp105834_c0_seq11:1517-1518 | comp105834 | 1517 | A | C | 167 | 0.646707 | 0.353293 | 57 | 0.0526316 | 0.947368 | GAGATGCTGCTGATGAAAATGCAGGCAAAGATGGCATAAACGACTGCCCTGAGGCTATGACAGAAAGTGTTAGTGGTGGTGACACTCTCCCCAGTGAAAAA[A/C]CAGAGGAAGATGATGCGGCTGCCAACAACAGCACATTTGAAGCGACTTCGAAACCCAAGGGAAAGAAAGCTAAAGAAGCAAAGAAACGAGCACAGCAGGCA |
| comp92699_c1_seq1:919-920 | comp92699 | 919 | A | T | 52 | 0.788462 | 0.211538 | 36 | 0.194444 | 0.805556 | AAAAAAGAAACCAAAGAAAAAAAGAATATGTAAAGTTGCTTTACATTATTATCATATAACTTATTTCCTGTTTTTTTTTTCTTATAGACATTCTTTTATCT[A/T]AACACTGAATTTGAAAAGCATTAACATTGGGGTCAAGTTGGGCACCTGTGAAATAGGAACAGCAAGTGATTTTGTGACTGCAGACAACTTTCAGGCCACAA |
| comp103267_c3_seq5:1828-1829 | comp103267 | 1828 | C | G | 111 | 0.882883 | 0.117117 | 90 | 0.288889 | 0.711111 | ATGTACACCATGTAATTCTTCTCGGGGTCGAGGCCGCGGAGGCACACTTTCACCACAGGGAACATGCGTCTTCCATTTTTCGTGATTATCATTTCCGTTCC[C/G]AGGCTGTGGAACTTGGCCCACAGGTCCTGCTGCAGAAGGTGAATGCTGATGTCCCCGTCGCCCTTGCTCCCTACGGTCTCCGGCCCGACCCATCTCACGCC |
| comp107621_c0_seq1:2141-2142 | comp107621 | 2141 | G | A | 55 | 0.927273 | 0.0727273 | 15 | 0.333333 | 0.666667 | TAATGAATATTATGTTAAGTCCCACTAGTCAATTTATTAGCCTCTCATTCATTCCATTCTGTCTACTACAAAAGCATGAAATATTTGATTTCTTAATCAGG[G/A]GAACTTGTTTCTTAACCATGACAATTATCATTTGTACTACTTTACATACAAGAAAAGTATTGAACTTCTCACGTCTTGAAGCAAGATATATATTTGATGTT |
| comp107858_c0_seq1:4391-4392 | comp107858 | 4391 | G | A | 66 | 0.666667 | 0.333333 | 55 | 0.0727273 | 0.927273 | TCTGAGAGCAATTCTTTGTATGGGCTAACATCAGTGCCAGACGGAAAGGCAATAAGTGCCATACACCTTTGTATAGATGGTAAATGAGCTGGATCCTGTCC[G/A]GTGAAGTGCTTTCTTGCATGTCGAATTGCTTCCATCCTACGGTCACTCTTGATCAACTCGATAAACTCTTGCATCCGAACGTTAAATTCTAAAGAAGAATT |
| comp94106_c0_seq17:649-650 | comp94106 | 649 | A | G | 227 | 0.722467 | 0.277533 | 459 | 0.12854 | 0.87146 | ATAAAGTGTTGATAAGAAATAAAGTGTTAATAGGGAATAAAGTGTTGATAAAGAAATAAAGTGTTGATAGGGAAATAAAGTGTTGATAGGAAAGTGTTGAT[A/G]ATAAAGTGTTAAGTAGGGAAATAAGGAAACCAATAAAGTGTTGATAGGGAATAAAATGTTGATAGGGAAGTGTTGATAATAGTGTTGTGTAGGGAAATTAG |
| comp107916_c0_seq1:1392-1393 | comp107916 | 1392 | G | A | 86 | 0.662791 | 0.337209 | 29 | 0.0689655 | 0.931035 | CTTTTAAAAATCAAATTTTCAATTCAGAAAAGTTACAAATTCTAGCATATTTCCCTAACGTTCCATAACCTATCAGTATCAGCTAAATACATATCAACAAC[G/A]CAACTTTCTAGATAATCTTAGCACACTTTCTTATGGTAATCCCTTCTTGTCATTCGCATTGCATATTTCCCATTAATTCAACCCCTGCTCAGGCAAAATGA |
| comp99936_c1_seq8:2356-2357 | comp99936 | 2356 | G | C | 77 | 0.649351 | 0.350649 | 54 | 0.0555556 | 0.944444 | GATAGTGTAACTCATTTGCCCGGTCGTGTCCCAGTCCTCGTCGGCCAAGGACCCGTTCCACTTCGCCTCCCACAGCCAACTGACGTTGTAAGTGTACACAG[G/C]GCAGTTTGTGAGCGGATGAGCCCAGCTGAGCGACACTAAGTCACTAGATTCCTTGATGATGGTGCAGTTGGCTGGCATTGGGGGAACTTCACTGTCAGTAA |
| comp107620_c0_seq1:1476-1477 | comp107620 | 1476 | T | G | 647 | 0.613601 | 0.386399 | 447 | 0.0201342 | 0.979866 | TAAAATACATCTTATTCTTTATCTTTCATTATTGTGACATAACTTTGCTAACACATCTAATATAAAAGATAAATGTAACATCATTTTGAAATTTGTACCTT[T/G]CATATTACATTTTCAGTTTAGGCTATGATAACAAATACTGGTTTAATGAAAATGTGTGTACTATAATTATCTAATCAGTTATCTCATTTGTACATCATCTC |
| comp107466_c0_seq7:2697-2698 | comp107466 | 2697 | T | C | 794 | 0.745592 | 0.254408 | 670 | 0.152239 | 0.847761 | TCGACGGCCAGAGACCCTGTGCGGGCCTTCTCACCAAGTGCTGCAATGCCTCGCTCAATGTCCAGGTAGTTCATTCCTTCCACGTTGCGCAGCACTGGCAC[T/C]ACGAGGCCCTTTGGAGTAGCCACAGCAACCGAGATATCTACGTAGTCCCTGTAGATAATTTCTGATCCTTCAATCACAGCATTGACCGTTGGCTGCTGTGT |
| comp100224_c1_seq2:717-718 | comp100224 | 717 | C | T | 29 | 0.793103 | 0.206897 | 60 | 0.2 | 0.8 | TGCCCATTTGTTATTCACGGTTTTTGTGGTTTTTGTTATTTTTATGTCATTATCTTGGCCGCATTTTAAAGTTCTTGACACTCTTTAGCTTTTATTGTTTA[C/T]AACTAGTTTTCAACTTCATATTTTTGTAACCGGAAAGAAAGATAGCTAAGCATGTTTTTTTTAATCAAAATTCACCTTAGAAATGAACGACACCTTAATAT |
| comp107628_c0_seq2:571-572 | comp107628 | 571 | T | G | 206 | 0.839806 | 0.160194 | 158 | 0.246835 | 0.753165 | ACAGATCTACTAATATACTTCCATATATGCCATTTATCATGAATATATCACTGTCAAATATATATATCAACAGACCTCGACCCAGTTATTTAAAAATATTT[T/G]GGAGATTCACAAAAGTTTAAATGGTTGCCCTTTAGAGGCTGGAGGGAAGAAGCTCAATAATGATTGAGTAAAATTGCAGTGATATTATAACATGGATGACA |
| comp106227_c0_seq1:1044-1045 | comp106227 | 1044 | A | C | 46 | 0.782609 | 0.217391 | 58 | 0.189655 | 0.810345 | GTAGCGGGCGAGGGTTTCGAACACCATGGAGACGCCCACGTGGCTCTCCCCAAGACCGCCCAGTTCTTTTGGCACATTGAGAGCCAGCAGATTCATCTCCG[A/C]CAGGGCATTCAGGGACTCTCGGGGGAAGGTGTAGTTCTTGTCGTAGTCGGCAGCACGTGGTCTGATCTTGGTCCTGCACAGCTCGATGAGGTCGTCGCGCA |
| comp99336_c0_seq3:159-160 | comp99336 | 159 | A | G | 79 | 0.696203 | 0.303797 | 58 | 0.103448 | 0.896552 | TTTGTCTTCCTACGTCAAACTAGAGGATCCATCTCCCGCAGGTTTATGAGAACAAACGTGGATAGCGATACTGACCAGAGTGGCTCCAACAACGGCGTCAA[A/G]GCCATGGATACGTCTGAGTGGCTGGAAGAGACGGGAAAGATGCACATCCACCGCGCTGACATGAACAAGCTAATTATGAACTACCTCGTCATGGAGGGTTT |
| comp100537_c0_seq7:649-650 | comp100537 | 649 | A | T | 353 | 0.603399 | 0.396601 | 93 | 0.0107527 | 0.989247 | TATATTTTTTTTCATTGCTACACCTCATCTCAAATGAAAAAGAAAATATTCTCATGTCTTAATAAAACTTCCATTTTATAGAGCTGAAATGTTTATAAACT[A/T]ATATAGACCTTTTTATTTAAATCTTTGCTGTAGCATTGCCTGCCTTACATATTTTTTTTCGTTTTTTAAACATATTCTTTTGAAACCAAAATGTCAAGACA |
| comp107286_c0_seq3:1086-1087 | comp107286 | 1086 | T | C | 288 | 0.732639 | 0.267361 | 250 | 0.14 | 0.86 | GGTAAGAATGTCGCCAACCCAACCGCCATGTTGCTTGCCTCGGCAAACATGCTTAACCACATCAACTTGCAGTACTATGGCGACATGATCAGGAATTCAGT[T/C]GACCGAGTCATTCGAGTCGGGAAGGTGAGGACGAAGGATATGGGTGGTTTTGCCACAACCACAGCCTTCGCAAATGCAGTCATCGCCAACCTCCAACAGTG |
| comp101475_c0_seq2:1110-1111 | comp101475 | 1110 | G | A | 106 | 0.801887 | 0.198113 | 43 | 0.209302 | 0.790698 | TCAGAGACTTTGACAAAGCACAAGAGTTTCTTCATAGGTGCAGGAATATACCTTATTCTTGAGAAATTGAAAACACTTGCTTTCAGAAACCTATTTAAGAA[G/A]GTGTACCTCATGCTTGGAACCCACCAGATTGAGATTACGTACTTCCTGCAGAGTCTCAAGTGGATGTGTGTTGAAGACGTGGACTTAGATGAGACAGAATG |
| comp103437_c2_seq23:1657-1658 | comp103437 | 1657 | C | T | 57 | 0.789474 | 0.210526 | 66 | 0.19697 | 0.80303 | TCCCATAGTTGATCAAATTAAGAGGCTTCACATTTTTCTGCTAAAATAAAAAAGCAAACACATACCTTAAATATTTTTATATCTTGGAAAGTTGAAGTCAA[C/T]TTCAAATAAACATTCCTTAACTATGTATGTCAAGCATGGAAAAGGGGATATCTAAAATTGAATGCAAGAAGAAAAGAGTACCCCACTATACCATTCTTGAT |
| comp105169_c0_seq1:1949-1950 | comp105169 | 1949 | T | C | 120 | 0.941667 | 0.0583333 | 63 | 0.349206 | 0.650794 | AAGTCAGCAATAGCAATGAGATCCATTTTGGGCAATGGATAGGCAATGTTGAAATAGTCCTTGTAGTATGGCAAAACCTTTGTCGCAACCTCTAGGGCAAA[T/C]TTCCCTTGTTCCTTCTTGCCAACGGGAGTATATACTCGCACTTTTATCCCATCAGTAGAGGTGTCCTCCACGAAGTCATACTCTCCAACCACAACAGCAAC |
| comp100970_c0_seq1:619-620 | comp100970 | 619 | A | G | 80 | 0.875 | 0.125 | 46 | 0.282609 | 0.717391 | AACAGGTGTATGATGATATAAGAGTTTATAAACAAAGGATGTCCTTGCAAGCTCTTTGTTATGGGAATATACCTGTGATATGAATTATTTATTTTAGATCC[A/G]CTAATATTTCCGAACTGGTTGTCTCCTAAGAATTAATATTACGTGTTTTCTAGATGATTTCTTTTTATAAAGTTTATTAATGACAAAGATTTTATTATATG |
| comp99630_c4_seq2:298-299 | comp99630 | 298 | G | A | 189 | 0.888889 | 0.111111 | 172 | 0.296512 | 0.703488 | TACACAAGCAGTCACACTTCCCATTGCTTTAAAAAATTATGACATTTATGCAAGTAATATTCGTTACCACTGTCTCCGTTTCCCATGTGTAAAACCACATC[G/A]TTTTCAAGTACGCTGGAAGTAAATAATGACTTATTAATAATGTTATGATGATTGCCAGGGTGGAGATATATTCCCGTCTTTCAAATGTCTAGAGAACTCGA |
| comp99358_c0_seq8:238-239 | comp99358 | 238 | T | G | 71 | 0.901408 | 0.0985916 | 55 | 0.309091 | 0.690909 | TCCCCTTGCCTTCTTCCTACCTCCATTTTGAAGAGATCCCTTCTCTCCTGGAGCTGCTGCAGCCTTCTTGAGGACTGCCATTCTCTTTACTGTGGGTACAG[T/G]AGGTTTCTTTTTCCTTTAAATTTCCACTGCTGTCTTAATAGCTTACTGCTACACAATTTCAAGTAGCTGTCTAATTATAACCTTTACTTATTTTATTTATT |
| comp99777_c0_seq2:247-248 | comp99777 | 247 | G | A | 83 | 0.795181 | 0.204819 | 69 | 0.202899 | 0.797101 | TCTGCTCCAATTTGACTGATGCGTCAGCTATGGTCCATAGTCTGTGTGGTCTGTTTCAGTGCATAAGCGCACCAGCTGTAGCCTGCTCCAACTCAGCTGGT[G/A]CACTATCTGCTGTGCGTTGTCCGTTTTGTCGTAGCAAATGCGCCGGCTGCAGTCTGCTCTAAGTTAACTGATGCACCAGCTGTAGTCTGTTGTATTTCAAT |
| comp104130_c1_seq3:1368-1369 | comp104130 | 1368 | T | C | 584 | 0.732877 | 0.267123 | 320 | 0.140625 | 0.859375 | AAACTGGCCGAGAATGCCGAGAGGCTGGGCAACATTCTGCGCTCAGAGCTGTCCAAATTGCCTCAGGAAGTGGTGTCTGTCGTTCGTGGCAAAGGACTTCT[T/C]GATGCCATTGTCATAAACAAGAATTTCGACGCGTGGAAGGTATGCCTGCGTCTCAAAGAAAACGGACTGCTGGCCAAACCCACTCACGGAGATATCATTAG |
| comp101532_c3_seq1:291-292 | comp101532 | 291 | C | T | 49 | 0.77551 | 0.22449 | 60 | 0.183333 | 0.816667 | ACAACATCCTCTCAGCTGCCATATTGGGTACAGACCGACCTGTGGGAGCTCTTGCAACGCCATGGTTCACGTCTTCATTTCGACCTTTGTAACTCTGCTCT[C/T]CCTCCCCGCGTCCTCTGCCGACGTCTGCGGACATGCAGGGGCGGGGAGAGGCGTCCTGCAGGGTCTGGACTTCCTTGGACAAGAGGAGAGCTCGCTTGATC |
| comp95149_c0_seq1:182-183 | comp95149 | 182 | C | A | 74 | 0.851351 | 0.148649 | 54 | 0.259259 | 0.740741 | GCATGCAATTCTCATCTGTATAATGCCGTGTCTAAATGTAAAAGATAAAGAAAAAAAAAAATAATAATAATAAGGATACAGACATAAATGAAAATGAAAAT[C/A]AAATCAAAACAAATGTACACATCATAGTCAGTCAAAATAATCAAAATACATTATTTGCAACCTATGAGAATGTTTTAGCACATATTTTTTGGTCAATTAAT |
| comp100402_c0_seq1:1439-1440 | comp100402 | 1439 | G | A | 286 | 0.93007 | 0.0699301 | 139 | 0.338129 | 0.66187 | AGCTTAAGGAACCCAGAGGTGTTGTCCTGGATGAGCTTGGTGGTGACGCGCAGGTCGGCGATGGCACTTGCGACATCCCCCACAGCCTGGTAGGCCTCCAC[G/A]CGCAACTCACGGAGGGAAGCGTCCCAGGGGCATCGCTCAATGATGTTCTTCAGGAGGAGGATAGCATGCTGATAACGCCCCGTTCTAACACTTGAGTAAGC |
| comp105149_c4_seq2:411-412 | comp105149 | 411 | C | A | 16 | 0.1875 | 0.8125 | 204 | 0.779412 | 0.220588 | TCCTCACCATCAAAACAGCTAAAGAGAGAGAACGCTTAGTTCACAAAGTAAAATAGGAAAAAGTATTTTGTTAATATTATGTTTGCTTGCTTTTTGGGTGA[C/A]GTCTTGTAGGTTTTATTCCTTACTTGTTTAGACTATTTGTTTGTTTGTAACATTTTGCGATTTTGTATATTTTTTATAGTTAGTTTTATGTTTTTTTTCTA |
| comp97602_c2_seq14:615-616 | comp97602 | 615 | A | C | 261 | 0.800766 | 0.199234 | 225 | 0.208889 | 0.791111 | TCAATTTGAAAGAGTTACCATTATTTTATTTTTTCTATGGGAAGATACCTTTTTTTAAACAAAAAATGACAGTAGTGATTTATTTACCCGATATTTTACGG[A/C]TTGGTTAACCGCCTATTCTATTATTTCATTTGACATCATTATCACTTGATGAAGAAATTAACTTCGATACATTTTGTTTGTAGAAAAATTTATTGTCTCCA |
| comp103058_c0_seq1:429-430 | comp103058 | 429 | G | A | 70 | 0.857143 | 0.142857 | 49 | 0.265306 | 0.734694 | GGGGAAGTACCACCTATATCAAGTGTTAATCTTCTATGTTGAAATCAGTGGATTTTCATGGGGTCCTCTTCGAAATAAAAAGGTCCTCTCGGCTCCAGGTA[G/A]CGGGCGTAGGCGCAGCACTGCCTGTACCGTGAGCAGCAGGATCGCCACTGGTCTGGGGAGAGAATGGCGCAGTATCTGGGTCTGCATCTCTTCGCCTCTTC |
| comp82970_c2_seq1:667-668 | comp82970 | 667 | T | C | 61 | 0.803279 | 0.196721 | 52 | 0.211538 | 0.788462 | AAATTTTCTATTGCTGGTAATTGTGAAAAGTCGGGCCATATGTTGTCATTTGGTGTTCCTAAAAGATTTATGATCAGATTGATTTGTTCTATTTCCGACTT[T/C]CCCGGGAGCAGTGGCTTGTGTAGCAGAAGTTCCCCAAGGATGCATCCAGCTGCCCACATGTCGACTCCTGTTGTCTGTGTCTTAGCCTGGAAGAGCAGCTC |
| comp106287_c0_seq4:2456-2457 | comp106287 | 2456 | G | A | 80 | 0.6375 | 0.3625 | 109 | 0.0458716 | 0.954128 | AAGTTGAAATAATTGTACATGTTATCAGAGAATCTTTGGAAATTAATGCAGGCAAATATGGATTAGTGTCAAGGACATTTGTCTTTTTTTTCGTTAATGAA[G/A]ATGCTAAAATTTGCAAGCTTTAAATATGGTGCATTGTTGTATATTGGGAAAACATAGGAAGAAGTGAGAGATAAAGTCTCTGGCAGAAAGGCACTTCAGTA |
| comp101909_c2_seq2:242-243 | comp101909 | 242 | C | A | 340 | 0.811765 | 0.188235 | 168 | 0.220238 | 0.779762 | TCGGATAATTTTAGAATGAATATGTCAAGTTCCTTTTAGTTCATGGAACAACAATAACAAGAAAGCCGTGTTTATCGTTAACGAGGTCAAATTTGCATTCT[C/A]TTCTATCAACTTTATTGGAAAATTGCACAGATAATCAGACACAGAATTCAGAACGAGGCGGACATATCCCGGTGAAAATTAACCTTAACTCGATCTATAGA |
| comp103620_c0_seq1:3221-3222 | comp103620 | 3221 | T | C | 59 | 0.355932 | 0.644068 | 19 | 0.947368 | 0.0526316 | GTATATAACTTGTGTGACACTTTGCAAGATGTATAAAGTCAGATTTTAGGCTGGTATTGGAAATTCATTGGGCCAAAGGTGAAATACACCAACAATAGTAC[T/C]GTCATATTTTGAGAAGTCTTTGCAGCATTTCCAATTTTTTCTGCTTGCCTTTCTTCTTTATTCAGTTTTCTTTTACACCCTTTTGCATATTCATTGCTGGA |
| comp107430_c1_seq3:1223-1224 | comp107430 | 1223 | T | C | 45 | 0.711111 | 0.288889 | 75 | 0.12 | 0.88 | CAGAATCTTGGGACCAGCAACAATCAAAGGCTTCCGAAAGTTTCTCAGTTGCTGTCTCCTCAGCAAGTGGAAGTACTGAGCAGGAGTTGTTGGATTAACAA[T/C]CCCCCAGTTAACATCTTCTCCATCCGGCCGGTCTTCCTTTGAGTCGCAGTTCTGCAGGAACCTCTCAATGTGGCATGATGAGTGTTCTGGTCCCGCTCCAT |
| comp103338_c3_seq1:138-139 | comp103338 | 138 | T | C | 139 | 0.748201 | 0.251799 | 70 | 0.157143 | 0.842857 | CTCTCTACCTCTACCTCCATCCGTCTATCTATTAACCCGTAAGAAAAAAGTTGTACTCTTTGCTATTTTCTCTTGTGTTGTCCTAGCGACGCAAAGAGGAA[T/C]TTCAATCACTTCTGTTCTGTAAGTTGCACATGGTATTGCATTAATTCTTTGCAACATTTGCTTCCCTCCGTCAGACATGAGTTCATCGGATCAAATTTCGG |
| comp107572_c0_seq1:1526-1527 | comp107572 | 1526 | T | C | 232 | 0.612069 | 0.387931 | 190 | 0.0210526 | 0.978947 | GCTGGTGCCATCATTTCCCCCTATTATGATTCGTTACTTGTCAAAGTGATTTCTAGAGCCTCAGATCTCCATGCTTCTGCTGCCAAGATGAACCGATCCCT[T/C]CGAGAATTTAGGATTCGTGGAGTCAAGACAAACATTCCATTCCTGCTCAATGTATTGGAGAATCAGAAATTCCTGAATGGCACAATCGACACAACCTTCAT |
| comp107241_c0_seq8:1202-1203 | comp107241 | 1202 | G | C | 402 | 0.619403 | 0.380597 | 421 | 0.0285036 | 0.971496 | CGCCAGTGCCTCGGGGAGTCGCTGGCCCGCATGGAGCTGTTCGTGTTCCTCTCCGCCCTCCTGCAGAACTTCTCCTTCTCGGCTCCCAAGGGCAAGGAGCT[G/C]TGCATCGAGAAGGACCCCAGGCTGCCCCTCGTCAACATGCCAAAGCCCATCGACGTTGTGATTACGAAAAGGAAATAGAAATGAAACAAGCATTTTCTTTT |
| comp107156_c0_seq5:139-140 | comp107156 | 139 | C | T | 169 | 0.846154 | 0.153846 | 94 | 0.255319 | 0.744681 | ACTCGTCTTCCTCAGTTCACACAACAATATCAAGGCCTAATAAACTTTTCTGATGAATCAGCCGTGTTTGTAAAATGTCCCATTGCATTGAACAGGTCCAT[C/T]GGATTTTCATCAACCTCCTGGCATTTGTCGTCAAACTGTGCGCCAACTGTCCACAGAGTCTCTAAGTCATATTCTTCTCTGATGTCTGGTTTCATGATGTG |
| comp96876_c0_seq1:378-379 | comp96876 | 378 | T | C | 9 | 0.111111 | 0.888889 | 57 | 0.701754 | 0.298246 | AGGCCAAAGCTGTATGCCGACGGACGCTTGCCGAGTCCGAACGCAAAGAGGCTGGCTGCGTCCTCGTCTTCGTTCGCCCGCTTGCCGAGTCCGAATGCGTA[T/C]TTGTTGGCCCTCTTGTCCATGTCGCCATCGAGGTCTCGCTTGCCAAGTCCAAAGGCGTAGTCGCGCTGTCGCTTCTCGAAGTCGGACTCCCTCTTGCCCAG |
| comp105701_c0_seq4:625-626 | comp105701 | 625 | G | A | 80 | 0.625 | 0.375 | 87 | 0.0344828 | 0.965517 | TGTACCAAGTCACGTGACGAGAACACGCTTCGGCTTCCTTATATTGCACTTCGAACGGGTGAAGACTGACCAGTTTTCTGTTCGGTGTCTCTCGTTACACC[G/A]GTAAGTATTCACAATCTGCTGAGGACCCCGTGGTGCACCGTCAGGGTACGTCCGTCTCAGAAGCATTGGCACGGGCGGCTTTGGGCGTCCTCTTCCTGATG |
| comp91228_c0_seq1:232-233 | comp91228 | 232 | G | A | 674 | 0.813056 | 0.186944 | 485 | 0.22268 | 0.77732 | ATGTCTTCGTTGTATATCGCTTTTGCCTTCTCAAAGTTGATGCGACGACGGGTCTGGAATGGTTTCTCATAATATCTGGTTAATCTGTACTGCTCAAAAAG[G/A]CCTTCTCGTCCCATTATCCGATTAACTACTCTCATTCCTTCCTCAATGTTATTATTTCGCACCAAGACCGTTTTTCCAACAAACTGGACATGCTTGTGTCC |
| comp88640_c0_seq1:163-164 | comp88640 | 163 | A | G | 803 | 0.278954 | 0.721046 | 1048 | 0.869275 | 0.130725 | AGTCCAGGACATGTGGCTCGGCGATTTCCCTTCCTCCACCTGGCCAGATGACAGACGGCTCGACAGAAGGTGATCCTGAATTCAGCGTCAAGTCATCATGA[A/G]TCGTCTCGCTATCATTAACCATAGTGGTAATGAGGCCTGTGGTAGCCATAGCCATGGCCGTGCCCATAACCATGGCCGAATCCGTGGCCGTATCCGCCGTG |
| comp107677_c0_seq1:4066-4067 | comp107677 | 4066 | G | A | 89 | 0.865169 | 0.134831 | 40 | 0.275 | 0.725 | TGAATGAGAAATCCAGTCTGTTTTTTTATGGCATTAAGTATTTACATGTTATATTCAACTTGTGCATTATTTAACAATATGGAAAACTTACAGTAATTTCA[G/A]TGCTGTCTCTCTATTGGCCACTATGACTTAATATTTCCCTTATAAAACCATATCTGCATGCAAAATTATTGATTCACAAAGAGATGATAGCTGTACATCCA |
| comp96049_c0_seq3:1525-1526 | comp96049 | 1525 | C | T | 51 | 0.784314 | 0.215686 | 36 | 0.194444 | 0.805556 | TCCTCGATGTACTTGTCATGATTATCTGTTTGTGAAGTTTGATCATCCAACTTCAACAAGTCAACGCCACTTAGCACTACTTGGACATTTTCCTCTCGATC[C/T]ACATCATCCTCTGATGTATTTCCATTAACCATCTTCTTTTTATTGTCATTCTTGCAGTTGTTTTTGTTCTGGTTATTCCGCATGTTGGCCAAGTGGTCCCT |
| comp106831_c0_seq14:1058-1059 | comp106831 | 1058 | C | G | 66 | 0.848485 | 0.151515 | 58 | 0.258621 | 0.741379 | GTGAGTCGCGACCTTCTGACGCGAACTGGAACCTCTTCAACGGAAGTGGTGCGTTGACTGGGTGTTCTCTGGTAGCCTGTGTCGTAGAGGTGCTTGTGTCC[C/G]TAGACTGTAAAGTAGACTGGCTCGACAGAGTGTGGCTTTTCGTAGACTGTAGCTCGGATATGTGCCACGAGAGAGTGCTCTTATCTGCCTGTAGTAGCGTA |
| comp97376_c0_seq1:579-580 | comp97376 | 579 | T | A | 87 | 0.747126 | 0.252874 | 127 | 0.15748 | 0.84252 | AAAATAAGTAAACATACGACATATGCTGTTTCCTGTAGTCAGAATTCCACCAAATACACTGTCTTGACAACAGACACGACACACATACCGGTTTTTATGTA[T/A]TAATGCCTGCATTTATAAATACAAGCAAGAGTACAACAATGTATATATACACAGATTATAAAATATTTAAACATACTTAATCACATAATAAATTAATACAG |
| comp95959_c0_seq2:1413-1414 | comp95959 | 1413 | G | A | 51 | 0.803922 | 0.196078 | 42 | 0.214286 | 0.785714 | TGTGTAGGATGTGAAACAACTACCCTGCAACCTTTGGGACGTGTTGTAGTTAATGGGAAAAGTGTGAAGTACCAGTTGTCACCAGGTCCTGCAGTCAGTCA[G/A]TCTTGTAACCATTGTGGCCACAGACACTCAGTTGCAGGCCCTATCTGGAATGCACCTATACATGACAAAGATTTCATTCATAAATTGAAAGAGTCCCTTGT |
| comp99267_c0_seq7:286-287 | comp99267 | 286 | T | A | 190 | 0.747368 | 0.252632 | 19 | 0.157895 | 0.842105 | TAAACCAATTCCAGCTCCATTAACACCACCTAAACCACCCCCATTTACAAGTCCAACGCCACTTCCTCCTTGTCCTCCAACAACTCCAACAGCGCCACCAA[T/A]GCCGGCTTCTCCACTAGCGACACCACCAGCGCCACCAAAGCCGGCTTCTCCACCTGCAATTCCTCCAGCACCTTCAAGTCCTCCGAGGACTTGACCGCCTG |
| comp101669_c0_seq1:447-448 | comp101669 | 447 | T | C | 52 | 0.0192308 | 0.980769 | 69 | 0.608696 | 0.391304 | AGTGATATGAAGGTATGGAAGATGCATAAAAAAGAGCATGGTTGTGTGAATCATGATGATGAAAATATGTTTTTTTGGACTTTCATGGAGGTTTTTCTAAT[T/C]CTGTAAAGTTGGCAAAATCATTGAATGTTTACTCTATTTTTTATCAAGCTTCATTTCTGTTATGGTCCTGTTTCTTCATACGAGTGTCGGGTGTATGTAAA |
| comp105307_c2_seq1:675-676 | comp105307 | 675 | T | G | 142 | 0.739437 | 0.260563 | 20 | 0.15 | 0.85 | CGTCACACTTCTGTCCACGTCATCCTGAAGGCAGCCACGTCATAACACTGTCATCTTGGGTCAAAGTCCAGTGTGAAAAGGTCAATTACACACTTGAAATG[T/G]CATGATGAGACGTCACACTTTTAGGTCATACTTCGATGTCACTTTACGAATGTTATGTTGATGTTTCGTATTATCTGAAACGCCGTTATTTTCATTGGAAT |
| comp106992_c0_seq13:1478-1479 | comp106992 | 1478 | A | G | 1241 | 0.692184 | 0.307816 | 1596 | 0.102757 | 0.897243 | CTCTCCGTCCAGTAGATAGAGGTCCTCTGATTGAAGAATTCGCCAACGACAATGCAGTCGTAGTCTCTCCCTCCCGCGCTCAGTGTTACCAGATCGAAGTG[A/G]ACGTCCGGGCCGTCGCTGGCAATGATGTGTTCTTTCCAGCCGATGCTAAAACCTGTTCCCTCGTTCTCGAACCAGACCAGGTCGTGTTTGGTCGAACCGAG |
| comp105468_c0_seq3:754-755 | comp105468 | 754 | G | A | 54 | 0.907407 | 0.0925926 | 22 | 0.318182 | 0.681818 | GCGTTAAGGGAGGTCATGTCCGTACTGACCTGAGTTCCCCCTTGCGTCGTGTTGTGCTTGCGGAGGTCAGCCGCAGAGGAATATCCAGACGTGGTACCAAG[G/A]GAGCCATTTCTTTCGGGTGGTGATGACCGTGTGGTGTCATTTTGTAATATGAAGTAAGAAAATTTTTTAAAAAGTCGAGGAACAAAGCATGGTAATTCGTG |
| comp105352_c2_seq3:653-654 | comp105352 | 653 | G | T | 106 | 0.933962 | 0.0660377 | 58 | 0.344828 | 0.655172 | ATTGGAAGTGCACTGTGATTGAAAAGGAGCATTTTAAAATGTTGCTAGATACCAAAGTCTTTGAAGGTTGTACTTTTAAGAGATTTTTTTTTCAAAGATCT[G/T]TTTGTGAGAAATAAATGAGACAAACTTTTATTATATAATATTGTGTTTAGGGTGTCAGTAGCATATATTCTGCAAAATTGTCTCTAGGGTATTATCACTGC |
| comp101759_c0_seq3:199-200 | comp101759 | 199 | C | A | 76 | 0.802632 | 0.197368 | 103 | 0.213592 | 0.786408 | CCTCCTGCCCTCTCTCCCTCTCTCCCCCATATGCTTAAATCATTCACACCTGGTCACACATGCGCACACTTATTGCGCACACATTTATGCACTAACACTCC[C/A]ATACTCACATTCACAATCTCCCTCATCCTCTTTCCCCCTCTCTGTCACCCTCCCCCCTCCTAATCCATAATCCCTCTCCCTCTCCCTGCTCACACTCTCTC |
| comp103961_c1_seq2:3120-3121 | comp103961 | 3120 | G | A | 469 | 0.748401 | 0.251599 | 207 | 0.15942 | 0.84058 | GAGAACTGACAATAATGATAACGGCAATGGTAATGATACGCAGAAGGATAACGAGCTTAGTAGTAATAAAAGTGATAATAAGATTAAGAATGATAATAAGA[G/A]TGATGATGAAAATCGTGATAATGGTGAGCATACATACAACTACCGCTGCTACTAGTAATAATAATAATAAGAATGATGTCGACTACATTTTCTTTTCTTTT |
| comp98353_c1_seq10:120-121 | comp98353 | 120 | G | A | 1432 | 0.768156 | 0.231844 | 915 | 0.179235 | 0.820765 | TGTTTTATTTTGTAAATGAAATACACATCCATAAATCTGAAGTGAGAGAATAATACATTTAGGTTCAGAGCTTCTCAAATATCCCTAACCCCAATCTTGAA[G/A]GTCACATTCTAAAAGGCTGTCTGGTGTTGGAGGAATTAGGGATGGTGGAAGGGATGCCACACTTTGCATTCTTGATGAATTTTTAAACACCACTTCCCATT |
| comp79754_c0_seq1:333-334 | comp79754 | 333 | C | A | 60 | 0.866667 | 0.133333 | 54 | 0.277778 | 0.722222 | CTCTGGACGTTGACAACTTCAGAAGAATCTTCTCTGTCTATCGATTGCGACGACTTCCTTCTCCAGCCTTCCCGAGGGTGCAGGAAAGACTTCCTAAGAAT[C/A]TGGGGCTCCGGGACGAATAAAAAATACTGTGGATCTCGGACTCCTGAGGTGTCTGTGGAGGGCAGAAGGCTTCGGGTTTTGTTCAAAACAAACAGAAAAAT |
| comp106238_c0_seq3:291-292 | comp106238 | 291 | A | T | 529 | 0.875236 | 0.124764 | 1107 | 0.28636 | 0.71364 | CACGCGGTGAATCGGCTCTGGCAACTTTCTTCAGTAATGTCAAACTTGTACTCGGCAAAAGTTGCCACTACGTCCTCTGGGTTGCTGCATGGCAAGGGGTT[A/T]TCTGCTGCTGTGATGTTCAGGATCATCTTCTTCCTTGCGATGCCGAACTTGCAGCAAAATTTCATTCCTTTAGTTGGCGGCTTTTTAGTCCTCCCTTTAAA |
| comp104079_c0_seq1:1142-1143 | comp104079 | 1142 | A | T | 379 | 0.741425 | 0.258575 | 72 | 0.152778 | 0.847222 | ACTGACCTGGAAATCTGGCGAGCTCGACTCCTGGGTGCCATACATCAAGGCTACATGGTCGATCGCAACGGTGACAAAGTTCCTCTTCGAGATGACGTCAC[A/T]TCCGGCAAGCGAGGGATCGACATCTTGGCCGACGCTCTCGAGGCCGACGCCGACCACAGCGTCAACTTCCCTTACTACGGCGACCTGCACAACATAGGCCA |
| comp99372_c4_seq3:738-739 | comp99372 | 738 | T | G | 57 | 0.649123 | 0.350877 | 33 | 0.0606061 | 0.939394 | GTATATGTTGGTGGTTTTAATACATTGTGGCAGTGGCTATTTATTTCAGAAATATACAAAGCAATCAGGATTAGTTGCAATTTTTTTTTCAATTTTTTTTT[T/G]GTTTTTATTCATAAATACCAAATGATCTTCAATTTGTTGGAAGATAATTATTACTATTATTGTATTTATATAAAAGAAGGACTTTGTAAGAAGGAATAAAC |
| comp106462_c1_seq7:264-265 | comp106462 | 264 | G | A | 238 | 0.852941 | 0.147059 | 136 | 0.264706 | 0.735294 | AGAAGAGGATTCGCAAAAATGGCCATGGCATCGAAGCACCGGGCATCTGCCCTCAAGCAACAGAACAAGAAGCATAAAGTCTTGGGGCATCGTACAAAGGG[G/A]CAGCTCTCCAAAGTCAACAAAGGCAGAGTAGCCATCTCCGCCCAGAGCAGGAGAGCCAAGAAAGAGATGAGCCGCGAGCAGAGGAAGAACCAGATGAAGCA |
| comp107788_c1_seq2:2112-2113 | comp107788 | 2112 | T | C | 121 | 0.92562 | 0.0743802 | 80 | 0.3375 | 0.6625 | CAGTCGGTGATATTAGACGAACAGGCAACTCGTGTTTTGGAAGAGGAACTGCCCATTCTAGTGCAGTGTGAGGGCGGGGTTGCAGACAAGTGTGAGAGTGA[T/C]CTGGAGTTGACGTATACCTCAAACACCACATACGCGCTCAAGAAAATGGAATCCATCCCCGTGACCTTCAGGCTGACGAACCGCGGGGAGGTTGCCTACAA |
| comp101428_c0_seq17:826-827 | comp101428 | 826 | A | G | 89 | 0.921348 | 0.0786517 | 30 | 0.333333 | 0.666667 | CTGCTGACCAGCGATTCCTTGCACAAAGAAGGTAAATCAGAAGCAAATTAAAACAGACAGAGAGCAAAGAATGCCCACTGTAACAAATGGAACTAAACTAA[A/G]TTATATAATGTATTTATATATTCCCATCCTGGAGAAATCGGCATCGCCTTCCTCAGTTACGACACAGCGGCGTTGATCAACTATAGAAGAACCCTTCAGTA |
| comp95479_c0_seq2:845-846 | comp95479 | 845 | A | G | 79 | 0.810127 | 0.189873 | 9 | 0.222222 | 0.777778 | AGGGAAAGATTTTTGCTTCGTTTTTAATCTGAAATTCTAGTACATATCGCTATGAACGACGAACTCAAATGATTTACATCATATAAAAAATACTATTTTAC[A/G]GACCGCATCCAATGGCTCATCAAGACCAGCACCCTCGGGGAGGTTGTCAAATTCTGGATTGGCTTTCTACCGACGTGCATGATCTGCAGCGCCAGAGGGGC |
| comp104612_c0_seq5:550-551 | comp104612 | 550 | G | A | 678 | 0.60472 | 0.39528 | 416 | 0.0168269 | 0.983173 | CAGTGGTTTAAGAAATTCTTTGATGCTAATTATGATGGAAGTGAATATGATGCAGTAATGGCAAGAGGAGGAGACATTTTAGGCAAGAGTAGTGCCTCAGC[G/A]CCTCGTAAAGCTGCAGCTTCCATTGGTGGACCAAGGTCAATGGGGCGGCCAGCTGGAGGTATGCGGCAAGCAGCCCCAACACGCATTGCAGCCCGACCAGC |
| comp107866_c0_seq12:3761-3762 | comp107866 | 3761 | T | C | 349 | 0.868195 | 0.131805 | 478 | 0.280335 | 0.719665 | AAGATGAAGAAGAGAGATCCAGGCACGGCTCCAAAACTGGGCGATCGAGTTCCTTTTGTGATCATTGCAGGAGCGAAAGGAACACCTGCTTATGAAAAGGC[T/C]GAGGACCCCATTTATGTGTTAGAGAACAGCTTGCCCATTGACTACGAATACTACCTGACCAATCAGCTGAGCAAACCCCTTTTGCGAATTTTTGAGCCCAT |
| comp101342_c0_seq3:1149-1150 | comp101342 | 1149 | C | T | 287 | 0.811847 | 0.188153 | 174 | 0.224138 | 0.775862 | AACCTCTTTAACTTCTCTTTTTTAAGAATTCAAAATTTTATATGGAAAAAAAATTGATTTTGCTTCCAGAACTCATTTTTTGCACAAATTGTATTAATTAG[C/T]TGTTTTGACTTTTGAGGGGTTCTGTACCTGACTTTTGTCACTTGGGTGTTATTACTTTTGCACCATAATAGTGTAGGTTTTTCATACTTTTTGTAACCATA |
| comp101663_c6_seq1:1563-1564 | comp101663 | 1563 | C | T | 182 | 0.214286 | 0.785714 | 101 | 0.80198 | 0.19802 | AAGCAACTGTGGCATTAAAGGAACAGTGCCTTTGCCAACATTAAAAAAATGATTGTTTTAATGTGATTAGTTTATACAAGTGAGGGAAAAATCATCAGTGT[C/T]GTATGTCAACATCACAGAAAACCATGATTTACAAGATAAATGCATTTGTTCACTTGACAATGGGCACTATTCTATTGACCAATAGCACCTACTCTGGCATT |
| comp100550_c1_seq1:1294-1295 | comp100550 | 1294 | A | C | 1272 | 0.775157 | 0.224843 | 48 | 0.1875 | 0.8125 | CAGTAGAACAGACTGTTAATCAGGTGGTGACTGCACCCTGCCAACAGACCCAGACTGGCTATAACTACAACGCCCCTGCCCGCCCCTTCACTTTCGGTTAA[A/C]TATTTTTTCGTACAATAAAAGTAAACCATAATAATTGTACGACGTTATACAGATTCAATCCTTTCATGAGTAGGACAAGCGTTAACTCTGTCAACATGTTT |
| comp104889_c0_seq4:847-848 | comp104889 | 847 | T | C | 65 | 0.676923 | 0.323077 | 56 | 0.0892857 | 0.910714 | ACAGCAACTTTTTATTTATTTATCATATTTTCTAACTAAAATAACAATCCCACATATTACTATAATCGTCCTAAATATCGCGACTTTGGTTTCCACTTGGT[T/C]GACTGCATCAGGGTATAGTTGAGATAAGAGCAGTGCTGTTCCGTGACGAAGGGGACGTAGAAGGCACGCAATAATCTGTGAGATCTTTGTGCATCCATAAA |
| comp101089_c1_seq21:701-702 | comp101089 | 701 | T | C | 373 | 0.718499 | 0.281501 | 252 | 0.130952 | 0.869048 | TTCTTGGGTGTGATCTTGGCTTTGGCTGTCGTCCGGTTCATAGTCTTTGTCATTATCTGGGTCCTAAGCATGGGCTACCACCACCTCTGGCTCCTTCCCAA[T/C]CTTACCGAGGATGTTGGGTTCTTTGCCTCATTCTGGCCTCTGTACCATTACGAGTACCGTGGGGAAGGTTATGAAAAGAAGAAGAAAAAGAAGAAAGACAA |
| comp107674_c1_seq1:1104-1105 | comp107674 | 1104 | A | G | 53 | 0.943396 | 0.0566038 | 73 | 0.356164 | 0.643836 | GCATGAAAGATAAATACACTGTCCTCATTTCCAATATTAAAGTTCTTTCAGGAAGGCATGCTTATTTGAGATTGTAAAGTTACAGAGAAGATTTTCCCGTC[A/G]CTCTTGTAACTAGTACATCTAGTATTAAGTATAGCACAGAAATGAAAAAATTGATTGAAAGAGAATCAGTACCACCCATGTTCACAATTAGGGGAATAATA |
| comp107725_c0_seq16:931-932 | comp107725 | 931 | A | C | 88 | 0.920455 | 0.0795455 | 24 | 0.333333 | 0.666667 | TTTGCCGTAACGACCGATGCCATTGTTACGGAACACGACCTCTACAGAGTCGACGAAGATACGATTCGGATTTGTGAAGTGTTATCTTAACCGTTAGTGTT[A/C]CCTTGTTCATGGCTAATGTTGTCCTAATTATCATTATCATTTGCCACTCTTATTGTTATCATGATTATCATCCCCATAATTATTTCCATTTGTCTGTATCG |
| comp107707_c0_seq1:209-210 | comp107707 | 209 | A | G | 239 | 0.74477 | 0.25523 | 190 | 0.157895 | 0.842105 | TAATTTGTGAAGAGTTTCCATGGCCAGGGAGAACCAAGAGTCAACTCGCAATATCTCATTTGTTTTTCCAATTTAATGTTTGCGTTTGCTTAGGGTAATTA[A/G]TGCTATATTTAGTGGTAATTAGTGGTCGGAGCTAAAGGAGATGGCATCGTCATTCTCTAGAAAAATAATGACTTTGCCCAATTCGGGATTTTAGTGGATGT |
| comp106611_c0_seq2:2341-2342 | comp106611 | 2341 | T | C | 260 | 0.634615 | 0.365385 | 251 | 0.0478088 | 0.952191 | GAGCACTCAGACCCCGTGATGTCTCTAAGCTTTCCCGAAAGTACTTCGTTGGCACAGTAATTTGGATGGACTATGGTTTCGACATTGCCTTCAACTATGTT[T/C]TGGGTCGCATAGAAGAGTGGATACATCTCATCATTCGGATATCCAAACTCGTCGAACACACCCCAACGTAACTTCGCCCACTCGTGAACCAAAGTCTTGCT |
| comp102574_c1_seq2:389-390 | comp102574 | 389 | G | T | 6097 | 0.752993 | 0.247007 | 4974 | 0.166265 | 0.833735 | ACGAGAATTCACAGACACACAGATGATCATGCTCTCCGAATGCGACGGCGTTGTGTCCAAGAGGATCTACAAGCGCCAGTGAGTCTGTGGCGGAGGAGGCC[G/T]TCCAGCCGAGCCCATGACGTCAGCGGCTTCCTTTGTCTGCACTCTTTGCTGTTCCGCTTTTCTCTGTTTCTTGCTCCTTTTCCTTTGCTCTTTGAGTTTCA |
| comp96744_c2_seq12:769-770 | comp96744 | 769 | T | C | 515 | 0.862136 | 0.137864 | 363 | 0.275482 | 0.724518 | AAAAATTAATAAAATATACAATAAAAGAACAATAATTACACATTAAGACAAGACTGAAGTGGAAACAATATATTCTTCATTAATTAATGAGGAAAACACAA[T/C]ATCCACCATACCTAATTACTTTTTTTCAAGGAAATACATTAATTCACACTGCATAAAAGATTGTTTATAATTACATCTACTTTACCAGACCTTCCAGTGAT |
| comp102758_c4_seq18:635-636 | comp102758 | 635 | T | C | 913 | 0.64184 | 0.35816 | 666 | 0.0555556 | 0.944444 | GCCGCCGTGCTCCTCGCCTCGTACGCCGTGCAGGCCAAGTACGGCGACTTCAACCAGGACTTCCATAAGCCGGGTTTCCTCGCCAACGACCGCCTTCTGCC[T/C]CAGCGCGTGTTGGACCAGCATAAACTGACCCGTGACGCGTGGGAGGAGAAGATCGCCACGTGGCACAAGGAGCACAAGGGTCGCATCAGAGACGAGGCTAT |
| comp100053_c0_seq1:221-222 | comp100053 | 221 | C | T | 18 | 0.944444 | 0.0555556 | 67 | 0.358209 | 0.641791 | ACCCAGTACATATCGTCATGGGAGACAGCCTCCCCGCCCGCCTCCACGACCTGAGCCAGGCGGGCGGCCTCCGTCAGCCAGTCCTCGTCGTCGTGCATGTA[C/T]CCGCCGACGGTCCTGGTGAACACGGTGATCTGCGGGCGGTCCTGGATGGTGACCAGCTGATCGTTGGGCGCGGGCGGGCTGGCCTGGTGATCCTCGCCGAT |
| comp105522_c1_seq12:433-434 | comp105522 | 433 | T | C | 100 | 0.79 | 0.21 | 103 | 0.203883 | 0.796117 | CACGGATCAGAGTCCCATAAGGATGATATTGACCAAGGCATCTTCGCTAAAGTGCTCTGGTCAACGTTTAAGACTGACCTCGTTTCCCACAGTAGCTTCTG[T/C]TGTGAAAAATTTCCTGAAGGTTTCACTCCGTTTCCGACACAGCGAGTCGAGAGGTCCTTTGTGGGGAATAAGGACTACAGTAAACATCCGGAGGGCTTCCA |
| comp100888_c0_seq6:1019-1020 | comp100888 | 1019 | C | T | 59 | 0.79661 | 0.20339 | 38 | 0.210526 | 0.789474 | TGAAAAGTTGGGGAGCTTCTCTCGGCGGATGGGGAAATTTCTCTCGACAGCTTGGAGCTTCTCTCGATAGATGAATCTCTCCGAAGGCTTCTCTCGATAGA[C/T]GAATCTCTCCGAAAGCTTCTCTCGACAGCGCTGATCCGGCTCTCCACTTCGCGCCTCCGCTCTCCACCGTCGCCCAGAACCCACTGAAGCTGCTTTAGAAA |
| comp102944_c0_seq10:449-450 | comp102944 | 449 | A | G | 120 | 0.658333 | 0.341667 | 96 | 0.0729167 | 0.927083 | TACTCCATGGACATCATCGAGAAAGACTACTTCGGCCTCCAGTACACCGACGCCAACCATGTCCCGCATTGGCTTGACCCGACTAAATCAATAAAAAAGCA[A/G]GTTAAAATCGGGCCGCCGTACACGTTCCGGTTCAAAGTCAAGTTCTATTCGTCCGAGCCGAACTTGCTGCGGGAGGAGCTCACTCGGTACCAGTTCTTTTT |
| comp101655_c0_seq3:684-685 | comp101655 | 684 | G | A,C | 158 | 0.85443 | 0.14557 | 104 | 0.269231 | 0.730769 | TGCGGCCCCGAGTGCGAGGCCCGCCCCGACCACGAGCCCGAGTGCCAGGTCACGAGTGCCGCCGGGGTCAACCTGGACGTCGAGATGGCCAACGCTGAGAC[G/A,C]CCTATTCACCTGTACGAGGTCGTCACTGTGCTAAGATGTCTCGTGATCGCAAGCAGAGACAGCAAGAAGTGGCGAGCAAGCACAGAGCGACTGAACACCCG |
| comp98919_c1_seq3:788-789 | comp98919 | 788 | C | T | 13 | 0.307692 | 0.692308 | 56 | 0.892857 | 0.107143 | GTCACAACTGCGGTAAGCCGCTGCGGTCACTCGCGCCCTCGAAGGATCAGCTGTTGTAATCATCACTGGTCCAAAAATCTCTTTATTTACAGTTGAAATCC[C/T]GATGGCTATGCATGCAGCTTAACCAATTTTAGTGTGCAACTACAAATTGCACATGTATTTTTTTTTTATTTGACATACATATCTTTCTTAAGATGAGCAAA |
| comp105799_c0_seq6:1361-1362 | comp105799 | 1361 | G | T | 309 | 0.320388 | 0.679612 | 148 | 0.905405 | 0.0945946 | ATTTTCTAAATGGTGTCAGATGAGGTGGAAATTGTTGAGTTTCTGTCATGTATGATGCTTTGGGACAATATTCCTTGCGAGTTAAAGTGCAGTTCAAACAT[G/T]GTCATTTCGTCATTAAGTACTTACCATATCGAGGATAAATGTAAAGTTTTTTTTTTTCTTTCTTTTTTTTATTAATCAGGTGATTTTTTCTGATATAAAGA |
| comp99170_c0_seq1:372-373 | comp99170 | 372 | G | A | 2240 | 0.895982 | 0.104018 | 1588 | 0.311083 | 0.688917 | TGCAGCAGCGACAGCCACTCCAGCAGTGCGAGTCAGGCCAGCGACGCCTCAGCGGGGAGTGGCTACGGTCCAGACAAGAAGAAGAAGAAGAACAAAAAGAA[G/A]AGCAGCTTCTGGAAGCAGTCTTGCTTTCCACTGAACTAGTCGAGATCTTACAGCAATATTCTTGTAGTTTTAAGCCCATTTTTCTGTGTGTGATAGGAAGA |
| comp107538_c1_seq2:756-757 | comp107538 | 756 | T | A | 185 | 0.875676 | 0.124324 | 196 | 0.290816 | 0.709184 | TATTATATGTGTTGTAATGCACCGAAAAATAGTAGATCCTCTATTGACCATAAGAAGGTTGAACGATGAGAGATAATAGATTACATAAAGGTAATTTATTC[T/A]TGAATATTAGAATGCATTACAATGTTTGCCTTGTGAGAGTTTCTATAAGCTTAAATAATTGCTCAAAGAGATATCTAAGTTCTTGGCTAGATAAGCACAAA |
| comp107118_c0_seq1:1145-1146 | comp107118 | 1145 | C | G | 89 | 0.741573 | 0.258427 | 51 | 0.156863 | 0.843137 | GTGCTGCCATCGGCCGCCGGGGTCCTCAGGCGCGGGAGGGCGCTCTCCCTCGCCGAGCCGCCCGAGCTGTGCACCATGAGGATCCTGGTGCTCACCCTGGA[C/G]CCCTGGTCGAAGGGGCGCAGGAGGTTGAGCACGCCCACGCCGCAGAGGACCAGCACCAGGCCCCACCGCAGCCGCCACGACCGCAGGAATCTCCACAGCCT |
| comp105393_c0_seq6:948-949 | comp105393 | 948 | G | A | 1755 | 0.593732 | 0.406268 | 443 | 0.00902935 | 0.990971 | TGCTCTGAAAACATGCAAGTGTATATTTATAGTGCGTGAGGTTTCAGTAGCAATGAGCCTTGTTGCATTATTCTTCATCGACAAAATAGTGTGAATGACTC[G/A]TGTTTCGTGTACAACTTTTGGATGTATGTGTGAGAACAAGCATGGTGTGATAGGGTTAAAGTTGTAAATTTTTCAAATGTATTTGGAAATCATTTTGTTAT |
| comp97190_c0_seq2:264-265 | comp97190 | 264 | G | C | 35 | 0.942857 | 0.0571429 | 67 | 0.358209 | 0.641791 | GCTGCAGATAAAATCCTGCACGTGTCAGCACACATGTATTCACAGGCAGGTGTCTGAGCCGAGCGCATCTGTGGATAGAGTTGAAAATGGCTTGATTTGCG[G/C]CCCCCAAAAGGCTTATTTCCAGTCAGCCAGATTGATCAGGCTGCTCGCCACCTGCGCTCGGCTCCGCGTCGCGCTCCAGGTGTTCCTTCTTCCCAGACGGT |
| comp96349_c0_seq3:1078-1079 | comp96349 | 1078 | A | G | 57 | 0.614035 | 0.385965 | 34 | 0.0294118 | 0.970588 | AATACTTGACCGTCGCAGCTCACAAGGATGGCACTCTACCAGAAGATTTAAGTGTAAAAGTGATAATGGACACCTGGACACTGCAGAAGGGCTATCCTGTC[A/G]TCCATGTCACGAGAAGTGCTGACGGTACTTCGGCTACCGTCTCTCAGGAGCGATTCCTTTTAGAAAAGAATGCGAACTCAAGTGACACACACGTCTACAAT |
| comp107837_c1_seq1:1356-1357 | comp107837 | 1356 | G | A | 97 | 0.927835 | 0.0721649 | 134 | 0.343284 | 0.656716 | TGTGAATTTCGATGATTCTTTCAGCTCTTGACGTTTTCAAACTGGTGACGTCACTTGACATTTGTTCGTGAATTTTGCCAATGTTTTCCTCGATGCGTGTC[G/A]TCTCTTTGAGGTAATTTTGCTTATCACTGCTGACTTTCTCTGACACGTTGTTCATCTTCTGTAACATCGGCTGGATTTCGACGTTCGTTTGAGCGTCTGAG |
| comp104615_c0_seq11:1546-1547 | comp104615 | 1546 | A | G | 113 | 0.938053 | 0.0619469 | 99 | 0.353535 | 0.646465 | GCTGTTGTAGGGTTTAAAAGAAGGAGTTGAACATGCATGCATTTGCTGTACACGAGAGAAATGCATGTTTTGTGTAATATAATGGTATATTATAAAAAAAA[A/G]ATATGATGTGCTTTCAGTAGTTAAACACAATTTGACAATTATAATCATTATTGTATTCCCAATAGAATATTGATTGCTCATTCATATGCTCTAAGATGCAG |
| comp106365_c0_seq2:2717-2718 | comp106365 | 2717 | A | T | 53 | 0.754717 | 0.245283 | 47 | 0.170213 | 0.829787 | AGCACTGTGAGTGAACAGAGTTATCAGGAAAGTGTTTTAACCTGTGAGAATTCTTTTCTTGAAGGAATCGCAATGTCAAGGCATGCATCAAAACCTAACTC[A/T]AGGGTCGCATCACCTGCAAACCTTAATAACACGGATCATTCTTCTGTAGAGCTCCACCCACTTGAATCTACTAGAGTAAAAGCTTCTTTCCACACTTTGAG |
| comp106765_c4_seq7:446-447 | comp106765 | 446 | T | C | 146 | 0.917808 | 0.0821918 | 9 | 0.333333 | 0.666667 | AACTCCACAGAGCCAATGAACATCAGCTGCACAACAACAGCCACGAGGTAGAGAGGGTAGGTGAGGCGACTCAGAGGCTGCCACGAAGGATGGGAAAGAAA[T/C]TCGTCGATGAGACCTGGGAAGGAGAGAAGGAGAGAAGGGGGAGAAATGTAGAGGCAATTCGGAAGAAAGAACTGGACTGAAAAGGAAACTGAATTTTTGAG |
| comp102579_c2_seq11:1405-1406 | comp102579 | 1405 | T | C | 72 | 0.791667 | 0.208333 | 82 | 0.207317 | 0.792683 | CACAAGGCCGCCGCCGACCTCGCCAACCTCCGGGAGACGGTCAGCCAGAGGGATTCCACGGTGATGGAGCTGCAGGCGAAATGGCAGGAAGCGGCAGAGAG[T/C]GTTTCCAGACTTCAGGGAGAGCTTCAGGTGAAGTCCGCCGCAGAGCCTCACCAGGAGAGATAAATAAAGAAAAAAGAAGATGGAGATAAAAGAAAATGTCA |
| comp98132_c1_seq1:867-868 | comp98132 | 867 | A | G | 46 | 0.826087 | 0.173913 | 62 | 0.241935 | 0.758065 | ATCTAAATAAGAAAAAAAAATCTTCTGAACTTGCACTATGAGATGTGTCTTTAAAAATGTGTTAAACCAAACTCTCTCCTTCATTCATCATTCGTTACACT[A/G]ATGAATGTTTTGTGTAAATGTCTCGACCTGTCCAATCCCAACACAATACACCTTTTTCAGTGATTGGTAAACAAGCTATTTTGCTTAACATTCCATACCTG |
| comp100134_c0_seq4:234-235 | comp100134 | 234 | T | C | 232 | 0.818965 | 0.181034 | 200 | 0.235 | 0.765 | CGGAATTTTGATAGTTCCGAAAAATCCTCACTCTTACTCCTCCGTTTCAATCTTCACTTCTCGGCTTCGCTCTCGTCTTTCTTCTCTTCCTTCGCTGGTTC[T/C]TCTTTCTCTTTGCTCTCCTCCTTCGGGGCCTCGCTCTTCTCTTCCTTGATTTCCTCGCCTTCTTTCTTCTCTTTATCCTCTGGCTCCAGTTCCTTCTTCTC |
| comp104969_c2_seq1:816-817 | comp104969 | 816 | A | G | 97 | 0.876289 | 0.123711 | 106 | 0.292453 | 0.707547 | CATCCCTTCTCAAACCTTTCCCTCACCTGGTCACTGTTCTCCTCTAATTTGGTAGGATCTATACCTTCTGCTAAGAGGTCTGCAAGTTTCCTTTTCTTTTT[A/G]ACCTCTTTCTTGATCTTGGGTTTTGTTTCCTGTTCTTCTTCATTTATGTCATTGGTGTTCAACGTCGTGTCTCCTTCTTGACTTTGCAAATCACCCATGTT |
| comp106193_c1_seq3:1174-1175 | comp106193 | 1174 | T | A | 343 | 0.895044 | 0.104956 | 302 | 0.311258 | 0.688742 | AGTAGTCAGTGATGGGAAATACCTTTGTGGAGAAGCTTAGGTAACTGAGAAATGAAATTTGTTTTCCTTATATGATGCCTCCAACAGTATGGATTTGATTT[T/A]TTGATCTTCAATTTGTGTAATGGTGAAAGGGGGTCAGTATTACACACAAATACAGCATATTATAAACAGACCCACACAAACATGTATACATACATACAGTA |
| comp99376_c1_seq1:836-837 | comp99376 | 836 | T | G | 127 | 0.251969 | 0.748031 | 140 | 0.835714 | 0.164286 | GCATGTATCACTCGCTCCTTCCGCAAACAATATCGAGAGGCTGAACAAGAGACCAAAAACGAAGACTTCTGCTGCCTTAGCCATGGTGACGAATGCACAGG[T/G]AGTCCTCCTACACACTGTACTTCGGTTACAACGTAGAAACTTTTGTTGGGTGGGTGCGAGCAGTGCAAGGACAGGCCATAGGGGTCCAAAGCGCCCATCTG |
| comp93721_c0_seq1:4948-4949 | comp93721 | 4948 | G | A | 89 | 0.741573 | 0.258427 | 57 | 0.157895 | 0.842105 | AGATCCCCATATTTTACATTTCTAAAATATCTAATTTTCAGACATTCATATAGCTTTTAATTCATGTCTTAGCCCATTCACAGCAGGTGATATCCTATCAC[G/A]TCATAGCAAAAAATTCATTGACACAGGTGACTTCTACTCATGTCATGAGCTTACTTTCACATAGCCAGGATGGTGCCTTGGGAGTCTAGCTGTGAGCCAGA |
| comp89653_c0_seq1:440-441 | comp89653 | 440 | A | T | 91 | 0.901099 | 0.0989011 | 63 | 0.31746 | 0.68254 | ATAGTATCTTCACCTAAACATGCCTAAATATAGGACACATGTCCCCTTTAGGATAAAGATATCTAGACCTTCTGTCTCAAGTGTAATTAGTCACTGAAGCC[A/T]AAAAATAAAACAAATGCTTAAAAAGACAGAAACCAATAATTTTACTCAATATCTAGAAATCTGTGATGTGGACTTCTAAGTACTGAGATCTCAAACACAGT |
| comp105821_c0_seq34:1025-1026 | comp105821 | 1025 | G | A | 748 | 0.762032 | 0.237968 | 353 | 0.17847 | 0.82153 | CCGAGCCTCTAGTCAAGAGACCGGACACGCTGCTGTGAGGGGGTTCCCATCAGCCCTGTCTGGTACGTGGCATCTTTCAAGCCGGGGTAAAGAACCTCGAG[G/A]GGGGAGAGTCTGGTGGCGAGGTCACCCGCGAGCCCGACGCAGGTGTAGCGACCCGGGACGAGCGGGGGGTCGTAGTCACGGTAGAACTCCATCAGGTCGTC |
| comp106112_c0_seq1:749-750 | comp106112 | 749 | A | G | 122 | 0.631148 | 0.368852 | 63 | 0.047619 | 0.952381 | GGGCCCACAGACACCATCAACTGCACGCTCAAGAACGAGTCGTCGGCCGTGCAGTTCTGGGAGAGAGGAGTCCTGAACATCGACAAGTCGGGAATGAAGGA[A/G]TTCGGCCAGATCGGAGACATCCAGGTGAAGCTGACCTTGTACCTGCTCCTGTCGTGGGTCATCGTGTTCCTGTGCCTCATGAAGGGCATCAAGTCCTCCGG |
| comp96213_c0_seq1:3560-3561 | comp96213 | 3560 | T | A | 109 | 0.743119 | 0.256881 | 119 | 0.159664 | 0.840336 | GGCCTACAGGATTTCCCAGTTTCAATATTCAATACCCAAACTGCTGTCAACAGTAGTGTGATTATAAAAAAAGATAAAAATACTAATGATTATATAAAAAA[T/A]AAAAGAACAGAAAAAAATTCTAAGCCTTTTTACAGAAGTAACATTGATCTCAGTGTTGGGCCTGTGCTCTCTTGTACGTCTTACATAATGTTCCTGTACTC |
| comp105787_c0_seq4:571-572 | comp105787 | 571 | C | T | 60 | 0.716667 | 0.283333 | 15 | 0.133333 | 0.866667 | TCCGAAGTTACTTTGATCCTCCGGTTAACGATTACGTTTGTAGCCGGAGTCAGCGTTTCGTCTGTGAATTTCAGGTTTAAGGATTCACAAGAGAAGACTTT[C/T]GGCACAGCAGTTATCACCTTAGGCAGTGCATTAATTGTCTTGATTCATCTTACTCTGCTAAGCCTAGCAAGTACGATACGCAATATTGTAATGACAGAGAA |
| comp102465_c0_seq1:910-911 | comp102465 | 910 | A | G | 2248 | 0.858986 | 0.141014 | 2890 | 0.275779 | 0.724221 | TAAGTTTTCCCGATGGATTTGATGCTCACCCACTCGTACTCCTGGGCAAGCTCGCTCATGTAGCCTTCAATCACTTCTAGCTCGTTGTATTCGTTCAGATG[A/G]ATCCCAGTTTTTCCTTGAGATTTCTCCAAGACTCTTCTCTCTTCGTCGATTTTCTTCTGAACGTCATCAATCAGGACCGAATGGGGAACGGAGAGCTCCTG |
| comp107539_c0_seq2:1461-1462 | comp107539 | 1461 | G | C | 45 | 0.0444444 | 0.955556 | 51 | 0.627451 | 0.372549 | ATTAACCTCTTGGGAGACTTTTAGGGAAAGGTCCGTCAGTTTTTTTATAATCCAGAGTGATATTCCCATTCAAAGGATCACAGTATTGTATGGCCTAATTT[G/C]TTTTAGTTTCCTGTTATTTCAAATTGTGCAATAAGAATTTTTTTTTTTTTAAATAAAGGATACTTTCCTTTATATTTCGTCTTGTAATATCTCAAGAATTA |
| comp95531_c0_seq13:926-927 | comp95531 | 926 | C | T | 55 | 0.909091 | 0.0909091 | 46 | 0.326087 | 0.673913 | TAATATTCTTCCGTGGCCGAGGACATGACCTTCCGCCTCGTGTCCTTGTTGACCCCCATGTGCATGAGGTAAACCAGGTCGTAGGGGCCCTGGGAGTACCG[C/T]GCCAGTGCCCAGTCAATCACTTTTATGGTGTGTTCCCCGTCGGTCTTCTCCTTGAAGAAGAGGTTGGAGGGCTGCAGGTCGCCGTGGATGGCCGTGGCGAA |
| comp86941_c0_seq1:662-663 | comp86941 | 662 | A | T | 88 | 0.897727 | 0.102273 | 54 | 0.314815 | 0.685185 | ATAAAAAATAAAAAAAGGTAAAAAACATCAATGAATAAATAAAAATGAATTCCGGATTACAGCTGCACCTTGCGAGGCTAGTAATCATATATCTCGCCCAC[A/T]CTCTGCATATTTCATTGTAATCACCAATAAGGCAAGCTAATGATGAAGCCGAGTCTCTGTGGATAATGATTTTAAAGATTGTAGCTCATCCCAAAATTCTT |
| comp97545_c1_seq3:414-415 | comp97545 | 414 | A | T | 100 | 0.72 | 0.28 | 51 | 0.137255 | 0.862745 | GAAAAGAAAAAAGAGAGAGAAGGCAATACGTGAAGAGGGAAAGGCAAGGGAGAGAAGGAATACATACCCAAGTTGGTATAACTGGTAATTAACAATTTTTT[A/T]AAATACCTTATAGATCTTTTAACAGTTTTAGAAGAATAATTGCAAAGATCCTTCTTTATGACAAACACATGGTCACAAAAGGTTGCAAAAATGTTGACTGG |
| comp99277_c1_seq1:169-170 | comp99277 | 169 | G | A | 107 | 0.859813 | 0.140187 | 166 | 0.277108 | 0.722892 | GAAACACTGACATCAAACCTGAGATTAGTTTTAAGTATGAGCAAAATGGGATTTCCCAGTTTTCTTTTGAACATGTTGCCACACAAAAGCAGCCTTATATG[G/A]AAAGTGTTATTTCTTCATATTATGAGTTTGCTAACACTCATTACTGCTGCTTTTGGTCATCTTTCCTATATACTGTTAAAACCACACACCAAAATTAAAAT |
| comp105878_c0_seq8:1425-1426 | comp105878 | 1425 | C | T | 170 | 0.8 | 0.2 | 115 | 0.217391 | 0.782609 | GTTGAGGAGGAGGATGACGTTCAAAGACTCAAGAGCCCAAAAGTGGCCAAAGTGACACTTGATGATGACGTGGATGGAGAGACTGAGCTAGACAATGAATT[C/T]CGCTTCATCCCAGAAATGGAAGACACCTCACCTGGATTTGGATTTGGCCCAGGCACTCGAGGGACCTTTGTGAGACCAGGTGGTGAGGAAGATGCCTTAGA |
| comp107411_c0_seq3:2464-2465 | comp107411 | 2464 | G | A | 463 | 0.799136 | 0.200864 | 254 | 0.216535 | 0.783465 | TTGACGCCACCATAGGCTGCCATGATCTCCCTCTGTCCTGCGATCTCCTTAGCCCATTTCTTGATGGAGAACTGCTCGCCGAAGCTATGCTGAATGCTGTG[G/A]GACGCGATCTCGTGACCGTCAGCATATAAGTTCTGAACCTGGGAATAGTCGGTCCACTCATGCGACACATACATAGTAGAAGTGATGGGACAACCGTTCGG |
| comp100744_c0_seq1:689-690 | comp100744 | 689 | G | A | 196 | 0.744898 | 0.255102 | 80 | 0.1625 | 0.8375 | TGGCCTCCGCAGCCACACACGAGGCGCTCATTCATGTCGATCACCTTCGCGAAGACCAAAAGCGTCTCTGGGTCTTCCCGCAGACACAGCATCGCGCAAGT[G/A]GTGGCCGACCTCTCTTCGTGGTCCTCGTGGTGGATCTCGAGCTTGTCGTAGTTCAGGAAATCCTCGTTGTAGCAGTACTTGTAGTCGAGGCAGAAAGCAGC |
| comp107951_c0_seq1:4787-4788 | comp107951 | 4787 | G | C | 83 | 0.915663 | 0.0843373 | 6 | 0.333333 | 0.666667 | AATTATGGACGTTCATGATTTCATGAGTGGAGATGTACAAGCGAAGATCGTTGACGAGGAGTTGGTCATCAAGGGACTAGTGGTGAAGAAGGAAGAAGGAA[G/C]CTCATCCGAAACCTCTCACTCCTTCAGACGCCGCTTCTCTCTTCCACAGTTTACTAAAATCACTTCTGTCATGTCTTTAGATGGCATCCTCACAGTCACTG |
| comp102621_c0_seq5:2554-2555 | comp102621 | 2554 | T | C | 51 | 0.745098 | 0.254902 | 43 | 0.162791 | 0.837209 | GCATCTTCAGAGTCACTGTCACAGCTGTTATCTTTGCCAATGGCCTCACCCTTGCTCTGATCCGGTTCTTCCACTTCACAATCTGTTGCAGCTGTCTTTTG[T/C]TTCTTCTTTGGAGATGGTGTTGTCCACAAAGATTTTTTCTTCTTTTTCTTTGATGTCTCATTGGCATTGCCTGCTATCCTCTCAATATCTTTATCTTGTCC |
| comp107824_c0_seq10:2268-2269 | comp107824 | 2268 | C | T | 153 | 0.673203 | 0.326797 | 44 | 0.0909091 | 0.909091 | ACGTGCGAGTGCCTCGTGTGCCTGGGCGGGCTGTTGGCTAGTTTGTTCTTCGTGTCTCGGGGTGGTTTCTACTTACTGAATGCAATGGACGCTTACATACC[C/T]TGGATGTACGGCTTGGTGCTTGGACTGCTAGAAGTATCCGGCCTCGTCTACCTTTACGGGACGAAGAACATCACCAAACACTTCCATGTGATGATACGAAA |
| comp100349_c0_seq3:401-402 | comp100349 | 401 | T | C | 80 | 0.8625 | 0.1375 | 107 | 0.280374 | 0.719626 | TTCGTACGAGGTCGGGGCGGACCCTGTGACCCCTAGCATGCAGGAAGCCTACAAGAAAAACGGCTATGTTATCATAAGGAACGTACTGAACGAAACCGAAA[T/C]GAGAAAACTTCGAAGAACAATGGAATGCGAAGGAATACAGAAGCACGCCTACATCACGGCGGACGGGGAAGCCAGGCAGTCCGGCTTGGTTATCTGGTTCA |
| comp106220_c1_seq1:836-837 | comp106220 | 836 | T | C | 955 | 0.597906 | 0.402094 | 948 | 0.0158228 | 0.984177 | TGCCATTCGAAGGAAGTGTGTTCTGATAGATTTTCTTTCTTATTGGAGAGATTACTCGCCGACTGCAGTAAAATTATTGTAAAAATGTGAAATGTGATTCG[T/C]ATGGAGGTTTGCTGACTTTCATAAAGGAGATTAAGGGTTACTTAAGGTACTCGATTTATGGTTCTACCTAGTCTATTAGTTTTGCTACTTTTATAAAGTTT |
| comp102555_c0_seq2:395-396 | comp102555 | 395 | G | A | 51 | 0.803922 | 0.196078 | 9 | 0.222222 | 0.777778 | TTGAGGTTGGAGAGGACAACGAGAAACTCCTCGTCTTCATCCATTGTTTCCAGTTTCATGATGAGGGTGTAGTCCGTGGCACAGACGCTACACTGCGCCCA[G/A]TAAGGAACCCAGCAGTTGACATTCTTCTCCCAGTCCCTCGCCGTGGTCAGATTTCCTGTGGTGTCTATGACGTAACGAACAAACTCTTCGAAGGTCGGGAA |
| comp106380_c0_seq1:343-344 | comp106380 | 343 | A | C | 61 | 0.885246 | 0.114754 | 56 | 0.303571 | 0.696429 | ATTGATTCCCTCTTTCAATATGCAAGAACATAACACAACTTTCTCATGTCTCTCCATTTCTGAAGACACGTCCTCTGACACCATAAGCAATCTTTATAATC[A/C]CTAAAAGATGAATACCTATTCAATTAATATAAATATACAACCTAATTTTCTCCTCTTTTACTTTTGATTTCTTTCTATAACAAACTTTTTCATATTATTTA |
| comp107851_c1_seq1:4262-4263 | comp107851 | 4262 | T | C | 143 | 0.748252 | 0.251748 | 84 | 0.166667 | 0.833333 | GTGTAGGATACTTGCACGAGGGTCTCAGTGCCAGAGACCGCAGGATGGTTGAGCGGCTCTTTGAGGCAGAAGCCATCCAGGTTGTGGTTGTGAGTCGTGCA[T/C]TGTGTTGGGCTGTGACGGCACCTGCTCACTTGGTGGTTATCATGGACACACAGAGCTACAATGGCCGTCTTCACAACTATGAAGATTATCCCATCACAGAC |
| comp105317_c0_seq18:2755-2756 | comp105317 | 2755 | C | T | 95 | 0.926316 | 0.0736842 | 29 | 0.344828 | 0.655172 | ACCAGTGAATCACAGAAATTAACGTCGTGTGACTGATAAGAGGAGCTTTACACGTAAATGTTTTGAGGTGTCTTAAGCAGCCGACTTTCAGCGGTTCAGAA[C/T]GAGTGGATGAGAAACCAGAGTTTGAATCTAGACATAGTTTCCGAAGATTTGGGCACGTTTGAAGAACCAGTGACAGGTACCCTACACATGCCTCGCCGACC |
| comp104311_c0_seq4:1076-1077 | comp104311 | 1076 | C | T | 65 | 0.815385 | 0.184615 | 47 | 0.234043 | 0.765957 | AAGCGGGGCTGCGAGGTGGAACCTGTCTCCGGAGAGTGCATCCTGGGTGGAGGTCCTGGCTATTGGCCTCAGGGACCCGGGGACTTCCCTGAGGATGGCTG[C/T]GGCGCCTGAGAGAGGAAACTAACAGCATGCTCGAGTGTTTGGAGTTTTCAGACGATGTGGAAATAAAATGTTGCAATGTAAAAGATGAAAGTAGATGGAAA |
| comp107821_c0_seq2:2518-2519 | comp107821 | 2518 | T | C | 75 | 0.88 | 0.12 | 77 | 0.298701 | 0.701299 | CAAATGGACGAACTCTCTGGCTTTATGCTCGCTGCCCTCATAAGATGTGGAATTGCTCGCACACAATCATCTTTCACATTTTTGTGGAAAAATTGCTCGTA[T/C]GACTTGCGGGCATCTGCTATCAGAGCCTCAGCCAAACCCACGAGGGACACCACCTGCACAGCTGGAATGGGGTCACGTTCCAACACTGCTTCATAATCAGC |
| comp106553_c0_seq13:2336-2337 | comp106553 | 2336 | C | T | 77 | 0.896104 | 0.103896 | 54 | 0.314815 | 0.685185 | TTTTTTTCTTTTTTTTCATGGCATTATTATTGCCAGTGTCTTTTTTCAGATTATGATAATGATAGTTTGTATATATTAGAGAGGCTTGCTAGACTTACCTA[C/T]ATTTTAAGTATTATGAAAAGTTCCATGTTGGCAACCAGTGAACAGTTTAAGTATGGAAGATATGGTACAATATACCACTTATACATATTGTAATAGGAGGA |
| comp101624_c0_seq5:153-154 | comp101624 | 153 | G | A | 9 | 0.111111 | 0.888889 | 117 | 0.692308 | 0.307692 | TCCCGAGAGTACAGGCCTCCCAGGCCTCGCTACTTCTACGATTCCAATGAATCTAAGTAAAACCCTGTTAATGAACACTTAGAATGAGTGCTACATATTAA[G/A]CAAACATTATTTATTTTAACCAATAAATTGAGCATCCGATAAATGACCTTGTGTATGAACACCCATAATGTTTCTAAGTATGTGTATAAAGGCATATACAC |
| comp106480_c0_seq2:163-164 | comp106480 | 163 | C | T | 125 | 0.936 | 0.064 | 31 | 0.354839 | 0.645161 | ATCGCTTTCTATCTTAATTAGCATTATTATATAAATATCTCGTTTCAAATTGTATATTTCATCGAGCAAATACATCAGTTATATTTCTATCCCTCATTCTA[C/T]ATTTATCGGGTAAAAATTATTGAATTTTGTATTACCTAAACAATGACAAAATAACATAAAGATAGCACCGTATTTTCTATTTCTGTTACCTTGTAGTTTCC |
| comp107711_c0_seq3:927-928 | comp107711 | 927 | C | T | 29 | 0.931035 | 0.0689655 | 60 | 0.35 | 0.65 | GGAATATATTATTATGTGAGAATATATTCTTGCAAATAAGAGGAACTCATAAAACTCGTTTATATATTATTTCCATAGGATGTGCCACGGGTGACTGAAGA[C/T]GAAGTGGCATGCTCCCTCTTTTGTTGTTGTATACATATATATCTATATATATATTTGATTGTTAGAAGGTATATATATACAGTTGTGTAGAAGAGAAAAGA |
| comp103592_c3_seq15:1256-1257 | comp103592 | 1256 | A | G | 49 | 0.0204082 | 0.979592 | 148 | 0.601351 | 0.398649 | GATACCCGAAAATCCTCGCCAAATTGGTCGACGATGGAAAAATGGGCATAAAGTCTGGCCAGGGATTTTACAACTACAACAAGGAATAATGTCGTCGAGCG[A/G]GAAAGAAAGGAAGAAAAGATGTCCAGGTGTTTTCCTTTCTTGGGGTCATAAGGATTTGTTGTTCAAATTTAGTTCATGGTATTGTTTTTTTCGTTGAAGTC |
| comp106508_c1_seq10:1387-1388 | comp106508 | 1387 | C | T | 588 | 0.789116 | 0.210884 | 566 | 0.208481 | 0.791519 | TACAAGTATCCCTGACAAAAGAGGAAGTTTCTTTGATCTAAGTACATGAAATACCTTGATGCATAATTATAATAATGATTATGAATTTACAGTTTAAGATG[C/T]TGTTTAAAAATTGTTCTCCTGGTGGTGCTTGTGATGCCAAACACTTGAGACGGTAAGACACACTAATGAGTAAATATGAGTAAAGTTAGCAGACCAAAAAG |
| comp101248_c3_seq3:422-423 | comp101248 | 422 | T | C | 70 | 0.657143 | 0.342857 | 39 | 0.0769231 | 0.923077 | CTTGATGGATGTTCCCTCTTTCATATCTGGAACCACGCAGTCAGACGACAGCGCACGACTGCTCAGGCGAATGAATCACCTGTCAGGACCCTGCTGTCACC[T/C]GATGCCTTCCTGATGACCAACCTGGTGCCTCAAGACGCCCCTCACCCTCTTTACAGCAGTCAGTTTACTGAACTCTGTAAACAATTCCTTCTCGACCATAT |
| comp102426_c0_seq1:789-790 | comp102426 | 789 | C | T | 698 | 0.815186 | 0.184814 | 553 | 0.235081 | 0.764919 | GGAGTGACTAGACCAGTTTCTCCAGGAGTTACTAACCCACTGACATCCTCATCAGATTCCTCCTCACTCTCTTCTTCTGACTCCTCTTCACTCTCACTTTC[C/T]AGTTCTCCCCACATAGTCCTTTCTACCTCCTCTTCTGTTAGCGGTGAGTCAAAATCTTTGCTCTGGGTACCAAAGACATCACCATATATAGGTTTACCCAT |
| comp105736_c0_seq2:2301-2302 | comp105736 | 2301 | C | A | 385 | 0.724675 | 0.275325 | 408 | 0.144608 | 0.855392 | GGAGTGACCTCTGTCATCTTGTTGAACGTCATCTCATAATCATTATTGAGGTTGGAGTACTGTTTGTTGGCTGTTTTGAGTGTGCATCCACTGATGAAATA[C/A]ACCTTGTCAACCTCAATCATATCCATGAACTTGTCCACCTGTTCATTGAAAGCTGTAGCCCTGATTTCTCCAGACTCATCCAACAGGTCCATTGAGAAGAG |
| comp101507_c0_seq12:3837-3838 | comp101507 | 3837 | G | A | 239 | 0.757322 | 0.242678 | 265 | 0.177358 | 0.822641 | GCCCAATACAAGCATTCCTTTCCCCTTTCTTCCTGACCTACTGGCCACCTCTGTACTGCGGGGATGAAACCAGCTTTGGCACACCTAACGCAATAAACTTC[G/A]AAAATGGTTGCTAATCATTGCTCAAACTTTTTATCTTTTTTAGATATAGTGAAGAGAGTTGCAACTAGGAAAATATAAAATAATTTTGATAATGGTAGATG |
| comp104943_c0_seq16:635-636 | comp104943 | 635 | A | C | 1214 | 0.867381 | 0.132619 | 908 | 0.287445 | 0.712555 | GGGTTCCCTGGGATACCATCATCATATGGGGAAACAATCTGGGTTGATAGTTCTGGTGGAGCTCCAGGAGCTCCGCCTGAAGTGGGTCCCCCAGCCCCTGC[A/C]GCACCACCCTGGATGATGATGTCCAGGACAGCCTTTGCAGCTGCGTGTTTTGCCTTCTTCTTGCTTTGTCCAGATCCATTTGCTGCAAACTCCCCCACAGT |
| comp105774_c0_seq1:308-309 | comp105774 | 308 | T | C | 139 | 0.741007 | 0.258993 | 31 | 0.16129 | 0.83871 | GCTGACAAGAACCTCCTGGAAGTAAACCTTCACGAAACTCACTCAATGAAGGCATGAACTGTTAATGTTGCGTCTTTCATTTTTATCGTAAATTTGTCATT[T/C]ATAGTATTCAATATTTTTGACAATGACACTAATACTCTACACGATTGAATTACTGTAATACTTTGTATGTGAGAAATAATTTAAAAGATATTGATAGCATA |
| comp101191_c5_seq1:406-407 | comp101191 | 406 | A | G | 123 | 0.934959 | 0.0650406 | 76 | 0.355263 | 0.644737 | CTTTTTATAGGTAGATATTAGACAAGTAGAATATTTTCTAGATATTTCCCGATAATTAAGGCCCCAAAAACGGGTGATCAGCGCCGACGATTGCGCATGAA[A/G]GATAGTCTTCTGTTTGTCATGTGTTCCAGTTTCATTTTTCTTAACTCTTAATACCTTGATTTAAGATTTGATTTCAATTAGAAATTCGATGGATACCAAAA |
| comp99959_c3_seq1:971-972 | comp99959 | 971 | T | C | 131 | 0.801527 | 0.198473 | 54 | 0.222222 | 0.777778 | CACTAGAAGTGCTAGTCACTATCAGACTCTCTATCCATAGTTAGGGATGTTATTCAAAGAATGCTTACACTGCATTTTTAAAATGTGTTTTGTTTCAGAAC[T/C]GAGAATTTCCAATCAAGAGTCAAAGTAATCTTATTTACCATTAGATACATATTGGAAGTGGATAAAATCACCTTCGTTACATCCATATATTTAAAAAATTG |
| comp94575_c0_seq2:403-404 | comp94575 | 403 | G | A | 181 | 0.596685 | 0.403315 | 57 | 0.0175439 | 0.982456 | TCGGGGTGGAAGTCGAGGCGGAAGAAGTCCAGGAGTTCCTTCGTCTCGTTGAAGACGTTGAAGGAAATGTCTTCGTATCGGACAGCTCTGAAGGACTCGGG[G/A]AACTTCCTCCTGAAGACCGTCGCCGTGATGTAGTCCCTGATCAGGTCCTCGCAAAGCGTCTCAGGGTCGTCGCAGTCCTTCTTCCCAGGACACCACTCCCT |
| comp95587_c0_seq1:410-411 | comp95587 | 410 | A | G | 360 | 0.666667 | 0.333333 | 274 | 0.0875912 | 0.912409 | TTCTGGTGCAGCTCCCAAGCCAGTGTCCATGATGCTCCTCCTGGGTACCCTGTATGGTGGAAGTACACACGCTTTTCCCACTCTATCCCAGGCATGGCAAT[A/G]TCACGGGAGTTTATGACAACAACATGATCTCCACAGTCATTTAGGGGATGATAAATAGGTTTTGTGCTCCCTGTAAGATAGTCCTTGATTTTATTGGCTGT |
| comp102826_c2_seq2:1446-1447 | comp102826 | 1446 | C | G | 36 | 0.944444 | 0.0555556 | 52 | 0.365385 | 0.634615 | ACCTCCTTCGCCATTTCGTGTTCGCGCGGCGCCCTGTCCAAGCTGGTGGCGCCGGACGAACTGGGCGCCGTGTTCTCCGTCATCGGGATGGGCGAGTCCCT[C/G]CTGCCCGTCCTGATCATGCCCCTTAACACCGCCATCTACAATAGCACTCTCGACGTCTTCCCTGGCACGGTCTTCCTCTTCGAGGCTGGCACTGGCGTGGT |
| comp100808_c0_seq2:517-518 | comp100808 | 517 | A | G | 342 | 0.798246 | 0.201754 | 228 | 0.219298 | 0.780702 | ATCGACTCAAAGGAGGATGAAGAATTGCGACACAGAGTAGTCAATCACGAACCACACGATGACGATCTTGCACTCAGTTCTATGGCCCAGAGTGCCTTCGA[A/G]ACTGCGGAGGAGTTAGTGCACAAGGTCTGGGAAGAAGTTTCAAGTTGGAAGACGCAACCTTTCTCAAAGCTGCCCAAGTGGCTCCAAGACAACGATTTCCT |
| comp106495_c0_seq2:1486-1487 | comp106495 | 1486 | C | G | 185 | 0.854054 | 0.145946 | 218 | 0.275229 | 0.724771 | TAATGCTGCTTCGACGCAATTCTGACTTTTCGGAATGTTTACTGATTTTCTGTCGAGATTTTTTTGATTGAAGGTGCGTCTTTGTTAGTCTTACGTAAGAA[C/G]GGAGATTTGACAAAAGATTGTTTTTCCTTTTCTTCTTGACCCTTGGAGAAGGCGTTTCGTTCGGAGAGGATAGAAGCGGGCGTGCGTTGTGTTGCGTTTCC |
[truncated: 2,926,036 more chars]
